# General Pyrrolidine Synthesis via Iridium-Catalyzed Reductive Azomethine Ylide Generation from Tertiary Amides and Lactams

**DOI:** 10.1021/acscatal.1c01589

**Published:** 2021-06-09

**Authors:** Ken Yamazaki, Pablo Gabriel, Graziano Di Carmine, Julia Pedroni, Mirxan Farizyan, Trevor A. Hamlin, Darren J. Dixon

**Affiliations:** † Department of Chemistry, Chemistry Research Laboratory, University of Oxford, 12 Mansfield Road, Oxford OX1 3TA, United Kingdom; ‡ Department of Theoretical Chemistry, Amsterdam Institute of Molecular and Life Sciences (AIMMS), and Amsterdam Center for Multiscale Modeling (ACMM), Vrije Universiteit Amsterdam, De Boelelaan 1083, 1081 HV Amsterdam, The Netherlands

**Keywords:** Vaska’s complex, amide reduction, [3 + 2] cycloaddition, azomethine ylide, pyrrolidines, polycyclic amine, density functional calculations

## Abstract

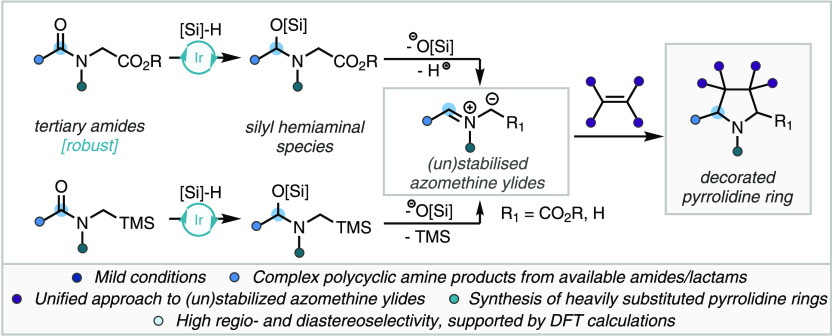

An iridium-catalyzed reductive generation of both stabilized and unstabilized azomethine ylides and their application to functionalized pyrrolidine synthesis via [3 + 2] dipolar cycloaddition reactions is described. Proceeding under mild reaction conditions from both amide and lactam precursors possessing a suitably positioned electron-withdrawing or a trimethylsilyl group, using 1 mol% Vaska’s complex [IrCl(CO)(PPh_3_)_2_] and tetramethyldisiloxane (TMDS) as a terminal reductant, a broad range of (un)stabilized azomethine ylides were accessible. Subsequent regio- and diastereoselective, inter- and intramolecular dipolar cycloaddition reactions with variously substituted electron-deficient alkenes enabled ready and efficient access to structurally complex pyrrolidine architectures. Density functional theory (DFT) calculations of the dipolar cycloaddition reactions uncovered an intimate balance between asynchronicity and interaction energies of transition structures, which ultimately control the unusual selectivities observed in certain cases.

## Introduction

**Scheme 1:**
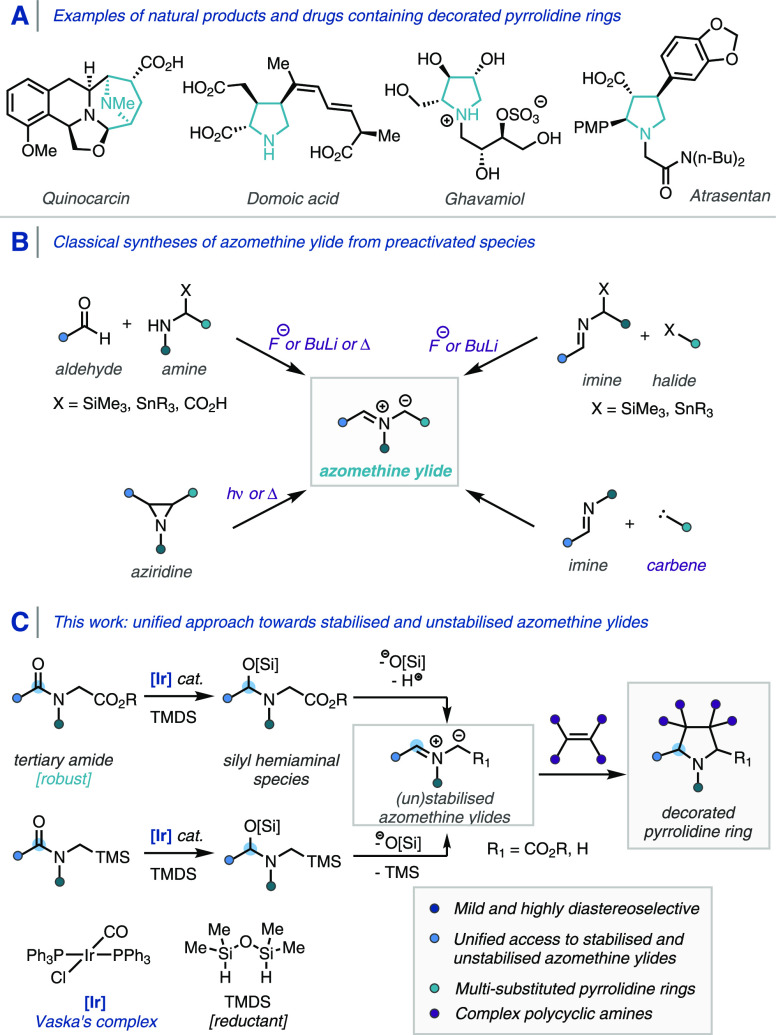
(A) Selected Examples of Natural Products and Drug Molecules Containing Decorated Pyrrolidine Rings. Ar = 4-OMeC_6_H_4_. (B) Traditional Synthetic Methods for the Preparation of Azomethine Ylides. (C) Iridium-Catalyzed Reductive Generation of (Un)stabilized Azomethine Ylides for 1,3-Dipolar Cycloaddition Reactions

Saturated pyrrolidine heterocycles are prevalent in biologically active natural products and are among the 10 most common ring systems in small drug molecules (Scheme 1A).^1−4^ Accordingly, new broad scope methods for their synthesis remain important. While relatively simple pyrrolidine derivatives are commercially available, polysubstituted pyrrolidines generally require synthetic effort. To this end, [3 + 2] dipolar cycloadditions of azomethine ylides are synthetically powerful, allowing the direct construction of the saturated five-membered ring system with control over up to four newly formed stereogenic centers in an atom-economic reaction.^5^ Consequently, the synthesis and reactions of azomethine ylides have been the focus of a number of research efforts over the years (Scheme 1B). These dipoles can be prepared from the opening of an aziridine ring^6i^ or more commonly from the activation of an imine/iminium ion species (usually accessed from the condensation of an aldehyde and a primary or secondary amine either *in* or *ex situ*) and are especially useful for the synthesis of pyrrolidines unsubstituted on the nitrogen atom.^5c,6s^ Other methods also exist, requiring the construction of finely tuned precursors.^6aa^ Notwithstanding these many advances, to date, a general reductive strategy for azomethine ylide 1,3-dipole generation from tertiary amides and lactams enabling downstream access to desirable pyrrolidine structures remains unsolved.^6af^ Toward this end and building on our program on reductive manipulation of amide functional groups,^7^ we reasoned that iridium-catalyzed hydrosilylation of suitably functionalized tertiary amides and lactams could provide a new entry point. With substrates possessing a suitably positioned electron-withdrawing or a trimethylsilyl group, following partial reduction and subsequent silanoate elimination, concomitant deprotonation or loss of a trimethylsilyl group adjacent to the iminium ion could feasibly generate the synthetically versatile azomethine ylide (Scheme 1C). Subsequent cycloaddition reaction with dipolarophiles would then give access to the decorated pyrrolidine ring in a convenient one-pot process. Such a strategy would potentially provide an avenue for the late-stage synthesis of highly functionalized pyrrolidines from stable and widely abundant amides, under mild conditions, while eliminating the need for handling sensitive amine functionalities. Herein, we wish to describe our findings.

## Results and Discussion

### Optimization Studies

**Scheme 2:**
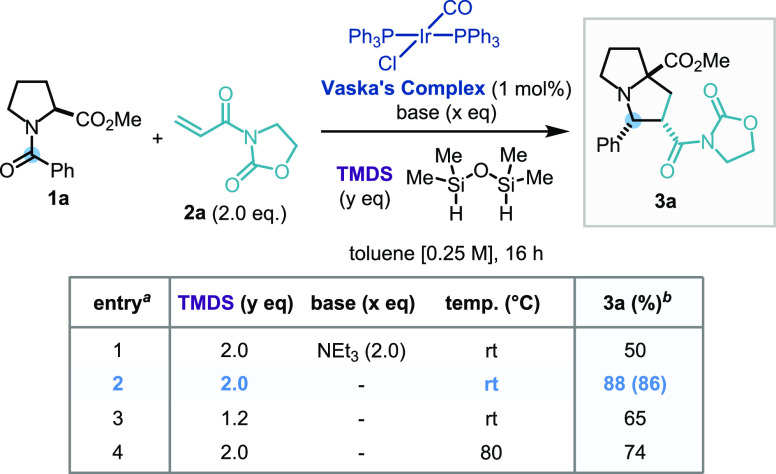
Iridium-Catalyzed Reductive Dipole Generation and Reaction Optimization Studies General conditions: **1a** (0.25 mmol scale), IrCl(CO)(PPh_3_)_2_ (1 mol %), additive, 1,1,3,3-tetramethyldisiloxane (TMDS), toluene (1 mL), rt, 16 h. NMR yield calculated with 1,3,5-trimethoxybenzene as an internal standard; isolated yield in parentheses.

Proline methyl ester benzoylamide derivative **1a** was chosen as a model system to investigate the transformation, alongside oxazolidinone dipolarophile **2a**, selected for its previous use in [3 + 2] cycloadditions and for its easy downstream derivatization.^8^ Using 1 mol % IrCl(CO)(PPh_3_)_2_ (Vaska’s complex) and 2 equiv of tetramethyldisiloxane (TMDS) for partial amide reduction, plus additional triethylamine as a Brønsted base for the generation of the dipole, we were pleased to isolate the desired product **3a** in a 50% yield as a single diastereoisomer, indicating the formation and subsequent stereoselective cycloaddition of the azomethine ylide (Scheme 2, entry 1). Notably, a control experiment revealed that no additional base was required for the reaction to proceed (entries 1 and 2), indicating that the eliminated silanoate was indeed a competent Brønsted base for dipole generation (see Scheme 1C). The use of 2 equiv of TMDS was optimal (entries 2 and 3), and the reaction could also proceed at higher temperature, albeit in a slightly reduced yield (entries 2 and 4). Therefore, entry 2 was chosen as standard conditions for assessing the scope of the reaction.

### Scope Development

**Scheme 3:**
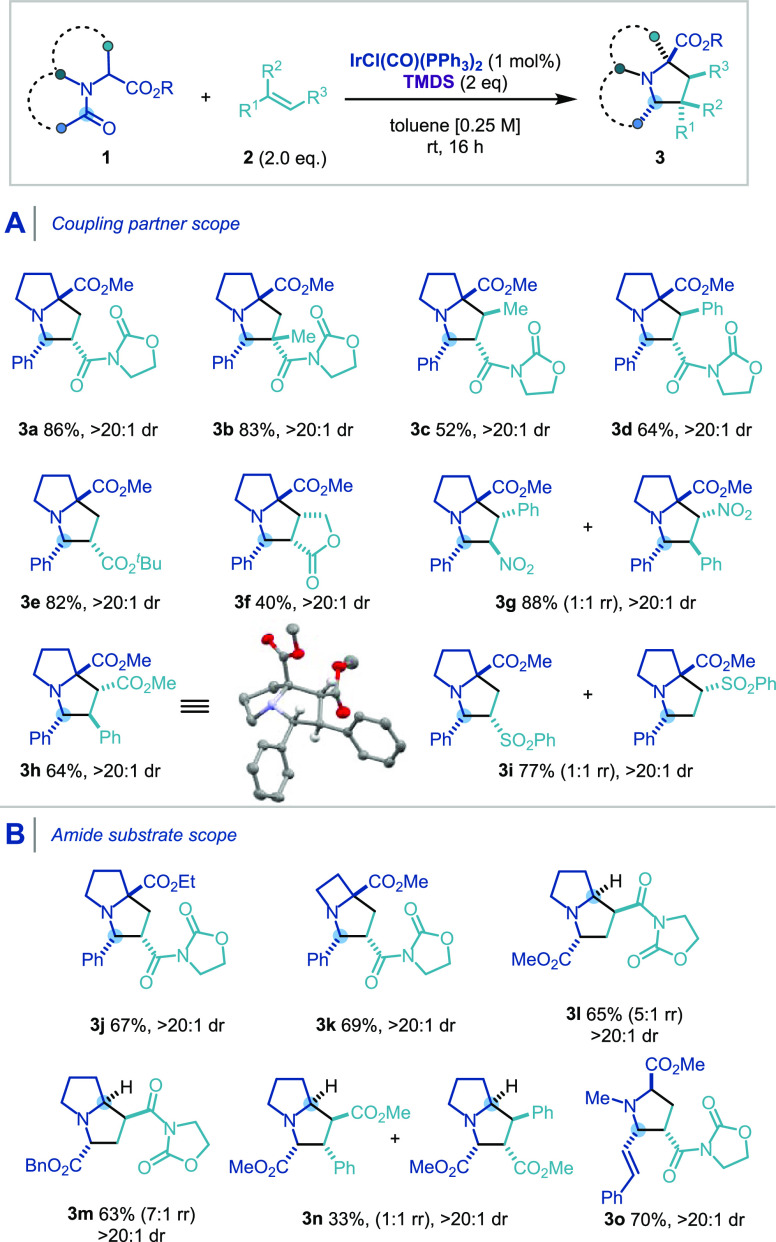
Substrate Scope of the [3 + 2] Cycloaddition via Stabilized Azomethine Ylides with Regard to the Dipolarophile (A) and the Amide or Lactam Substrates (B) Standard conditions: **1** (0.25 mmol), **2** (0.5 mmol), IrCl(CO)(PPh_3_)_2_ (1 mol %), TMDS (0.5 mmol), toluene (1 mL), rt, 16 h; yields of purified product following flash column chromatography. CCDC number for **3h**: 2056518. dr: diastereomeric ratio. rr: regioisomeric ratio.

With the optimal reaction conditions in hand, we explored the scope of the [3 + 2] cycloaddition reaction with a range of electron-deficient alkenes as coupling partners. A 1,1′-disubstituted methacrylic acid derivative successfully underwent cyclization to give a cycloadduct possessing a quaternary carbon center (**3b**), while crotonic and cinnamic acid derivatives gave rise to the corresponding polysubstituted products bearing four adjacent stereocenters in good yields with high diastereoselectivity (**3c**, **3d**). Importantly, the reaction was not limited to *N*-enoyl oxazolidinone coupling partners; acrylate, furanone, nitroalkene, cinnamate, and vinyl sulfone derivatives (**3e**–**3i**, respectively) all produced the desired cycloadducts, with a large diversity of substitution patterns on the pyrrolidine ring, and in good to excellent yields. Interestingly, the regioselectivity of the formation of **3h** was reversed when compared to **3d**, as confirmed by single-crystal X-ray diffraction analysis, while **3g** and **3i** were formed as a 1:1 mixture of the two regioisomers (in line with the literature precedent) (Scheme 3).^9^

Pleasingly, the reaction sequence was found to be general with respect to the amide substrate. The presence of a methyl ester was found not to be a limiting requirement (**3j**, **3m**), while the azetidine-containing amino acid derivative afforded the corresponding [3,2,0] bicyclic compound in moderate yield (**3k**). Importantly, this methodology could also be applied to lactam substrates, providing bicyclic cycloadducts containing a tertiary stereocenter adjacent to the nitrogen atom, which would otherwise be inaccessible from other azomethine ylide generation methods (**3l**–**3n**).

This also demonstrated that the methodology was not limited to the reduction of reactive benzoyl amides, therefore increasing the diversity of scaffolds made accessible. A range of 2-carboxy-substituted pyrrolizidine was thus obtained with a 4-carbonyl (**3l, 3m**) or 3-aryl 4-carbomethoxy (**3n**) substitution pattern. Cinnamoyl amide **1o** also gave rise to the 4-alkenyl trisubstituted pyrrolidine **3o** in good yield.

**Scheme 4:**
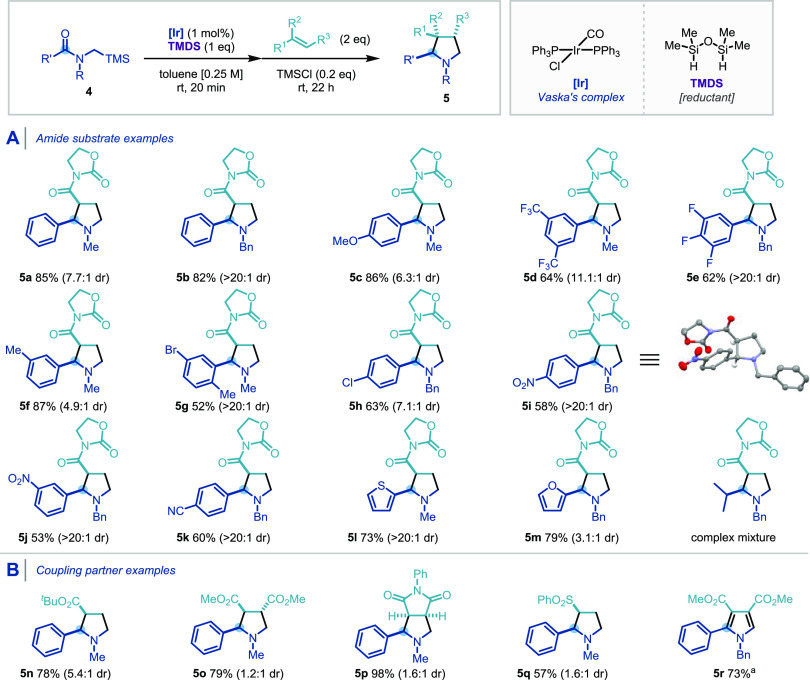
Substrate Scope of the [3 + 2] Cycloaddition via Unstabilized Azomethine Ylides with Regard to the Amides (A) and the Dipolarophiles (B) Standard conditions: **4** (0.3 mmol), **2** (0.6 mmol), IrCl(CO)(PPh_3_)_2_ (1 mol %), TMDS (0.3 mmol), toluene (1 mL), rt; yields of purified product following flash column chromatography. Overall yield following the oxidation by DDQ (2.0 equiv), 80 °C, 16 h. CCDC number for **5i**: 2056517.

Having established that amides and lactams possessing a β-ester appendage on the nitrogen atom were excellent precursors to stabilized azomethine ylides, we next turned our attention to unstabilized 1,3-dipole generation. Using *N*-(trimethylsilyl)methyl amides as substrates, and following standard conditions for Vaska’s complex-catalyzed hydrosilylation, with substoichiometric amounts of TMSCl as an additive to trigger the desilylation, we were pleased to observe [3 + 2] dipolar cycloaddition of unstabilized azomethine ylides taking place. This approach is complementary to the one described above, as the resulting pyrrolidine ring of the cycloadduct bears no substituent *a* to the nitrogen atom. As shown in Scheme 4, the reaction was found to be tolerant of a good range of aryl and heteroaryl amides, although alkyl amides remained difficult substrates due to the rapid formation of reactive enamine species. Pleasingly, diastereocontrol was improved by increasing the steric demand of the substituent on the amide nitrogen (R) from methyl (**5a**) to benzyl (**5b**). Aryl amides containing both electron-donating (methoxy, methyl) and electron-withdrawing (halides, nitro, and nitriles) groups afforded the corresponding pyrrolidines with good to excellent diastereoselectivity (**5c**–**5k**). Heteroaromatic amides also underwent cycloaddition smoothly (**5l**, **5m**). Alkyl amide substrates gave a complex mixture, indicating a lack of regio- and diastereocontrol. Several other dipolarophiles could also be used, leading to products derived from *tert*-butyl acrylate (**5n**), dimethyl fumarate (**5o**), *N*-phenyl maleimide (**5p**), and phenyl vinyl sulfone (**5q**), although a reduced diastereoselectivity was observed (Scheme 4B). Pyrrole **5r** was obtained in a good yield using dimethyl acetylenedicarboxylate as the dipolarophile under standard conditions, followed by the oxidation of the resulting dihydropyrrolidine ring by 2,3-dichloro-5,6-dicyano-1,4-benzoquinone (DDQ). This process can provide substituted pyrroles in a one-pot sequence from inexpensive and robust tertiary amides.

### Intramolecular Cyclization

**Scheme 5:**
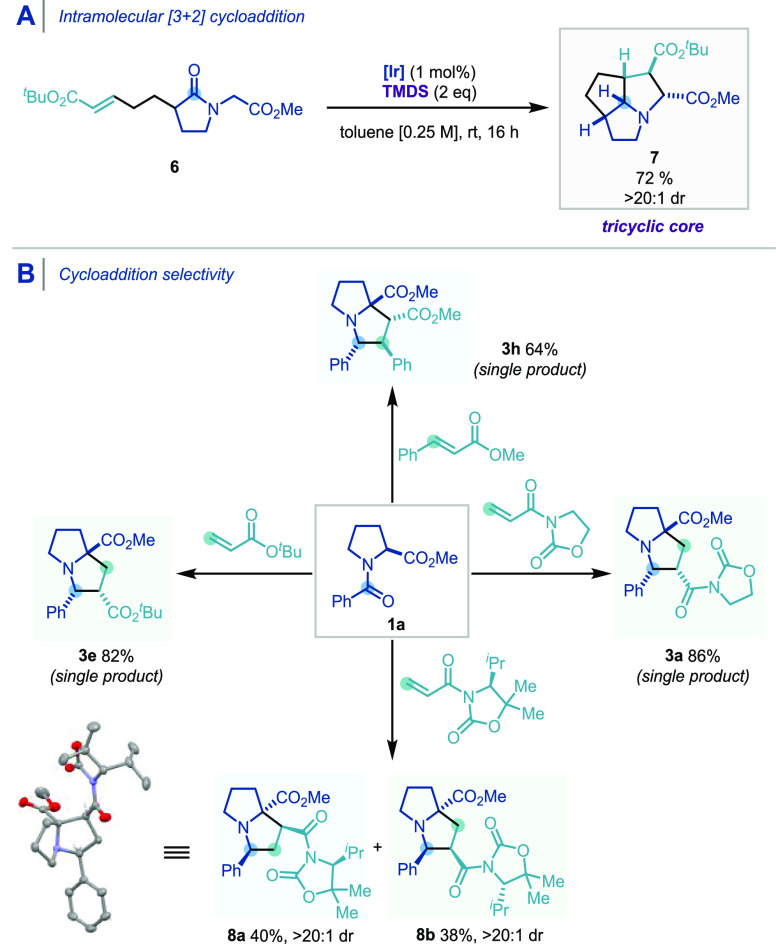
(A) Intramolecular Reductive [3 + 2] Cycloaddition. (B) Regio- and Diastereoselectivities of the Reductive [3 + 2] Cycloaddition with α,β-Unsaturated Carbonyl Compounds CCDC for **8a**: 2056519.

To demonstrate potential applications of our methodology, a linear substrate for an intramolecular cyclization was synthesized and subjected to the reductive cycloaddition using the optimized standard conditions (Scheme 5A). Pleasingly, a remarkably chemoselective reduction of the amide carbonyl **6** was achieved, leading to the formation of the tricyclic 1-azatricyclo[3.3.0.2^4,6^]decane core **7**, via the putative azomethine ylide, as a single diastereoisomer, in good yield.

### Selectivity of Cycloaddition

As shown previously in Schemes 3 and 5B, the reductive [3 + 2] cycloaddition reaction showed a high regio- and diastereocontrol, according to the dipolarophile used. *N*-Enoyl oxazolidinone and *tert*-butyl acrylate gave products (**3a**, **3e**) with new C–C bond formation occurring at the carbonyl carbon atom and the α position of the alkene as a single product, whereas methyl cinnamate gave a product (**3h**) with new C–C bond formation occurring at the carbonyl carbon atom and the β position of the alkene as a single product. These results indicate that the selectivity is highly dependent on the dipolarophile, and accordingly, we turned our attention to the use of a chiral auxiliary as it can potentially give diastereo- and enantiomerically pure pyrrolidines after the removal of the oxazolidinone group. Interestingly and unexpectedly, the cycloaddition with a chiral coupling partner gave two regioisomers of the product in a 1:1 ratio and a combined 78% yield (**8a**, **8b**, Scheme 5B). These two isomers are fully separable by silica gel chromatography, and each of them is obtained essentially as a single isomer (>20:1 dr). The absolute and relative configuration of **8a** was unambiguously determined via single X-ray diffraction analysis.

## Mechanistic Investigations

### DFT Study

To further investigate the selectivity involved in the cycloaddition reaction, we turned to DFT calculations. Our focus was on elucidating the origin of the inversion of selectivity when methyl cinnamate was used as a coupling partner as opposed to *N*-enoyl oxazolidinone, giving, respectively, **3h** and **3d**. The regio- and diastereoselectivities of the 1,3-dipolar cycloaddition involving an azomethine ylide and a dipolarophile are determined by either the strain or the interaction energies of the cycloaddition transition structures depending on the nature of the dipolarophile. The strain energy is decisive for the selectivity when the dipolarophile is methyl cinnamate, whereas the interaction energy controls for the selectivity when the dipolarophile possesses an oxazolidinone group. The origin of this unique divergent behavior depending on the structure of the dipolarophile is quantified and explained below.

**Scheme 6:**
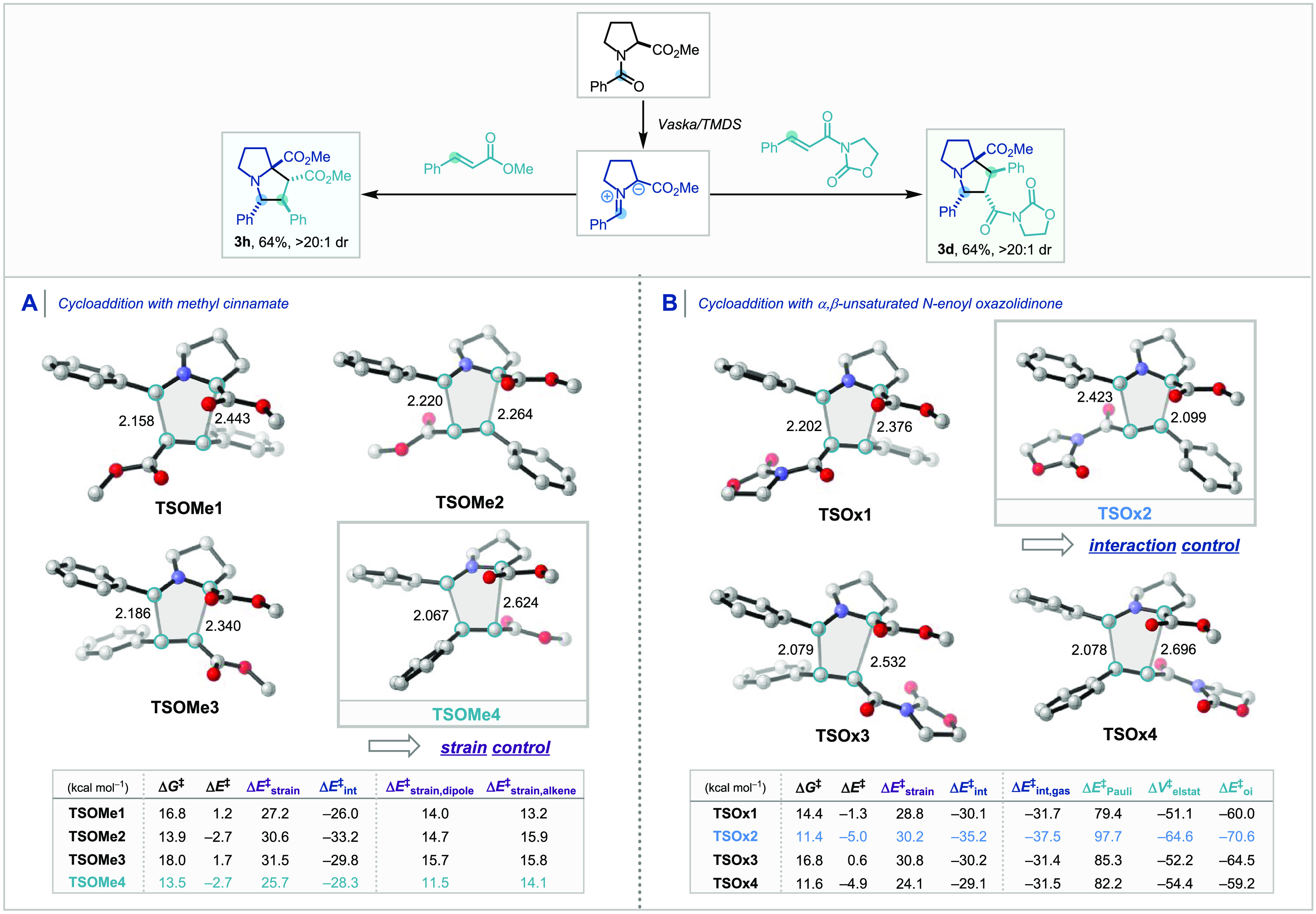
Transition Structures for the 1,3-Dipolar Cycloaddition between the Azomethine Ylide and Methyl Cinnamate (A) and *N*-Enoyl Oxazolidinone (B) Computed at COSMO(Toluene)-M06-2X/TZ2P//BP86/TZ2P Energies (kcal mol^–1^) and forming bond lengths (Å) of TS geometries are provided in the inset.

**Scheme 7:**
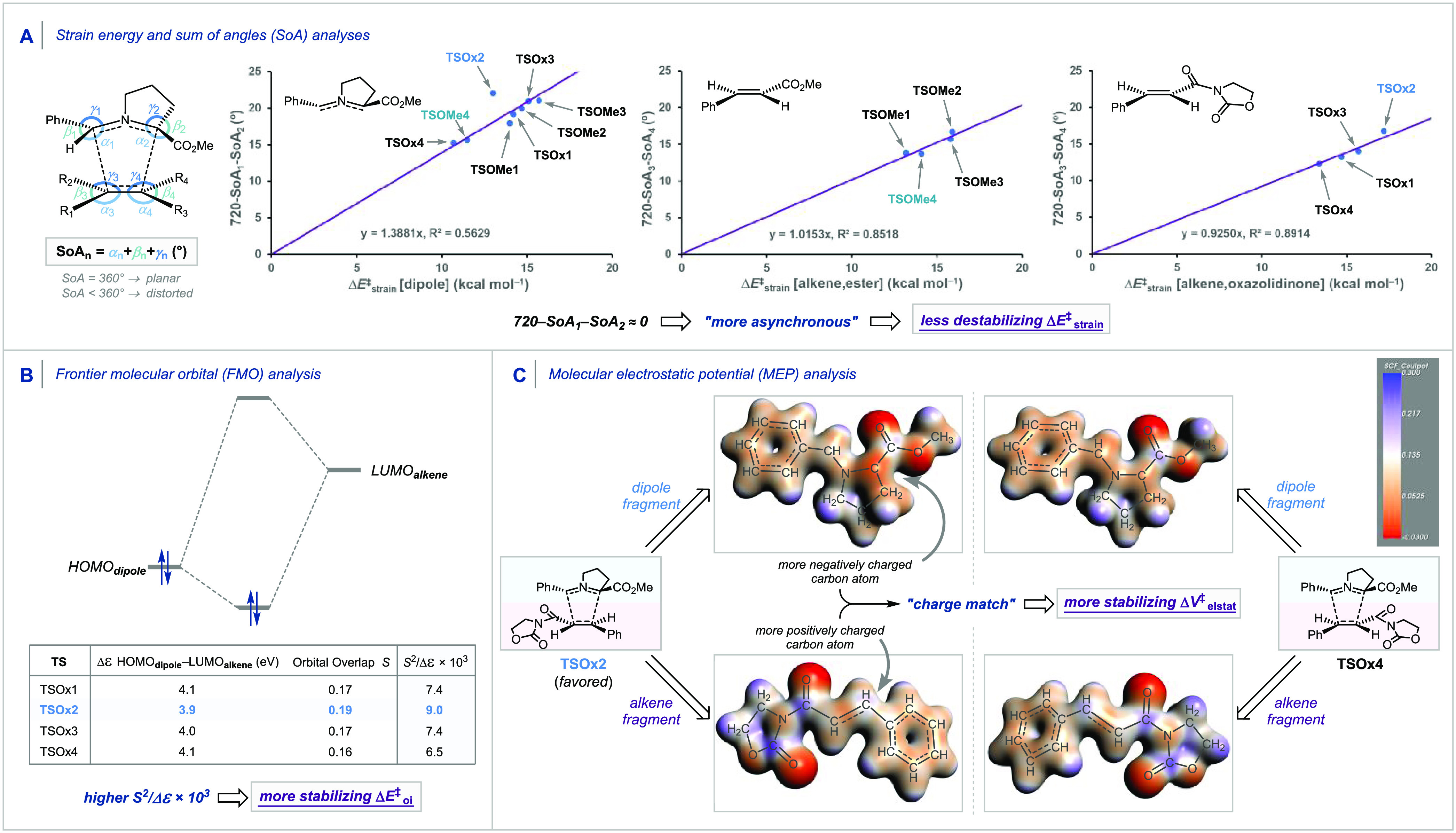
(A) Correlation between the Sum of Angles (SoA in degrees) and the Strain Energies of the Azomethine Ylide and the Dipolarophiles, (B) Frontier Molecular Orbital (FMO) Diagrams with Calculated Key Orbital Energy Gaps and Overlaps of the Normal Electron Demand (NED) HOMO_dipole_–LUMO_alkene_ Interaction, and (C) Molecular Electrostatic Potential (MEP) Map Surfaces (at 0.03 Bohr^–3^) from −0.03 (Red) to 0.03 (Blue) Hartree e^–1^ of Distorted Dipole and Dipolarophile Fragments for the 1,3-Dipolar Cycloadditions between the Azomethine Ylide and the Oxazolidinone-Substituted Alkene Computed at COSMO(Toluene)-M06-2X/TZ2P//BP86/TZ2P

The key cycloaddition transition structures between the *in situ* generated azomethine ylide^10^ and methyl cinnamate or *N*-enoyl oxazolidinone are provided in Scheme 6. Among the four TSs with methyl cinnamate as the dipolarophile, the energy barrier via **TSOMe4** is the most favorable, whereas, for *N*-enoyl oxazolidinone, **TSOx2** is the most energetically favorable transition structure.^11^ The origin of the kinetic preference for the regio- and diastereomer-determining cycloaddition steps was quantified using the activation strain model (ASM),^12a−12d,12e^ also known as the distortion-interaction model.^12f,12g^ The ASM involves the decomposition of the electronic activation barrier (Δ*E*^‡^) into two distinct energy terms, namely, the strain energy (Δ*E*_strain_^‡^) that results from the deformation of the individual reactants and the interaction energy (Δ*E*_int_^‡^) between the deformed reactants. These analyses have revealed that the strain energy controls the selectivity through **TSOMe4** with methyl cinnamate, while the interaction energy is decisive for the selectivity through **TSOx2** with *N*-enoyl oxazolidinone. The higher degree of asynchronicity, defined as the difference in the length of two newly forming C–C bonds in the transition structures, in **TSOMe4** leads to a less destabilizing strain energy. We recently reported the connection between asynchronicity and strain energy in the related Diels–Alder cycloaddition reaction.^13^ A higher degree of asynchronicity leads to one C–C bond to form before the other and results in a smaller degree of pyramidalization (sum of angles [SoA] around a carbon atom in degrees) at the reacting carbon atoms (Scheme 7A). A good linear relationship was found between the normalized sum of angles of pyramidalization at two reacting carbon atoms (720–SoA1–SoA2 [for the dipole fragment] or 720–SoA3–SoA4 [for the dipolarophile fragment]) and the strain energies Δ*E*_strain_^‡^ of each fragment. Therefore, the TS that minimizes the pyramidalization of the reacting carbon atoms (SoA closer to 360°) benefits from a less destabilizing strain energy by the asynchronicity of the TS and thus a lower activation barrier if interaction energies are all similar.

On the other hand, the interaction energy was found to be operative in controlling the selectivity when the dipolarophile contains an oxazolidinone group and actually overrules the strain energy, which was decisive with the methyl cinnamate substrate, as previously discussed. The prominent role of the interaction energy on the observed reactivity trends stimulated the analysis of various contributions to the interaction using a canonical energy decomposition analysis (EDA).^14^ Our canonical EDA decomposed the Δ*E*_int_^‡^ between the distorted reactants in the transition state into three physically meaningful energy terms: classical electrostatic interaction (Δ*V*_elstat_^‡^), steric (Pauli) repulsion (Δ*E*_Pauli_^‡^), which, in general, arises from the two-center four-electron repulsion between the closed-shell orbitals of both reactants, and stabilizing orbital interactions (Δ*E*_oi_^‡^) that account, among others, for HOMO–LUMO interactions. Analysis of EDA terms computed on the solution phase geometries in the gas phase^15^ revealed that the more stabilizing orbital interactions (Δ*E*_oi_^‡^) and electrostatic interactions (Δ*V*_elstat_^‡^) for **TSOx2** set the trend in Δ*E*_int_^‡^. Analysis of the bonding mechanism and frontier molecular orbital (FMO) interactions revealed that the origin of the more stable Δ*E*_oi_^‡^ associated with **TSOx2** originates from both a smaller normal electron demand (NED) HOMO_dipole_–LUMO_alkene_ energy gap and the larger orbital overlap *S* compared to the other TSs. These combined effects result in the most stabilizing orbital interactions (S^2^/Δε × 10^3^ = 9.0) for **TSOx2** (Scheme 7B).^16^ The more stable Δ*V*_elstat_^‡^ for **TSOx2** can be understood from analysis of the molecular electrostatic potential (MEP) maps (Scheme 7C). Here, we see that the two carbon atoms participating in the shorter newly forming C–C bond for **TSOx2** benefit from a complimentary “charge match” compared to that of **TSOx4** (the next most favorable TS). That is, the negatively charged carbon atom on the dipole and the positively charged carbon atom on the dipolarophile conveniently enter into a stabilizing electrostatic interaction, a feature that is maximized when the electron-withdrawing group on the dipole and alkene is positioned opposite of each other, such as in **TSOx2**.

## Conclusions

In summary, we have developed a new, general, and highly selective reductive [3 + 2] cycloaddition reaction of amides and conjugated alkenes for structurally complex pyrrolidine synthesis. This unified strategy enabled by the use of Vaska’s complex and TMDS to reductively generate a range of stabilized and unstabilized azomethine ylide species (including some inaccessible via previous nonreductive approaches), which afforded after cycloaddition a wide range of highly and diversely substituted pyrrolidines and polycyclic amine products. The reaction proceeds under mild conditions, enabling generally high diastereoselectivity and compatibility with a variety of electron-poor olefins. The use of single enantiomer or tethered dipolarophiles demonstrates applicability to the synthesis of complex and enantiopure cyclic amine architectures. The origin of high regio- and diastereoselectivities in the cycloaddition reaction was elucidated by means of density functional theory (DFT) computations. Use of the activation strain model (ASM) in conjunction with the matching canonical energy decomposition analysis (EDA) revealed that the selectivity is determined by either the strain or the interaction energies depending on the substituent on the dipolarophile. Highly asynchronous transition states are energetically preferred and go with a lower strain energy than the synchronous one, unless a highly stabilizing interaction energy between the reactants is present, in which the orbital and electrostatic interactions are decisive. Furthermore, the successful application of this method to the construction of a complex tricyclic core efficiently from a readily prepared substrate shows the potential to synthesize complex molecules possessing naturally abundant pyrrolidine scaffolds.

## Supporting Information Available

Experimental procedures, NMR data, characterization, crystallographic data, and computational data (PDF)
