# Supplementary material for: General Pyrrolidine Synthesis via Iridium-Catalyzed Reductive Azomethine Ylide Generation from Tertiary Amides and Lactams
Source: ACS Catal. 2021 Jun 9;11(12):7489–97. doi: 10.1021/acscatal.1c01589 (PMC8291578; doi:10.1021/acscatal.1c01589)
Supplement: Supplementary file 1 — cs1c01589_si_001.pdf [file cs1c01589_si_001.pdf]

Supporting information

For

A General Pyrrolidine Synthesis via Iridium-Catalysed Reductive Azomethine Ylide Generation from Tertiary Amides & Lactams

Ken Yamazaki,[a,b] Pablo Gabriel,[a] Graziano Di Carmine,[a] Julia Pedroni,[a] Mirxan Farizyan[a], Trevor A. Hamlin*[b] and Darren J. Dixon*[a]

[a] Department of Chemistry, Chemistry Research Laboratory, University of Oxford, 12 Mansfield Road, Oxford, OX1 3TA, UK.

[b] Department of Theoretical Chemistry, Amsterdam Institute of Molecular and Life Sciences (AIMMS), and Amsterdam Center for Multiscale Modeling (ACMM), Vrije Universiteit Amsterdam, De Boelelaan 1083, 1081 HV Amsterdam, The Netherlands

* Email: t.a.hamlin@vu.nl

* Email: darren.dixon@chem.ox.ac.uk

Contents

1. General information 7

2. General procedures 10

General Procedure A: cycloaddition of amides and lactams with a pending ester group 10

General Procedure B: cycloaddition of amides with a TMS group 11

General Procedure C: cycloaddition of amides followed by DDQ oxidation 11

General Procedure D: Synthesis of amides 12

General Procedure E: Synthesis of amides 13

General Procedure F: Synthesis of amides 14

General Procedure G: alkylation of lactams 14

3. Synthesis and characterization of starting materials 15

Commercially available and previously reported substrates and coupling partners 15

benzyl 2-(2-oxopyrrolidin-1-yl)acetate (1m) 16

4-methoxy-N-methyl-N-((trimethylsilyl)methyl)benzamide (4c) 17

N-methyl-3,5-bis(trifluoromethyl)-*N*-((trimethylsilyl)methyl)benzamide (4d) 18

N-benzyl-3,4,5-trifluoro-*N*-((trimethylsilyl)methyl)benzamide (4e) 19

N,3-dimethyl-*N*-((trimethylsilyl)methyl)benzamide (4f) 20

5-bromo-*N*,2-dimethyl-*N*-((trimethylsilyl)methyl)benzamide (4g) 21

N-benzyl-4-chloro-*N*-((trimethylsilyl)methyl)benzamide (4h) 22

N-benzyl-4-nitro-*N*-((trimethylsilyl)methyl)benzamide (4i) 23

N-benzyl-3-nitro-*N*-((trimethylsilyl)methyl)benzamide (4j) 24

N-benzyl-4-cyano-*N*-((trimethylsilyl)methyl)benzamide (4k) 25

N-methyl-*N*-((trimethylsilyl)methyl)thiophene-2-carboxamide (4l) 26

N-benzyl-*N*-((trimethylsilyl)methyl)furan-2-carboxamide (4m) 27

N-benzyl-*N*-((trimethylsilyl)methyl)isobutyramide (4n) 28

4. Synthesis and characterization of pyrrolidines 29

methyl (2S,3R,7aR)-2-(2-oxooxazolidine-3-carbonyl)-3-phenyltetrahydro-1H-pyrrolizine-7a(5H)-carboxylate (3a) 29

methyl (2S,3S,7aR)-2-methyl-2-(2-oxooxazolidine-3-carbonyl)-3-phenyltetrahydro-1H-pyrrolizine-7a(5H)-carboxylate (3b) 30

methyl (1S,2S,3R,7aR)-1-methyl-2-(2-oxooxazolidine-3-carbonyl)-3-phenyltetrahydro-1H-pyrrolizine-7a(5H)-carboxylate (3c) 31

methyl (1R,2S,3R,7aR)-2-(2-oxooxazolidine-3-carbonyl)-1,3-diphenyltetrahydro-1H-pyrrolizine-7a(5H)-carboxylate (3d) 32

2-(tert-butyl) 7a-methyl (2S,3R,7aR)-3-phenyltetrahydro-1H-pyrrolizine-2,7a(5H)-dicarboxylate (3e) 33

methyl (3aS,4R,8aR,8bR)-1-oxo-4-phenylhexahydro-3H-furo[3,4-a]pyrrolizine-8a(6H)-carboxylate (3f) 34

methyl (1S,2S,3S,7aR)-2-nitro-1,3-diphenyltetrahydro-1H-pyrrolizine-7a(5H)-carboxylate (3g1) and methyl (1R,2S,3R,7aR)-1-nitro-2,3-diphenyltetrahydro-1H-pyrrolizine-7a(5H)-carboxylate (3g2) 35

dimethyl (1R,2S,3R,7aR)-2,3-diphenyltetrahydro-1H-pyrrolizine-1,7a(5H)-dicarboxylate (3h) 37

methyl (2S,3S,7aR)-3-phenyl-2-(phenylsulfonyl)tetrahydro-1H-pyrrolizine-7a(5H)-carboxylate (3i1) and methyl (1R,3R,7aS)-3-phenyl-1-(phenylsulfonyl)tetrahydro-1H-pyrrolizine-7a(5H)-carboxylate (3i2) 38

ethyl (2S,3R,7aR)-2-(2-oxooxazolidine-3-carbonyl)-3-phenyltetrahydro-1H-pyrrolizine-7a(5H)-carboxylate (3j) 39

methyl (2R,3S,5S)-3-(2-oxooxazolidine-3-carbonyl)-2-phenyl-1-azabicyclo[3.2.0]heptane-5-carboxylate (3k) 40

methyl (1S,3R,7aS)-1-(2-oxooxazolidine-3-carbonyl)hexahydro-1H-pyrrolizine-3-carboxylate (3l1) and methyl (2S,3R,7aS)-2-(2-oxooxazolidine-3-carbonyl)hexahydro-1H-pyrrolizine-3-carboxylate (3l2) .. 41

benzyl (1S,3R,7aS)-1-(2-oxooxazolidine-3-carbonyl)hexahydro-1H-pyrrolizine-3-carboxylate (3m1) and benzyl (2S,3R,7aS)-2-(2-oxooxazolidine-3-carbonyl)hexahydro-1H-pyrrolizine-3-carboxylate (3m2) 43

dimethyl (1S,2R,3R,7aS)-2-phenylhexahydro-1H-pyrrolizine-1,3-dicarboxylate (3n1) and dimethyl (1R,2S,3R,7aS)-1-phenylhexahydro-1H-pyrrolizine-2,3-dicarboxylate (3n2) 45

methyl (2S,4R,5R)-1-methyl-4-(2-oxooxazolidine-3-carbonyl)-5-((E)-styryl)pyrrolidine-2-carboxylate (3o) 46

3-(1-methyl-2-phenylpyrrolidine-3-carbonyl)oxazolidin-2-one (5a) 47

3-(1-benzyl-2-phenylpyrrolidine-3-carbonyl)oxazolidin-2-one (5b) 48

3-(2-(4-methoxyphenyl)-1-methylpyrrolidine-3-carbonyl)oxazolidin-2-one (5c) 49

3-(2-(3,5-bis(trifluoromethyl)phenyl)-1-methylpyrrolidine-3-carbonyl)oxazolidin-2-one (5d) 50

3-(1-benzyl-2-(3,4,5-trifluorophenyl)pyrrolidine-3-carbonyl)oxazolidin-2-one (5e) 51

3-(1-methyl-2-(m-tolyl)pyrrolidine-3-carbonyl)oxazolidin-2-one (5f) 52

3-(2-(5-bromo-2-methylphenyl)-1-methylpyrrolidine-3-carbonyl)oxazolidin-2-one (5g) 53

3-(1-benzyl-2-(4-chlorophenyl)pyrrolidine-3-carbonyl)oxazolidin-2-one (5h) 54

3-(1-benzyl-2-(4-nitrophenyl)pyrrolidine-3-carbonyl)oxazolidin-2-one (5i) 55

3-(1-benzyl-2-(3-nitrophenyl)pyrrolidine-3-carbonyl)oxazolidin-2-one (5j) 56

4-(1-benzyl-3-(2-oxooxazolidine-3-carbonyl)pyrrolidin-2-yl)benzonitrile (5k) 57

3-(1-methyl-2-(thiophen-2-yl)pyrrolidine-3-carbonyl)oxazolidin-2-one (5l)....58

3-(1-benzyl-2-(furan-2-yl)pyrrolidine-3-carbonyl)oxazolidin-2-one (5m) 59

tert-butyl 1-methyl-2-phenylpyrrolidine-3-carboxylate (5n) 60

dimethyl 1-methyl-2-phenylpyrrolidine-3,4-dicarboxylate (5o) 61

5-methyl-2,4-diphenyltetrahydropyrrolo[3,4-c]pyrrole-1,3(2H,3aH)-dione (5p)....62

1-methyl-2-phenyl-3-(phenylsulfonyl)pyrrolidine (5q) 63

dimethyl 1-benzyl-2-phenyl-1H-pyrrole-3,4-dicarboxylate (5r) 64

5. Synthesis and characterization of compounds related to the intramolecular and asymmetric exemples 65

methyl 2-(3-(but-3-en-1-yl)-2-oxopyrrolidin-1-yl)acetate (S1) 65

tert-butyl (E)-5-(1-(2-methoxy-2-oxoethyl)-2-oxopyrrolidin-3-yl)pent-2-enoate (6)....66

1-(*tert*-butyl) 2-methyl (1S,2S,31R,5aS,7aR)-octahydro-2H-cyclopenta[gh]pyrrolizine-1,2-dicarboxylate (7) 67

methyl (2R,3S,7aS)-2-((S)-4-isopropyl-5,5-dimethyl-2-oxooxazolidine-3-carbonyl)-3-phenyltetrahydro-1H-pyrrolizine-7a(5H)-carboxylate (8a) and methyl (1S,3S,7aS)-1-((S)-4-isopropyl-5,5-dimethyl-2-oxooxazolidine-3-carbonyl)-3-phenyltetrahydro-1H-pyrrolizine-7a(5H)-carboxylate (8b)....68

6. NMR data 70

NMR spectra for 1m....70

NMR spectra for 4c. 71

NMR spectra for 4d....72

NMR spectra for 4e. 74

NMR spectra for 4f....76

NMR spectra for 4g. 77

NMR spectra for 4h....78

NMR spectra for 4i. 79

NMR spectra for 4j....80

NMR spectra for 4k. 81

NMR spectra for 4l. 82

NMR spectra for 4m....83

NMR spectra for 4n. 84
NMR spectra for 3a. 85
NMR spectra for 3b. 86
NMR spectra for 3c. 87
NMR spectra for 3d. 88
NMR spectra for 3e. 89
NMR spectra for 3h. 91
NMR spectra for 3i1 and 3i2. 92
NMR spectra for 3j. 93
NMR spectra for 3k. 94
NMR spectra for 3l1. 95
NMR spectra for 3l2. 96
NMR spectra for 3m1 and 3m2. 97
NMR spectra for 3n1 and 3n2. 98
NMR spectra for 3o. 99
NMR spectra for 5a. 100
NMR spectra for 5b. 101
NMR spectra for 5c. 102
NMR spectra for 5d. 103
NMR spectra for 5e. 105
NMR spectra for 5f. 107
NMR spectra for 5g. 108
NMR spectra for 5h. 109
NMR spectra for 5i. 110
NMR spectra for 5j. 111
NMR spectra for 5k. 112
NMR spectra for 5l. 113
NMR spectra for 5m. 114
NMR spectra for 5n. 115
NMR spectra for 5o. 116
NMR spectra for 5p. 117

NMR spectra for 5q. 118

NMR spectra for 5r. 119

NMR spectra for S1. 120

NMR spectra for 6. 121

NMR spectra for 7. 122

NMR spectra for 8a. 123

NMR spectra for 8b. 124

7. Crystallographic Data 125

Data for 3h. 125

Data for 5i. 127

Data for 8a. 129

8. Computational details 131

Computational methods. 131

Cartesian coordinates and energies 132

9. References 147

1. General information

General Techniques: Reactions were carried out under a nitrogen atmosphere unless stated otherwise. Glassware was oven-dried and cooled under vacuum then purged with nitrogen before use. Room temperature refers to 22 ± 2 °C. Inert atmosphere techniques, such as Schlenk technique, were used for the handling of air/moisture sensitive reagents. Reactions carried out at 0 °C were cooled using an ice bath, those at –10/–15 °C were cooled using a mixture of ice and NaCl, and those at –78 °C were carried out using a solid CO_2/acetone bath. Reactions carried out at high temperatures were heated using an oil bath or, preferably, using DrySyn® heating blocks. Reaction temperatures refer to external temperatures, for example of a heating block or oil bath, not of internal reaction temperatures unless stated otherwise.

Nomenclature and Numbering: Compounds are named following IUPAC nomenclature as generated by ACD LABS or ChemDraw. Carbon atoms are numbered in a systematic way where possible to ease comparison between related compounds in a synthetic sequence, not necessarily in accordance with IUPAC conventions, with the exception of natural products (conventional numbering following biosynthesis).

Solvents and Reagents: Ether refers to diethyl ether; petrol refers to the fraction of petroleum ether that boils between 30 and 40 °C. Where solvent dryness was important, CH_2Cl_2, Et_2O, methanol, THF, and toluene were either obtained from dry solvent bottles with septa (Aldrich), an MBRAUN-SPS solvent purification system in which solvent is passed through an activated alumina column under nitrogen, or by standing over 3 Å molecular sieves under an atmosphere of nitrogen. Reagents were used as obtained without further purification unless stated otherwise. Chromatography: Thin layer chromatography (TLC) was carried out using Merck aluminium backed DC60 F254 plates (particle size 0.2 mm). TLC sheets were visualised by UV light, then developed by staining with potassium permanganate, anisaldehyde, cerium ammonium molybdate, vanillin, or iodine on silica. Purification by flash column chromatography[1] was carried out using Merck silicagel 60 F254 (particle size 43–60 μm).

Characterisation: Proton (^{1}H) and carbon (^{13}C) spectra were recorded on Bruker AVG400 (400/101 MHz), Bruker AVH400 (400/101 MHz), Bruker AVF400 (400/101 MHz), Bruker AVB500 (500/126 MHz),

Bruker AVC500 (500/126 MHz), and Bruker DPX200 (200 MHz) NMR spectrometers. Spectra are referenced to the residual solvent peak ($CHCl_3$: δ_H 7.26, δ_C 77.16; C_7D_7H: δ_H 2.08, δ_C 20.43; $(CD_3)_2CO$: δ_H 2.05, δ_C 206.26; C_6D_5H: δ_H 7.16; CD_2HOD: 3.31, δ_C 49.00). Chemical shifts (δ) are given in parts per million (ppm, ± 0.01) and coupling constants (*J*) are given in Hertz (Hz, ± 0.1 as measured on Mestrenova, without rounding). The following convention is used to report chemical shifts: δ (multiplicity, number of protons, coupling constant(s), assignment), with chemical shifts reported in descending order. When a large roof effect was observed, it was included after the multiplicity. Peak assignments were made based on chemical shifts, integrations, coupling constants, and two-dimensional techniques such as COSY and HSQC. Where necessary, additional experiments such as HMBC, nOe, and NOESY were also used. Peak multiplicities are described as singlet (s), doublet (d), triplet (t), quartet (q), pentet (p), heptet (h), nonuplet (n), a combination e.g. doublet of doublets (dd), or as a multiplet (m) over a peak range. Additionally, peaks may be described as broad (br), or apparent (app)

Infrared spectra were recorded using a Bruker Tensor 27 FT-IR spectrometer. Selected diagnostic absorption maxima (νmax) are reported in wavenumbers (cm^{-1}). Low-resolution mass spectra were acquired using a Micromass LCT Premier spectrometer (ESI). High resolution mass spectra were recorded by Chemistry Research Laboratory staff using a Bruker Daltronics MicroTOF spectrometer (ESI). Mass to charge ratios (m/z) are reported in Daltons. Melting points were recorded using a Leica Galen III hot-stage microscope apparatus and are reported uncorrected in degrees Celcius (°C). Optical rotations were recorded using a Perkin Elmer 241 optical activity polarimeter at 25 °C. No specific rotations were measured in this work, due to the reactions developed being racemic.

X-Ray Crystallography: Single crystal X-ray diffraction data were collected using an Enraf–Nonius KCCD diffractometer or an Oxford Diffraction SuperNova diffractometer at 150 K. Crystal structures were solved with SIR92 and refined with CRYSTALS by Dr Heyao Shi (University of Oxford) with assistance and guidance from Dr Amber L. Thompson and Dr Kirsten E. Christensen (University of Oxford Chemical Crystallography department).

Synthesis of Known Compounds: The synthesis of compounds previously reported in the literature are labelled as such. Two relevant pieces of characterisation are given, unless the compound is used without purification in the next step. References are given for experimental procedures and data.

Assignment of stereochemistry: Relative stereochemistry was assigned using 2D NOESY NMR experiments, or single crystal X-ray analysis when possible.

2. General procedures

General Procedure A: cycloaddition of amides and lactams with a pending ester group

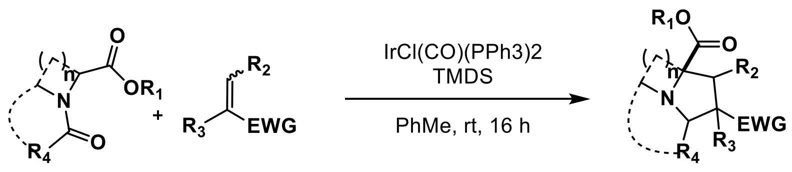

The amide (0.25 mmol), the coupling partner (0.28 mmol) and Vaska's complex (2.0 mg, 1 mol%) were loaded in a dried Schlenk tube under inert conditions. Toluene (1 mL) was introduced under a positive flow of nitrogen, followed by TMDS (88 µL, 0.50 mmol). The Schlenk tube was connected to a nitrogen line equipped with an oil bubbler, allowing gas to escape the reaction vessel. Upon addition of TMDS, bubbling should be observed within 10–15 s. The resulting solution was stirred for 16 h.

Note: For oily or liquid amides/lactams, Vaska's complex was introduced after the toluene. Sometimes poor conversion was observed when the iridium catalyst was added in neat substrate.

The reaction mixture was then loaded on a small Silica gel column (bubbling can occur), the reaction vessel rinsed with 8 : 1 EtOAc/MeOH, and this was also loaded on the silica gel column. Elution was performed with the above solvent (3 × 5 mL). The resulting solution was then concentrated *in vacuo*. Purification *via* FCC afforded the corresponding product.

General Procedure B: cycloaddition of amides with a TMS group

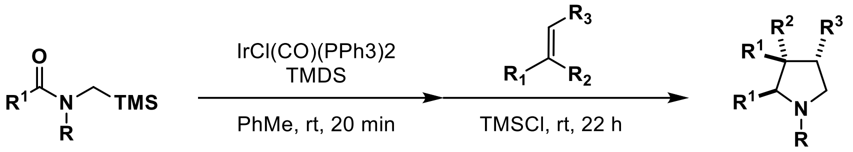

To an oven-dried 5 ml round bottom flask was added an amide (0.25 mmol, 1.0 eq.), $IrCl(CO)(PPh_3)_2$ (2.0 mg, 0.0025 mmol, 0.01 eq.) and toluene (1.0 ml), followed by the addition of TMDS (44 µL, 0.25 mmol, 1.0 eq.). The mixture was stirred under nitrogen atmosphere at room temperature. After 20 min, dipolarophile (0.50 mmol, 2.0 eq.) and TMSCl (6.3 µL, 0.050 mmol, 0.2 eq.) were added and stirred at room temperature for 22 h. DCM was added and the mixture was washed with sat. aq. Na_2CO_3 and brine. After drying over Na_2SO_4 and concentration under reduced pressure, the remaining crude was purified by flush column chlomatography on silica gel to give the product.

General Procedure C: cycloaddition of amides followed by DDQ oxidation

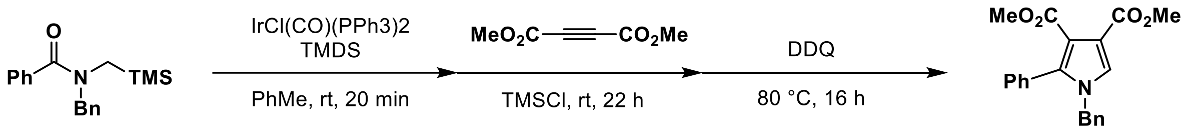

To an oven-dried 5 ml round bottom flask was added an amide (0.25 mmol, 1.0 eq.), $IrCl(CO)(PPh_3)_2$ (2.0 mg, 0.0025 mmol, 0.01 eq.) and toluene (1.0 ml), followed by the addition of TMDS (44 µL, 0.25 mmol, 1.0 eq.). The mixture was stirred under nitrogen atmosphere at room temperature. After 20 min, dimethyl acetylenedicarboxylate (0.50 mmol, 2.0 eq.) and TMSCl (6.3 µL, 0.050 mmol, 0.2 eq.) were added and stirred at room temperature for 22 h. DDQ (0.50 mmol, 2.0 eq.) was added and the reaction mixture was stirred at 80 °C for further 16 h. DCM was added and the mixture was washed with sat. aq. Na_2CO_3 and brine. After

drying over Na_2SO_4 and concentration under reduced pressure, the remaining crude was purified by flush column chlomatography on silica gel to give the product.

General Procedure D: Synthesis of amides

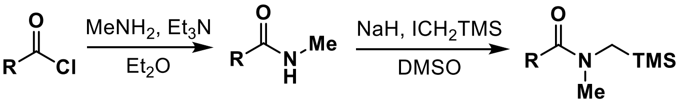

In a solution of triethylamine (8.3 mL, 60 mmol, 2.0 equiv.) in Et_2O (60 mL) under nitrogen atmosphere, acid chloride (30 mmol, 1.0 equiv.) was added dropwise at 0 °C. The mixture was added methylamine solution 33 wt. % in EtOH (5.5 mL, 45 mmol, 1.5 equiv.) dropwise. The resulting mixture was allowed to warm to room temperature and stirred 1 h. Et_2O was added and the mixture was washed with 1M HCl, sat. aq. $NaHCO_3$ and brine. After drying over Na_2SO_4 and concentration under reduced pressure, the remaining crude was purified by flush column chlomatography on silica gel to give *N*-methylamide. The amide (10 mmol, 1.0 equiv.) was added to a suspension of NaH (60 % w/w in mineral oil, 440 mg, 11 mmol, 1.1 equiv.) in DMSO (30 mL) at room temperature under nitrogen atmosphere. After 1.5 h, ICH_2TMS (1.5 mL, 10 mmol, 1.0 equiv.) was added and stirred at room temperature for 16 h. The reaction was then poured into water and extracted with Et_2O. The combined organic phase was dried over Na_2SO_4, filtered, and concentrated under reduced pressure. The crude was purified by flush column chlomatography on silica gel.

General Procedure E: Synthesis of amides

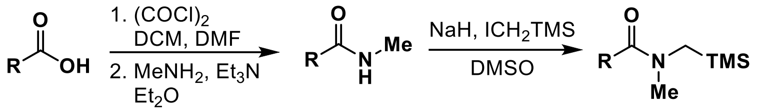

To a stirred solution of a carboxylic acid (30 mmol, 1.0 equiv.) and DMF (10 drops) in DCM (20 mL), $(COCl)_2$ (3.0 mL, 36 mmol, 1.2 equiv.) was added dropwise. The solution was stirred at room temperature for 2 h. The solvent was then removed by evaporation under reduced pressure, and the resulting residue was dissolved in DCM (30 mL). After cooling the reaction mixture to 0 °C, the mixture was added methylamine solution 33 wt. % in EtOH (5.5 mL, 45 mmol, 1.5 equiv.) dropwise. The resulting mixture was allowed to warm to room temperature and stirred 1 h. DCM was added and the mixture was washed with 1M HCl, sat. aq. $NaHCO_3$ and brine. After drying over Na_2SO_4 and concentration under reduced pressure, the remaining crude was purified by flush column chlomatography on silica gel to give *N*-methylamide. The amide (10 mmol, 1.0 equiv.) was added to a suspension of NaH (60 % w/w in mineral oil, 440 mg, 11 mmol, 1.1 equiv.) in DMSO (30 mL) at room temperature under nitrogen atmosphere. After 1.5 h, ICH_2TMS (1.5 mL, 10 mmol, 1.0 equiv.) was added and stirred at room temperature for 16 h. The reaction was then poured into water and extracted with Et_2O. The combined organic phase was dried over Na_2SO_4, filtered, and concentrated under reduced pressure. The crude was purified by flush column chlomatography on silica gel.

General Procedure F: Synthesis of amides

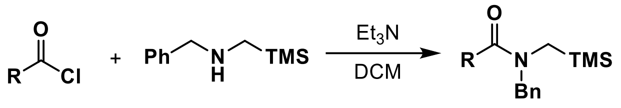

In a solution of triethylamine (0.14 mL, 2.0 mmol, 2.0 equiv.) in DCM (1.0 mL) under nitrogen atmosphere, acid chloride (1.0 mmol, 1.0 equiv.) was added dropwise at 0 °C. The mixture was added *N*-[(Trimethylsilyl)methyl]benzylamine (0.19 mL, 1.0 mmol, 1.0 equiv.) dropwise. The resulting mixture was allowed to warm to room temperature and stirred 1 h. DCM was added and the mixture was washed with 1M HCl, sat. aq. $NaHCO_3$ and brine. After drying over Na_2SO_4 and concentration under reduced pressure, the remaining crude was purified by flush column chlomatography on silica gel to give the product.

General Procedure G: alkylation of lactams

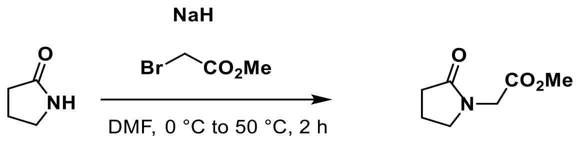

The lactam was dissolved in anhydrous DMF (4 mL/mmol substrate). NaH was added carefully portionwise (2.00 equiv.), and the resulting suspension was placed in a pre-heated oil bath at 50 °C for 30 min (to help dissolution of the sodium salt). The reaction mixture was then cooled down to 0 °C, and methyl bromoacetate was added dropwise. The reaction flask was placed back in the oil bath, and left to stir at 50 °C for 2 h. It was cooled down to rt, and quenched with saturated NH_4Cl (1mL/mmol substrate). Extraction with EtOAc (3 × 10 mL/mmol substrate) was then carried out. The combined organics washed with brine, dried over Na_2SO_4 and concentrated *in vacuo*. Purification *via* FCC afforded the corresponding product.

3. Synthesis and characterization of starting materials

Commercially available and previously reported substrates and coupling partners

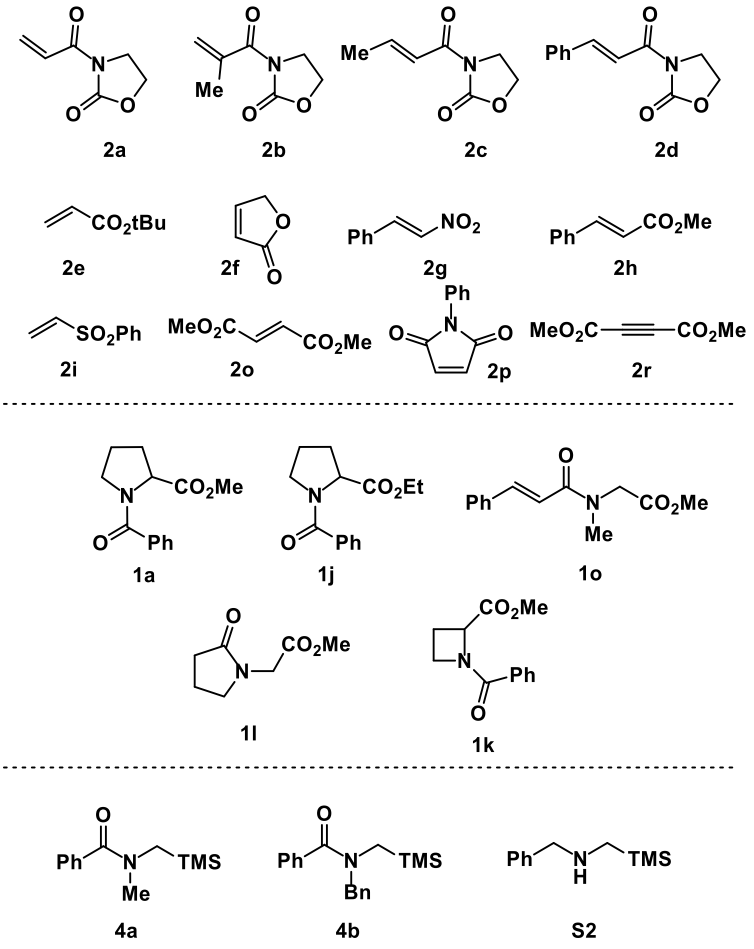

The coupling partners or amide starting materials above were either commercial (1a, 1l, 2e, 2f, 2g, 2h, 2o, 2p, 2r, 4a, 4b) or made according to reported literature procedures (1o,[1] 1j,[2] 1k,[3] 2a-2d,[4] 4a-4b,[5] S2 [6]).

benzyl 2-(2-oxopyrrolidin-1-yl)acetate (1m)

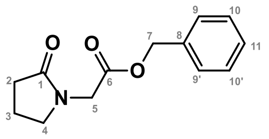

Prepared according to **General Procedure G** from pyrrolidinone and benzyl bromoacetate. Purification *via* FCC (1 : 1 pentane/EtOAc) gave **1m** as a pale yellow oil (48 mg, 54%).

IR 2926, 1745 (C^6=O), 1682 (C^1=O), 1496, 1461, 1440, 1425, 1289, 1176.

^{1}H NMR ($CDCl_3$, 400 MHz) δ_H: 7.29–7.38 (m, 5H, C^9H, $C^{9'}$H, C^{10}H, $C^{10'}$H, C^{11}H), 5.15 (s, 2H, C^7H_2), 4.09 (s, 2H, C^5H_2), 3.42–3.49 (m, 2H, C^4H_2), 2.37–2.43 (m, 2H, C^2H_2), 2.00–2.10 (m, 2H, C^3H_2).

^{13}C NMR ($CDCl_3$, 101 MHz) $\delta_{C:}$ 175.7 (C^1), 168.6 (C^6), 135.3 (C^8), 128.7 (C^9), 128.6 (C^{11}), 128.4 (C^{10}), 67.1 (C^7), 47.7 (C^4), 44.2 (C^5), 30.3 (C^2), 18.0 (C^3).

HRMS (ES^+) exact mass calculated for $[M+H]^+$ ($C_{13}H_{15}O_3NNa^+$) requires **m/z** 256.0944, found **m/z** 256.0944.

4-methoxy-N-methyl-N-((trimethylsilyl)methyl)benzamide (4c)

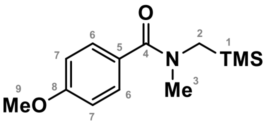

Prepared according to **General Procedure D**. Purification *via* FCC (4 : 1 pentane/EtOAc) gave **4c** as a pale yellow solid (2.1 g, 83%).

m.p. 28-30 °C.

IR 2953, 1608 (C^4=O), 1482, 1394, 1246, 1172, 1029.

^{1}H NMR ($CDCl_3$, 400 MHz) δ_H: 7.38–7.30 (m, 2H, C^6H), 6.92-6.85 (m, 2H, C^7H), 3.82 (s, 3H, C^9H), 3.06 (s, 2H, C^2H_2), 3.00 (s, 3H, C^3H_3), 0.13 (s, 9H, C^1H_3).

^{13}C NMR ($CDCl_3$, 101 MHz) δ_C: 165.6 (C^4), 160.3 (C^9), 129.0 (C^5), 128.8 (C^6), 113.6 (C^7), 55.3 (C^9), 40.5 (C^2), 40.2 (C^3), −1.4 (C^1).

HRMS (ES^+) exact mass calculated for $[M+H]^+$ ($C_{13}H_{22}NO_2Si^+$) requires **m/z** 252.1414, found **m/z** 252.1413.

N-methyl-3,5-bis(trifluoromethyl)-N-((trimethylsilyl)methyl)benzamide (4d)

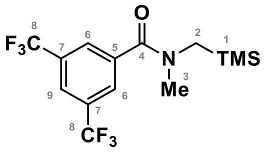

Prepared according to **General Procedure D**. Purification *via* FCC (4 : 1 pentane/EtOAc) gave **4d** as a pale yellow oil (2.4 g, 68%).

IR 1636 (C^4=O), 1366, 1277, 1129, 864.

^{1}H NMR ($CDCl_3$, 400 MHz) δ_H: 168.41 (C^4), 140.23 (C^5), 133.39 (q, J = 33.5 Hz, C^7), 128.64 (C^6), 125.05 (C^8), 122.96 (C^9), 41.95 (C^2), 41.44 (C^3), 0.00 (C^1).

^{13}C NMR ($CDCl_3$, 101 MHz) δ_C: 168.4 (C^4), 140.2 (C^5), 133.4 (q, J = 33.5 Hz, C^7), 128.6 (C^6), 125.1 (C^8), 123.0 (C^9), 42.0 (C^2), 41.4 (C^3), 0.0 (C^1).

^{19}F NMR ($CDCl_3$, 377 MHz) δ_H: −63.0.

HRMS (ES^+) exact mass calculated for $[M+H]^+$ ($C_{14}H_{18}NOSi^+$) requires **m/z** 358.1056, found **m/z** 358.1045.

***N*-benzyl-3,4,5-trifluoro-*N*-((trimethylsilyl)methyl)benzamide (4e)**

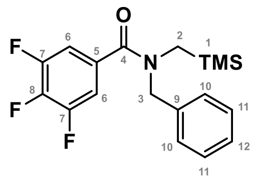

Prepared according to **General Procedure F**. Purification *via* FCC (4 : 1 pentane/EtOAc) gave **4e** as a pale yellow oil (0.34 mg, 98%), and as a 88(**A**) : 12(**B**) mixture of rotamers.

IR 1626 (C^4=O), 1451, 1247, 851

^{1}H NMR ($CDCl_3$, 400 MHz) δ_H: 7.40–7.34 (m, 2H, ArH), 7.33–7.28 (m, 1H, ArH), 7.12 (d, J = 7.4 Hz, 2H, ArH), 7.08–7.01 (t, J = 6.8 Hz, 2H, ArH), 4.74 (s, 2H, C^3H_2, (**B**)), 4.48 (s, 2H, C^3H_2, (**A**)), 2.97 (s, 2H, C^2H_2, (**A**)), 2.82 (s, 2H, C^2H_2, (**B**)), 0.13 (s, 9H, C^1H_3).

^{13}C NMR ($CDCl_3$, 101 MHz) δ_C: 169.0 (C^4), 152.3 (ddd, J = 253.0, 10.2, 3.7 Hz, C^7), 141.7 (dt, J = 255.1, 15.3 Hz, C^8), 137.1 (C^9), 133.7 – 133.3 (m, C^5), 130.3 (C^{11}), 129.2 (C^{12}), 127.8 (C^{10}), 113.55 – 112.0 (m, C^6), 56.3 (C^3), 39.3 (C^2), 0.0 (C^1).

^{19}F NMR ($CDCl_3$, 377 MHz) δ_H: −132.5 (dd, J = 20.4, 7.3 Hz), −158.0 – −158.4 (m).

HRMS (ES$^+$) exact mass calculated for $[M+H]^+$ ($C_{18}H_{21}F_3NOSi^+$) requires **m/z** 352.1345, found **m/z** 352.1345.

***N*,3-dimethyl-*N*-((trimethylsilyl)methyl)benzamide (4f)**

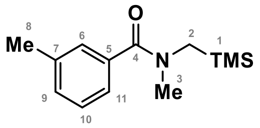

Prepared according to **General Procedure D**. Purification *via* FCC (4 : 1 pentane/EtOAc) gave **4f** as a pale yellow oil (0.93 g, 39%), and as a 80(**A**) : 20(**B**) mixture of rotamers.

IR 1624 (C^4=O), 1393, 1247, 1074, 842

^{1}H NMR ($CDCl_3$, 400 MHz) δ_H: 7.30–7.10 (m, 4H, ArH), 3.09 (s, 2H, C^2H_2), 2.95 (s, 3H, C^3H_3 (**A**)), 2.89 (s, 3H, C^3H_3, (**B**)), 2.36 (s, 3H, C^8H_3), 0.16 (s, 9H, C^1H_3 (**A**)), 0.05 (s, 9H, C^1H_3 (**B**)).

^{13}C NMR ($CDCl_3$, 101 MHz) δ_C: 171.9 (C^4), 139.5 (C^7), 138.2 (C^5), 131.2 (C^9), 129.5 (C^{10}), 128.8 (C^6), 125.2 (C^{11}), 41.6 (C^3 or C^2), 41.3 (C^2 or C^3), 0.0 (C^1).

HRMS (ES^+) exact mass calculated for $[M+H]^+$ ($C_{13}H_{22}NOSi^+$) requires **m/z** 236.1465, found **m/z** 236.1465.

5-bromo-*N*,2-dimethyl-*N*-((trimethylsilyl)methyl)benzamide (4g)

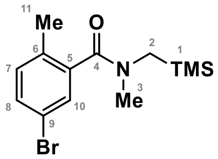

Prepared according to **General Procedure E**. Purification *via* FCC (4 : 1 pentane/EtOAc) gave **4g** as a pale yellow oil (2.1 g, 67%), and as a 87(**A**) : 13(**B**) mixture of rotamers.

IR 2922, 1621 (C^4=O), 1462, 1396, 1246, 840, 814

^{1}H NMR ($CDCl_3$, 400 MHz) δ_H: 7.36 (dd, *J* = 8.1, 2.0 Hz, 1H, C^8H), 7.29–7.21 (m, 1H, C^7H), 7.07 (d, *J* = 8.1 Hz, 1H, $C^{10}H$), 3.10 (s, 2H, C^2H_2), 2.82 (s, 3H, C^3H_3 (**A**)), 2.71 (s, 3H, C^3H_3 (**B**)), 2.23 (s, 3H, $C^{11}H_3$), 0.17 (s, 9H, C^1H_3 (**A**)), 0.05 (s, 9H, C^1H_3 (**B**)).

^{13}C NMR ($CDCl_3$, 101 MHz) δ_C: 169.7 (C^4), 140.3 (C^5), 134.3 (ArC), 133.3 (ArC), 132.8 (ArC), 130.4 (ArC), 130.3 (ArC), 130.0 (ArC), 129.5 (ArC), 126.6 (ArC), 120.7 (ArC), 43.7 (C^3, (**B**)), 40.7 (C^3, (**A**)), 40.2 (C^2, (**A**)), 36.0 (C^2, (**B**)), 19.9 (C^{11}), 0.0 (C^1, (**A**)), –0.3 (C^1, (**B**)).

HRMS (ES^+) exact mass calculated for $[M+H]^+$ ($C_{13}H_{21}BrNOSi^+$) requires **m/z** 314.0570, 316.0550, found **m/z** 314.0569, 316.0547.

N-benzyl-4-chloro-N-((trimethylsilyl)methyl)benzamide (4h)

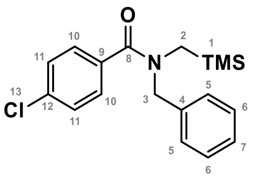

Prepared according to **General Procedure F**. Purification *via* FCC (4 : 1 pentane/EtOAc) gave **4h** as a white solid (0.23 g, 97%), and as a 88(**A**) : 12(**B**) mixture of rotamers.

IR 1627 (C^8=O), 1452, 1436, 1397, 1247, 1089

^{1}H NMR ($CDCl_3$, 400 MHz) δ_H: 7.42–7.25 (m, 7H, ArH), 7.13 (d, J = 7.3 Hz, 2H, ArH), 4.77 (s, 2H, C^3H_2, (**B**)), 4.48 (s, 2H, C^3H_2, (**A**)), 2.96 (s, 2H, C^2H_2, (**A**)), 2.86 (s, 2H, C^2H_2, (**B**)), 0.13 (s, 9H, C^1H_3, (**A**)), 0.02 (s, 9H, C^1H_3, (**B**)).

^{13}C NMR ($CDCl_3$, 101 MHz) δ_C: 171.2 (C^8), 137.6 (ArC), 136.5 (ArC), 136.1 (ArC), 130.0. (ArC), 129.8 (ArC), 129.3 (ArC), 128.8 (ArC), 127.8 (ArC), 56.2 (C^3), 38.8 (C^2), 0.0 (C^1).

HRMS (ES^+) exact mass calculated for $[M+H]^+$ ($C_{18}H_{23}ClNOSi^+$) requires **m/z** 332.1232, found **m/z** 332.1233.

N-benzyl-4-nitro-N-((trimethylsilyl)methyl)benzamide (4i)

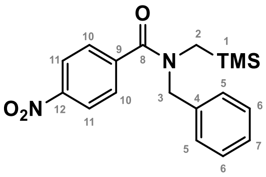

Prepared according to **General Procedure F**. Purification *via* FCC (4 : 1 pentane/EtOAc) gave **4i** as a yellow oil (0.21 g, 44%), and as a 87(**A**) : 13(**B**) mixture of rotamers.

IR 1713 (C^8=O), 1525, 1489, 1443, 1294, 1244, 1209, 1066

^{1}H NMR ($CDCl_3$, 400 MHz) δ_H: 8.25–8.18 (m, 2H, $C^{11}H$), 7.61–7.52 (m, 2H, C^{10}), 7.42–7.28 (m, 3H, ArH), 7.12 (d, J = 7.3 Hz, 2H, ArH), 4.79 (s, 2H, C^3H_2, (**B**)), 4.43 (s, 2H, C^3H_2, (**A**)), 3.02 (s, 2H, C^2H_2, (**A**)), 2.78 (s, 2H, C^2H_2, (**B**)), 0.16 (s, 9H, C^1H_3, (**A**)), 0.02 (s, 9H, C^1H_3, (**B**)) ppm.

^{13}C NMR ($CDCl_3$, 101 MHz) δ_C: 170.0 (C^8), 149.3 (C^{12}), 144.0 (C^9), 137.1 (C^4), 130.2 (C^6), 129.1 (C^5), 128.8 (C^7), 127.8 (C^{10}), 125.0 (C^{11}), 56.0 (C^3), 39.0 (C^2), 0.0 (C^1) ppm.

HRMS (ES^+) exact mass calculated for $[M+H]^+$ ($C_{18}H_{23}N_2O_3Si^+$) requires **m/z** 343.1473, found **m/z** 343.1475.

N-benzyl-3-nitro-N-((trimethylsilyl)methyl)benzamide (4j)

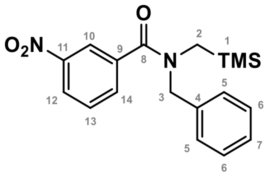

Prepared according to **General Procedure F**. Purification *via* FCC (4 : 1 pentane/EtOAc) gave **4j** as a white solid (0.21 g, 44%), and as a 87(**A**) : 13(**B**) mixture of rotamers.

mp 48-52 °C

IR 1626 (C^8=O), 1526, 1351, 1243, 842

^{1}H NMR ($CDCl_3$, 400 MHz) δ_H: 8.33-8.18 (m, 2H, C^{10}, C^{12}), 7.72 (d, J = 7.6 Hz, 1H, C^{14}H), 7.55 (t, J = 7.9 Hz, 1H, C^{13}H), 7.42-7.28 (m, 3H, ArH), 7.13 (d, J = 7.4 Hz, 2H, C^5H), 4.79 (s, 3H, C^3H_2, (**B**)), 4.47 (s, 3H, C^3H_2, (**A**)), 3.02 (s, 3H, C^2H_2, (**A**)), 2.82 (s, 3H, C^2H_2, (**B**)), 0.16 (s, 9H, C^1H_3, (**A**)), 0.04 (s, 9H, C^1H_3, (**B**)) ppm.

^{13}C NMR ($CDCl_3$, 101 MHz) δ_C: 169.4 (C^8), 149.2 (C^{11}), 139.4 (C^9), 137.1 (C^4), 133.7 (C^{14}), 130.8 (ArH), 130.2 (ArH), 129.1 (ArH), 127.8 (ArH), 125.3 (ArH), 123.1 (ArH), 56.3 (C^3), 39.2 (C^2), 0.0 (C^1) ppm.

HRMS (ES^+) exact mass calculated for $[M+H]^+$ ($C_{18}H_{23}N_2O_3Si^+$) requires **m/z** 343.1473, found **m/z** 343.1475.

***N*-benzyl-4-cyano-*N*-((trimethylsilyl)methyl)benzamide (4k)**

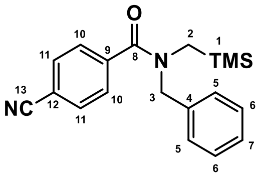

Prepared according to **General Procedure F**. Purification *via* FCC (4 : 1 pentane/EtOAc) gave **4k** as a clear oil (0.36 g, 84%), and as a 86(**A**) : 14(**B**) mixture of rotamers.

IR 1626 (C^8=O), 1451, 1247, 851

^{1}H NMR ($CDCl_3$, 400 MHz) δ_H: 7.64 (d, J = 8.0 Hz, 2H, $C^{10}H$), 7.49 (d, J = 8.1 Hz, 2H, $C^{11}H$), 7.41-7.27 (m, 3H, ArH), 7.11 (d, J = 7.3 Hz, 2H, ArH), 4.77 (s, 2H, C^3H_2, (B)), 4.42 (s, 2H, C^3H_2, (**A**)), 2.99 (s, 2H, C^2H_2, (**A**)), 2.77 (s, 2H, C^2H_2, (**B**)), 0.14 (s, 9H, C^1H_3, (**A**)), 0.01 (s, 9H, C^1H_3, (**B**)).

^{13}C NMR ($CDCl_3$, 101 MHz) δ_C: 170.2 (C^8), 142.2 (C^9), 137.2 (C^4), 133.5 (C^{11}), 130.1 (ArC), 129.0 (ArC), 128.5 (ArC), 127.8 (ArC), 119.2 (C^{13}), 114.3 (C^{12}), 56.1 (C^3), 39.0 (C^2), 0.0 (C^1).

HRMS (ES^+) exact mass calculated for $[M+H]^+$ ($C_{19}H_{23}N_2OSi^+$) requires **m/z** 323.1574, found **m/z** 323.1573.

***N*-methyl-*N*-((trimethylsilyl)methyl)thiophene-2-carboxamide (4l)**

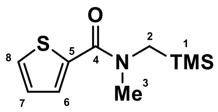

Prepared according to **General Procedure E**. Purification *via* FCC (4 : 1 pentane/EtOAc) gave **4l** as a yellow oil (1.5 g, 66%).

IR 1606 (C^4=O), 1525, 1393, 1247, 841

^{1}H NMR ($CDCl_3$, 400 MHz) δ_H: 7.41 (dd, J = 5.1, 1.1 Hz, 1H, C^6H), 7.31 (dd, J = 3.7, 1.1 Hz, 1H, C^8H), 7.03 (dd, J = 5.0, 3.7 Hz, 1H, C^7H), 3.21 (s, 3H, C^3H_3), 3.12 (s, 2H, C^2H_2), 0.13 (s, 9H, C^1H_3).

^{13}C NMR ($CDCl_3$, 101 MHz) δ_C: 172.4 (C^4), 139.6 (C^5), 130.1 (ArC), 129.7 (ArC), 128.0 (ArC), 43.1 (C^2), 41.6 (C^3), 0.0 (C^1).

HRMS (ES^+) exact mass calculated for $[M+H]^+$ ($C_{10}H_{18}NOSSi^+$) requires **m/z** 228.0873, found **m/z** 228.0875.

N-benzyl-N-((trimethylsilyl)methyl)furan-2-carboxamide (4m)

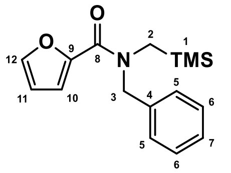

Prepared according to **General Procedure F**. Purification *via* FCC (4 : 1 pentane/EtOAc) gave **4m** as a yellow oil (0.26 g, 89%), and as a 77(**A**) : 23(**B**) mixture of rotamers.

IR 1614 (C^8=O), 1489, 1436, 1247, 843

^{1}H NMR ($CDCl_3$, 400 MHz) δ_H: 7.36 (s, 1H, $C^{12}H$), 7.32–7.11 (m, 5H, C^5H, C^6H and C^7H), 6.84 (s, 1H, $C^{10}H$), 6.36 (s, 1H, $C^{11}H$), 4.74 (s, 2H, C^3H_2), 3.11 (s, 2H, C^2H_2 (**B**)), 2.85 (s, 2H, C^2H_2 (**A**)), 0.00 (s, 9H, C^1H_3).

^{13}C NMR ($CDCl_3$, 101 MHz) δ_C: 160.8 (C^8), 149.2 (C^9), 144.7 (C^{12}), 138.2 (C^4), 129.9 (ArC), 128.7 (ArC), 128.3 (ArC), 116.6 (C^{10}), 112.4 (C^{11}), 55.4 (C^3), 40.2 (C^2), 0.0 (C^1).

HRMS (ES^+) exact mass calculated for $[M+H]^+$ ($C_{16}H_{22}NO_2Si^+$) requires **m/z** 288.1414, found **m/z** 288.1415.

***N*-benzyl-*N*-((trimethylsilyl)methyl)isobutyramide (4n)**

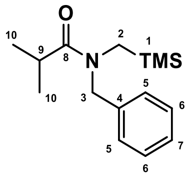

Prepared according to **General Procedure F**. Purification *via* FCC (4 : 1 pentane/EtOAc) gave **4n** as a white solid (0.10 g, 81%), and as a 75(**A**) : 25(**B**) mixture of rotamers.

mp 30–32 °C

IR 1625 (C^8=O), 1435, 1248, 1218, 845

^{1}H NMR ($CDCl_3$, 400 MHz) δ_H: 7.39–7.24 (m, 3H, ArH), 7.20–7.12 (m, 2H, ArH), 4.59 (s, 2H, C^3H_2, (**B**)), 4.54 (s, 2H, C^3H_2, (**A**)), 2.85 (s, 2H, C^2H_2, 2.84–2.74 (m, 1H, C^9H), 1.18 (d, *J* = 6.7 Hz, 2H, (**B**)), 1.13 (d, *J* = 6.7 Hz, 2H, (**A**)), 0.11 (s, 9H, C^1H_3, (**B**)), 0.06 (s, 9H, C^1H_3, (**A**)).

^{13}C NMR ($CDCl_3$, 101 MHz) δ_C: 177.6 (C^8), 139.0 (C^4), 138.4 (C^4), 130.0 (ArC), 129.7 (ArC), 129.0 (ArC), 128.7 (ArC), 128.4 (ArC), 127.5 (ArC), 54.4 (C^3, (**A**)), 51.1 (C^3, (**B**)), 39.9 (C^2, (**A**)), 39.1 (C^2, (**B**)), 31.5 (C^9, (**B**)), 31.3 (C^9, (**A**)), 21.1 (C^{10}, (**A**)), 20.5 (C^{10}, (**B**)), 0.0 (C^1, (**A**)), –0.4 (C^1, (**B**)).

HRMS (ES^+) exact mass calculated for $[M+H]^+$ ($C_{16}H_{22}NO_2Si^+$) requires **m/z** 288.1414, found **m/z** 288.1415.

4. Synthesis and characterization of pyrrolidines

methyl (2S,3R,7aR)-2-(2-oxooxazolidine-3-carbonyl)-3-phenyltetrahydro-1H-pyrrolizine-7a(5H)-carboxylate (3a)

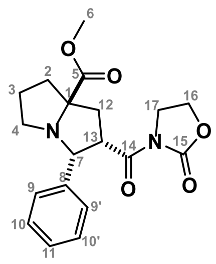

Prepared according to **General Procedure A** from **1a** and **2a**. Purification *via* FCC (4 : 1 pentane/EtOAc) gave **3a** as a pale yellow oil (77 mg, 86%).

IR 2953, 1778 (C^{14}=O), 1726 (C^{5}=O), 1698 (C^{15}=O), 1478, 1457, 1387, 1366, 1285, 1224, 1197.

^{1}H NMR ($CDCl_3$, 500 MHz) δ_H: 7.23–7.31 (m, 3H, C^{10}H, $C^{10'}$H, C^{11}H), 7.15–7.19 (2H, m, C^{9}H, $C^{9'}$H), 4.95 (d, 1H, *J* = 9.4 Hz, C^{7}H), 4.79 (ddd, 1H, *J* = 11.5, 9.3, 7.6 Hz, C^{13}H), 4.15 (td, 1H, *J* = 9.0, 5.7 Hz, C^{16}Ha), 3.83 (td, 1H, *J* = 8.9, 7.9 Hz, C^{16}Hb), 3.77 (s, 3H, $C^{6}H_3$), 3.70 (ddd, 1H, *J* = 10.9, 9.3, 7.8 Hz, C^{17}Ha), 3.04 (ddd, 1H, *J* = 10.9, 9.1, 5.6 Hz, C^{17}Hb), 2.65–2.74 (m, 2H, $C^{4}H_2$), 2.45–2.56 (m, 2H, $C^{12}H_2$) 2.35 (ddd, 1H, *J* = 11.4, 6.7, 3.8 Hz, C^{2}Ha), 1.89–2.06 (m, 3H, C^{2}Hb, $C^{3}H_2$).

^{13}C NMR ($CDCl_3$, 126 MHz) δ_C: 177.1 (C^5), 172.0 (C^{14}), 152.8 (C^{15}), 138.1 (C^8), 129.4 (C^9, $C^{9'}$), 128.2 (C^{10}, $C^{10'}$), 128.1 (C^{11}), 76.1 (C^1), 65.2 (C^7), 62.1 (C^{16}), 53.0 (C^{13}), 52.4 (C^6), 46.4 (C^4), 42.7 (C^{17}), 36.1 (C^{12}), 35.8 (C^2), 28.0 (C^3).

HRMS (ES^+) exact mass calculated for $[M+H]^+$ ($C_{19}H_{23}O_5N_2^+$) requires **m/z** 359.1600, found **m/z** 359.1601.

methyl (2S,3S,7aR)-2-methyl-2-(2-oxooxazolidine-3-carbonyl)-3-phenyltetrahydro-1H-pyrrolizine-7a(5H)-carboxylate (3b)

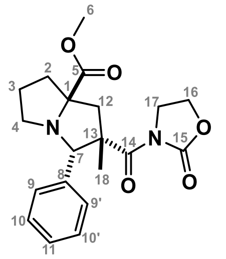

Prepared according to **General Procedure A** from **1a and 2b**. Purification *via* FCC (4 : 1 pentane/EtOAc) gave **3b** as a white solid (77 mg, 83%).

mp 98–100 °C

IR 2951 (br, C–H), 1783 (C^{14}=O), 1725 (C^{5}=O), 1680 (C^{15}=O), 1476, 1383, 1360, 1308, 1276, 1201.

^{1}H NMR ($CDCl_3$, 500 MHz) δ_H: 7.24–7.30 (m, 3H, C^{10}H, $C^{10'}$H, C^{11}H), 7.15–7.19 (m, 2H, C^{9}H, $C^{9'}$H), 4.99 (s, 1H, C^{7}H), 4.08 (td, 1H, *J* = 8.8, 5.4 Hz, C^{16}Ha), 3.78 (s, 3H, $C^{6}H_3$), 3.73 (ddd, 1H, *J* = 10.9, 9.0, 8.1 Hz, C^{17}Ha), 3.56 (app q, 1H, *J* = 8.5 Hz, C^{16}Hb), 3.22 (ddd, 1H, *J* = 10.9, 8.5, 5.4 Hz, C^{17}Hb), 2.93 (roofed d, 1H, *J* = 14.1 Hz, C^{12}Ha), 2.84 (roofed d, 1H, *J* = 14.1 Hz, C^{12}Hb), 2.78–2.85 (m, 1H, C^{4}Ha), 2.52 (dt, 1H, *J* = 10.7, 6.8 Hz, C^{4}Hb), 2.13 (ddd, 1H, *J* = 12.8, 7.5, 5.3 Hz, C^{2}Ha), 1.92 (dt, 1H, *J* = 12.8, 8.2 Hz, C^{2}Hb), 1.68–1.77 (m, 4H, C^{3}Ha, $C^{18}H_3$), 1.55–1.64 (m, 1H, C^{3}Hb).

^{13}C NMR ($CDCl_3$, 126 MHz) δ_C: 178.9 (C^{5}), 174.9 (C^{14}), 151.2 (C^{15}), 139.4 (C^{8}), 129.5 (br, C^{9}, $C^{9'}$), 128.4 (C^{10}, $C^{10'}$), 127.9 (C^{11}), 75.2 (C^{1}), 71.4 (C^{7}), 62.2 (C^{16}), 60.3 (C^{13}), 52.7 (C^{6}), 48.6 (C^{4}), 47.9 (C^{12}), 44.4 (C^{17}), 39.8 (C^{2}), 27.4 (C^{3}), 23.2 (C^{18}).

HRMS (ES^+) exact mass calculated for $[M+H]^+$ ($C_{20}H_{25}O_5N_2^+$) requires **m/z** 373.1758, found **m/z** 373.1758.

methyl (1S,2S,3R,7aR)-1-methyl-2-(2-oxooxazolidine-3-carbonyl)-3-phenyltetrahydro-1H-pyrrolizine-7a(5H)-carboxylate (3c)

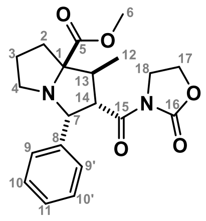

Prepared according to **General Procedure A**. from **1a** and **3c**. Purification *via* FCC (4 : 1 pentane/EtOAc) gave **3c** as a pale yellow oil (48 mg, 52%).

IR 1777 (C^{15}=O), 1724 (C^{5}=O), 1692 (C^{16}=O), 1386, 1221, 703.

^{1}H NMR ($CDCl_3$, 400 MHz) δ_H: 7.31–7.21 (m, 3H, C^{10}H, $C^{10'}$H C^{11}H), 7.13–7.18 (m, 2H, C^{9}H, $C^{9'}$H), 5.10 (d, 1H, J = 9.5 Hz, C^{7}H), 4.60 (dd, 1H, J = 11.7, 9.5 Hz, C^{14}H), 4.14 (ddd, 1H, J = 9.3, 8.6, 5.7 Hz, C^{17}Ha), 3.85–3.78 (m, 1H, C^{17}Hb), 3.77 (s, 3H, $C^{6}H_3$), 3.67 (ddd, 1H, J = 10.8, 9.3, 7.8 Hz, C^{18}Ha), 2.95 (ddd, 1H, J = 10.8, 9.1, 5.7 Hz, C^{18}Hb), 2.83 (dq, 1H, J = 11.6, 6.7 Hz, C^{13}H), 2.68–2.56 (m, 2H, C^{2}Ha, C^{4}Ha), 2.50 (ddd, 1H, J = 9.2, 7.3, 4.3 Hz, C^{4}Hb), 1.91–2.01 (m, 1H, C^{3}Ha), 1.80–1.90 (m, 1H, C^{3}Hb) 1.72 (ddd, 1H, J = 12.1, 9.5, 7.9 Hz, C^{2}Hb), 0.97 (d, 3H, J = 6.8 Hz, $C^{12}H_3$).

^{13}C NMR ($CDCl_3$, 101 MHz) δ_C: 176.0 (C^{5}), 171.7 (C^{15}), 152.8 (C^{16}), 138.2 (C^{8}), 129.4 (C^{9}), 128.1 (C^{10}), 127.9 (C^{11}), 79.9 (C^{1}), 64.8 (C^{7}), 62.1 (C^{17}), 60.1 (C^{14}), 51.9 (C^{6}), 46.11 (C^{4}), 43.20 (C^{13}), 42.5 (C^{18}), 34.2 (C^{2}), 28.0 (C^{3}), 14.3 (C^{12}).

HRMS (ES^+) exact mass calculated for $[M+H]^+$ ($C_{20}H_{25}N_2O_5^+$) requires **m/z** 373.1758, found **m/z** 373.1758.

methyl (1R,2S,3R,7aR)-2-(2-oxooxazolidine-3-carbonyl)-1,3-diphenyltetrahydro-1H-pyrrolizine-7a(5H)-carboxylate (3d)

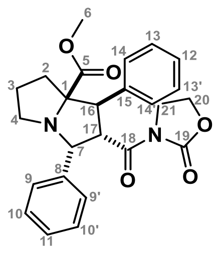

Prepared according to **General Procedure A** from **1a** and **2d**. Purification *via* FCC (6 : 1 pentane/EtOAc) gave **3d** as a pale yellow oil (69 mg, 64%).

IR 2949 (C–H), 1778 (C^{18}=O), 1727 (C^{5}=O), 1697 (C^{19}=O), 1387, 1280, 1223.

^{1}H NMR ($CDCl_3$, 500 MHz) δ_H: 7.19–7.36 (m, 10H, C^9H, $C^{9'}H$, $C^{10}H$, $C^{10'}H$, $C^{11}H$, $C^{12}H$, $C^{13}H$, $C^{13'}H$, $C^{14}H$, $C^{14'}H$), 5.63 (dd, 1H, J = 11.9, 9.5 Hz, $C^{17}H$), 5.28 (d, 1H, J = 9.5 Hz, C^7H), 4.09–4.15 (m, 2H, $C^{16}H$, $C^{20}Ha$), 3.84 (app td, 1H, J = 9.0, 7.6 Hz, $C^{20}Hb$), 3.59 (ddd, 1H, J = 10.9, 9.3, 7.5 Hz, $C^{21}Ha$), 3.49 (s, 3H, C^6H_3), 2.91 (ddd, 1H, J = 10.9, 9.2, 6.0 Hz, $C^{21}Hb$), 2.76 (app dt, 1H, J = 8.4, 7.0 Hz, C^4Ha), 2.62–2.67 (m, 1H, C^2Ha), 2.55 (ddd, 1H, J = 8.7, 7.3, 3.9 Hz, C^4Hb), 2.06–2.13 (m, 1H, C^3Ha), 1.88–2.01 (m, 2H, C^2Hb, C^3Hb).

^{13}C NMR ($CDCl_3$, 126 MHz) δ_C: 174.9 (C^5), 171.1 (C^{18}), 153.1 (C^{19}), 138.3 (C^8), 137.1 (C^{15}), 129.4 (C^9, $C^{9'}$), 128.6 (C^{10} or C^{13}, $C^{10'}$ or $C^{13'}$), 128.2 (C^{10} or C^{13}, $C^{10'}$ or $C^{13'}$), 128.1 (C^{11} or C^{12}), 127.7 (C^{14}, $C^{14'}$), 127.5 (C^{11} or C^{12}), 81.1 (C^1), 64.2 (C^7), 62.1 (C^{20}), 57.3 (C^{17}), 53.6 (C^{16}), 51.7 (C^6), 45.2 (C^4), 42.5 (C^{21}), 34.4 (C^2), 28.0 (C^3).

HRMS (ES^+) exact mass calculated for $[M+H]^+$ ($C_{25}H_{27}N_2O_5^+$) requires **m/z** 435.1914, found **m/z** 435.1908.

2-(tert-butyl) 7a-methyl (2S,3R,7aR)-3-phenyltetrahydro-1H-pyrrolizine-2,7a(5H)-dicarboxylate (3e)

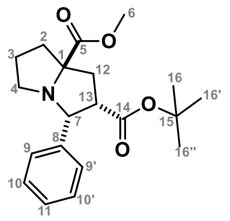

Prepare according to **General Procedure A** from **1a** and **2e** and the corresponding (2.5 equiv.). Purification *via* FCC (93 : 7 to 90 : 10 pentane/EtOAc) gave **3e** as a pale yellow oil (71 mg, 82%).

IR 2976, 1727 (C^5=O, C^{14}=O), 1458, 1392, 1156.

^{1}H NMR ($CDCl_3$, 500 MHz) δ_H: 7.22–7.32 (m, 3H, C^{10}H, $C^{10'}$H, C^{11}H), 7.16–7.20 (m, 2H, C^9H, $C^{9'}$H), 4.66 (d, 1H, *J* = 8.8 Hz, C^7H), 3.81 (ddd, 1H, *J* = 13.2, 8.9, 7.2 Hz, C^{13}H), 3.75 (s, 3H, C^6H_3), 2.67–2.73 (m, 1H, C^4Ha), 2.52–2.60 (m, 2H, C^4Hb, C^{12}Ha), 2.29–2.40 (m, 2H, C^2Ha, C^{12}Hb), 1.77–1.93 (m, 3H, C^2Hb, C^3H_2), 0.98 (s, 9H, $C^{16}H_3$, $C^{16'}H_3$, $C^{16''}H_3$).

^{13}C NMR ($CDCl_3$, 126 MHz) δ_C: 177.7 (C^5), 170.8 (C^{14}), 138.7 (C^8), 129.8 (br, C^9, $C^{9'}$), 128.3 (C^{10}, $C^{10'}$), 127.8 (C^{11}), 80.5 (C^{15}), 75.2 (C^1), 67.6 (C^7), 53.0 (C^{13}), 52.5 (C^6), 47.6 (C^4), 37.2 (C^{12}), 36.5 (C^2), 28.3 (C^3), 27.5 (C^{16}, $C^{16'}$, $C^{16''}$).

HRMS (ES^+) exact mass calculated for $[M+H]^+$ ($C_{20}H_{28}O_4N^+$) requires **m/z** 346.2013, found **m/z** 346.2014.

methyl (3aS,4R,8aR,8bR)-1-oxo-4-phenylhexahydro-3H-furo[3,4-a]pyrrolizine-8a(6H)-carboxylate (3f)

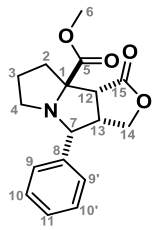

Prepared according to **General Procedure A** from **1a** and **2f**. Purification *via* FCC (1 : 4 pentane/EtOAc) gave **3f** as a white solid (30 mg, 40%).

mp 90–92 °C

IR 2981, 1765 (C^{15}=O, ester), 1728 (C^{5}=O, ester), 1250, 1161.

^{1}H NMR ($CDCl_3$, 400 MHz) δ_H: 7.34–7.40 (m, 2H, C^{10}H, $C^{10'}$H), 7.26–7.32 (m, 1H, C^{11}H), 7.20–7.24 (m, 2H, C^{9}H, $C^{9'}$H), 4.37 (d, 1H, *J* = 7.5 Hz, C^{7}H), 4.07 (dd, 1H, *J* = 10.1, 7.6 Hz, C^{14}Ha), 3.78 (s, 3H, $C^{6}H_3$), 3.72 (dd, 1H, *J* = 10.1, 2.6 Hz, C^{14}Hb), 3.54 (app qd, 1H, *J* = 7.7, 2.5 Hz, C^{13}H), 3.47 (roofed d, 1H, *J* = 8.3 Hz, C^{12}H), 2.71–2.82 (m, 2H, $C^{4}H_2$), 2.50–2.57 (m, 1H, C^{2}Ha), 2.02–2.20 (m, 3H, C^{2}Hb, $C^{3}H_2$).

^{13}C NMR ($CDCl_3$, 101 MHz) δ_C: 176.1 (C^{5}), 174.5 (C^{15}), 137.6 (C^{8}), 128.8 (C^{10}), 128.2 (C^{9}), 127.9 (C^{11}), 78.5 (C^{1}), 68.4 (C^{14}), 62.7 (C^{7}), 52.1 (C^{6}), 48.9 (C^{13}), 46.6 (C^{12}), 43.0 (C^{4}), 27. 5 (C^{2}), 26.6 (C^{3}).

HRMS (ES^+) exact mass calculated for $[M+H]^+$ ($C_{17}H_{20}NO_4^+$) requires m/z 302.1387, found m/z 302.1387.

methyl (1S,2S,3S,7aR)-2-nitro-1,3-diphenyltetrahydro-1H-pyrrolizine-7a(5H)-carboxylate (3g1) and methyl (1R,2S,3R,7aR)-1-nitro-2,3-diphenyltetrahydro-1H-pyrrolizine-7a(5H)-carboxylate (3g2)

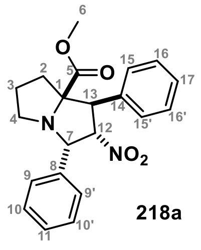

218a

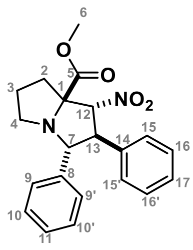

218b

Prepared according to **General Procedure A** from **1a and 2g**. Purification *via* FCC (9 : 1 pentane/EtOAc) gave **3g1** as a pale yellow oil (41 mg, 44%).

Data for **3g1**:

^{1}H NMR ($CDCl_3$, 500 MHz) δ_H: 7.26–7.39 (m, 8H, C^{9}H, C$^{9'}$H, C^{10}H, C$^{10'}$H, C^{11}H, C^{16}H, C$^{16'}$H, C^{17}H), 7.19–7.23 (m, 2H, C^{15}H, C$^{15'}$H), 6.36 (dd, 1H, *J* = 11.1, 8.9 Hz, C^{12}H), 5.27 (d, 1H, *J* = 8.8 Hz, C^{7}H), 4.31 (d, 1H, *J* = 11.2 Hz, C^{13}H), 3.43 (s, 3H, C^{6}H$_3$), 2.88 (dt, 1H, *J* = 8.7, 7.3 Hz, C^{4}Ha), 2.73–2.79 (m, 1H, C^{2}Ha), 2.61 (td, 1H, *J* = 8.2, 3.4 Hz, C^{4}Hb), 2.17–2.24 (m, 1H, C^{3}Ha), 1.96–2.06 (m, 2H, C^{2}Hb, C^{3}Hb).

^{13}C NMR ($CDCl_3$, 126 MHz) δ_C: 174.1 (C^{5}), 134.9 (C^{8}), 134.4 (C^{14}), 129.3 (C^{9}, C$^{9'}$), 129.1 (C^{11}), 128.9 (C^{10} or C^{16}), 128.6 (C^{10} or C^{16}), 128.1 (C^{17}), 127.2 (C^{15}, C$^{15'}$), 94.0 (C^{12}), 80.1 (C^{1}), 65.6 (C^{7}), 53.9 (C^{13}), 51.9 (C^{6}), 44.9 (C^{4}), 34.3 (C^{2}), 27.8 (C^{3}).

All other data in perfect agreement with literature.[7]

Further elution (9 : 1 pentane/EtOAc) gave regioisomer **3g2** as a pale yellow oil (41 mg, 44%).

Data for **3g2**:

^{1}H NMR ($CDCl_3$, 500 MHz) δ_H: 7.35–7.39 (m, 4H, C^9H, $C^{9'}H$, $C^{15}H$, $C^{15'}H$), 7.25–7.33 (m, 4H, $C^{10}H$, $C^{10'}H$, $C^{16}H$, $C^{16'}H$), 7.17–7.24(m, 2H, $C^{11}H$, $C^{17}H$), 5.95 (d, 1H, *J* = 10.2 Hz, $C^{12}H$), 4.85 (d, 1H, *J* = 12.3 Hz, C^7H), 4.44 (dd, 1H, *J* = 12.3, 10.2 Hz, $C^{13}H$), 3.93 (s, 3H, C^6H_3), 2.64 (ddd, 1H, *J* = 11.5, 9.1, 5.6 Hz, C^4Ha), 2.45–2.51 (m, 2H, C^2Ha, C^4Hb), 1.86–1.94 (m, 1H, C^3Ha), 1.80–1.86 (m, 1H, C^3Hb), 1.60 (ddd, 1H, *J* = 13.4, 11.8, 7.2 Hz, C^2Hb).

^{13}C NMR ($CDCl_3$, 126 MHz) δ_C: 173.6 (C^5), 136.4 (C^{14}), 134.9 (C^8), 129.4 (C^9, $C^{9'}$), 129.2 (C^{10} or C^{16}), 128.5 (C^{10} or C^{16}), 128.2 (C^{11} or C^{17}), 128.1 (C^{15}, $C^{15'}$), 127.9 (C^{11} or C^{17}), 97.7 (C^{12}), 76.3 (C^1), 68.0 (C^7), 53.6 (C^6), 51.0 (C^4), 48.2 (C^{13}), 32.2 (C^2), 25.6 (C^3).

All other data in perfect agreement with literature.[7]

dimethyl (1R,2S,3R,7aR)-2,3-diphenyltetrahydro-1H-pyrrolizine-1,7a(5H)-dicarboxylate (3h)

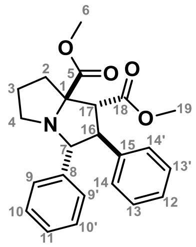

Prepared according to **General Procedure A** from **1a** and **2h** (2.5 equiv.). Purification *via* FCC (3 : 1 pentane/EtOAc) gave **3h** as a colourless oil that crystallized upon standing (61 mg, 64%). Crystals for X-ray analysis were obtained from slow evaporation from EtOAc.

mp 136–138 °C (significant sublimation happens from 100 °C).

IR 2951, 1734 (C^{14}=O, C^{5}=O), 1436, 1224, 1167.

^{1}H NMR ($CDCl_3$, 500 MHz) δ_H: 7.36–7.40 (m, 4H, C^{9}H, $C^{9'}$H, C^{14}H, $C^{14'}$H), 7.21–7.26 (m, 4H, C^{10}H, $C^{10'}$H, C^{13}H, $C^{13'}$H), 7.16–7.20 (m, 1H, C^{11}H), 7.11–7.16 (m, 1H, C^{12}H), 4.83 (d, 1H, *J* = 11.6 Hz, C^{7}H), 3.99 (app roofed t, 1H, *J* = 11.2 Hz, C^{16}H), 3.92 (d, 1H, *J* = 11.2 Hz, C^{17}H), 3.89 (s, 3H, $C^{6}H_3$), 3.63 (s, 3H, $C^{19}H_3$), 2.53 (ddd, 1H, *J* = 11.1, 9.0, 5.8 Hz, C^{4}Ha), 2.47 (ddt, 1H, *J* = 12.8, 6.0, 1.5 Hz, C^{2}Ha), 2.42 (ddt, 1H, *J* = 8.9, 5.9, 1.5 Hz, C^{4}Hb), 1.71–1.84 (m, 2H, $C^{3}H_2$), 1.62 (td, 1H, *J* = 12.4, 7.0 Hz, C^{2}Hb).

^{13}C NMR ($CDCl_3$, 126 MHz) δ_C: 175.4 (C^{5}), 172.5 (C^{18}), 138.9 (C^{15}), 136.1 (C^{8}), 129.7 (C^{9}, $C^{9'}$), 128.7 (C^{14}, $C^{14'}$), 128.3 (C^{10}, $C^{10'}$, C^{13}, $C^{13'}$), 127.7 (C^{11}), 127.0 (C^{12}), 75.5 (C^{1}), 70.3 (C^{7}), 60.2 (C^{17}), 53.1 (C^{6}), 52.0 (C^{19}), 51.0 (C^{4}), 47.0 (C^{16}), 32.9 (C^{2}), 25.6 (C^{3}).

HRMS (ES^{+}) exact mass calculated for $[M+H]^{+}$ ($C_{23}H_{26}O_4N^{+}$) requires **m/z** 380.1856, found **m/z** 380.1857.

methyl (2S,3S,7aR)-3-phenyl-2-(phenylsulfonyl)tetrahydro-1H-pyrrolizine-7a(5H)-carboxylate (3i1) and methyl (1R,3R,7aS)-3-phenyl-1-(phenylsulfonyl)tetrahydro-1H-pyrrolizine-7a(5H)-carboxylate (3i2)

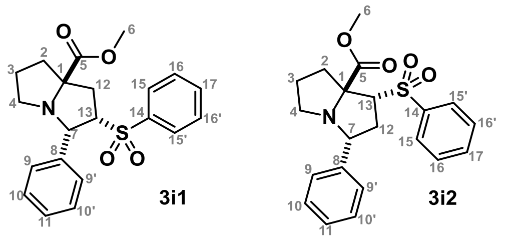

Prepared according to **General Procedure A** from **1a** and **2i**. Purification *via* FCC (98 : 2 to 60 : 40 pentane/EtOAc) gave a 53 : 47 mixture of **3i1** (**A**) and **3i2** (**B**), respectively, as a white solid (74 mg, 77%).

mp 104–106 °C (from EtOAc).

IR 1728 (C^5=O), 1447, 1291 (S=O), 1149, 1086, 721, 690.

^{1}H NMR ($CDCl_3$, 400 MHz) δ_H: 7.96–8.01 (m, 0.94H, $C^{15}H$ (**B**), $C^{15'}H$ (**B**)), 7.64–7.70 (m, 0.47H, $C^{17}H$ (**B**)), 7.52–7.62 (m, 1.47H, $C^{17}H$ (**A**), $C^{16}H$ (**B**), $C^{16'}H$ (**B**)), 7.42–7.46 (m, 1.06H, $C^{15}H$ (**A**), $C^{15'}H$ (**A**)), 7.23–7.40 (m, 5H, C^9H (**B**), $C^{9'}H$ (**B**), $C^{10}H$ (**A**), $C^{10'}H$ (**A**), $C^{10}H$ (**B**), $C^{10'}H$ (**B**), $C^{11}H$ (**A**), $C^{11}H$ (**B**), $C^{16}H$ (**A**), $C^{16'}H$ (**A**)), 7.05–7.09 (m, 1.06H, C^9H (**A**), $C^{9'}H$ (**A**)), 4.62 (dd, 0.47H, *J* = 12.3, 6.2 Hz, C^7H (**B**)), 4.51–4.60 (m, 1.06H, C^7H (**A**), $C^{13}H$ (**A**)), 4.38 (dd, 0.47H, *J* = 12.8, 4.0 Hz, $C^{13}H$ (**B**)), 3.76 (s, 1.41H, C^6H_3 (**B**)), 3.73 (s, 1.59H, C^6H_3 (**A**)), 2.35–2.78 (m, 5H, C^2H_2 (**B**), C^2Ha (**A**), C^4H_2 (**A**), C^4H_2 (**B**), $C^{12}H_2$ (**A**), $C^{12}Ha$ (**B**)), 2.15 (ddd, 0.47H, *J* = 11.6, 6.3, 4.1 Hz, $C^{12}Hb$ (**B**)), 1.86–1.61 (m, 2.53H, C^2Hb (**A**), C^3H_2 (**A**), C^3H_2 (**B**)).

^{13}C NMR ($CDCl_3$, 101 MHz) δ_C: 176.4 (C^5 (**B**)), 174.6 (C^5 (**A**)), 140.7 (C^{14} (**B**)), 139.2 (C^{14} (**A**)), 136.7 (C^8 (**B**)), 134.7 (C^8 (**A**)), 133.9 (C^{17} (**B**)), 133.4 (C^{17} (**A**)), 131.0 (C^9, $C^{9'}$ (**A**)), 129.4 (C^{16}, $C^{16'}$ (**B**)), 129.0 (C^{16} (**A**), $C^{16'}$ (**A**)), 128.7 (C^9, $C^{9'}$ (**B**)), 128.5 (C^{11} (**B**)), 128.4 (C^{11} (**A**)), 128.23 (C^{15} (**A**)), 128.19 (C^{15} (**B**)), 127.99 (C^{10}, $C^{10'}$(**B**)), 127.97 (C^{10}, $C^{10'}$ (**A**)), 76.2 (C^1 (**A**)), 75.7 (C^1 (**B**)), 70.1 (C^7 (**A**)), 68.5 (C^7 (**B**)), 66.2 (C^{13} (**A**)), 64.0 (C^{13} (**B**)), 53.1 (C^6 (**B**)), 52.7 (C^6 (**A**)), 50.4 (C^4 (**B**)), 45.9 (C^4 (**A**)), 36.1 (C^2(**A**)), 35.4 (C^2 (**B**)), 31.6 (C^{12} (**A**)), 29.5 (C^{12} (**B**)), 28.2 (C^3 (**A**)), 26.3 (C^3 (**B**)).

HRMS (ES$^+$) exact mass calculated for $[M+H]^+$ ($C_{21}H_{24}NO_4S^+$) requires m/z 386.1421, found **m/z** 386.1420.

ethyl (2S,3R,7aR)-2-(2-oxooxazolidine-3-carbonyl)-3-phenyltetrahydro-1H-pyrrolizine-7a(5H)-carboxylate (3j)

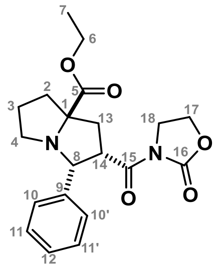

Prepared according to **General Procedure A** from **1j** and **2a**. Purification *via* FCC (8 : 1 pentane/EtOAc) gave **3j** as a pale yellow oil (66 mg, 71%).

IR 2979, 1778 (C^{15}=O), 1721 (C^{5}=O), 1698 (C^{16}=O), 1457, 1387, 1366, 1284, 1095.

^{1}H NMR ($CDCl_3$, 500 MHz) δ_H: 7.22–7.30 (m, 3H, $C^{11}H$, $C^{11'}H$, $C^{12}H$), 7.14–7.19 (m, 2H, $C^{10}H$, C10'H), 4.94 (d, 1H, *J* = 9.3 Hz, $C^{8}H$), 4.82 (ddd, 1H, *J* = 11.6, 9.3, 7.4 Hz, $C^{14}H$), 4.22 (q, 2H, *J* = 7.1 Hz, $C^{6}H_2$), 4.15 (td, 1H, *J* = 9.0, 5.6 Hz, C^{17}Ha), 3.82 (td, 1H, *J* = 8.9, 8.0 Hz, C^{17}Hb), 3.69 (ddd, 1H, *J* = 11.0, 9.4, 7.9 Hz, C^{18}Ha), 3.02 (ddd, 1H, *J* = 10.8, 9.1, 5.6 Hz, C^{18}Hb), 2.65–2.73 (m, 2H, $C^{4}H_2$), 2.42–2.54 (m, 2H, $C^{13}H_2$), 2.35 (ddd, 1H, *J* = 12.2, 7.0, 3.9 Hz, C^{2}Ha), 1.97–2.04 (m, 2H, $C^{3}H_2$), 1.90 (ddd, 1H, *J* = 12.3, 9.7, 7.9 Hz, C^{2}Hb), 1.32 (t, 3H, *J* = 7.1 Hz, $C^{7}H_3$).

^{13}C NMR ($CDCl_3$, 126 MHz) δ_C: 176.4 (C^5), 172.0 (C^{15}), 152.9 (C^{16}), 138.3 (C^9), 129.3 (C^{10}, $C^{10'}$), 128.2 (C^{11}, $C^{11'}$), 128.0 (C^{12}), 76.1 (C^1), 64.9 (C^8), 62.1 (C^{17}), 61.0 (C^6), 53.1 (C^{14}), 46.1 (C^4), 42.6 (C^{18}), 35.9 (C^{13}), 35.5 (C^2), 28.0 (C^3), 14.4 (C^7).

HRMS (ES$^+$) exact mass calculated for $[M+H]^+$ ($C_{20}H_{25}O_5N_2^+$) requires **m/z** 373.1758, found **m/z** 373.1759.

methyl (2R,3S,5S)-3-(2-oxooxazolidine-3-carbonyl)-2-phenyl-1-azabicyclo[3.2.0]heptane-5-carboxylate (3k)

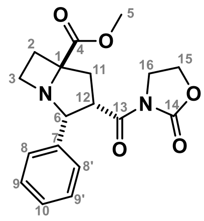

Prepared according to **General Procedure A** from **1k** and **2a**. Purification *via* FCC (1 : 1 pentane/EtOAc) gave **3k** as a pale yellow oil (60 mg, 69%).

mp 94–96 °C

^{1}H NMR ($CDCl_3$, 500 MHz) δ_H: 7.24–7.31 (m, 5H, C^8H, $C^{8'}H$, C^9H, $C^{9'}H$, $C^{10}H$), 4.98 (roofed d, 1H, J = 9.8 Hz, C^6H), 4.89 (dt, 1H, J = 9.8, 8.5 Hz, $C^{12}H$), 4.15 (ddd, 1H, J = 9.0, 8.1, 5.6 Hz, $C^{15}Ha$), 3.93 (app q, 1H, J = 8.1 Hz, C^3Ha), 3.80–3.87 (m, 1H, $C^{16}Ha$), 3.79 (s, 3H, C^5H_3), 3.71–3.79 (m, 1H, $C^{15}Ha$), 3.42 (ddd, 1H, J = 10.0, 8.6, 5.6 Hz, $C^{16}Hb$), 3.12 (td, 1H, J = 8.2, 3.0 Hz, C^3Hb), 3.07 (dd, 1H, J = 14.0, 8.3 Hz, $C^{11}Ha$), 2.74 (dt, 1H, J = 10.8, 8.5 Hz, C^2Ha), 2.62 (dd, 1H, J = 14.0, 8.8 Hz, $C^{11}Hb$), 2.26 (ddd, 1H, J = 11.0, 8.2, 3.0 Hz, C^2Hb).

^{13}C NMR ($CDCl_3$, 126 MHz) δ_C: 174.9 (C^{13}), 174.0 (C^4), 152.8 (C^{14}), 135.8 (C^7), 129.2 (C^9, $C^{9'}$), 128.2 (C^8, $C^{8'}$), 127.9 (C^{10}), 75.0 (C^1), 70.9 (C^6), 61.9 (C^{15}), 52.5 (C^5), 52.0 (C^{12}), 44.6 (C^3), 42.9 (C^{16}), 38.1 (C^{11}), 27.2 (C^2).

HRMS (ES^+) exact mass calculated for $[M+H]^+$ ($C_{18}H_{21}O_5N_2^+$) requires **m/z** 345.1445, found **m/z** 345.1445.

methyl (1S,3R,7aS)-1-(2-oxooxazolidine-3-carbonyl)hexahydro-1H-pyrrolizine-3-carboxylate (3l1) and methyl (2S,3R,7aS)-2-(2-oxooxazolidine-3-carbonyl)hexahydro-1H-pyrrolizine-3-carboxylate (3l2)

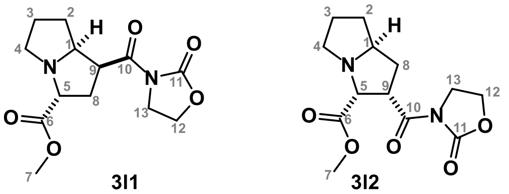

Prepared according to **General Procedure A** from **1l** and **2a** to afford an 82 : 18 mixture of regioisomers **3l1** and **3l2**, respectively. Purification *via* FCC (3 : 7 pentane/EtOAc) gave a 93 : 7 mixture of **3l1** and **3l2**, respectively (46 mg, 65%).

Data for **3l1**

IR 2955, 1776 (C^{10}=O), 1738 (C^{6}=O), 1691 (C^{11}=O), 1387, 1266, 1204.

^{1}H NMR ($CDCl_3$, 500 MHz) δ_H: 4.39–4.44 (m, 2H, $C^{12}H_2$), 4.33 (td, 1H, *J* = 8.0, 6.0 Hz, C^9H), 4.16 (td, 1H, *J* = 8.7, 6.8 Hz, C^1H), 3.96–4.09 (m, 2H, $C^{13}H_2$), 3.73 (s, 3H, C^7H_3), 3.69–3.74 (m, 1H, C^5H), 3.21 (ddd, 1H, *J* = 9.9, 6.1, 4.2 Hz, C^4Ha), 2.57–2.63 (m, 2H, C^4Hb, C^8Ha), 2.22 (ddd, 1H, *J* = 13.0, 7.6, 6.5 Hz, C^8Hb), 1.84–1.91 (m, 2H, C^3H_2), 1.76 (1H, dtd, *J* = 12.1, 6.5, 4.4 Hz, C^2Ha), 1.20–1.29 (1H, m, C^2Hb).

^{13}C NMR ($CDCl_3$, 126 MHz) δ_C: 174.4 (C^6), 173.6 (C^{10}), 153.2 (C^{11}), 67.6 (C^5), 66.8 (C^1), 62.1 (C^{12}), 55.3 (C^4), 52.3 (C^7), 45.3 (C^9), 42.8 (C^{13}), 32.6 (C^8), 28.2 (C^2), 27.3 (C^3).

HRMS (ES^+) exact mass calculated for $[M+H]^+$ ($C_{13}H_{19}O_5N_2^+$) requires **m/z** 283.1288, found **m/z** 283.1288.

Further elution (3 : 7 pentane/EtOAc) gave a 2 : 5 mixture of **311** and **312**, respectively.
Data for **312** (selected peaks):

^{1}H NMR ($CDCl_3$, 400 MHz) δ_H [selected peaks]: 4.53 (ddd, 1H, J = 11.5, 9.3, 6.9 Hz, C^9H), 3.92 (d, 1H, J = 9.3 Hz, C^5H), 3.72 (s, 3H, C^7H_3), 3.15 (dt, 1H, J = 11.6, 6.1 Hz, C^4Ha), 2.80 (dt, 1H, J = 10.4, 6.4 Hz, C^4Hb), 2.49 (dt, 1H, J = 11.9, 6.6 Hz, C^8Ha).

^{13}C NMR ($CDCl_3$, 126 MHz) δ_C [selected peaks]: 70.6 (C^5), 55.4 (C^4), 48.3 (C^9), 36.9 (C^8).

Scale-up : Reaction performed on 2 mmol scale without further modifications gave 367 mg, 65% (combined).

benzyl (1S,3R,7aS)-1-(2-oxooxazolidine-3-carbonyl)hexahydro-1H-pyrrolizine-3-carboxylate (3m1) and benzyl (2S,3R,7aS)-2-(2-oxooxazolidine-3-carbonyl)hexahydro-1H-pyrrolizine-3-carboxylate (3m2)

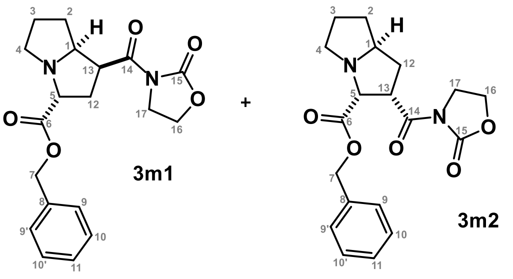

Prepared according to **General Procedure A** from **1m** and **2a** to afford a 87 : 13 mixture of regiosisomers **3m1** and **3m2**, respectively. Purification *via* FCC (1 : 1 pentane/EtOAc) gave a 87 : 13 mixture of **3m1** and **3m2**, respectively (56 mg, 63%).

Data for **3m1**:

IR 2959, 1777 (C^{14}=O), 1739 (C^{6}=O), 1692 (C^{15}=O), 1387, 1265, 1218.

^{1}H NMR ($CDCl_3$, 500 MHz) δ_H: 7.29–7.38 (m, 5H, C^9H, $C^{9'}$H, C^{10}H, $C^{10'}$H, C^{11}H), 5.19 (very roofed d, 1H, *J* = 12.4 Hz, C^7Ha), 5.16 (very roofed d, 1H, *J* = 12.4 Hz, C^7Hb), 4.41 (ddd, 2H, *J* = 8.8, 7.3, 1.8 Hz, $C^{16}H_2$), 4.34 (td, 1H *J* = 8.0, 5.9 Hz, C^{13}Ha), 4.19 (td, 1H, *J* = 8.8, 6.9 Hz, C^1H), 3.96–4.08 (m, 2H, $C^{17}H_2$), 3.75 (t, 1H, *J* = 7.0 Hz, C^5H), 3.22 (ddd, 1H, *J* = 9.9, 6.2, 4.1 Hz, C^4Ha), 2.58–2.65 (m, 2H, C^4Hb, C^{12}Ha), 2.22 (ddd, 1H, *J* = 13.0, 7.6, 6.6 Hz, C^{12}Hb), 1.83–1.91 (m, 2H, C^3H_2), 1.76 (dtd, 1H, *J* = 12.2, 6.5, 4.2 Hz, C^2Ha), 1.20–1.29 (m, 1H, C^2Hb).

^{13}C NMR ($CDCl_3$, 126 MHz) δ_C: 173.70 (C^6), 173.66 (C^{14}), 153.1 (C^{15}), 136.1 (C^8), 128.7 (C^{10}, $C^{10'}$), 128.3 (C^{11}), 128.2 (C^9, $C^{9'}$), 67.7 (C^5), 66.8 (C^1), 66.7 (C^7), 62.1 (C^{16}), 55.2 (C^4), 45.3 (C^{13}), 42.8 (C^{17}), 32.6 (C^{12}), 28.3 (C^2), 27.4 (C^3).

HRMS (ES^+) exact mass calculated for $[M+H]^+$ ($C_{19}H_{23}O_5N_2^+$) requires **m/z** 359.1601, found **m/z** 359.1598.

Data for **3m2**:

^{1}H NMR ($CDCl_3$, 400 MHz) δ_H, **selected peaks** : 4.61 (ddd, 1H, *J* = 11.5, 9.6, 6.8 Hz, $C^{13}H$), 4.27 (dtd, 1H, *J* = 9.1, 6.5 Hz, $C^{16}Ha$), 3.92 (d, 1H, *J* = 9.6 Hz, $C^{5}H$), 3.77–3.84 (m, 1H, $C^{1}H$), 3.67 (ddd, 1H, *J* = 11.0, 9.3, 6.5 Hz, $C^{17}Ha$), 3.16 (dt, 1H, *J* = 10.7, 6.1 Hz, $C^{4}Ha$), 2.80 (dt, 1H, *J* = 10.5, 6.3 Hz, $C^{4}Hb$), 2.43 (dt, 1H, *J* = 11.5, 6.5 Hz, $C^{12}Ha$).

^{13}C NMR ($CDCl_3$, 126 MHz) δ_C, **selected peaks**: 71.0 (C^{5}), 65.4 (C1), 61.9 (C16), 55.5 (C^{4}), 48.0 (C^{13}), 37.2 (C^{12}).

dimethyl (1S,2R,3R,7aS)-2-phenylhexahydro-1H-pyrrolizine-1,3-dicarboxylate (3n1) and dimethyl (1R,2S,3R,7aS)-1-phenylhexahydro-1H-pyrrolizine-2,3-dicarboxylate (3n2)

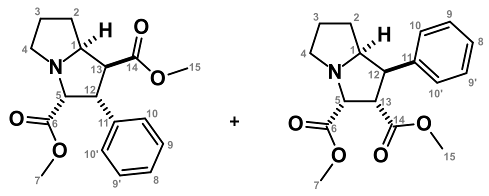

3n1 **3n2**

Prepared according to **General Procedure A** from **1m** and **2h** (2.5 equiv.) gave a 1 : 1 mixture of **3n1** and **3n2**, respectively. Purification *via* FCC (3 : 2 pentane/EtOAc) gave a 54 : 46 mixture of **3n1** and **3n2**, respectively, as a pale yellow oil (25 mg, 33%).

IR 2952, 1736 (C=O), 1437, 1197, 1172.

^{1}H NMR ($CDCl_3$, 500 MHz) δ_H: 7.30–7.34 (m, 0.92H, C9H (**B**), C9'H (**B**)), 7.18–7.29 (m, 4.08H, C^8H (**A**), C^8H (**B**), C^9H (**A**), $C^{9'}$H (**A**), C^{10}H (**A**), C^{10}H (**B**), $C^{10'}$H (**A**), $C^{10'}$H (**B**)), 4.19 (td, 0.54H, *J* = 9.1, 6.4 Hz, C^1H (**A**)), 3.95 (dd, 0.54H, *J* = 10.1, 8.2 Hz, C^{12}H (**A**)), 3.81–3.90 (m, 2H, C^1H (**B**), C^5H (**A**), C^5H (**B**), C^{13}H (**A**)), 3.79 (s, 1.38H, C^7H_3 (**B**)), 3.66 (s, 1.62H, C^7H_3 (**B**)), 3.57–3.63 (m, 0.46H, C^{13}H (**B**)), 3.57 (s, 1.38H, $C^{15}H_3$ (**B**)), 3.33-3.37 (m, 0.54H, C^4Ha (**A**)), 3.33 (s, 1.62H, $C^{15}H_3$ (**A**)), 3.14–3.21 (m, 0.46H, C^4Ha (**B**)), 3.10 (dd, 0.46H, *J* = 11.7, 9.7 Hz, C^{12}H (**B**)), 2.83-2.89 (m, 0.46H, C^4Hb (**B**)), 2.69 (td, 0.54H, *J* = 9.5, 6.1 Hz, C^4Hb (**A**)), 1.91–2.00 (m, 1H, C^3Ha (**A**), C^3Ha (**B**)), 1.81–1.92 (m, 2H, C^2Ha (**A**), C^2Ha (**B**), C^3Hb (**A**), C^3Hb (**B**)), 1.64–1.72 (m, 0.46H, C^2Hb (**B**)), 1.41–1.52 (m, 0.54H, C^2Hb (**A**)).

^{13}C NMR ($CDCl_3$, 126 MHz) δ_C: 173.1 (C^6 (**A**)), 172.9 (C^6 (**B**), C^{14} (**A**)), 172.5 (C^{14} (**B**)), 138.6 (C^{11} (**B**)), 137.3 (C^{11} (**A**)), 128.9 (C^9 (**B**), $C^{9'}$ (**B**)), 128.6 (C^9 (**A**), $C^{9'}$ (**A**)), 128.0 (C^{10} (**A**), $C^{10'}$ (**A**)), 127.7 (C^{10} (**B**), $C^{10'}$ (**B**)), 127.5 (C^8 (**B**)), 127.3 (C^8 (**A**)), 73.5 (C^5 (**A**)), 72.8 (C^1 (**B**)), 71.3 (C^5 (**B**)), 66.3 (C^1 (**A**)), 56.5 (C^{13} (**B**)), 56.2 (C^4 (**A**)), 56.0 (C^{12} (**B**)), 55.7 (C^4 (**B**)), 52.7 (C^7 (**B**)), 52.3 (C^{15} (**B**)), 52.0 (C^7 (**A**)), 51.5 (C^{15} (**A**)), 50.5 (C^{13} (**A**)), 48.6 (C^{12} (**A**)), 30.5 (C^2 (**B**)), 29.0 (C^2 (**A**)), 26.6 (C^3 (**A**)), 25.5 (C^3 (**B**)).

HRMS (ES^+) exact mass calculated for $[M+H]^+$ ($C_{17}H_{22}O_4N^+$) requires **m/z** 304.1543, found **m/z** 304.1542.

methyl (2S,4R,5R)-1-methyl-4-(2-oxooxazolidine-3-carbonyl)-5-((E)-styryl)pyrrolidine-2-carboxylate (3o)

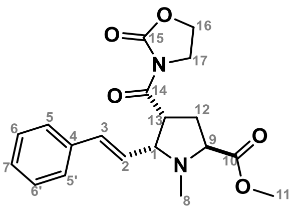

Prepared according to **General Procedure A** from **1o** and **2a**. Purification *via* FCC (3 : 2 pentane/EtOAc) gave **3o** as a pale yellow oil (66 mg, 70%).

IR 2952 (br, N–H), 1777 (C^{14}=O), 1731 (C^{10}=O), 1696 (C^{15}=O), 1450, 1387, 1361, 1261, 1200, 1171.

^{1}H NMR ($CDCl_3$, 500 MHz) δ_H: 7.31–7.35 (m, 2H, C^5H, $C^{5'}$H), 7.27–7.31 (m, 2H, C^6H, $C^{6'}$H), 7.21–7.25 (m, 1H, C^7H), 6.56 (d, 1H, *J* = 15.8 Hz, C^3H), 5.87 (dd, 1H, *J* = 15.8, 9.5 Hz, C^2H), 4.71 (app q, 1H, *J* = 9.1 Hz, C^{13}H), 4.18–4.25 (m, 1H, C^{16}Ha), 4.11 (t, 1H, *J* = 9.5 Hz, C^1H), 3.89–3.95 (m, 2H, C^{16}Hb, C^{17}Ha), 3.87 (dd, 1H, *J* = 8.3, 2.2 Hz, C^9H), 3.73–3.79 (m, 1H, C^{17}Hb), 3.74 (s, 3H, $C^{11}H_3$), 2.91 (ddd, 1H, *J* = 13.1, 9.2, 8.3 Hz, C^{12}Ha), 2.35 (s, 3H, C^8H_3), 2.02 (ddd, 1H, *J* = 13.1, 8.6, 2.2 Hz, C^{12}Hb).

^{13}C NMR ($CDCl_3$, 101 MHz) δ_C: 173.8 (C^{10}), 172.5 (C^{14}), 153.2 (C^{15}), 136.5 (C^4), 133.8 (C^3), 128.8 (C^6, $C^{6'}$), 128.0 (C^7), 127.8 (C^2), 126.5 (C^5, $C^{5'}$), 67.2 (C^1), 65.5 (C^9), 62.1 (C^{16}), 51.6 (C^{11}), 46.4 (C^{13}), 42.9 (C^{17}), 35.7 (C^8), 30.1 (C^{12}).

HRMS (ES^+) exact mass calculated for $[M+H]^+$ ($C_{19}H_{23}O_5N_2^+$) requires **m/z** 359.1602, found **m/z** 359.1601.

3-(1-methyl-2-phenylpyrrolidine-3-carbonyl)oxazolidin-2-one (5a)

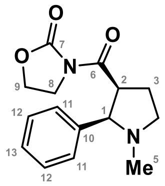

Prepared according to **General Procedure B**. Purification *via* FCC (4 : 1 pentane/EtOAc) gave **5a** as a white solid (65.6 mg, 85%). Crude dr = 7.7 : 1.

mp 118-120 °C.

IR 1769 (C^6=O), 1693 (C^7=O), 1382, 1251, 1220, 1196, 1109, 753.

^{1}H NMR ($CDCl_3$, 400 MHz) δ_H: 7.36–7.20 (m, 5H, ArH), 4.68 (ddd, *J* = 10.0, 8.8, 7.9 Hz, 1H, C^2H), 4.04 (ddd, *J* = 9.1, 8.2, 5.2 Hz, 1H, C^9H_a), 3.70-3.49 (m, 3H, C^1H, C^9H_b and C^8H), 3.30 (ddd, *J* = 8.7, 7.4, 0.9 Hz, 1H, C^4H_a), 3.01 (ddd, *J* = 10.2, 8.8, 5.2 Hz, 1H, C^8H_b), 2.65 (ddt, *J* = 12.4, 11.3, 7.7 Hz, 1H, C^3H_a), 2.37 (ddd, *J* = 11.2, 8.8, 6.5 Hz, 1H, C^4H_b), 2.17 (s, 3H, C^5H_3), 1.91 (dddd, *J* = 12.3, 8.9, 6.6, 1.1 Hz, 1H, C^3H_b).

^{13}C NMR ($CDCl_3$, 101 MHz) δ_C: 173.0 (C^6), 153.0 (C^7), 139.4 (C^{10}), 128.6 (ArC), 127.9 (ArC), 127.6 (ArC), 73.2 (C^1), 61.7 (C^9), 56.1 (C^4), 47.9 (C^2), 42.6 (C^8), 40.2 (C^5), 25.4 (C^3).

HRMS (ES^+) exact mass calculated for $[M+H]^+$ ($C_{15}H_{19}N_2O_3^+$) requires **m/z** 275.1390, found **m/z** 275.1390.

3-(1-benzyl-2-phenylpyrrolidine-3-carbonyl)oxazolidin-2-one (5b)

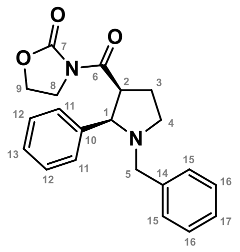

Prepared according to **General Procedure B**. Purification *via* FCC (4 : 1 pentane/EtOAc) gave **5b** as a clear oil (71.8 mg, 82%). Crude dr = >20 : 1.

IR 1769 (C^6=O), 1694 (C^7=O), 1384, 1220, 1038, 700.

^{1}H NMR ($CDCl_3$, 400 MHz) δ_H: 7.48–7.38 (m, 2H, ArH), 7.35–7.17 (m, 8H, ArH), 4.66 (dt, J = 10.3, 8.6 Hz, 1H, C^2H), 4.09–4.01 (m, 1H, C^9H_a), 3.93 (d, J = 10.2 Hz, 1H, C^1H), 3.79 (d, J = 13.3 Hz, 1H, C^5H_a), 3.70-3.55 (m, 2H, C^9H_b, C^8H_a), 3.17 (t, J = 8.2 Hz, 1H, C^4H_a), 3.11 (d, J = 13.3 Hz, 1H, C^5H_b), 3.04–2.94 (m, 1H, C^8H_b), 2.61 (tt, J = 11.8, 7.9 Hz, 1H, C^3H_a), 2.27 (ddd, J = 11.2, 8.7, 6.3 Hz, 1H, C^4H_b), 1.93–1.78 (m, 1H, C^3H_b).

^{13}C NMR ($CDCl_3$, 101 MHz) δ_C: 153.1 (C^6), 128.9 (ArC), 128.6 (ArC), 128.1 (ArC), 127.9 (ArC), 127.7 (ArC), 126.8 (ArC), 70.7 (C^1), 61.7 (C^9), 57.3 (C^5), 52.3 (C^4), 47.6 (C^2), 42.6 (C^8), 25.3 (C^3).

HRMS (ES^+) exact mass calculated for $[M+H]^+$ ($C_{21}H_{23}N_2O_3^+$) requires **m/z** 351.1703, found **m/z** 351.1700.

3-(2-(4-methoxyphenyl)-1-methylpyrrolidine-3-carbonyl)oxazolidin-2-one (5c)

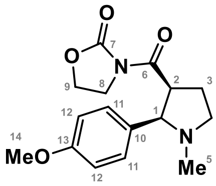

Prepared according to **General Procedure B**. Purification *via* FCC (4 : 1 pentane/EtOAc) gave **5c** as a white solid (65.6 mg, 86%). Crude dr = 6.3 : 1.

mp 80–85 °C.

IR 1759 (C^6=O), 1694 (C^7=O), 1387, 1240, 1219, 1023, 831.

^{1}H NMR ($CDCl_3$, 400 MHz) δ_H: 7.22–7.16 (m, 2H, $C^{11}H$), 6.83-6.77 (m, 2H, $C^{12}H$), 4.61 (dt, *J* = 9.9, 8.3 Hz, 1H, C^2H), 4.11–4.01 (m, 1H, C^9H_a), 3.76 (s, 3H, $C^{14}H_3$), 3.70-3.57 (m, 2H, C^8H_a, C^9H_b), 3.52 (d, *J* = 10.0 Hz, 1H, C^1H), 3.26 (t, *J* = 8.2 Hz, 1H, C^4H_a), 3.14-3.04 (m, 1H, C^8H_b), 2.61 (ddt, *J* = 12.3, 11.3, 7.7 Hz, 1H, C^3H_a), 2.32 (ddd, *J* = 11.4, 8.8, 6.5 Hz, 1H, C^4H_b), 2.13 (s, 3H, C^5H_3), 1.86 (dddd, *J* = 12.4, 8.8, 6.5, 1.0 Hz, 1H, C^3H_b).

^{13}C NMR ($CDCl_3$, 101 MHz) δ_C: 173.1 (C^6), 159.1 (C^{13}), 153.0 (C^7), 131.3 (C^{10}), 129.6 (C^{11}), 113.2 (C^{12}), 72.5 (C^1), 61.7 (C^9), 56.0 (C^4), 55.3 (C^{14}), 47.7 (C^2), 42.7 (C^8), 40.2 (C^5), 25.4 (C^3).

HRMS (ES^+) exact mass calculated for $[M+H]^+$ ($C_{16}H_{21}N_2O_4^+$) requires **m/z** 305.1496, found **m/z** 305.1494.

3-(2-(3,5-bis(trifluoromethyl)phenyl)-1-methylpyrrolidine-3-carbonyl)oxazolidin-2-one (5d)

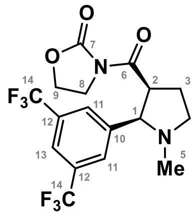

Prepared according to **General Procedure B**. Purification *via* FCC (4 : 1 pentane/EtOAc) gave **5d** as a white solid (65.3 mg, 64%). Crude dr = 11.1 : 1.

mp 40–44 °C.

IR 1768 (C^6=O), 1686 (C^7=O), 1390, 1256, 1123, 1007.

^{1}H NMR ($CDCl_3$, 400 MHz) δ_H: 7.80 (s, 2H, $C^{11}H$), 7.76 (s, 1H, $C^{13}H$), 4.67 (dt, *J* = 10.1, 8.4 Hz, 1H, C^2H), 4.14 (td, *J* = 8.7, 6.3 Hz, 1H, C^9H_a), 3.80 (d, *J* = 9.9 Hz, 1H, C^1H), 3.78-3.65 (m, 2H, C^9H_b, C^8H_a), 3.33 (t, *J* = 8.1 Hz, 1H, C^4H_a), 3.02 (ddd, *J* = 10.9, 8.8, 6.3 Hz, 1H, C^8H_b), 2.63 (dddd, *J* = 12.2, 11.2, 8.3, 7.4 Hz, 1H, C^3H_a), 2.46 (ddd, *J* = 11.2, 8.8, 6.3 Hz, 1H, C^4H_b), 2.19 (s, 3H, C^5H_3), 1.93 (dddd, *J* = 12.3, 8.6, 6.3, 1.1 Hz, 1H, C^3H_b).

^{13}C NMR ($CDCl_3$, 101 MHz) δ_C: 171.9 (C^6), 152.9 (C^7), 143.0 (C^{10}), 131.2 (q, *J* = 33.4 Hz, C^{12}), 128.9 (d, *J* = 3.8 Hz, C^{11}), 123.2 (q, *J* = 272.8 Hz, C^{14}), 121.6-121.2 (m, C^{13}), 71.8 (C^1), 61.7 (C^9), 55.8 (C^4), 48.3 (C^2), 42.3 (C^8), 40.1 (C^5), 26.0 (C^3).

^{19}F NMR ($CDCl_3$, 377 MHz) δ_F: −62.8.

HRMS (ES$^+$) exact mass calculated for $[M+H]^+$ ($C_{17}H_{17}F_6N_2O_3^+$) requires **m/z** 411.1138, found **m/z** 411.1135.

3-(1-benzyl-2-(3,4,5-trifluorophenyl)pyrrolidine-3-carbonyl)oxazolidin-2-one (5e)

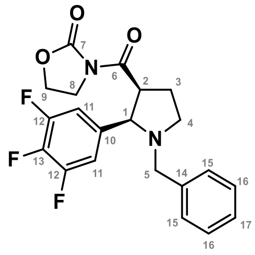

Prepared according to **General Procedure B**. Purification *via* FCC (4 : 1 pentane/EtOAc) gave **5e** as a pale yellow oil (62.8 mg, 62%). Crude dr = >20 : 1.

IR 1776 (C^6=O), 1698 (C^7=O), 1387, 1260, 1223.

^{1}H NMR ($CDCl_3$, 400 MHz) δ_H: 7.33–7.24 (m, 2H, ArH), 7.25–7.19 (m, 3H, ArH), 7.09 (dd, *J* = 8.8, 6.7 Hz, 2H, C^{11}H), 4.56 (ddd, *J* = 10.4, 9.5, 8.2 Hz, 1H, C^1H), 4.24 (td, *J* = 9.1, 6.7 Hz, 1H, C^9H_a), 4.04 (td, *J* = 9.1, 6.9 Hz, 1H, C^9H_b), 3.96 (d, *J* = 10.4 Hz, 1H, C^2H), 3.81 (ddd, *J* = 11.1, 9.3, 6.9 Hz, 1H, C^8H_a), 3.70 (d, *J* = 13.1 Hz, 1H, C^5H_a), 3.35–3.11 (m, 3H, C^8H_b, C^5H_b, C^4H_a), 2.64-2.47 (m, 1H, C^3H), 2.41–2.30 (m, 1H, C^4H_b), 1.92–1.79 (m, 1H, C^3H_b).

^{13}C NMR ($CDCl_3$, 101 MHz) δ_C: 171.8 (C^6), 153.2 (C^7), 150.6 (ddd, *J* = 249.7, 9.8, 3.7 Hz, C^{12}), 138.3 (C^{10}), 137.1 (bs, C^{13}), 128.6 (C^{15}), 128.3 (C^{16}), 127.1 (C^{17}), 112.6 (d, *J* = 20.7 Hz, C^{11}), 68.9 (C^2), 61.9 (C^9), 57.6 (C^5), 52.2 (C^4), 47.9 (C^1), 42.5 (C^8), 26.0 (C^3).

^{19}F NMR ($CDCl_3$, 377 MHz) δ_F: -134.2 (dd, *J* = 20.7, 9.2 Hz), –161.0 (dt, *J* = 20.8, 6.9 Hz).

HRMS (ES^+) exact mass calculated for $[M+H]^+$ ($C_{21}H_{20}F_3N_2O_3^+$) requires **m/z** 405.1426, found **m/z** 405.1426.

3-(1-methyl-2-(m-tolyl)pyrrolidine-3-carbonyl)oxazolidin-2-one (5f)

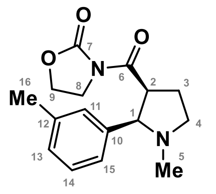

Prepared according to **General Procedure B**. Purification *via* FCC (4 : 1 pentane/EtOAc) gave **5f** as a pale yellow oil (62.4 mg, 87%). Crude dr = 4.9 : 1.

IR 1780 (C^6=O), 1699 (C^7=O), 1388, 1259.

^{1}H NMR ($CDCl_3$, 400 MHz) δ_H: 7.15 (t, J = 7.4 Hz, 1H, $C^{14}H$), 7.11-7.00 (m, 3H, $C^{11}H$, $C^{13}H$, $C^{15}H$), 4.67 (ddd, J = 10.0, 8.8, 7.8 Hz, 1H, C^2H), 4.03 (ddd, J = 9.3, 8.3, 5.0 Hz, 1H, C^9H_a), 3.62 (dt, J = 10.4, 8.7 Hz, 1H, C^8H_a), 3.56-3.44 (m, 2H, C^1H, C^9H_b), 3.29 (ddd, J = 8.7, 7.5, 1.0 Hz, 1H, C^4H_a), 2.98 (ddd, J = 10.4, 8.9, 5.0 Hz, 1H, C^8H_b), 2.63 (ddt, J = 12.4, 11.3, 7.7 Hz, 1H, C^3H_a), 2.34 (ddd, J = 11.2, 8.8, 6.6 Hz, 1H, C^4H_b), 2.30 (s, 3H, $C^{16}H_3$), 2.16 (s, 3H, C^5H_3), 1.89 (dddd, J = 12.3, 8.8, 6.6, 1.0 Hz, 1H, C^3H_b).

^{13}C NMR ($CDCl_3$, 101 MHz) δ_C: 173.0 (C^6), 153.0 (C^7), 139.3 (C^{10}), 137.5 (C^{12}), 129.1 (ArC), 128.2 (ArC), 127.8 (C^{14}), 125.7 (ArC), 73.2 (C^1), 61.7 (C^9), 56.1 (C^4), 47.9 (C^2), 42.7 (C^8), 40.3 (C^5), 25.3 (C^3), 21.2 (C^{16}).

HRMS (ES^+) exact mass calculated for $[M+H]^+$ ($C_{16}H_{21}N_2O_3^+$) requires **m/z** 289.1547, found **m/z** 289.1547.

3-(2-(5-bromo-2-methylphenyl)-1-methylpyrrolidine-3-carbonyl)oxazolidin-2-one (5g)

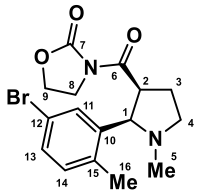

Prepared according to **General Procedure B**. Purification *via* FCC (4 : 1 pentane/EtOAc) gave **5g** as a pale yellow oil (48.0 mg, 52%). Crude dr = >20 : 1.

IR 1774 (C^6=O), 1697 (C^7=O), 1386, 1258, 1222, 1039

^{1}H NMR ($CDCl_3$, 400 MHz) δ_H: 7.45 (d, J = 2.2 Hz, 1H, $C^{11}H$), 7.25 (dd, J = 8.0, 2.4 Hz, 1H, $C^{13}H$), 7.00 (d, J = 8.1 Hz, 1H, ^{C14}H), 4.72 (td, J = 8.8, 5.1 Hz, 1H, C^9H_a), 4.10 (m, 1H, C^8H_a), 3.79-3.68 (m, 2H, C1H, C^9H_b), 3.62 (q, J = 8.7 Hz, 1H, C^8H_b), 3.43-3.33 (m, 2H, C^4H_a), 2.59 (dddd, J = 12.7, 9.6, 8.3, 5.2 Hz, 1H, C^3H_a), 2.39-2.29 (m, 4H, $C^{16}H_3$, C^4H_b), 2.11 (s, 3H, C^5H_3), 1.98 (dtd, J = 12.7, 8.6, 2.0 Hz, 1H, C^3H_b).

^{13}C NMR ($CDCl_3$, 101 MHz) δ_C: 172.3 (C^9), 152.0 (C^8), 138.2 (C^{10}), 135.7 (C^{15}), 131.1 (C^{11}), 129.4 (C^{14}), 128.9 (C^{13}), 118.3 (C^{12}), 68.3 (C^1), 60.9 (C^9), 54.7 (C^4), 45.5 (C^2), 41.8 (C^8), 39.1 (C^5), 24.0 (C^3), 17.9 (C^{16}).

HRMS (ES^+) exact mass calculated for $[M+H]^+$ ($C_{16}H_{20}BrN_2O_3^+$) requires **m/z** 367.0652, 369.0632, found **m/z** 367.0653, 369.0633.

3-(1-benzyl-2-(4-chlorophenyl)pyrrolidine-3-carbonyl)oxazolidin-2-one (5h)

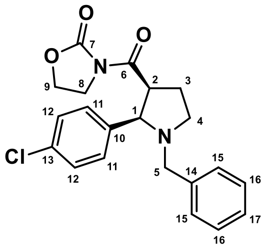

Prepared according to **General Procedure B**. Purification *via* FCC (4 : 1 pentane/EtOAc) gave **5h** as a pale yellow oil (63.1 mg, 73%). Crude dr = >20 : 1.

IR 1774 (C^6=O), 1697 (C^7=O), 1386, 1258, 1222, 1039

^{1}H NMR ($CDCl_3$, 400 MHz) δ_H: 7.43–7.35 (m, 2H, C^{12}H), 7.31-7.25 (m, 4H, ArH), 7.23 (d, J = 7.3 Hz, 3H, ArH), 4.63 (dt, J = 10.3, 8.6 Hz, 1H, C^2H), 4.14 (td, J = 8.3, 7.7, 5.8 Hz, 1H, C^9H_a), 3.94 (d, J = 10.3 Hz, 1H, C^1H), 3.82–3.66 (m, 3H, C^9H_b, C^8H_a, C^5H_a), 3.21-3.07 (m, 3H, C^8H_b, C^5H_b, C^4H_a), 2.60 (dddd, J = 12.3, 11.3, 8.6, 7.4 Hz, 1H, C^3H_a), 2.30 (ddd, J = 11.3, 8.8, 6.2 Hz, 1H, C^4H_b), 1.87 (dddd, J = 12.3, 8.6, 6.3, 1.1 Hz, 1H, C^3H_b).

^{13}C NMR ($CDCl_3$, 101 MHz) δ_C: 172.5 (C^6), 153.1 (C^7), 138.7 (C^{10} or C^{14}), 138.6 (C^{10} or C^{14}), 133.3 (C^{13}), 130.3 (C^{12}), 128.6 (ArC), 128.1 (ArC), 128.0 (ArC), 126.9 (ArC), 69.8 (C^1), 61.8 (C^9), 57.4 (C^5), 52.3 (C^4), 47.7 (C^2), 42.6 (C^8), 25.5 (C^3).

HRMS (ES^+) exact mass calculated for $[M+H]^+$ ($C_{21}H_{22}ClN_2O_3^+$) requires **m/z** 385.1313, found **m/z** 385.1313.

3-(1-benzyl-2-(4-nitrophenyl)pyrrolidine-3-carbonyl)oxazolidin-2-one (5i)

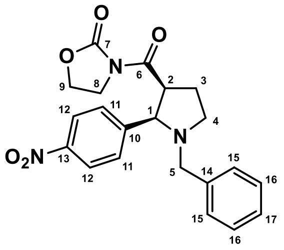

Prepared according to **General Procedure B**. Purification *via* FCC (4 : 1 pentane/EtOAc) gave **5i** as a pale yellow oil (57.3 mg, 58%). Crude dr = >20 : 1.

IR 1780 (C^6=O), 1692 (C^7=O), 1530, 1210

^{1}H NMR ($CDCl_3$, 400 MHz) δ_H: 8.18–8.10 (m, 2H, $C^{12}H$), 7.64-7.57 (m, 2H, $C^{11}H$), 7.30–7.14 (m, 5H, $C^{15}H$, $C^{16}H$, $C^{17}H$), 4.64 (ddd, J = 10.4, 9.2, 8.3 Hz, 1H, C^2H), 4.15 (td, J = 9.1, 6.6 Hz, 1H, C^9H_a), 4.12 (d, J = 10.4 Hz, 1H, C^1H) 3.85 (td, J = 9.0, 6.9 Hz, 1H, C^9H_b), 3.73 (ddd, J = 11.3, 9.4, 7.0 Hz, 1H, C^8H_a), 3.67 (d, J = 13.3. Hz, 1H, C^5Ha), 3.25 (d, J = 13.2 Hz, 1H, C^5H_b), 3.21 (d, J = 8.0 Hz, 1H, C^8H_b), 3.08 (ddd, J = 11.0, 9.2, 6.7 Hz, 1H, C^4H_a), 2.69–2.57 (m, 1H, C^3H_a), 2.39 (ddd, J = 11.6, 8.9, 6.0 Hz, 1H, C^4H_b), 1.91 (dddd, J = 12.3, 8.3, 6.0, 1.0 Hz, 1H, C^3H_b).

^{13}C NMR ($CDCl_3$, 101 MHz) δ_C: 171.8 (C^8), 153.0 (C^7), 148.2 (C^{10}), 147.5 (C^{13}), 138.2 (C^{14}), 129.9 (C^{11}), 128.6 (ArC), 128.2 (ArC), 127.1 (ArC), 123.0 (C^{12}), 69.6 (C^1), 61.8 (C^9), 57.8 (C^5), 52.56 (C^4), 48.2 (C^2), 42.4 (C^8), 26.2 (C^3).

HRMS (ES^+) exact mass calculated for $[M+H]^+$ ($C_{21}H_{22}N_3O_5^+$) requires **m/z** 396.1554, found **m/z** 396.1555.

3-(1-benzyl-2-(3-nitrophenyl)pyrrolidine-3-carbonyl)oxazolidin-2-one (5j)

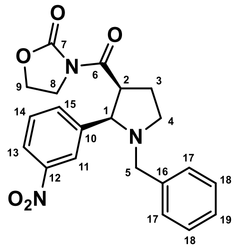

Prepared according to **General Procedure B**. Purification *via* FCC (4 : 1 pentane/EtOAc) gave **5j** as a brown oil (53.1 mg, 54%). Crude dr = >20 : 1.

IR 1774 (C^6=O), 1696 (C^7=O), 1529, 1338, 1351, 1224.

^{1}H NMR ($CDCl_3$, 400 MHz) δ_H: 8.27 (t, J = 2.0 Hz, 1H, $C^{11}H$), 8.08 (ddd, *J* = 8.1, 2.3, 1.1 Hz, 1H, $C^{13}H$), 7.76 (dt, *J* = 7.7, 1.4 Hz, 1H, $C^{15}H$), 7.45 (t, *J* = 7.9 Hz, 1H, $C^{14}H$), 7.29-7.16 (m, 5H, ArH), 4.63 (ddd, *J* = 10.3, 9.3, 8.2 Hz, 1H, C^2H), 4.20-4.12 (m, 2H, C1H, C^9H_a), 3.85 (td, *J* = 9.0, 7.0 Hz, 1H, C^9H_b), 3.76-3.67 (m, 2H, C^5H_a, C^8H_a), 3.30 (d, *J* = 13.2 Hz, 1H, C^5H_b), 3.23 (dd, *J* = 8.8, 7.1 Hz, 1H, C^4H_a), 3.07 (ddd, *J* = 11.0, 9.2, 6.6 Hz, 1H, C^8H_b), 2.63 (tdd, *J* = 12.0, 9.3, 7.2 Hz, 1H, C^3H_a), 2.41 (ddd, *J* = 11.5, 8.8, 5.9 Hz, 1H, C^4H_b), 1.91 (dddd, *J* = 12.4, 8.2, 5.9, 1.0 Hz, 1H, C^3H_b).

^{13}C NMR ($CDCl_3$, 101 MHz) δ_C: 161.8 (C^6), 154.2 (C^7), 148.0 (C^{12}), 143.0 (C^{10}), 135.0 (C^{15}), 131.7 (ArC), 128.7 (C^{14}), 128.2 (ArC), 127.0 (ArC), 123.7 (C^{11}), 122.6 (C^{13}), 69.4 (C^1), 61.8 (C^9), 57.9 (C^5), 52.5 (C^4), 48.1 (C^2), 42.4 (C^8), 26.1 (C^3).

HRMS (ES^+) exact mass calculated for $[M+H]^+$ ($C_{21}H_{22}N_3O_5^+$) requires **m/z** 396.1554, found **m/z** 396.1555.

4-(1-benzyl-3-(2-oxooxazolidine-3-carbonyl)pyrrolidin-2-yl)benzonitrile (5k)

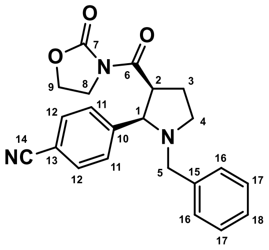

Prepared according to **General Procedure B**. Purification *via* FCC (4 : 1 pentane/EtOAc) gave **5k** as a pale yellow oil (56.4 mg, 60%). Crude dr = >20 : 1.

IR 1776 (C^6=O), 1698 (C^7=O), 1387, 1260, 1223.

^{1}H NMR ($CDCl_3$, 400 MHz) δ_H: 7.62–7.52 (m, 4H, $C^{11}H$, $C^{12}H$), 7.30–7.16 (m, 5H, $C^{16}H$, $C^{17}H$, $C^{18}H$), 4.63 (dt, *J* = 10.4, 8.7 Hz, 1H, C^2H), 4.20–4.09 (m, 1H, C^9H_a), 4.05 (d, *J* = 10.4 Hz, 1H, C^1H), 3.84 (td, *J* = 9.0, 7.1 Hz, 1H, C^9H_b), 3.77–3.64 (m, 2H, C^8H_a, C^5H_a), 3.26-3.17 (m, 2H, C^5H_b, C^4H_a), 3.06 (ddd, *J* = 10.9, 9.2, 6.5 Hz, 1H, C^8H_b), 2.62 (tdd, *J* = 11.9, 9.0, 7.2 Hz, 1H, C^3H_a), 2.36 (ddd, *J* = 11.5, 8.9, 6.0 Hz, 1H, C^4H_b), 1.95–1.84 (m, 1H C^3H_b).

^{13}C NMR ($CDCl_3$, 101 MHz) δ_C: 131.6 (ArC), 129.8 (ArC), 128.6 (ArC), 128.2 (ArC), 69.9 (C^1), 61.8 (C^9), 57.7 (C^5), 52.5 (C^4), 48.1 (C^2), 42.4 (C^8), 26.0 (C^3).

HRMS (ES^+) exact mass calculated for $[M+H]^+$ ($C_{22}H_{22}N_3O_5^+$) requires **m/z** 376.1656, found **m/z** 376.1654.

3-(1-methyl-2-(thiophen-2-yl)pyrrolidine-3-carbonyl)oxazolidin-2-one (5l)

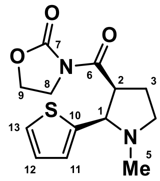

Prepared according to **General Procedure B**. Purification *via* FCC (4 : 1 pentane/EtOAc) gave **5l** as a white solid (44.2 mg, 63%). Crude dr = 7.1:1.

mp 95–100 °C.

IR 1770 (C^6=O), 1697 (C^7=O), 1386, 1220, 1038, 701.

^{1}H NMR ($CDCl_3$, 400 MHz) δ_H: 7.20 (ddd, *J* = 5.1, 1.3, 0.6 Hz, 1H, $C^{13}H$), 6.96 (dt, *J* = 3.4, 0.8 Hz, 1H, $C^{12}H$), 6.91 (dd, *J* = 5.0, 3.5 Hz, 1H, $C^{11}H$), 4.61 (dt, *J* = 10.1, 8.6 Hz, 1H, C^2H), 4.16 (ddd, *J* = 9.1, 8.4, 5.6 Hz, 1H, C^9H_a), 4.01 (d, *J* = 10.1 Hz, 1H, C^1H), 3.86 (dt, *J* = 8.9, 8.1 Hz, 1H, C^9H_b), 3.75 (ddd, *J* = 10.8, 9.1, 7.9 Hz, 1H, C^8H_a), 3.34–3.22 (m, 2H, C^8H_b, C^4H_a), 2.66 (dddd, *J* = 12.3, 11.3, 8.5, 7.4 Hz, 1H, C^3H_a), 2.37 (ddd, *J* = 11.3, 9.0, 6.3 Hz, 1H, C^4H_b), 2.27 (s, 3H, C^5H_3), 1.88 (dddd, *J* = 12.4, 8.6, 6.3, 1.1 Hz, 1H, C^3H_b).

^{13}C NMR ($CDCl_3$, 101 MHz) δ_C: 172.4 (C^6), 153.3 (C^7), 144.4 (C^{10}), 126.2 (C^{11}), 125.9 (C^{12}), 125.2 (C^{13}), 68.0 (C^1), 61.9 (C^9), 55.7 (C^4), 48.1 (C^2), 42.9 (C^8), 40.5 (C^5), 25.3 (C^3).

HRMS (ES^+) exact mass calculated for $[M+H]^+$ ($C_{13}H_{17}N_2O_3S^+$) requires **m/z** 281.0954, found **m/z** 281.0951.

3-(1-benzyl-2-(furan-2-yl)pyrrolidine-3-carbonyl)oxazolidin-2-one (5m)

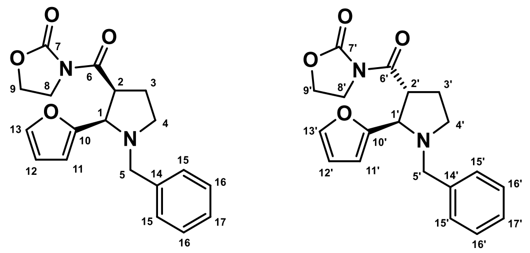

Prepared according to **General Procedure B**. Purification *via* FCC (4 : 1 pentane/EtOAc) gave **5m** as the inseparable mixtures of title compounds as a pale yellow oil (67.6 mg, 79%). Crude dr = 3.1 : 1.

IR 1777 (C^6=O), 1699 (C^7=O), 1387, 1224.

^{1}H NMR ($CDCl_3$, 400 MHz) (for major isomer) δ_H: 7.43–7.18 (m, 6H, C^{15}H, C^{16}H, C^{17}H, C^{13}H), 6.36–6.27 (m, 2H, C^{11}H, C^{12}H), 4.55–4.44 (m, 1H, C^2H), 4.42-4.32 (m, 1H, C^9H_a), 4.29–4.19 (m, 1H, C^1H), 4.09–3.97 (m, 2H, C^9H_b, C^8H_a), 3.88–3.79 (m, 2H, C^5H_a), 3.47 (ddd, *J* = 10.8, 9.2, 5.6 Hz, 1H, C^8H_b), 3.35 (d, *J* = 13.4 Hz, 1H, C^5H_b), 3.11 (ddd, *J* = 8.9, 7.4, 1.6 Hz, 1H, C^4H_a), 2.63 (dddd, *J* = 12.3, 10.7, 9.1, 7.3 Hz, 1H, C^3H_b), 2.45–2.33 (m, 1H, C^4H_b), 1.87 (dddd, *J* = 12.1, 8.0, 6.0, 1.5 Hz, 1H, C^3H_b).

^{13}C NMR ($CDCl_3$, 101 MHz) (for major isomer) δ_C: 172.0 (C^6), 154.0 (C^7), 142.0 (C^{14}), 128.8 (ArC), 128.2 (ArC), 128.1 (ArC), 126.9 (ArC), 110.4 (C^{12}), 110.1 (C^{10}), 108.7 (C^{11}), 63.0 (C^1), 62.0 (C^9), 57.4 (C^6), 51.8 (C^4), 46.6 (C^2), 42.8 (C^8), 25.2 (C^3).

HRMS (ES^+) exact mass calculated for $[M+H]^+$ ($C_{19}H_{21}N_2O_4^+$) requires **m/z** 341.1496, found **m/z** 341.1496.

tert-butyl 1-methyl-2-phenylpyrrolidine-3-carboxylate (5n)

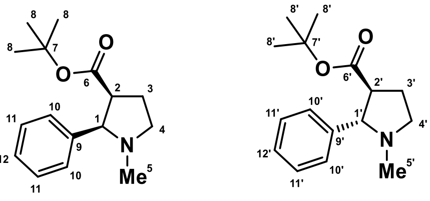

Prepared according to **General Procedure B**. Purification *via* FCC (4 : 1 pentane/EtOAc) gave **5n** as the inseparable mixtures of title compounds as a pale yellow oil (50.9 mg, 78%). Crude dr = 5.4 : 1.

IR 1728 (C^{6}=O, $C^{6'}$=O), 1367, 1151.

^{1}H NMR ($CDCl_3$, 400 MHz) δ_H: 7.33–7.13 (m, 6.8H, ArH), 3.45–3.38 (m, 1H, C^{1}H (**A**)), 3.25–3.00 (m, 2.5H), 2.78 (ddd, *J* = 10.8, 8.7, 5.5 Hz, 1H, C^{2}H (**B**)), 2.43–2.23 (m, 2H), 2.21-2.07 (m, 6H), 2.06–1.81 (m, 2H), 1.29 (s, 9H, $C^{8}H_3$ (**B**)), 0.90 (s, 2.2H, $C^{8}H_3$ (**A**)).

^{13}C NMR ($CDCl_3$, 101 MHz) δ_C: 173.8 (C^{6} (**B**)), 141.3 (C^{6} (**A**)), 128.9 (ArC), 128.5 (ArC), 128.4 (ArC), 128.0 (ArC), 127.8 (ArC), 127.5 (ArC), 127.5 (ArC), 127.2 (ArC), 80.4 (C^{7} (**B**)), 79.8 (C^{7} (**A**)), 74.8 (C^{1} (**B**)), 73.0 (C^{1} (**A**)), 56.1 (C^{4} (**B**)), 56.0 (C^{4} (**A**)), 53.4 (C^{2} (**B**)), 50.3 (C^{2} (**A**)), 40.5 (C^{5} (**A**)), 40.2 (C^{5} (**B**)), 28.0 (C^{8} (**B**)), 27.4 (C^{8} (**A**)), 27.1 (C^{3} (**B**)), 27.0 (C^{3} (**A**)).

HRMS (ES^+) exact mass calculated for $[M+H]^+$ ($C_{16}H_{24}NO_2^+$) requires **m/z** 262.1802, found **m/z** 262.1802.

dimethyl 1-methyl-2-phenylpyrrolidine-3,4-dicarboxylate (5o)

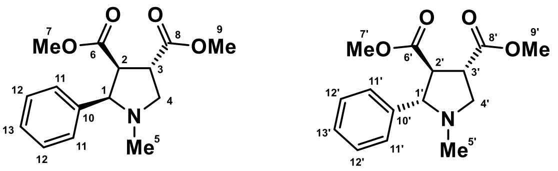

Prepared according to **General Procedure B**. Purification *via* FCC (4 : 1 pentane/EtOAc) gave **5o** as the inseparable mixtures of title compounds as a pale yellow oil (50.9 mg, 78%). Crude dr = 5.4 : 1.

IR 1732 (C^6=O, C^8=O), 1436, 1170, 1006, 701.

^{1}H NMR ($CDCl_3$, 400 MHz) δ_H: 7.39–7.16 (m, 5H, ArH), 3.80-3.71 (m, 1H), 3.74 (s, 3H, C^7H_3 (**B**)), 3.69 (s, 3H, C^7H_3 (**A**)), 3.60 (s, 3H, C^9H_3 (**B**)), 3.63-3.44 (m, 5H), 3.33 (ddd, *J* = 8.8, 5.4, 1.9 Hz, 1H, C^4H_a (**B**)), 3.25 (d, *J* = 8.8 Hz, 1H, (**B**)), 3.05 (s, 3H, C^9H_3 (**A**)), 2.66 (dd, *J* = 9.7, 8.7 Hz, 1H, C^4H_b (**B**)), 2.49 (dd, *J* = 10.4, 9.0 Hz, 1H, C^4H_b (**A**)), 2.16 (s, 3H, C^5H_3 (**A**)), 2.08 (s, 3H, C^5H_3 (**B**)).

^{13}C NMR ($CDCl_3$, 101 MHz) δ_C: 174.0 (C^8), 173.6 (C^8), 173.5 (C^6), 172.2 (C^6), 140.2 (C^{10}), 138.2 (C^{10}), 128.5 (ArC), 128.2 (ArC), 128.1 (ArC), 128.0 (ArC), 127.9 (ArC), 127.7 (ArC), 74.7 (C^1), 73.1 (C^1), 58.8 (C^2), 58.6 (C^2), 54.9, 52.8, 52.4, 52.1, 52.0, 51.4, 45.0 (C^5), 44.2 (C^5), 40.0 (C^3), 39.6 (C^3).

HRMS (ES^+) exact mass calculated for $[M+H]^+$ ($C_{15}H_{20}NO_4^+$) requires **m/z** 278.1387, found **m/z** 278.1388.

5-methyl-2,4-diphenyltetrahydropyrrolo[3,4-c]pyrrole-1,3(2H,3aH)-dione (5p)

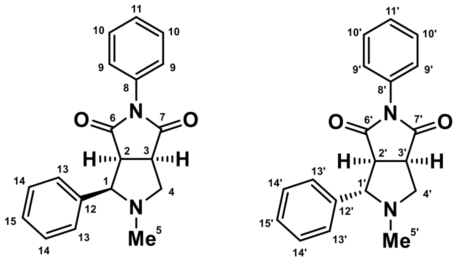

Prepared according to **General Procedure B**. Purification *via* FCC (4 : 1 pentane/EtOAc) gave **5p** as the inseparable mixtures of title compounds as a pale yellow oil (78.1 mg, 98%). Crude dr = 1.2 : 1.

IR 1712 (C^6=O, C^7=O), 1499, 1382, 1184.

^{1}H NMR ($CDCl_3$, 400 MHz) δ_H: 7.51–7.20 (m, 28H, ArH), 3.72 (d, *J* = 9.5 Hz, 1H, C^4H_a (**A**)), 3.68–3.54 (m, 5.9H, C^4H_a (**B**), C^3H (**B**), C^1H (**A**, **B**)), 3.49 (dd, *J* = 8.9, 7.9 Hz, 1H, C^2H (**A**)), 3.42 (dd, *J* = 8.7, 6.1 Hz, 1.6H, C^2H (**B**)), 3.35 (ddd, *J* = 7.7, 6.8, 0.6 Hz, 1H, C^3H (**A**)), 2.73 (dd, *J* = 9.2, 6.1 Hz, 1.6H, C^4H_b (**B**)), 2.63 (dd, *J* = 9.5, 6.7 Hz, 1H, C^4H_b (**A**)), 2.21 (s, 3H, C^5H_3 (**A**)), 2.17 (s, 4.8H, C^5H_3 (**B**)).

^{13}C NMR ($CDCl_3$, 101 MHz) δ_C: 178.2 (C(C=O)), 177.0 (C(C=O)), 176.4 (C(C=O)), 174.8 (C(C=O)), 136.7 (ArC), 132.1 (ArC), 131.8 (ArC), 129.2 (ArC), 129.1 (ArC), 129.0 (ArC), 128.8 (ArC), 128.6 (ArC), 128.5 (ArC), 128.3 (ArC), 128.2 (ArC), 128.1 (ArC), 128.0 (ArC), 127.8 (ArC), 126.5 (ArC), 126.2 (ArC), 73.4 (C^1 (**A**)), 72.7 (C^1 (**B**)), 58.3 (C^4 (**A**)), 57.5 (C^4 (**B**)), 53.6 (C^2 (**B**)), 50.7 (C^2 (**A**)), 44.6 (C^3 (**A**)), 44.3 (C^3 (**B**)), 39.7 (C^5 (**A**)), 38.8 (C^5 (**B**)).

HRMS (ES^+) exact mass calculated for $[M+H]^+$ ($C_{19}H_{19}N_2O_2^+$) requires **m/z** 307.1441, found **m/z** 307.1441.

1-methyl-2-phenyl-3-(phenylsulfonyl)pyrrolidine (5q)

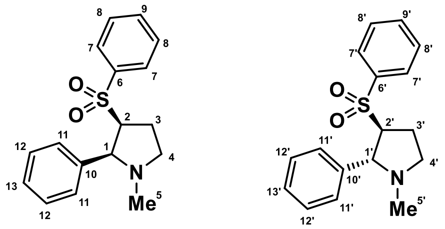

Prepared according to **General Procedure B**. Purification *via* FCC (4 : 1 pentane/EtOAc) gave **5q** the inseparable mixtures of title compounds as a pale yellow oil (43.3 mg, 57%). Crude dr = 1.6 : 1.

IR 1447, 1305, 1144, 1086.

^{1}H NMR ($CDCl_3$, 400 MHz) δ_H: 7.96–7.90 (m, 2H, ArH), 7.79–7.72 (m, 3H, ArH), 7.69–7.62 (m, 1H, ArH), 7.60–7.49 (m, 3H, ArH), 7.45–7.37 (m, 3H, ArH), 7.34–7.21 (m, 5H, ArH), 7.19–7.13 (m, 4H, ArH), 7.13–7.06 (m, 3H, ArH), 3.92–3.82 (m, 1H, C^2H), 3.67 (ddd, *J* = 10.4, 7.1, 3.3 Hz, 1H, C^2H), 3.54 (d, *J* = 7.1 Hz, 1H, C^1H), 3.35 (dt, *J* = 9.8, 7.6 Hz, 2H), 3.18 (ddt, *J* = 9.0, 7.1, 0.9 Hz, 1H), 2.82 (dd, *J* = 9.9, 8.8 Hz, 1H), 2.65 (ddd, *J* = 13.9, 7.2, 4.2 Hz, 1H), ddd, *J* = 11.1, 9.0, 6.7 Hz, 1H), 2.45–2.25 (m, 3H), 2.15 (s, 3H), 2.15 (s, 3H), 2.00 (ddd, *J* = 14.0, 10.7, 9.6 Hz, 1H).

^{13}C NMR ($CDCl_3$, 101 MHz) δ_C: 140.8 (C^{10}), 140.4 (C^{10}), 138.7 (C^6), 138.5 (C^6), 133.8 (C^9), 133.6 (C^9), 129.4 (ArC), 129.1 (ArC), 129.0 (ArC), 128.6 (ArC), 128.5 (ArC), 128.4 (ArC), 128.4 (ArC), 127.7 (ArC), 127.7 (ArC), 127.4 (ArC), 70.9 (C^2), 70.8 (C^2), 70.2 (C^1), 61.0 (C^1), 56.2 (C^4), 55.6 (C^4), 39.8 (C^5), 39.8 (C^5), 36.3 (C^3), 25.9 (C^3).

HRMS (ES^+) exact mass calculated for $[M+H]^+$ ($C_{17}H_{20}NO_2S^+$) requires **m/z** 302.1209, found **m/z** 302.1208.

dimethyl 1-benzyl-2-phenyl-1H-pyrrole-3,4-dicarboxylate (5r)

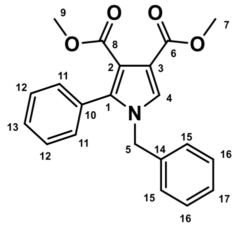

Prepared according to **General Procedure B**. Purification *via* FCC (1 : 1 pentane/EtOAc) gave **3k** as a colorless oil (63.8 mg, 67%). Data is consistent with the published literature.[8]

^{1}H NMR ($CDCl_3$, 400 MHz) δ_H: 7.42–7.33 (m, 3H, ArH), 7.32–7.23 (m, 7H, ArH), 7.00-6.91 (m, 1H, C^4H), 4.93 (s, 2H, C^5H_2), 3.81 (s, 3H, C^7H^3 or C^9H_3), 3.66 (s, 3H, C^7H_3 or C^9H_3).

^{13}C NMR ($CDCl_3$, 101 MHz) δ_C: 164.1 (C^7 or C^9), 137.3 (C^{14}), 136.4 (C^{10}), 130.5 (ArC), 130.1 (ArC), 128.9 (ArC), 128.3 (ArC), 128.1 (ArC), 127.0 (ArC), 126.9 (ArC), 115.6 (ArC), 115.1 (ArC), 51.7 (C^5), 51.5 (C^7 or C^9), 51.2 (C^7 or C^9).

HRMS (ES^+) exact mass calculated for $[M+H]^+$ ($C_{21}H_{20}NO_4^+$) requires **m/z** 350.1392, found **m/z** 350.1392.

5. Synthesis and characterization of compounds related to the intramolecular and asymmetric exemples

methyl 2-(3-(but-3-en-1-yl)-2-oxopyrrolidin-1-yl)acetate (S1)

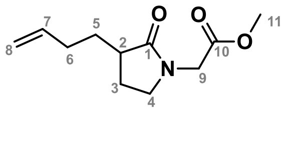

Prepared according to **General Procedure G**. Purification *via* FCC (2 : 3 pentane/EtOAc) gave **S1** as a pale yellow oil (77 mg, 56%).

IR 2980, 1748 (C^{10}=O), 1687 (C^{1}=O), 1438, 1281, 1210, 1180.

^{1}H NMR ($CDCl_3$, 400 MHz) δ_H: 5.81 (ddt, 1H, *J* = 16.9, 10.2, 6.6 Hz, $C^{7}H$), 5.05 (app dq, 1H, *J* = 17.1, 1.7 Hz, $C^{8}Ha$), 4.98 (ddt, 1H, *J* = 10.1, 2.2, 1.3 Hz, $C^{8}Hb$), 4.10 (roofed d, 1H, *J* = 17.5 Hz, $C^{9}Ha$), 4.03 (d, 1H, *J* = 17.6 Hz, $C^{9}Hb$), 3.73 (s, 3H, $C^{11}H_3$), 3.35–3.45 (m, 2H, $C^{4}H_2$), 2.47 (qd, 1H, *J* = 9.0, 4.5 Hz, $C^{2}H$), 2.08–2.28 (m, $C^{3}Ha$, $C^{6}H_2$), 1.99 (dddd, 1H, *J* = 13.7, 9.3, 6.8, 4.4 Hz, $C^{5}Ha$), 1.74 (app dq, 1H, *J* = 12.7, 8.6 Hz, $C^{3}Hb$), 1.43–1.52 (m, 1H, $C^{5}Hb$).

^{13}C NMR ($CDCl_3$, 101 MHz) δ_C: 177.5 (C^{1}), 169.4 (C^{10}), 138.1 (C^{7}), 115.3 (C^{8}), 52.3 (C^{11}), 46.1 (C^{4}), 44.2 (C^{9}), 40.8 (C^{2}), 31.4 (C^{6}), 30.5 (C^{5}), 25.2 (C^{3}).

HRMS (ES^{+}) exact mass calculated for $[M+H]^{+}$ ($C_{11}H_{17}O_3NNa^{+}$) requires **m/z** 234.1101, found **m/z** 234.1104.

tert-butyl (E)-5-(1-(2-methoxy-2-oxoethyl)-2-oxopyrrolidin-3-yl)pent-2-enoate (6)

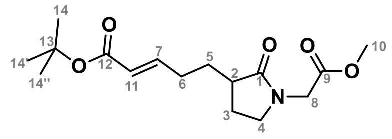

The lactam **S1** was dissolved in degassed CH_2Cl_2 (3 mL/mmol substrate). *t*-butyl acrylate was added (3.00 equiv.), followed by CuI (6 mol%) and Grubbs-II (5 mol%). The resulting solution was heated for 3 h at 40 °C. The reaction mixture was cooled down to rt and directly loaded on a Silica Gel Column. Purification *via* FCC (4 : 1 pentane/EtOAc) gave **6** as a pale yellow oil (67 mg, 63%)

IR 2977, 2932, 1751 (C^9=O), 1694 (C^{12}=O), 1693 (C^1=O), 1438, 1367, 1285, 1212, 1151.

^{1}H NMR ($CDCl_3$, 500 MHz) δ_H: 6.85 (dt, 1H, *J* = 15.7, 6.9 Hz, C^7H), 5.78 (dt, 1H, *J* = 15.6, 1.6 Hz, C^{11}H), 4.09 (roofed d, 1H, *J* = 17.6 Hz, C^8Ha), 4.04 (roofed d, 1H, *J* = 17.6 Hz, C^8Hb), 3.73 (s, 3H, $C^{10}H_3$), 3.43 (dt, 1H, *J* = 9.2, 7.6 Hz, C^4Ha), 3.39 (td, 1H, *J* = 9.0, 2.6 Hz, C^4Hb), 2.47 (qd, 1H, *J* = 8.8, 5.0 Hz, C^2H), 2.22–2.37 (m, 3H, C^3Ha, C^6H_2), 2.03 (dddd, 1H, *J* = 13.9, 9.1, 6.9, 5.0 Hz, C^5Ha), 1.73 (dq, 1H, *J* = 12.8, 8.5 Hz, C^3Hb), 1.50–1.61 (m, 1H, C^5Hb), 1.48 (s, 9H, $C^{14}H_3$, $C^{14'}H_3$, $C^{14''}H_3$).

^{13}C NMR ($CDCl_3$, 101 MHz) δ_C: 177.0 (C^1), 169.3 (C^9), 166.1 (C^{12}), 146.8 (C^7), 123.9 (C^{11}), 80.3 (C^{13}), 52.4 (C^{10}), 46.1 (C^4), 44.2 (C^8), 40.7 (C^2), 29.8 (C^5), 29.6 (C^6), 28.3 (C^{14}, $C^{14'}$, $C^{14''}$), 25.2 (C^3).

HRMS (ES^+) exact mass calculated for $[M+H]^+$ ($C_{16}H_{25}O_5NNa^+$) requires **m/z** 334.1625, found **m/z** 334.1626.

1-(*tert*-butyl) 2-methyl (1S,2S,31R,5aS,7aR)-octahydro-2H-cyclopenta[gh]pyrrolizine-1,2-dicarboxylate (7)

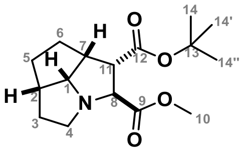

Prepared according to **General Procedure A** from **6** (no coupling partner used). Purification *via* FCC (1 : 1 to 1 : 2 pentane/EtOAc) gave **7** as a pale yellow oil (28 mg, 72%).

IR 2950, 1742 (C^{12}=O, C^{9}=O (shoulder)), 1693 (C^{1}=O), 1368, 1199, 1158.

^{1}H NMR ($CDCl_3$, 500 MHz) δ_H: 4.09 (t, 1H, *J* = 7.5 Hz, C^{1}H), 3.81 (d, 1H, *J* = 7.0 Hz, C^{8}H), 3.70 (s, 3H, $C^{10}H_3$), 3.15 (ddd, 1H, *J* = 10.0, 8.0, 3.1 Hz, C^{4}Ha), 3.04 (qd, 1H, *J* = 7.5, 3.2 Hz, C^{7}H), 2.82 (t, 1H, *J* = 7.2 Hz, C^{11}H), 2.60 (td, 1H, *J* = 9.8, 5.8 Hz, C^{4}Hb), 2.47–2.52 (m, 1H, C^{2}H), 1.90 (ddt, 1H, *J* = 12.7, 9.9, 7.0 Hz, C^{3}Ha), 1.74–1.81 (m, 2H, C^{5}Ha, C^{6}Ha), 1.54–1.62 (m, 2H, C^{3}Hb,C^{6}Hb), 1.22–1.28 (m, 1H, C^{5}Hb).

^{13}C NMR ($CDCl_3$, 101 MHz) δ_C: 172.3 (C^{9}), 171.3 (C^{12}), 81.0 (C^{13}), 75.7 (C^{1}), 69.9 (C^{8}), 53.7 (C^{4}), 52.3 (C^{11}), 51.9 (C^{10}), 47.4 (C^{7}), 44.0 (C^{2}), 31.7 (C^{6}), 30.8 (C^{5}), 30.6 (C^{3}), 28.1 (C^{14}, $C^{14'}$, $C^{14''}$).

HRMS (ES^+) exact mass calculated for $[M+H]^+$ ($C_{16}H_{26}O_4N^+$) requires **m/z** 296.1856, found **m/z** 296.1858.

methyl (2R,3S,7aS)-2-((S)-4-isopropyl-5,5-dimethyl-2-oxooxazolidine-3-carbonyl)-3-phenyltetrahydro-1H-pyrrolizine-7a(5H)-carboxylate (8a) and methyl (1S,3S,7aS)-1-((S)-4-isopropyl-5,5-dimethyl-2-oxooxazolidine-3-carbonyl)-3-phenyltetrahydro-1H-pyrrolizine-7a(5H)-carboxylate (8b)

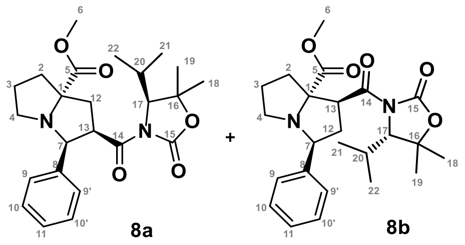

Prepared according to **General Procedure A** from **1a** and a Super quat chiral auxiliary derived from L-valine. Purification *via* FCC (9 : 1 pentane/EtOAc) gave **8a** as a pale yellow oil (43 mg, 40%).

mp 120 – 122 °C (from EtOAc).

IR 2961, 1775 (C^{14}=O), 1728 (C^5=O), 1694 (C^{15}=O), 1494, 1393, 1314, 1277, 1173.

^{1}H NMR ($CDCl_3$, 500 MHz) δ_H: 7.23–7.28 (m, 2H, C^{10}H, $C^{10'}$H), 7.18-7.22 (m, 3H, C^9H, $C^{9'}$H, C^{10}H), 5.04 (d, 1H, *J* = 9.2 Hz, C^7H), 4.76 (ddd, 1H, *J* = 11.3, 9.2, 8.4 Hz, C^{13}H), 3.79 (s, 3H, C^6H_3), 3.65 (d, 1H, *J* = 3.2 Hz, C^{17}H), 2.57–2.65 (m, 2H, C^4Ha, C^{12}H), 2.50–2.56 (m, 1H, C^4Hb), 2.28–2.36 (m, 1H, C^2Ha), 2.01 (qqd, 1H, *J* = 7.0, 6.8, 3.2 Hz, C^{20}H), 1.86–1.96 (m, 2H, C^2Hb, C^3Ha), 1.77–1.86 (m, 1H, C^3Hb), 1.32 (s, 3H, $C^{18}H_3$), 0.93 (d, 1H, *J* = 7.0 Hz, $C^{21}H_3$), 0.87 (d, 1H, *J* = 6.8 Hz, $C^{22}H_3$), 0.44 (s, 3H, $C^{19}H_3$).

^{13}C NMR ($CDCl_3$, 126 MHz) δ_C: 177.4 (C^5), 171.9 (C^{14}), 152.8 (C^{15}), 137.7 (C^8), 130.1 (C^9, $C^{9'}$), 128.5 (C^{10}, $C^{10'}$), 128.2 (C^{11}), 82.4 (C^{16}), 75.9 (C^1), 66.6 (C^{17}), 65.4 (C^7), 52.7 (C^{13}), 52.6 (C^6), 47.6 (C^4), 38.3 (C^{12}), 36.4 (C^2), 29.6 (C^{20}), 27.9 (C^3), 27.8 (C^{18}), 21.6 (C^{19}), 21.3 (C^{21}), 17.3 (C^{22}).

HRMS (ES^+) exact mass calculated for $[M+H]^+$ ($C_{24}H_{33}O_5N_2^+$) requires **m/z** 429.2383, found **m/z** 429.2384.

Further elution gave **8b** (40 mg, 38%).

mp 140 – 142 °C (from EtOAc).

IR 2955, 2926, 1778 (C^{14}=O), 1734 (C^{5}=O), 1691 (C^{15}=O), 1457, 1363, 1312, 1221, 1174, 1121, 1068.

^{1}H NMR ($CDCl_3$, 500 MHz) δ_H: 7.45–7.48 (m, 2H, C^{9}H, $C^{9'}$H), 7.33–7.37 (m, 2H, C^{10}H, $C^{10'}$H), 7.27–7.31 (m, 1H, C^{11}H), 4.83 (dd, 1H, *J* = 11.7, 6.5 Hz, C^{13}H), 4.67 (dd, 1H, *J* = 12.9, 4.2 Hz, C^{7}H), 4.14 (d, 1H, *J* = 3.3 Hz, C^{17}H), 3.81 (s, 3H, $C^{6}H_3$), 2.76 (app q, 1H, *J* = 12.1 Hz, C^{12}Ha), 2.40-2.49 (m, 2H, C^{2}Ha, C^{4}Ha), 2.31–2.37 (m, 1H, C^{4}Hb), 2.15–2.24 (m, 2H, C^{12}H, C^{20}H), 1.58–1.70 (m, 3H, C^{2}Hb, $C^{3}H_2$), 1.52 (s, 3H, $C^{18}H_3$), 1.47 (s, 3H, $C^{19}H_3$), 1.08 (d, 3H, J = 7.0 Hz, $C^{21}H_3$), 0.97 (s, 3H, $C^{22}H_3$).

^{13}C NMR ($CDCl_3$, 126 MHz) $\delta_{C:}$ 175.7 (C^{5}), 172.7 (C^{14}), 153.4 (C^{15}), 138.0 (C^{8}), 129.0 (C^{9}, $C^{9'}$), 128.3 (C^{10}, $C^{10'}$), 127.7 (C^{11}), 83.0 (C^{16}), 76.9 (C^{1}, masked by chloroform peak), 67.7 (C^{17}), 64.8 (C^{7}), 53.0 (C^{6}), 50.5 (C^{4}), 49.8 (C^{13}), 31.8 (C^{2}), 31.1 (C^{12}), 29.7 (C^{20}), 28.7 (C^{18}), 25.6 (C^{3}), 21.7 (C^{19}), 21.6 (C^{21}), 17.3 (C^{22}).

HRMS (ES^+) exact mass calculated for $[M+H]^+$ ($C_{24}H_{33}O_5N_2^+$) requires **m/z** 429.2383, found **m/z** 429.2384.

6. NMR data

NMR spectra for 1m.

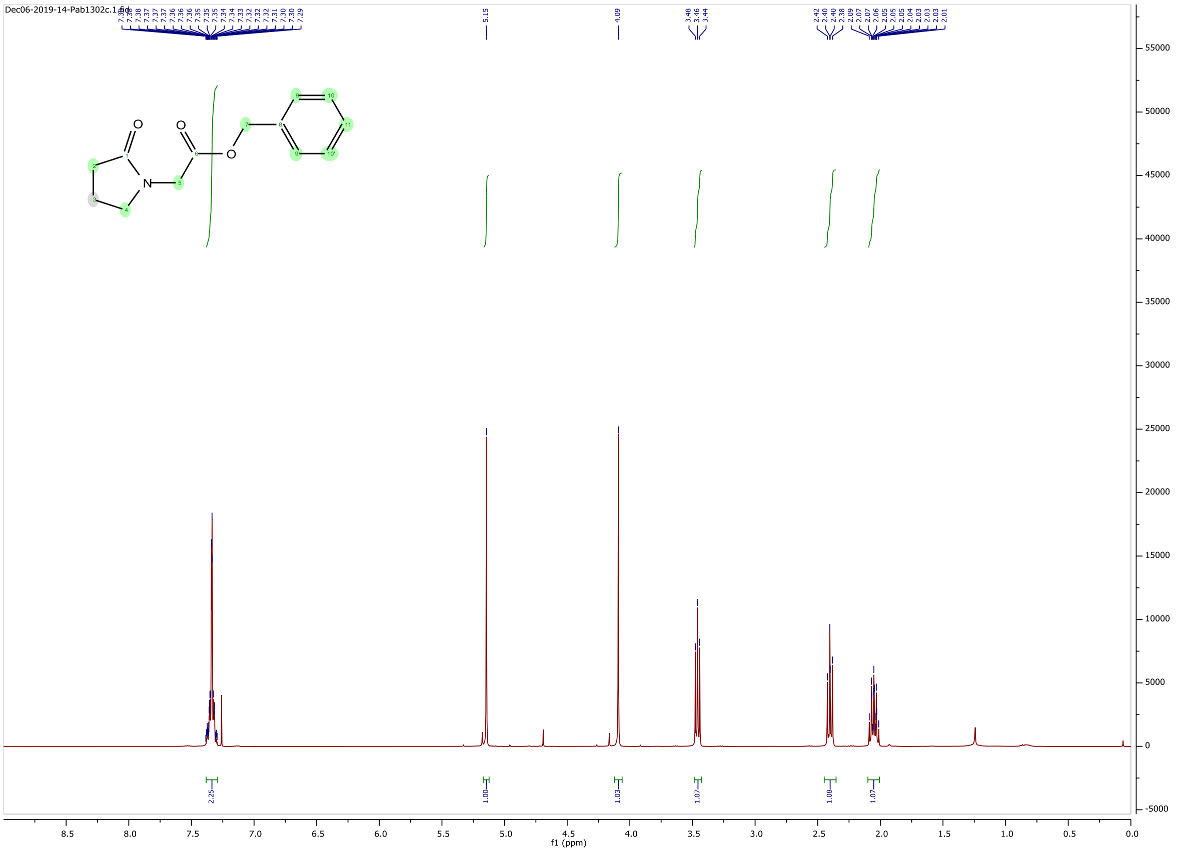

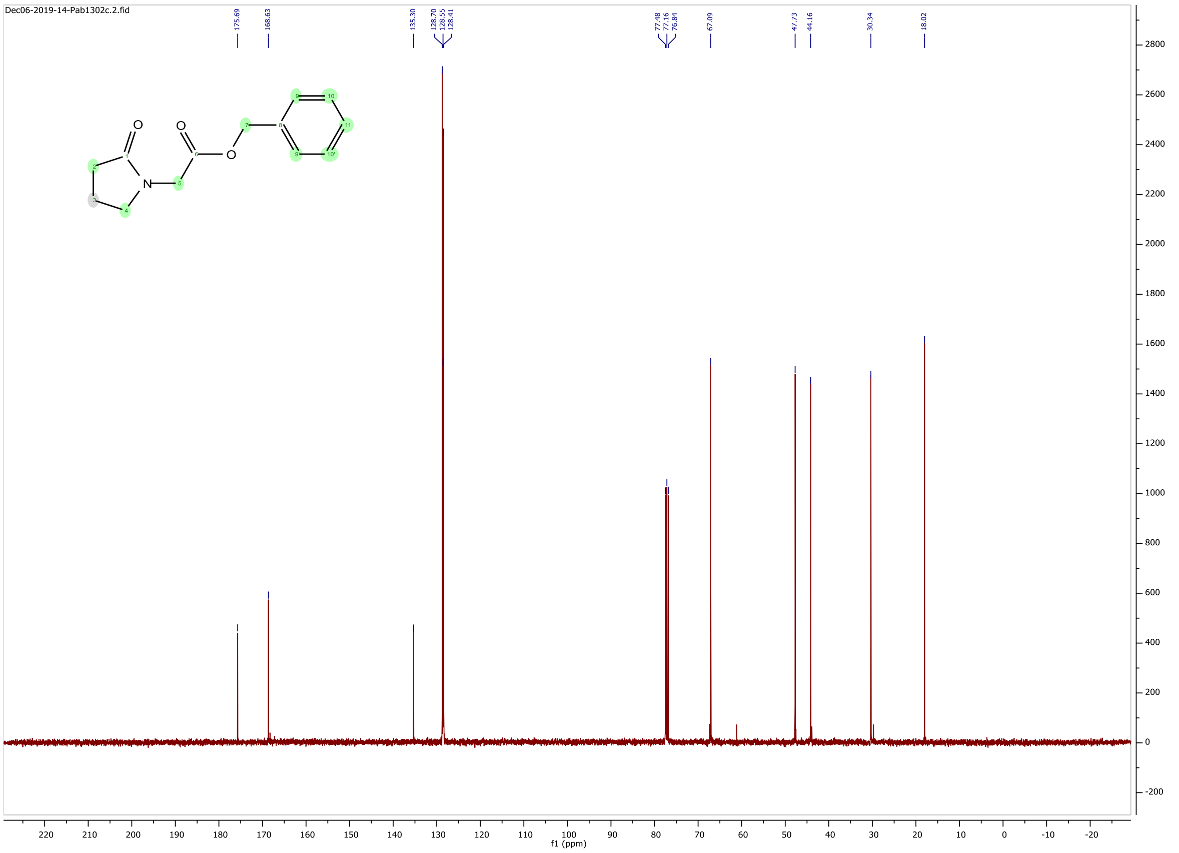

NMR spectra for 4c.

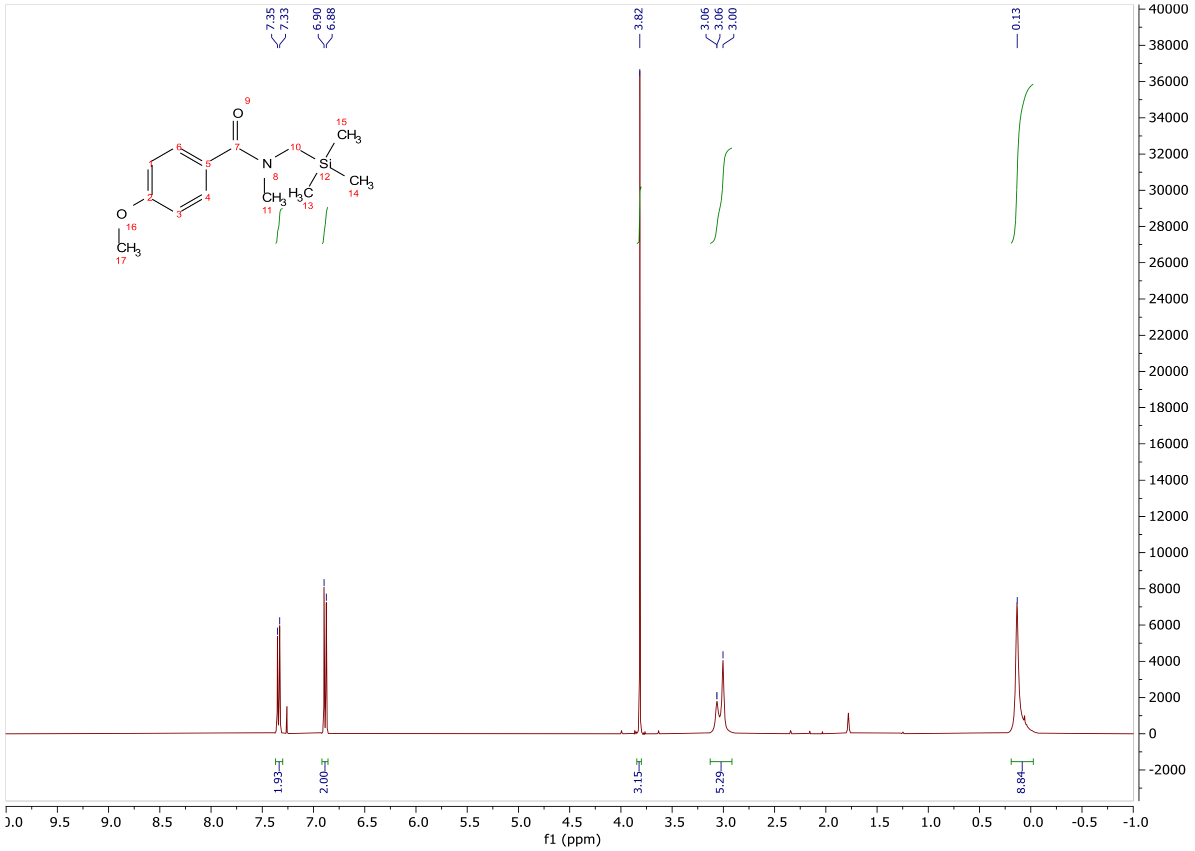

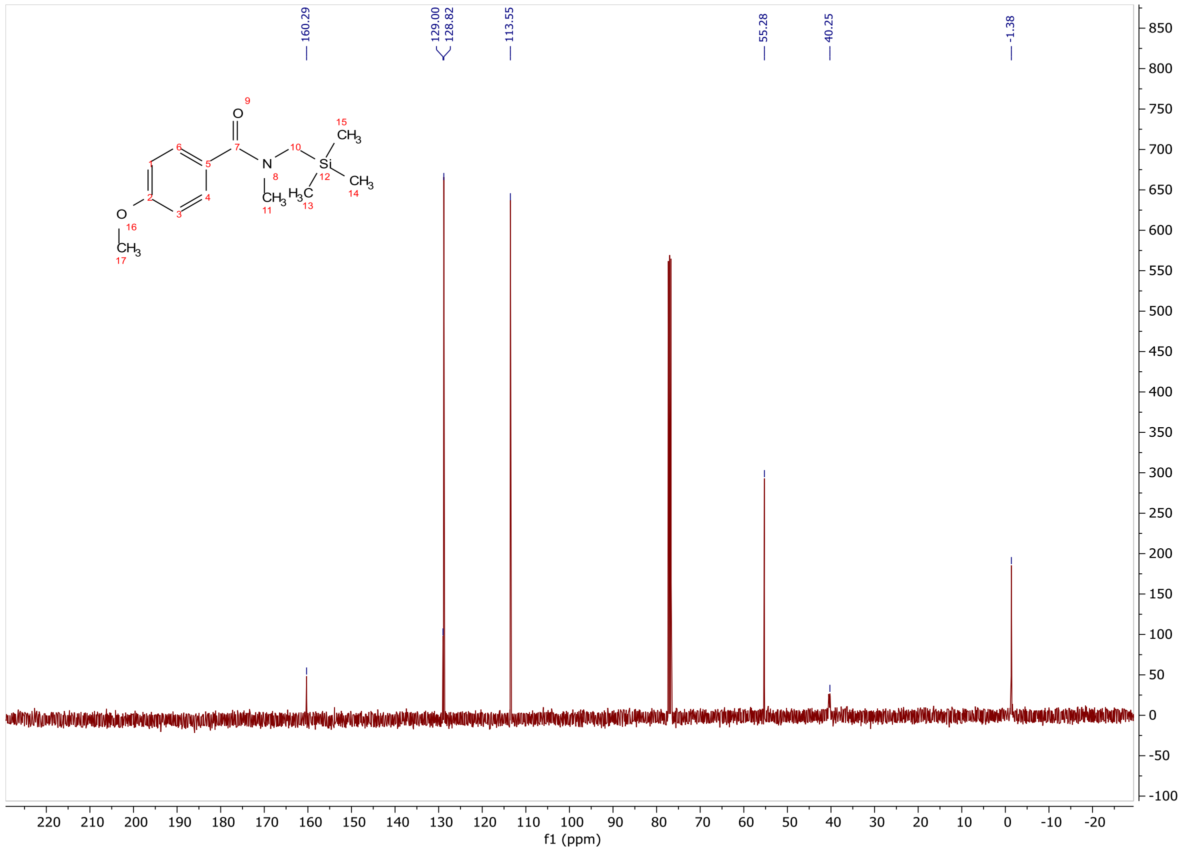

NMR spectra for 4d.

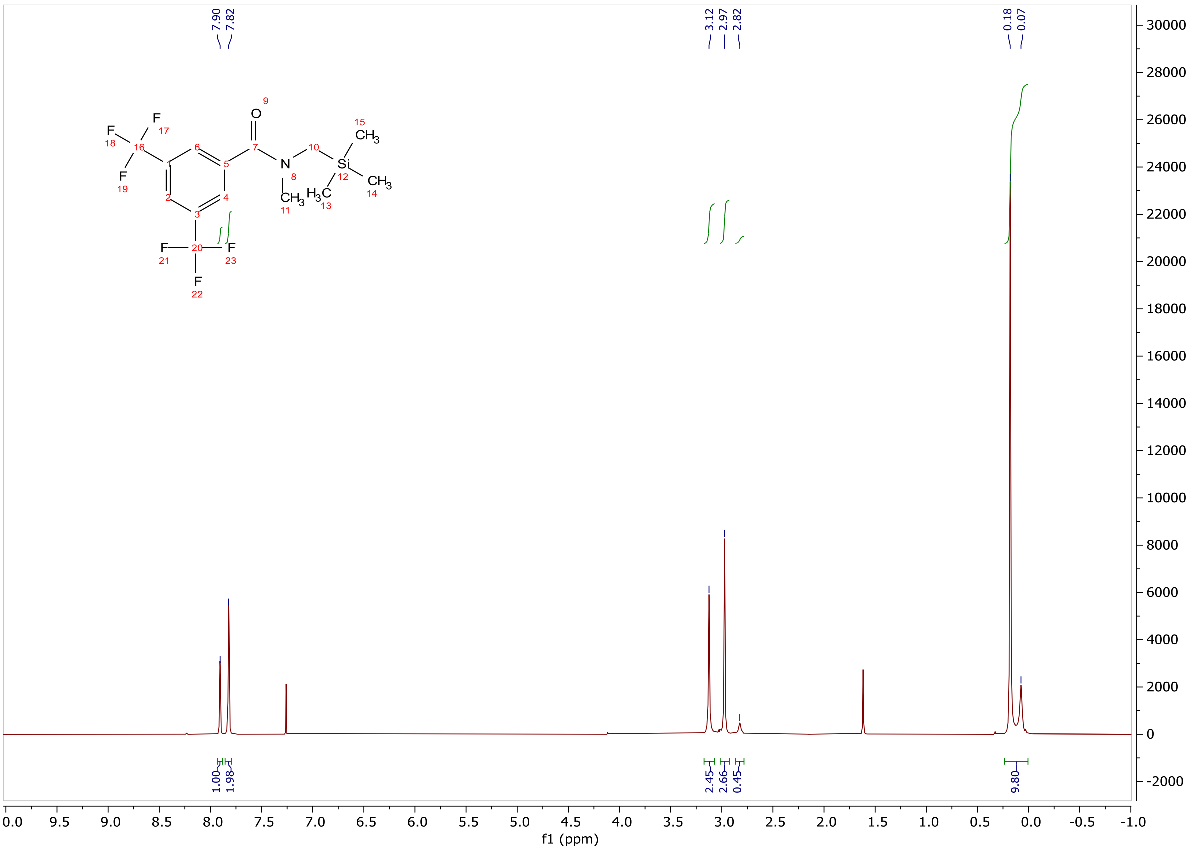

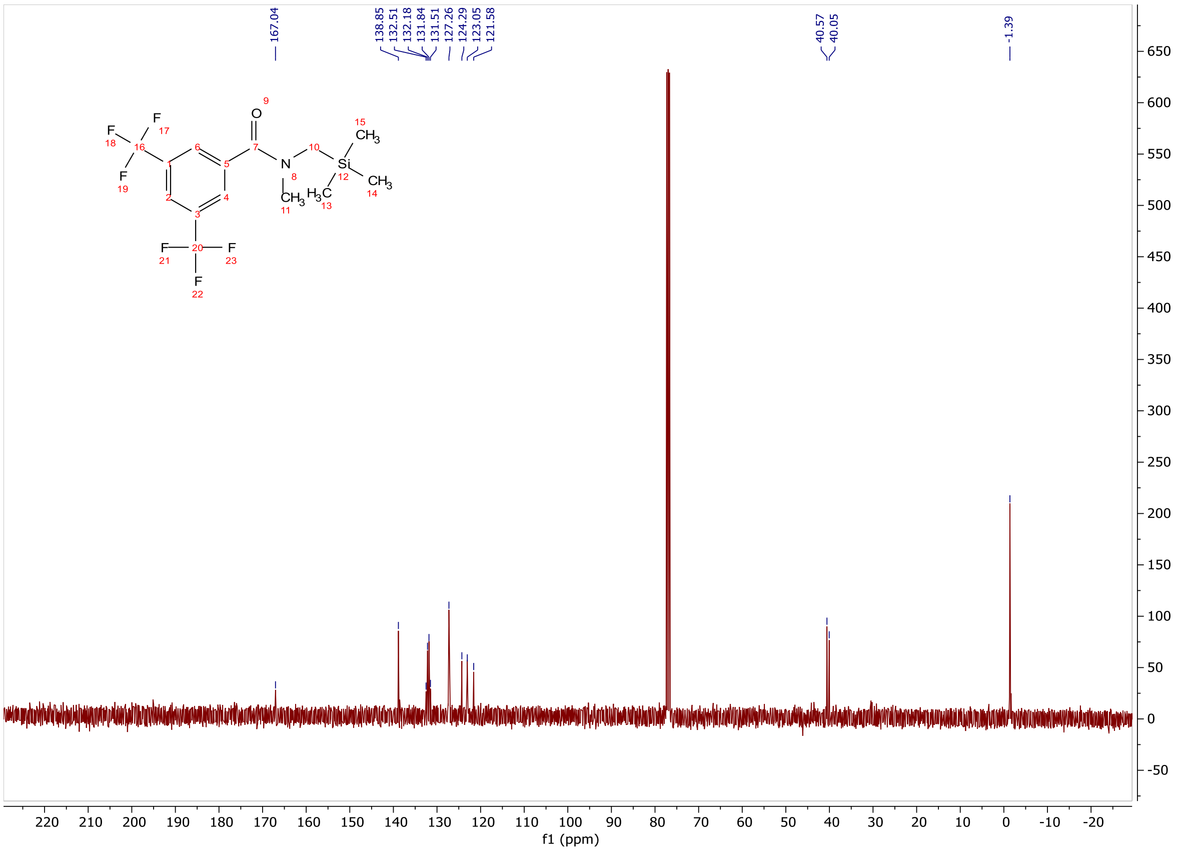

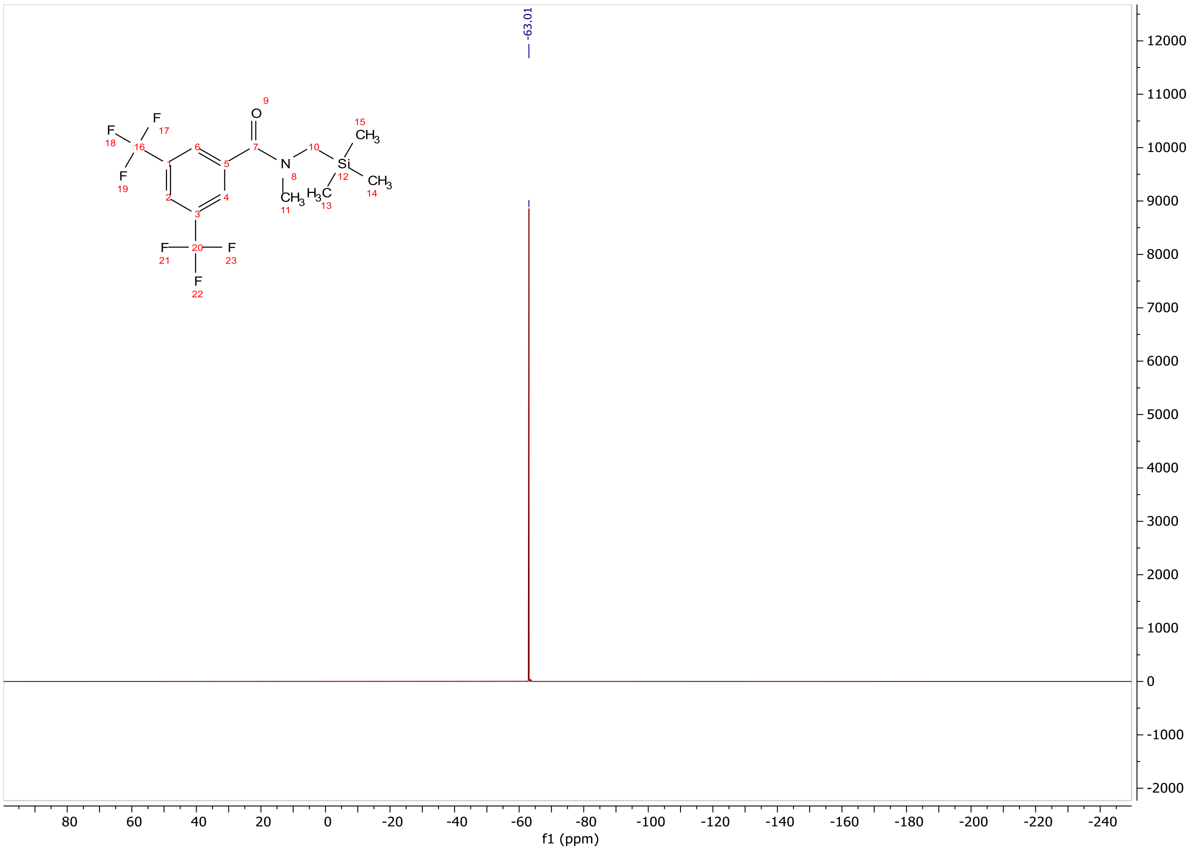

-63.01
12000
11000
10000
9000
8000
7000
6000
5000
4000
3000
2000
1000
0
-1000
-2000
80
60
40
20
0
-20
-40
-60
-80
-100
-120
-140
-160
-180
-200
-220
-240
f1 (ppm)

NMR spectra for 4e.

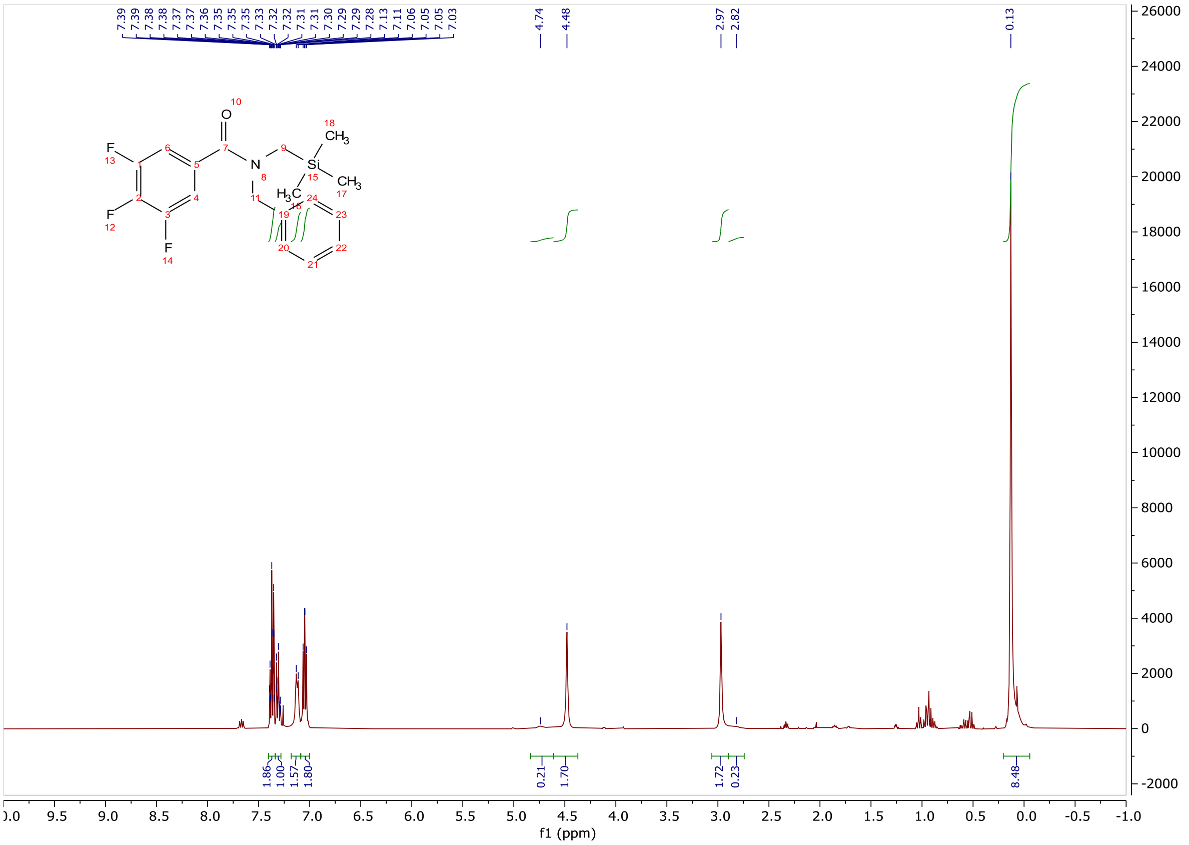

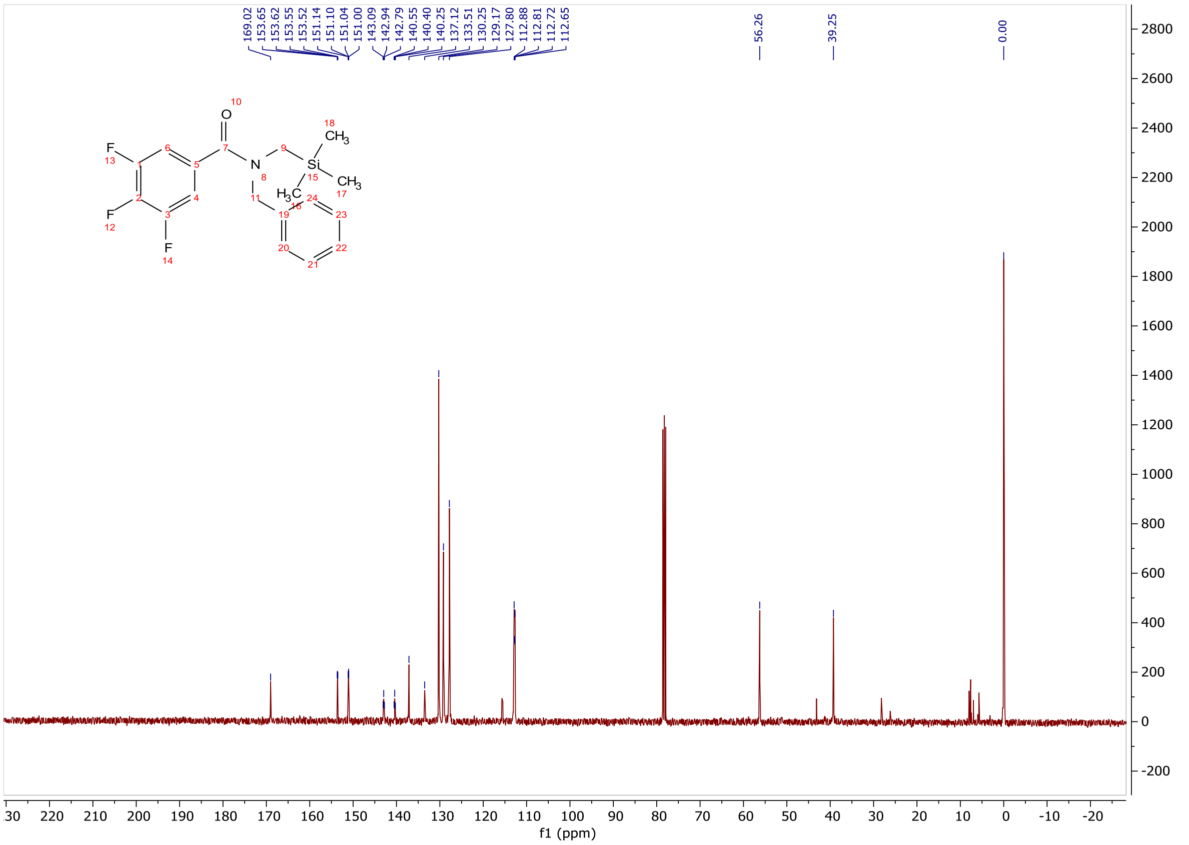

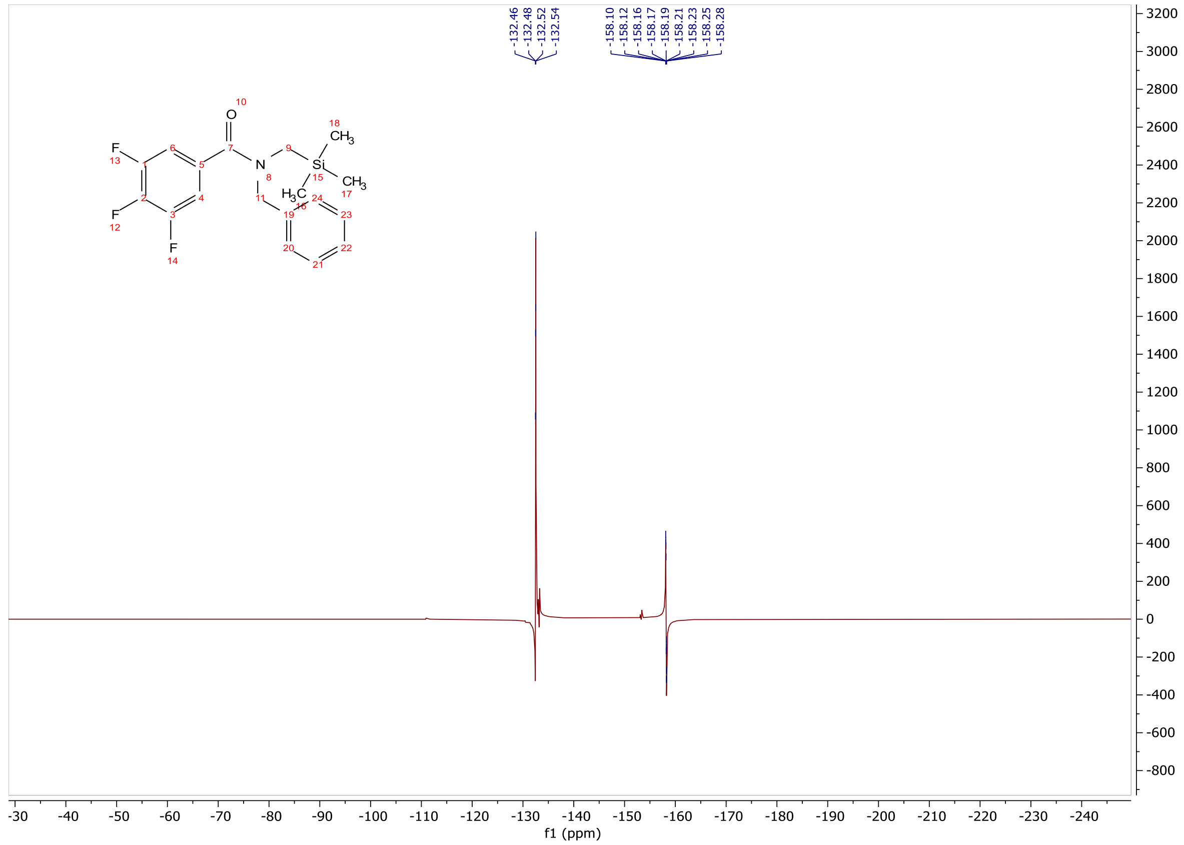

-132.46
-132.48
-132.52
-132.54
-158.10
-158.12
-158.16
-158.17
-158.19
-158.21
-158.23
-158.25
-158.28
f1 (ppm)

NMR spectra for 4f.

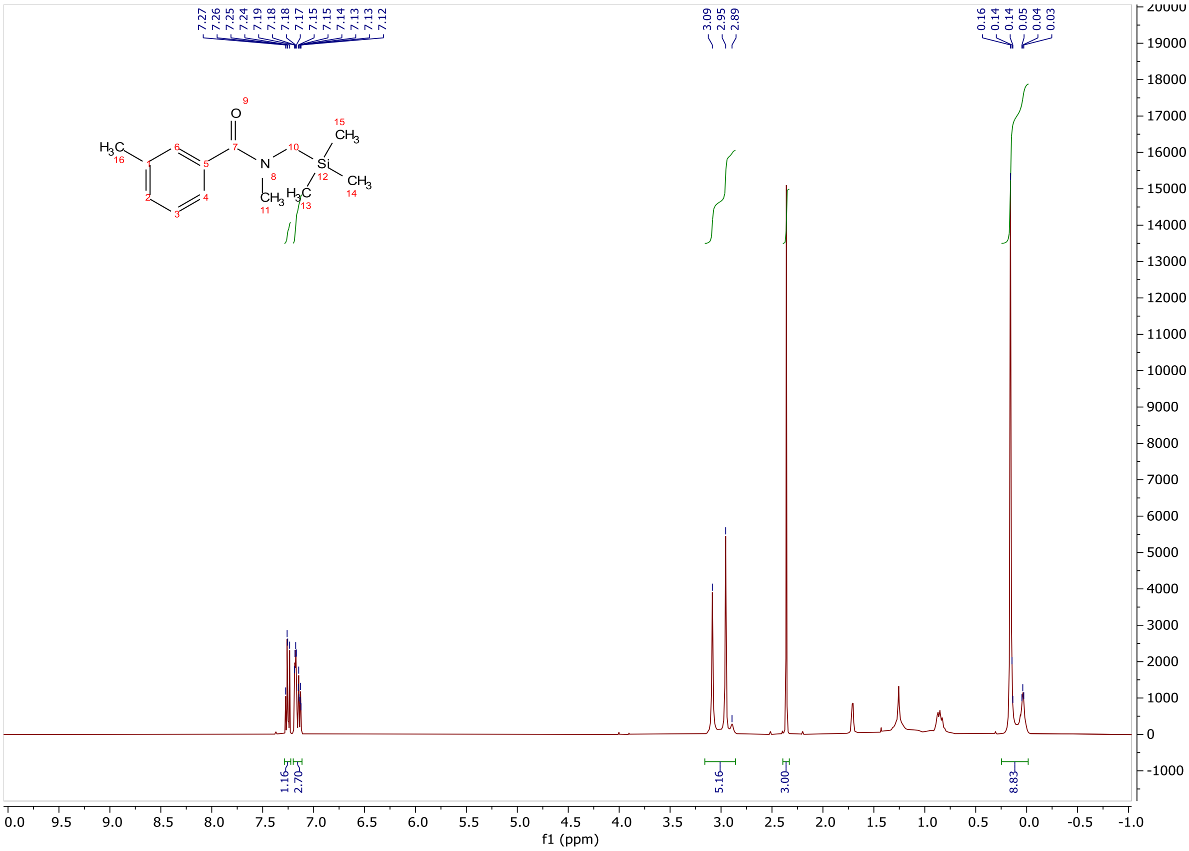

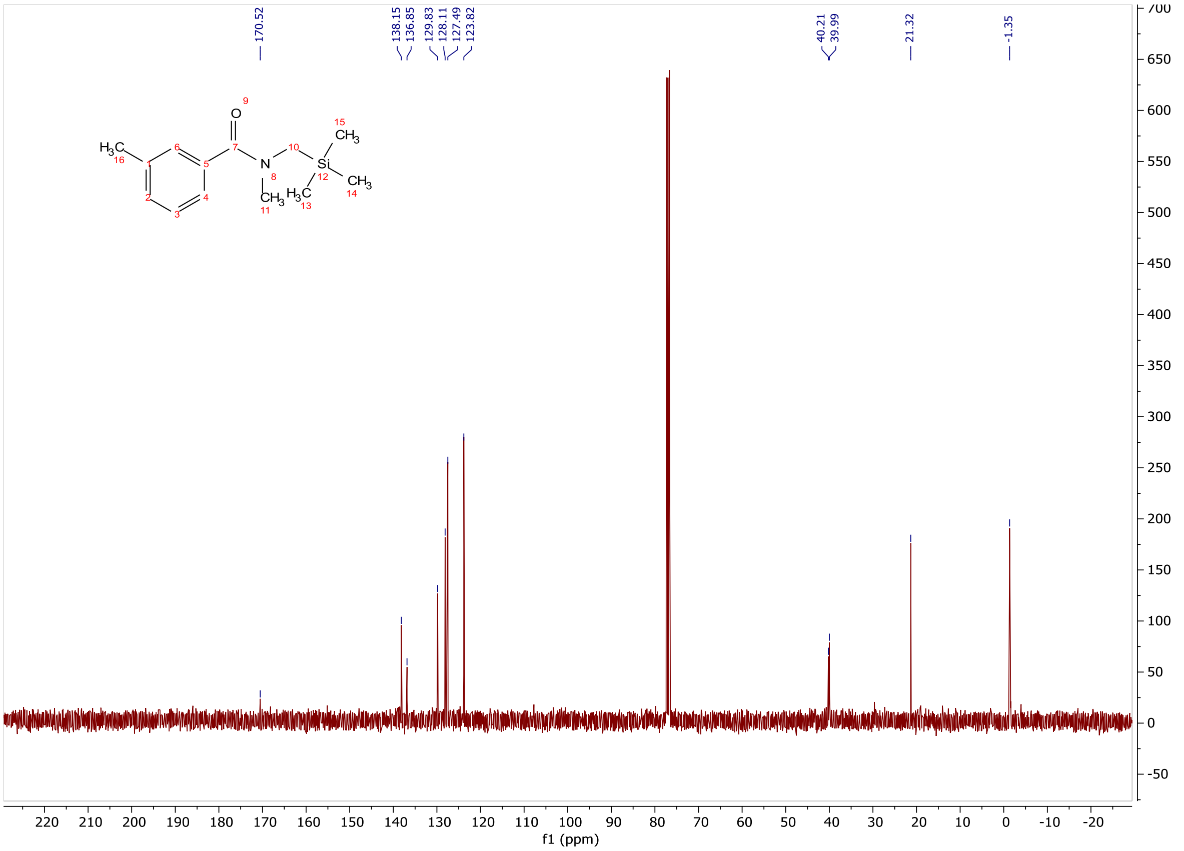

NMR spectra for 4g.

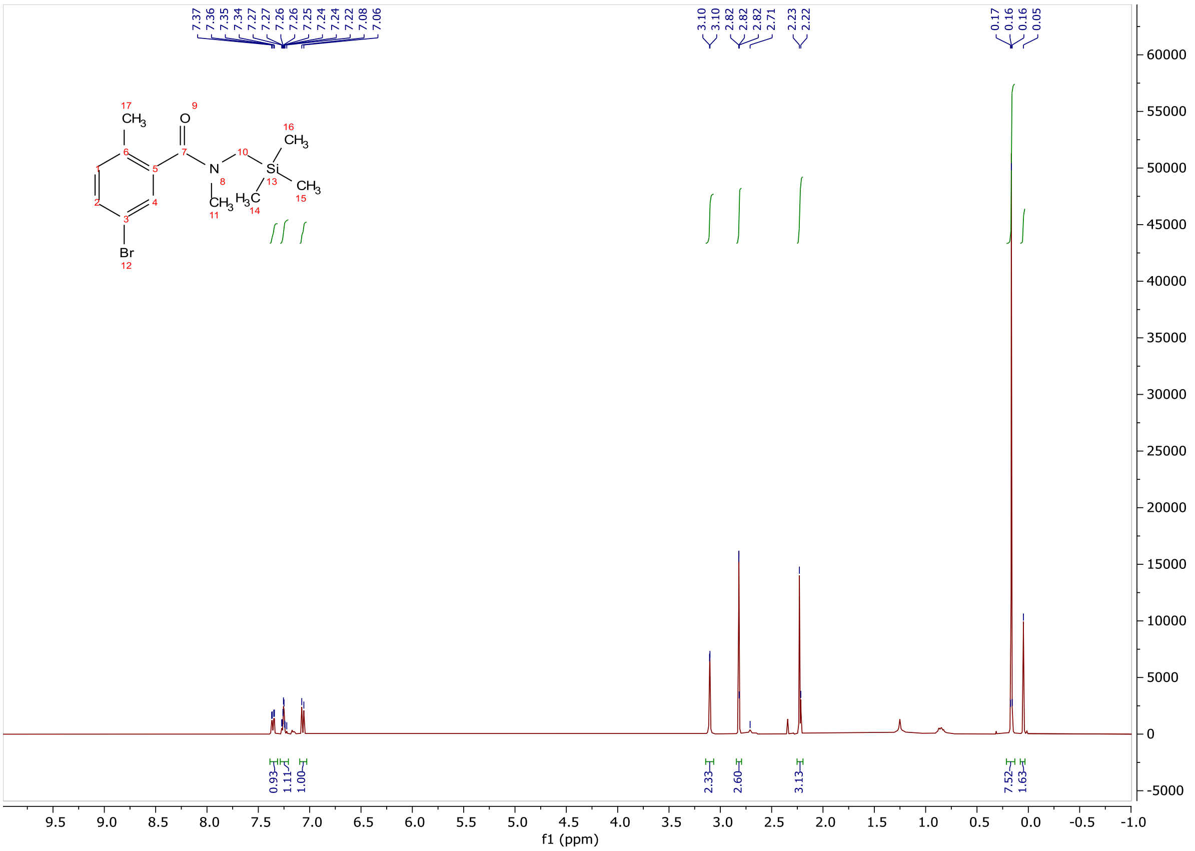

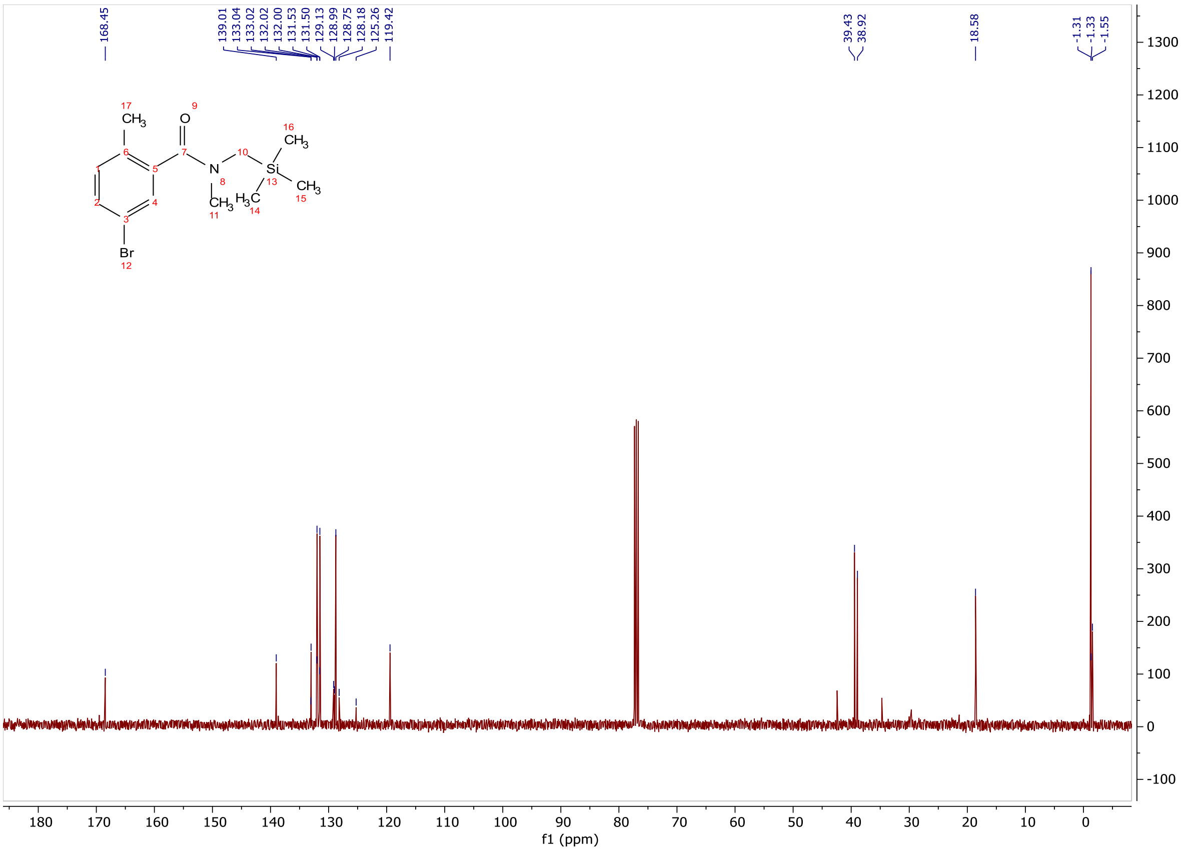

NMR spectra for 4h.

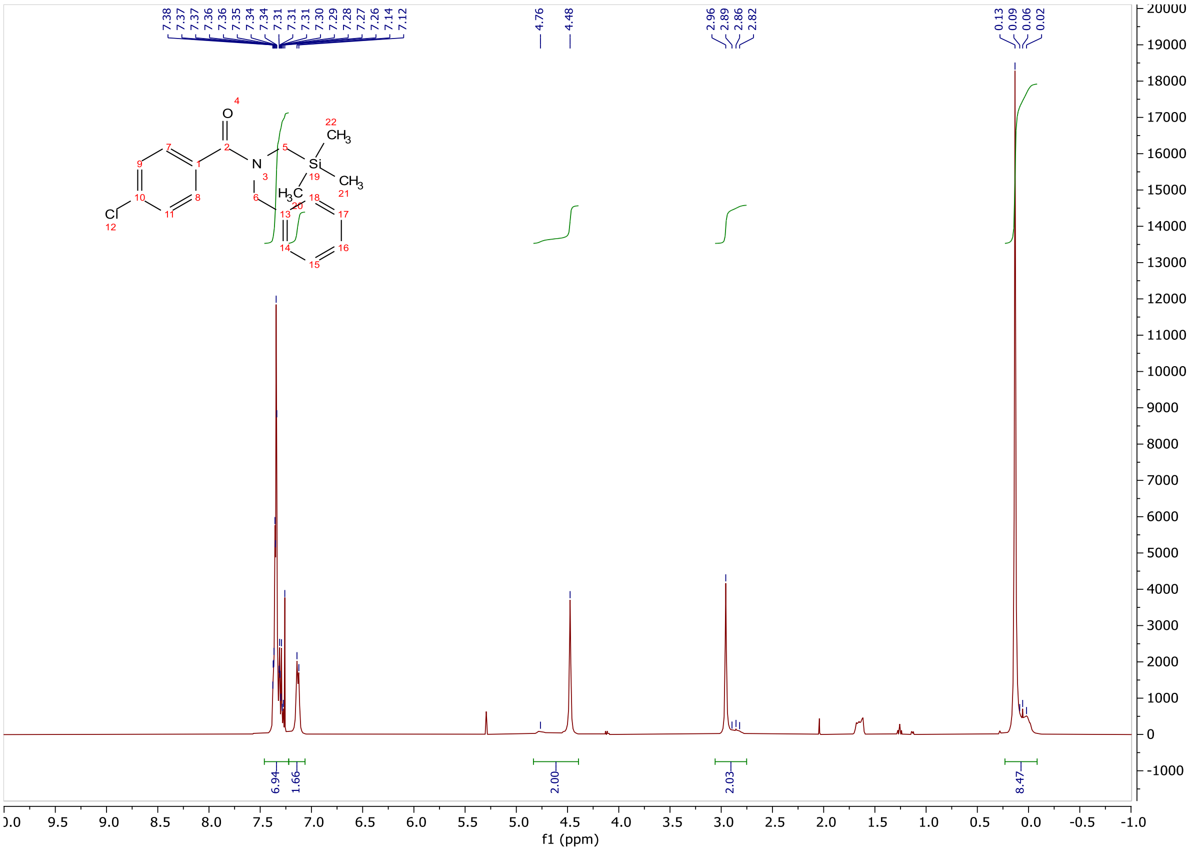

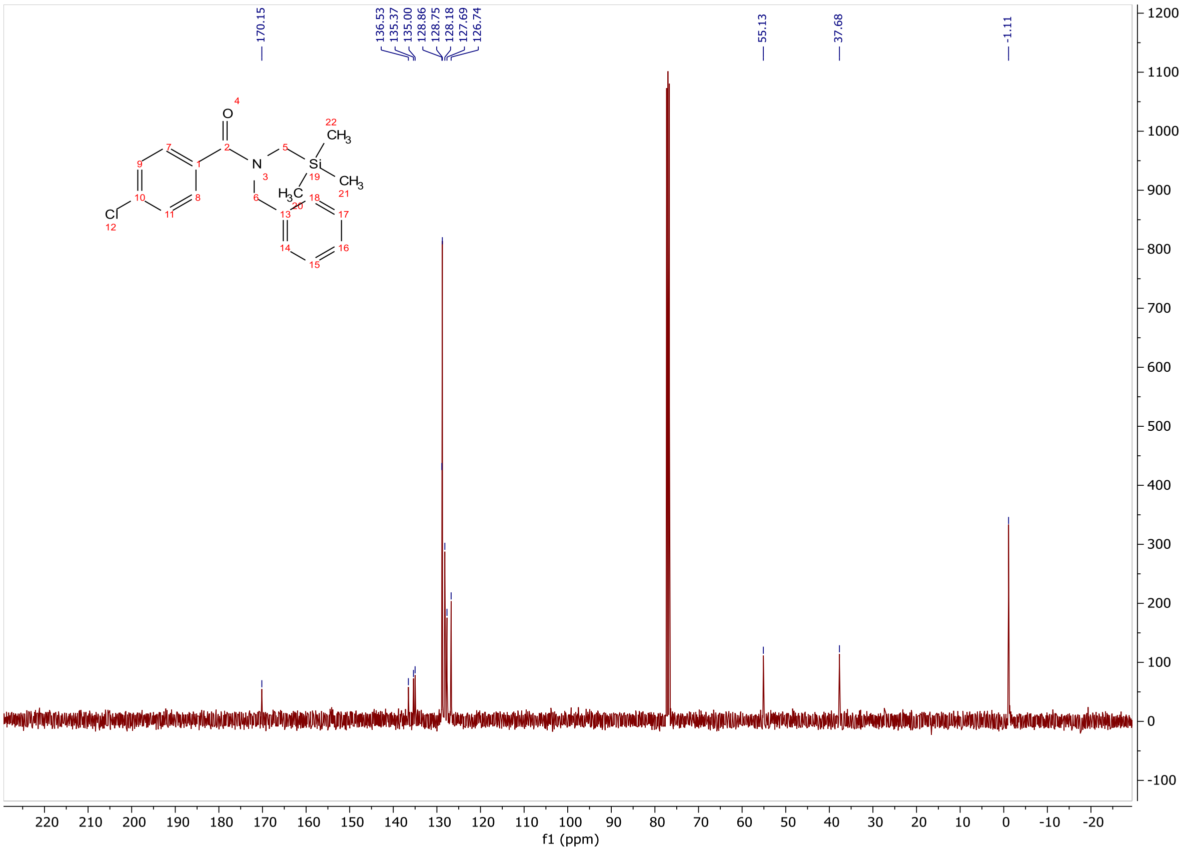

NMR spectra for 4i.

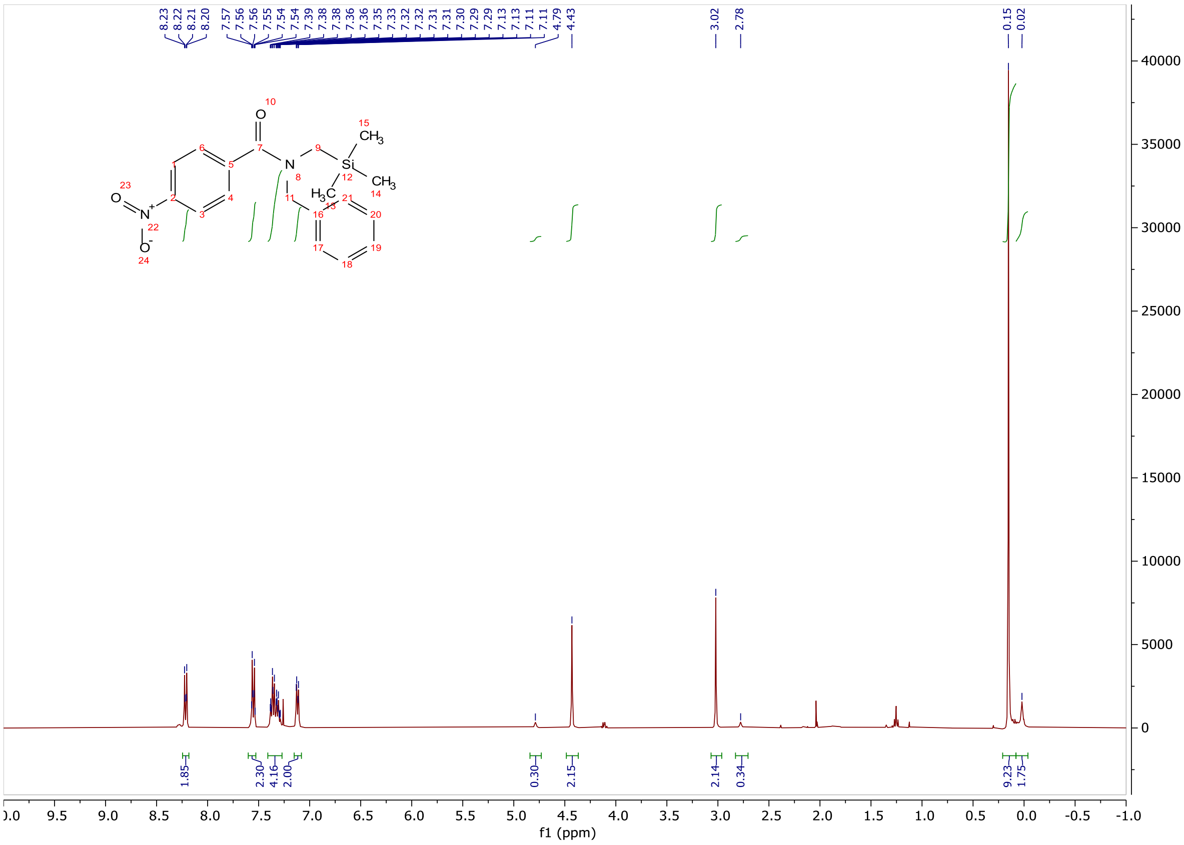

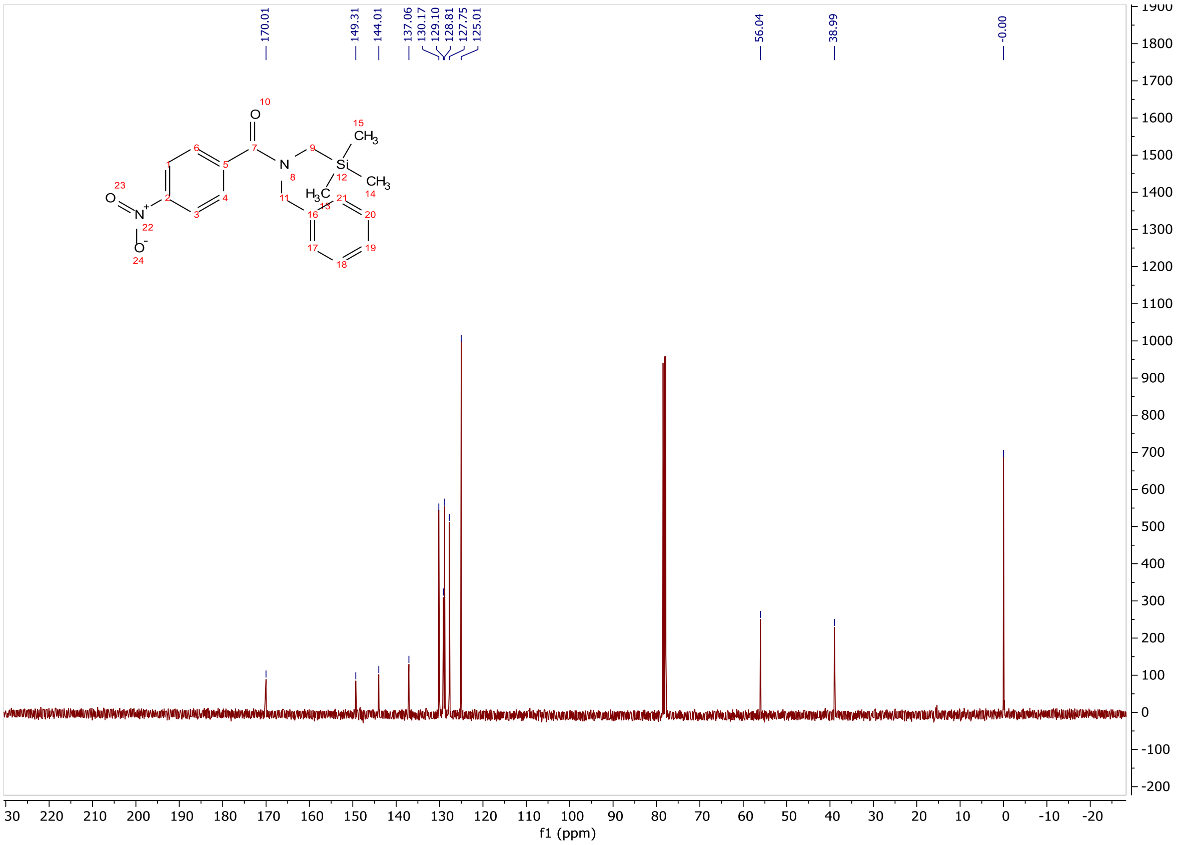

NMR spectra for 4j.

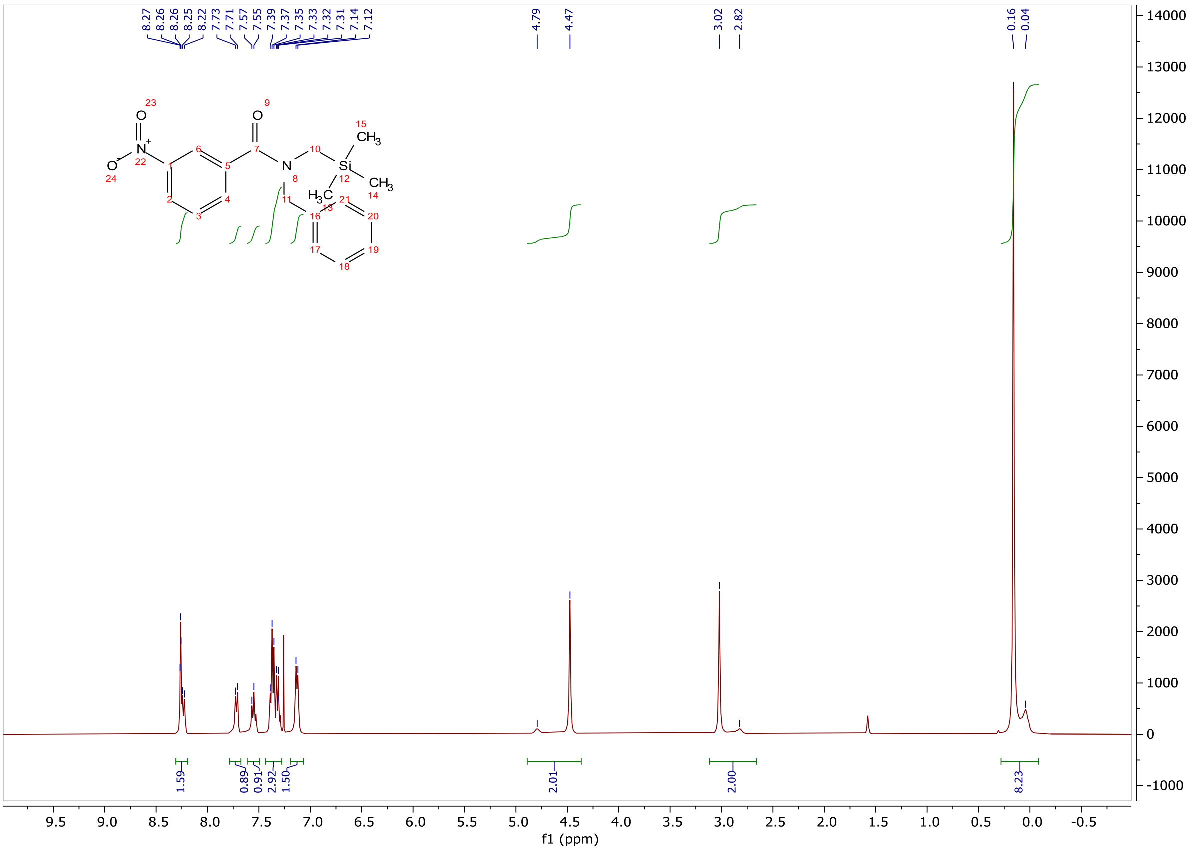

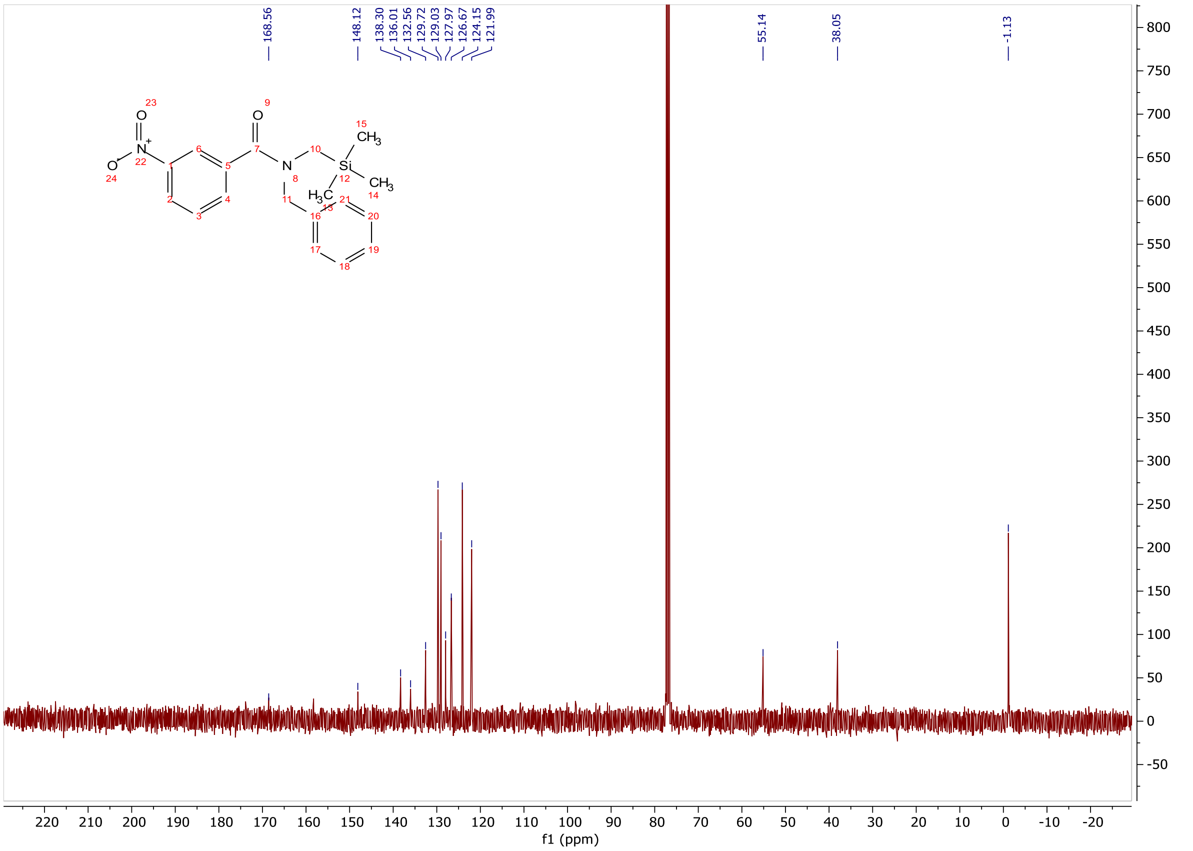

NMR spectra for 4k.

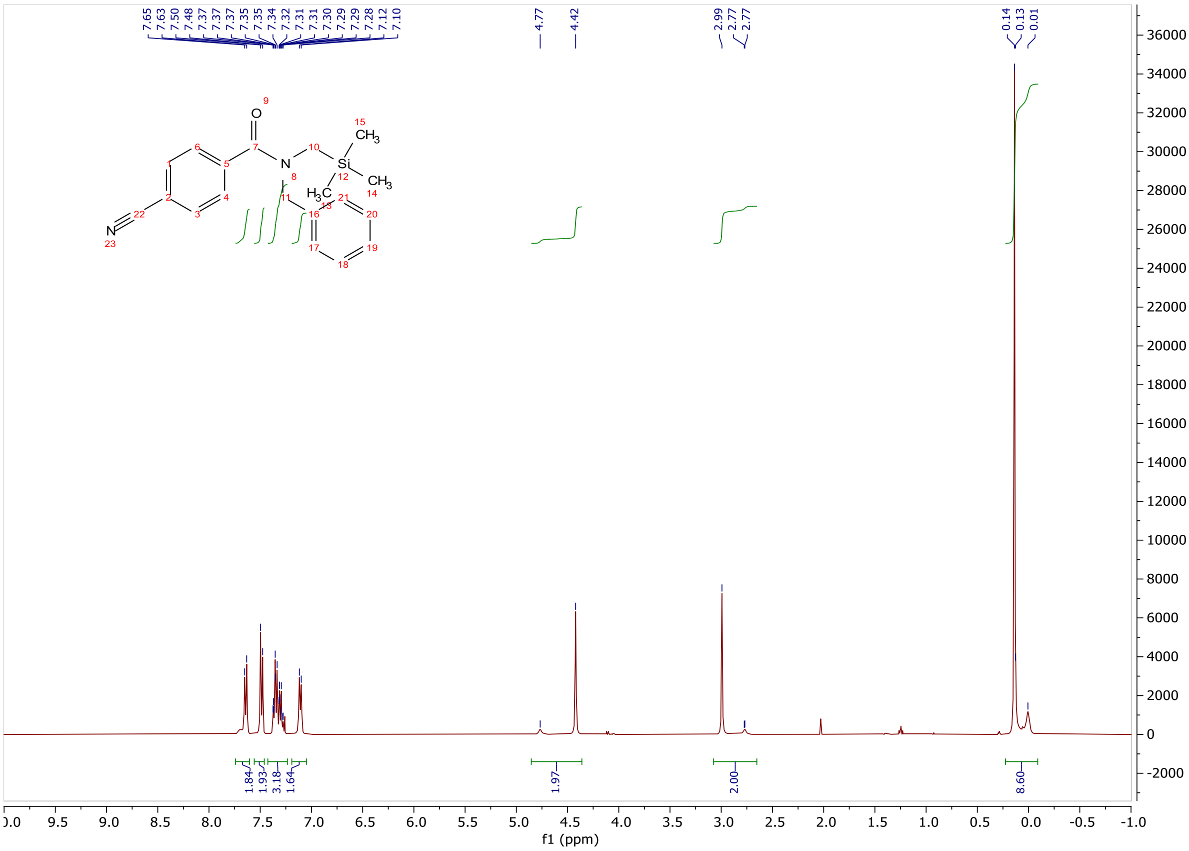

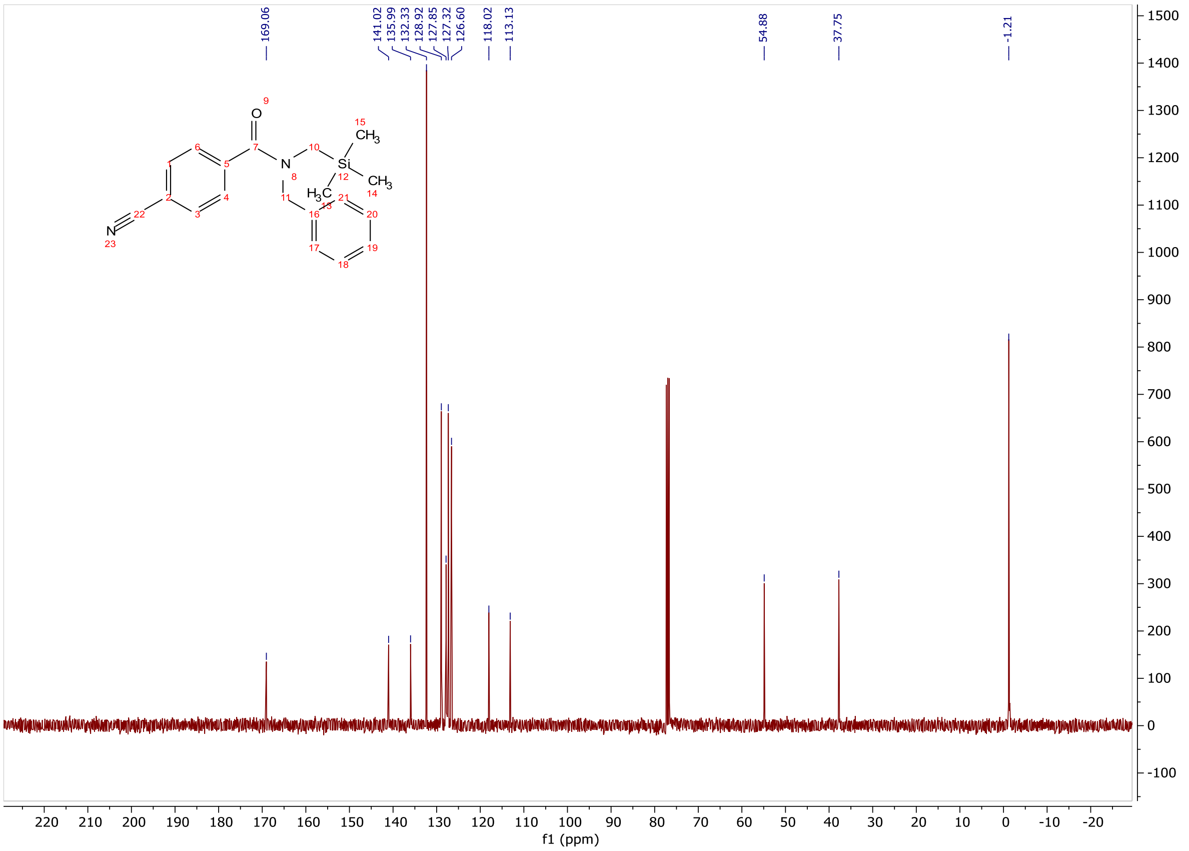

NMR spectra for 4l.

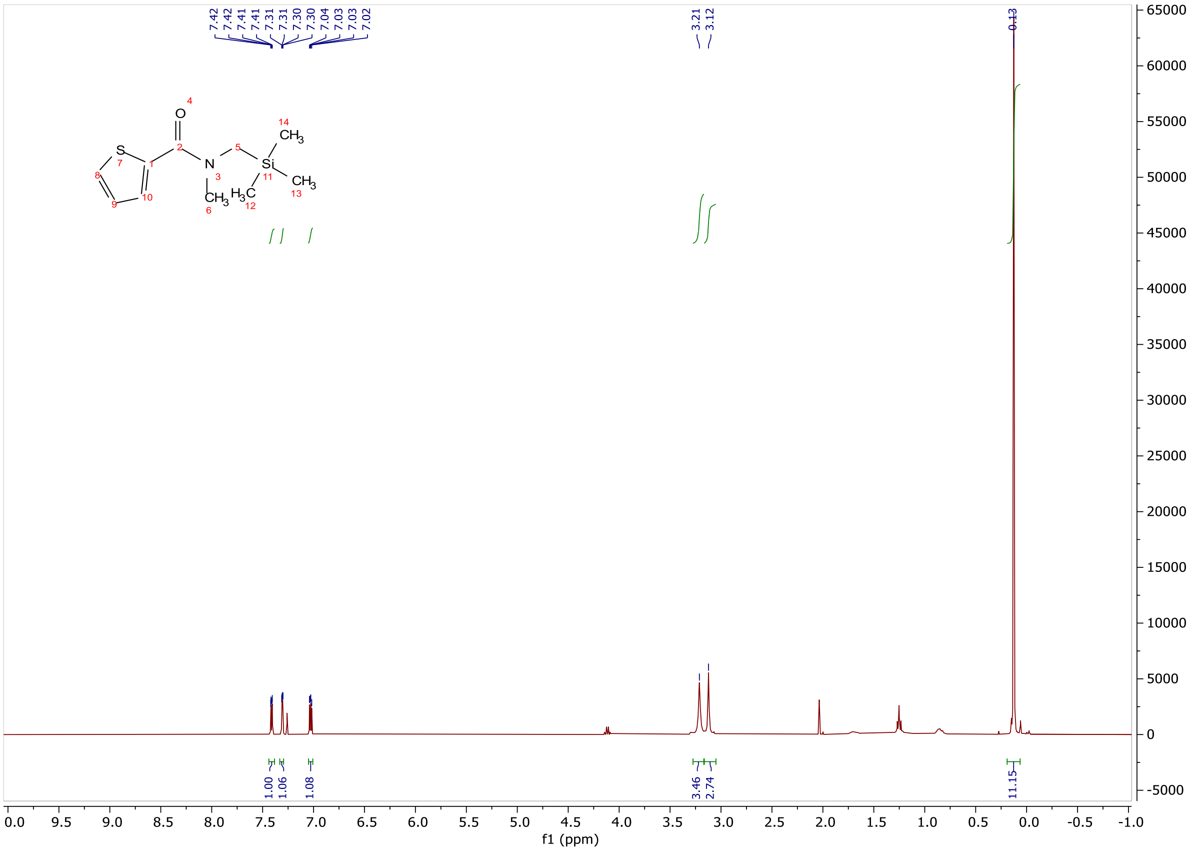

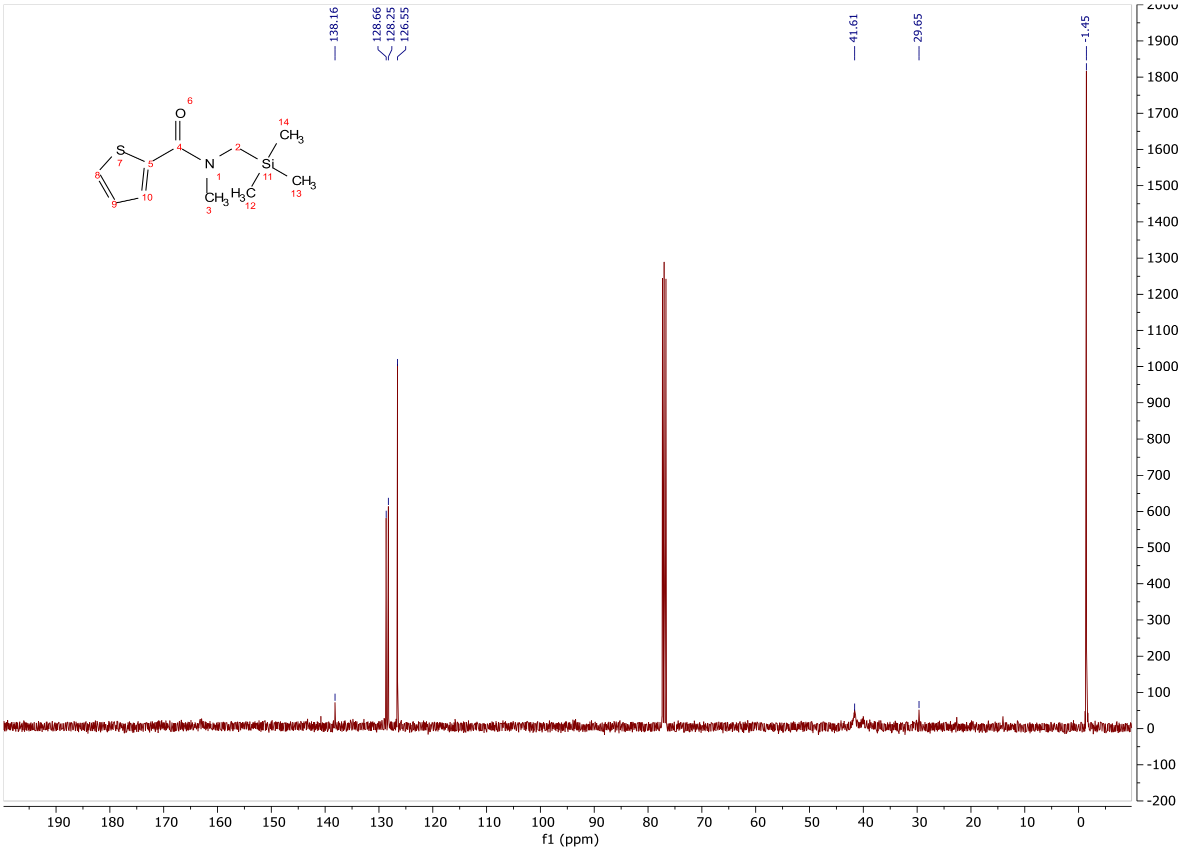

NMR spectra for 4m.

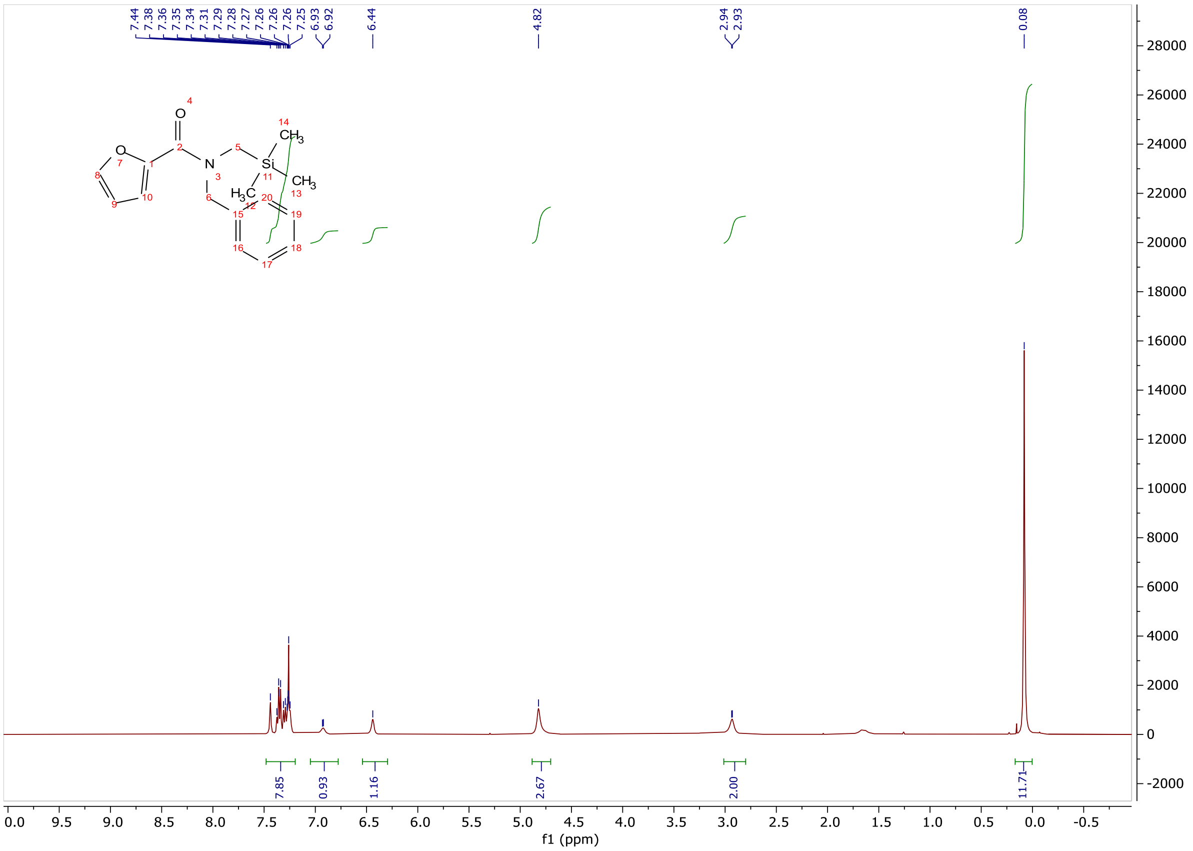

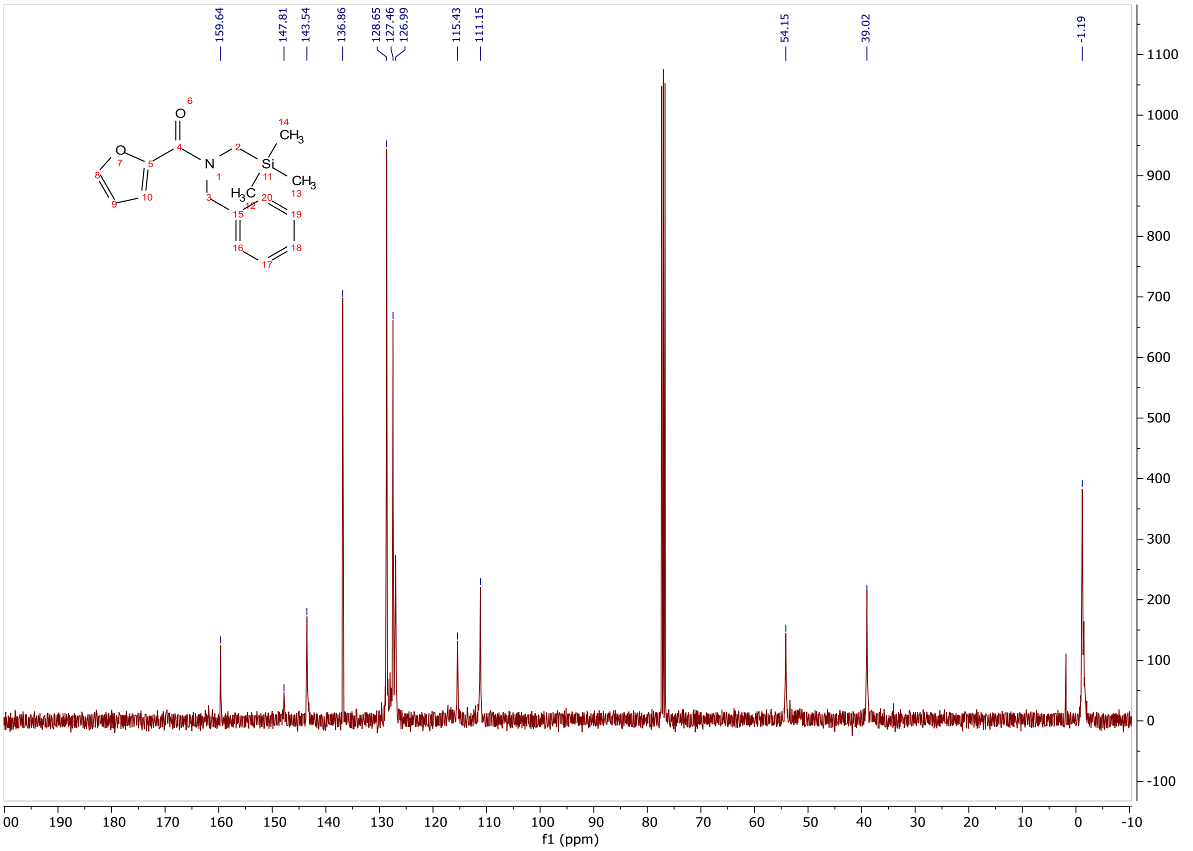

NMR spectra for 4n.

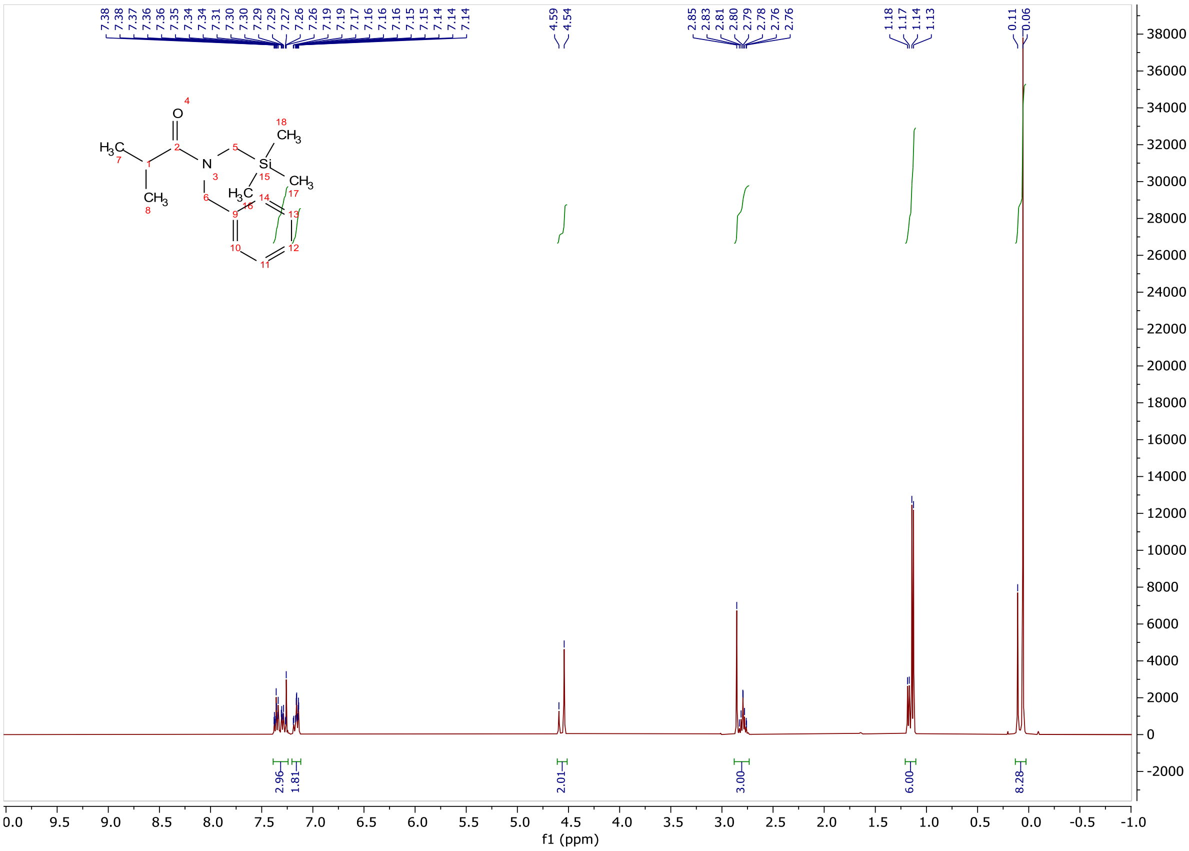

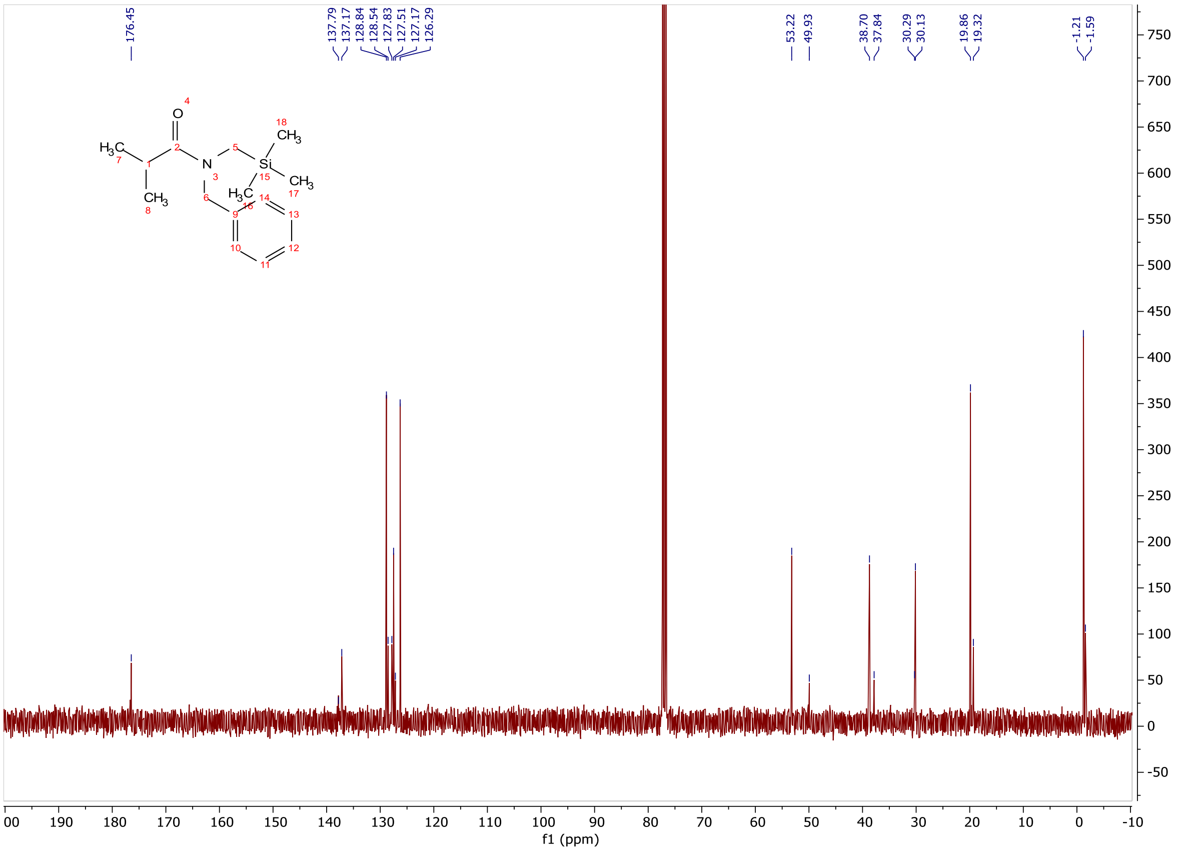

NMR spectra for 3a.

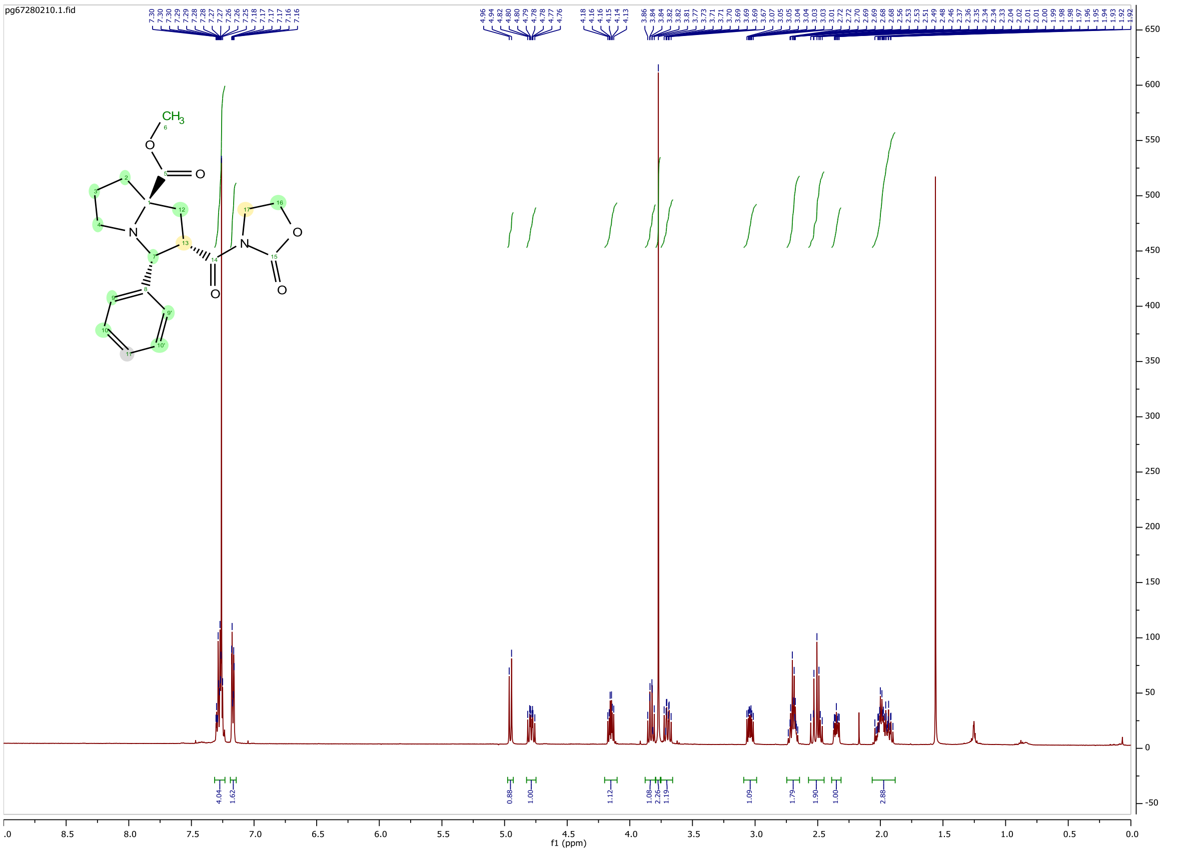

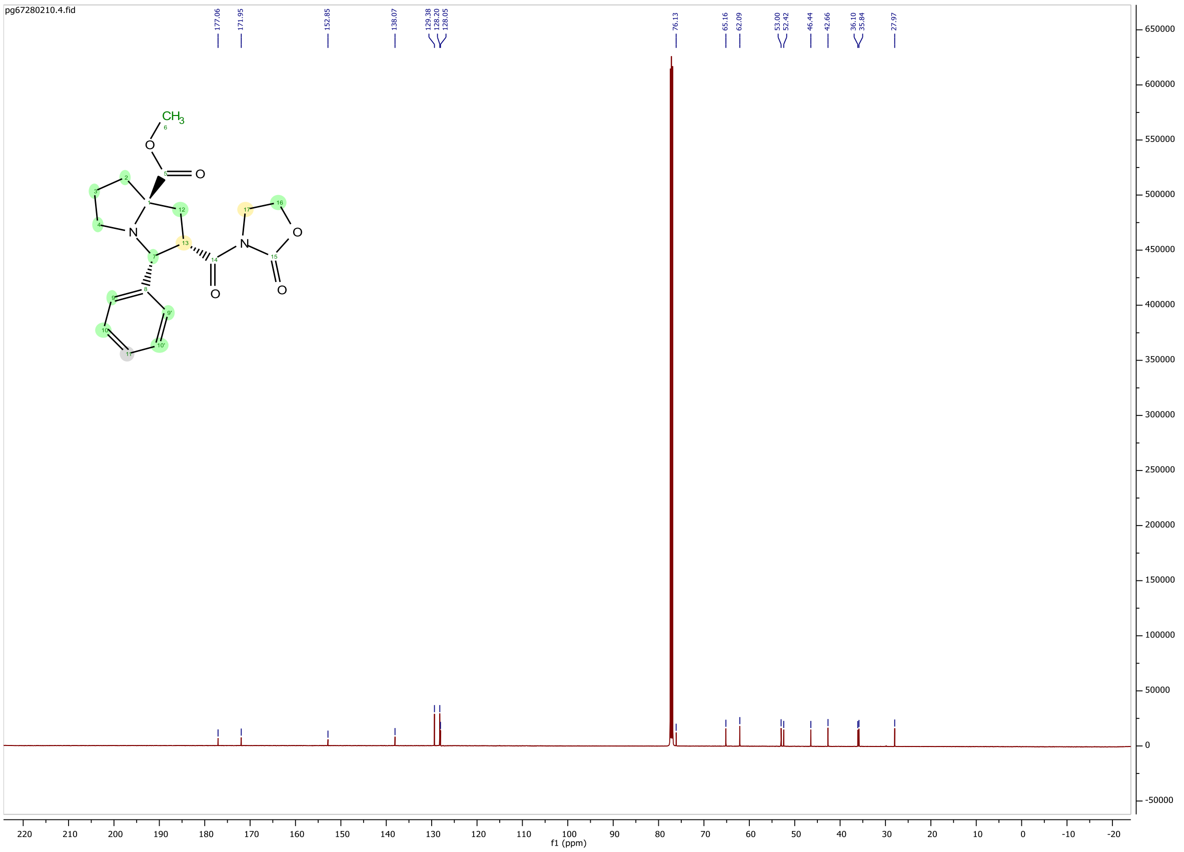

NMR spectra for 3b.

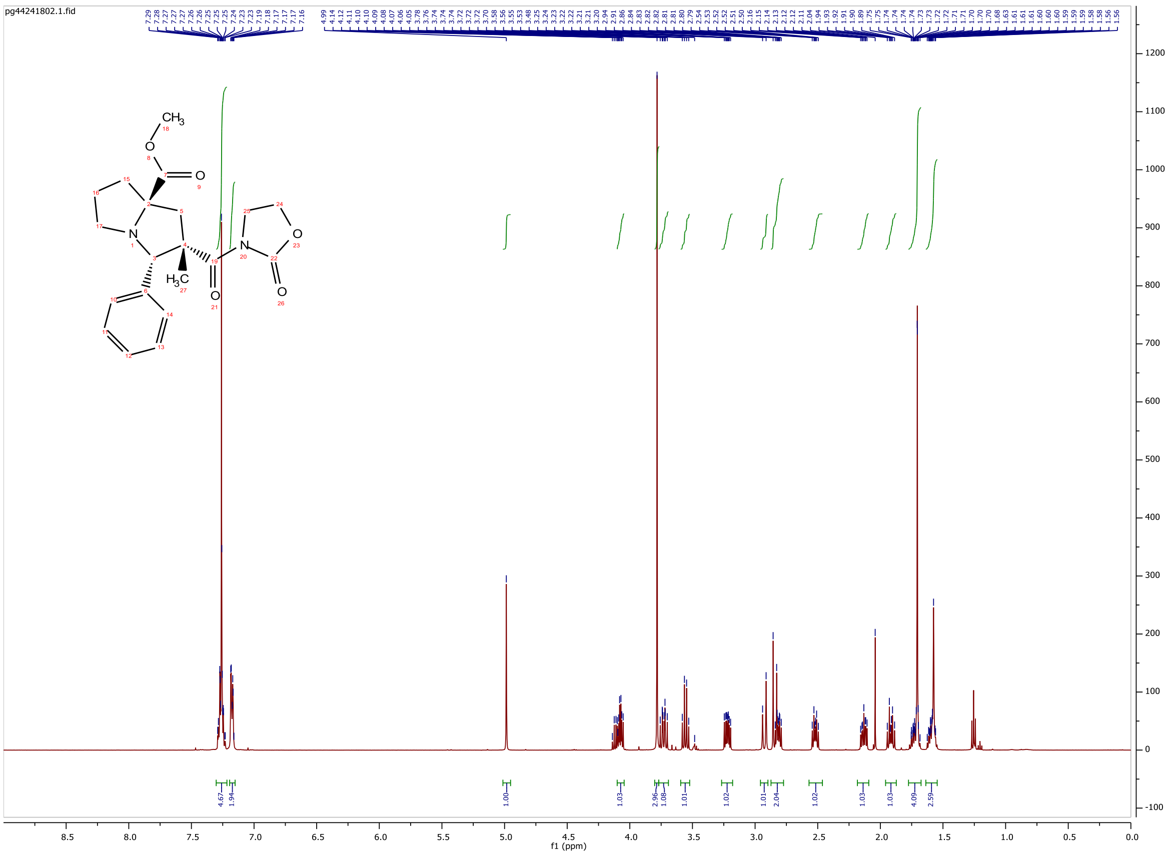

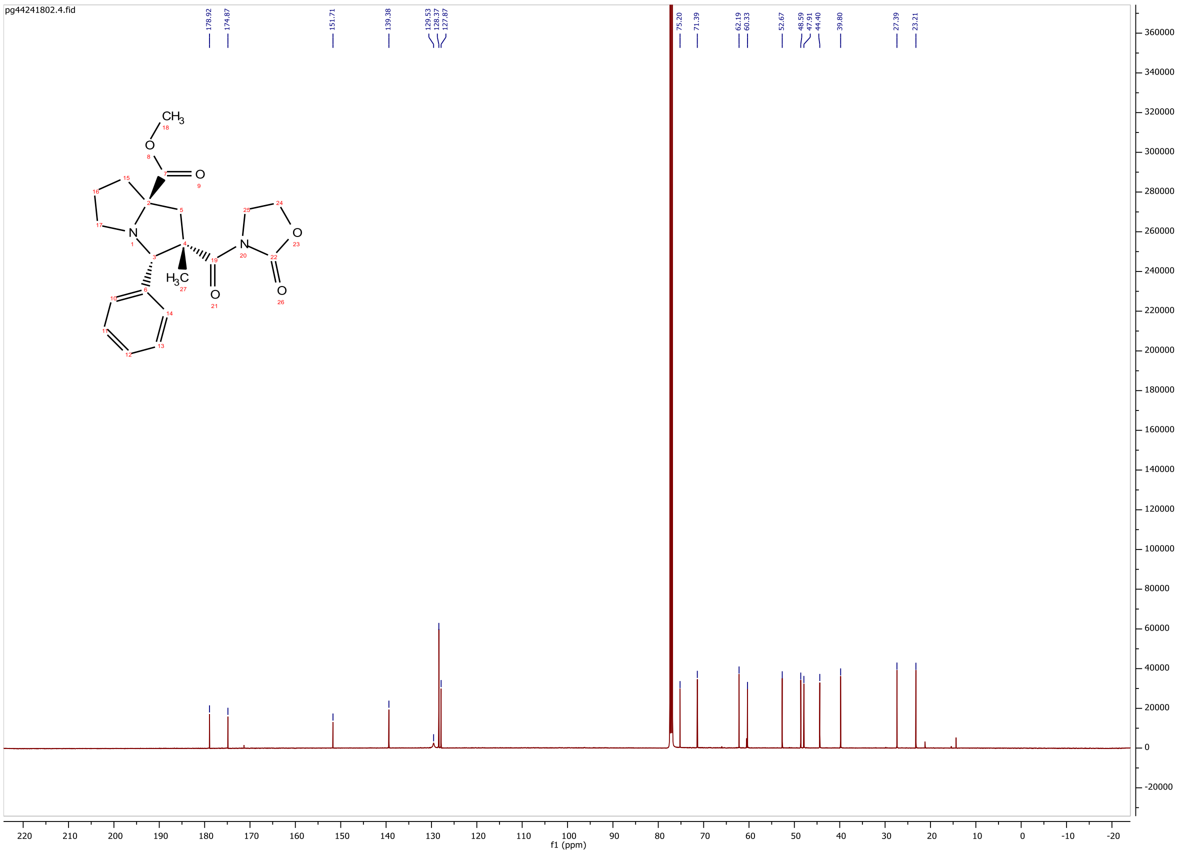

NMR spectra for 3c.

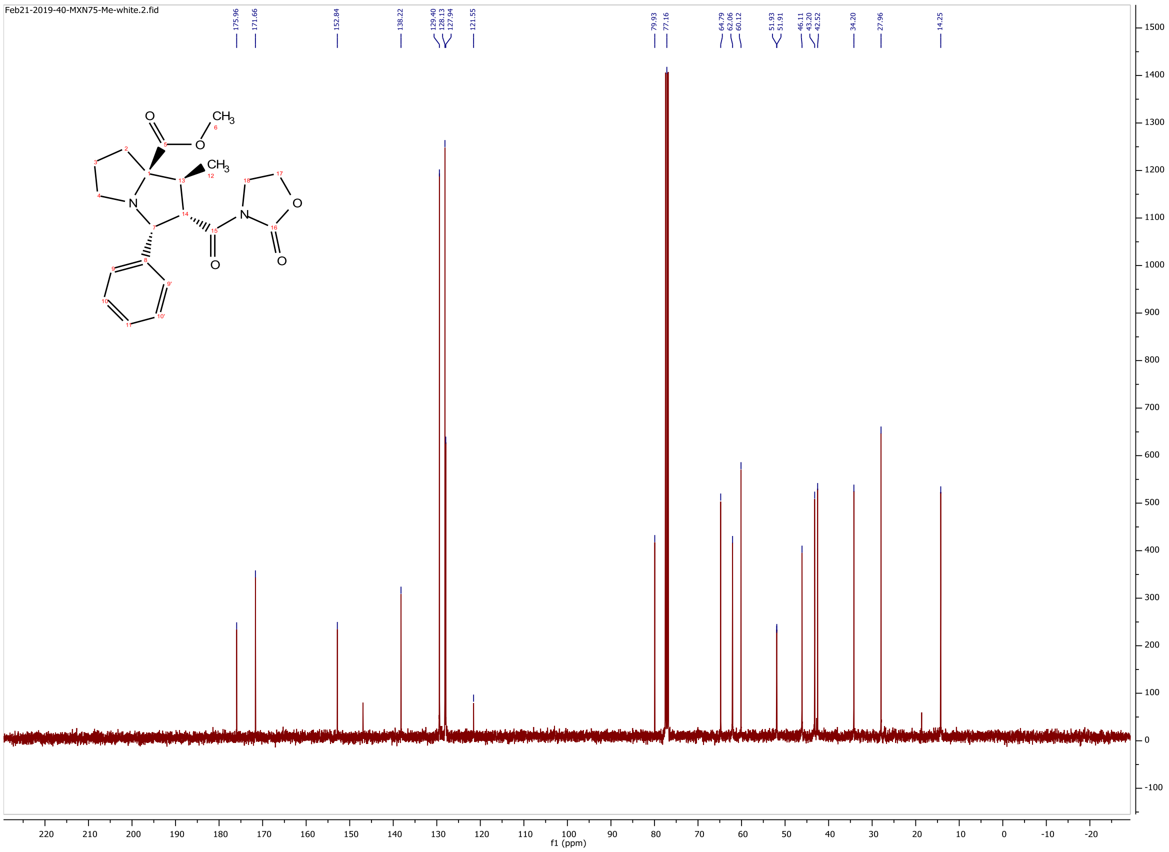

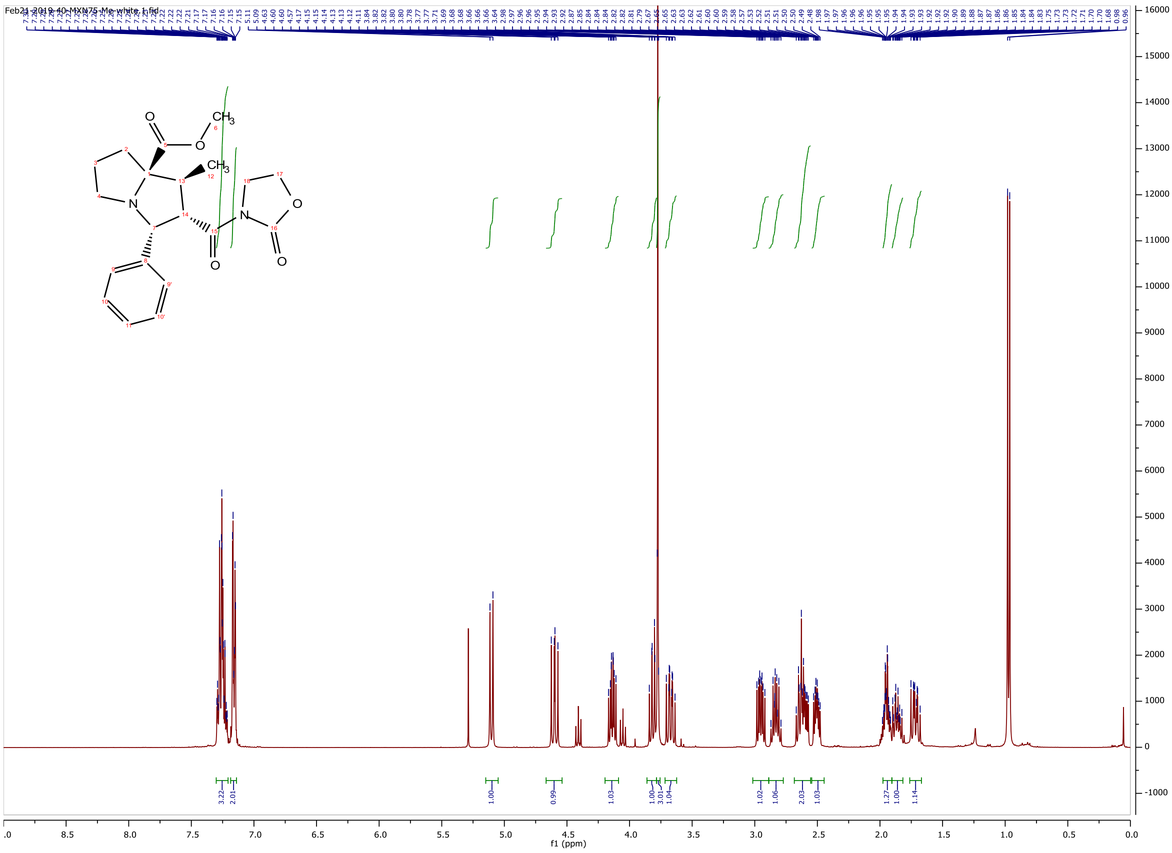

NMR spectra for 3d.

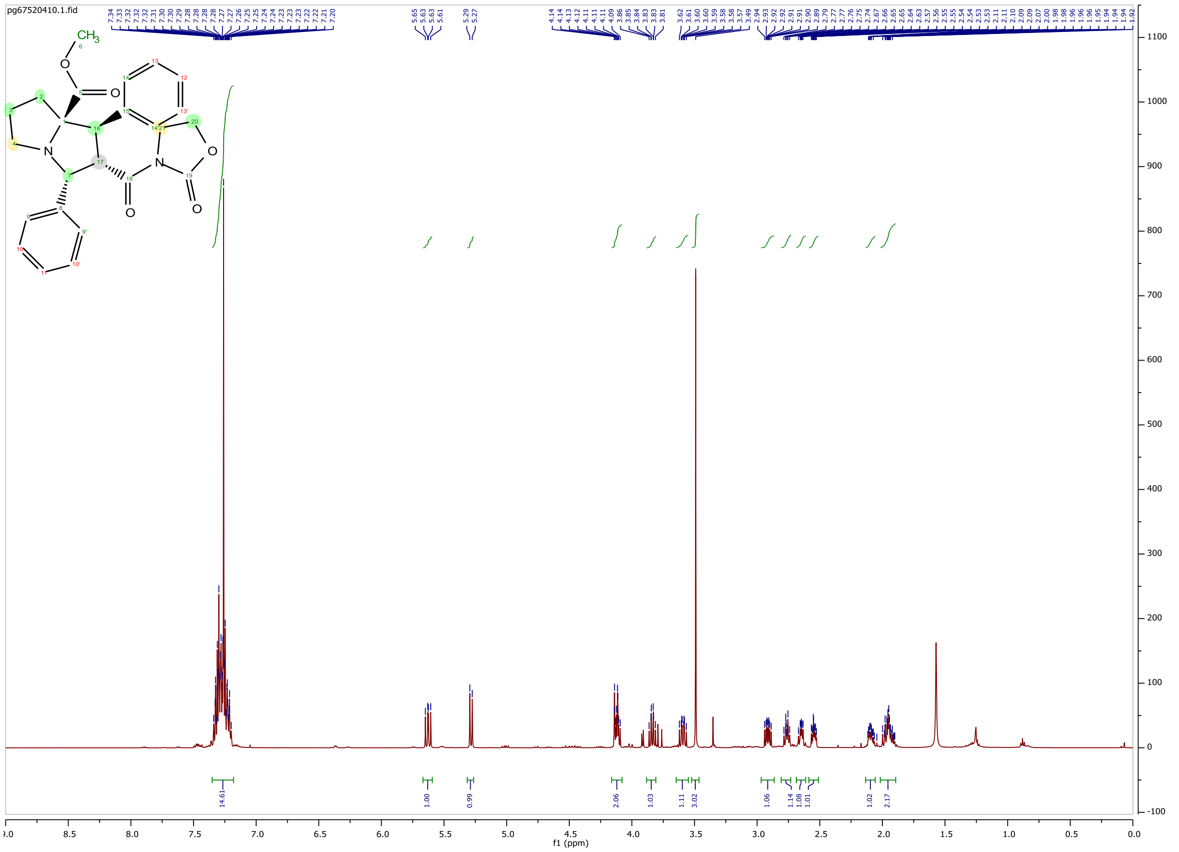

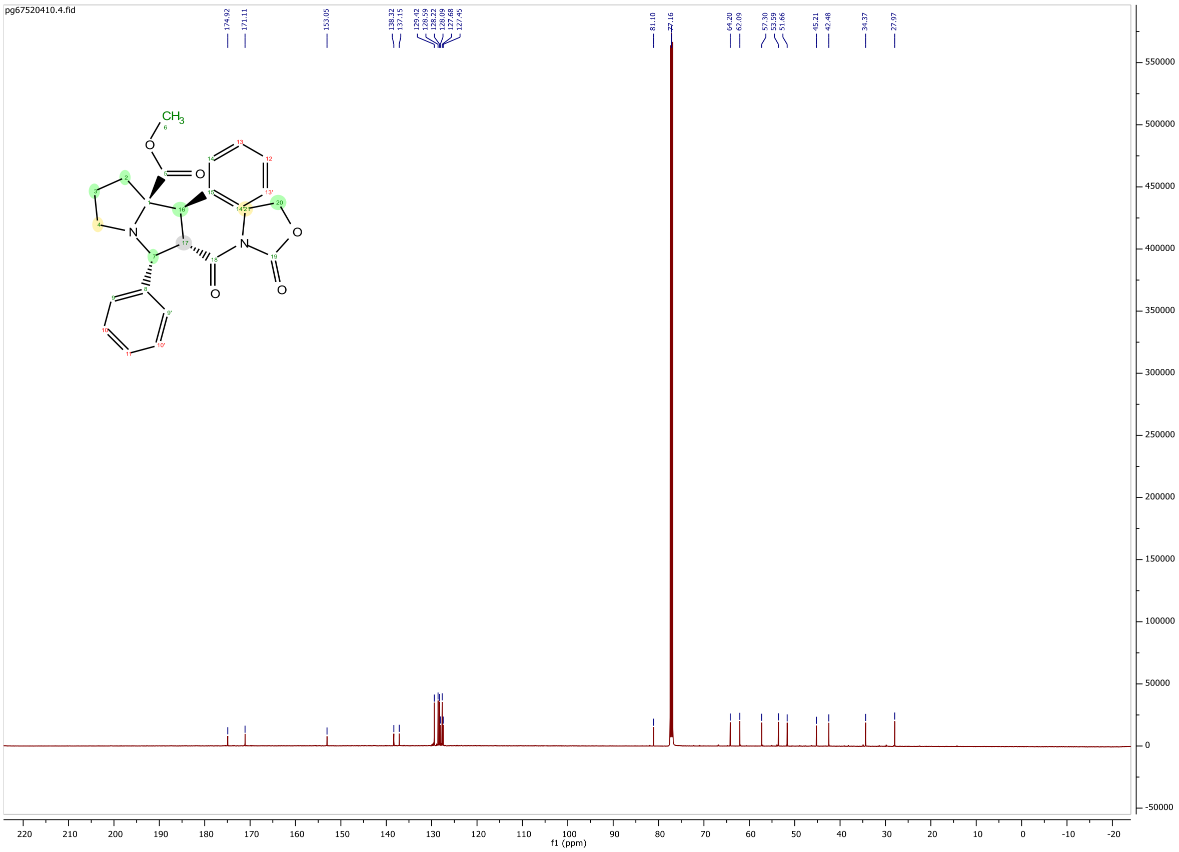

NMR spectra for 3e.

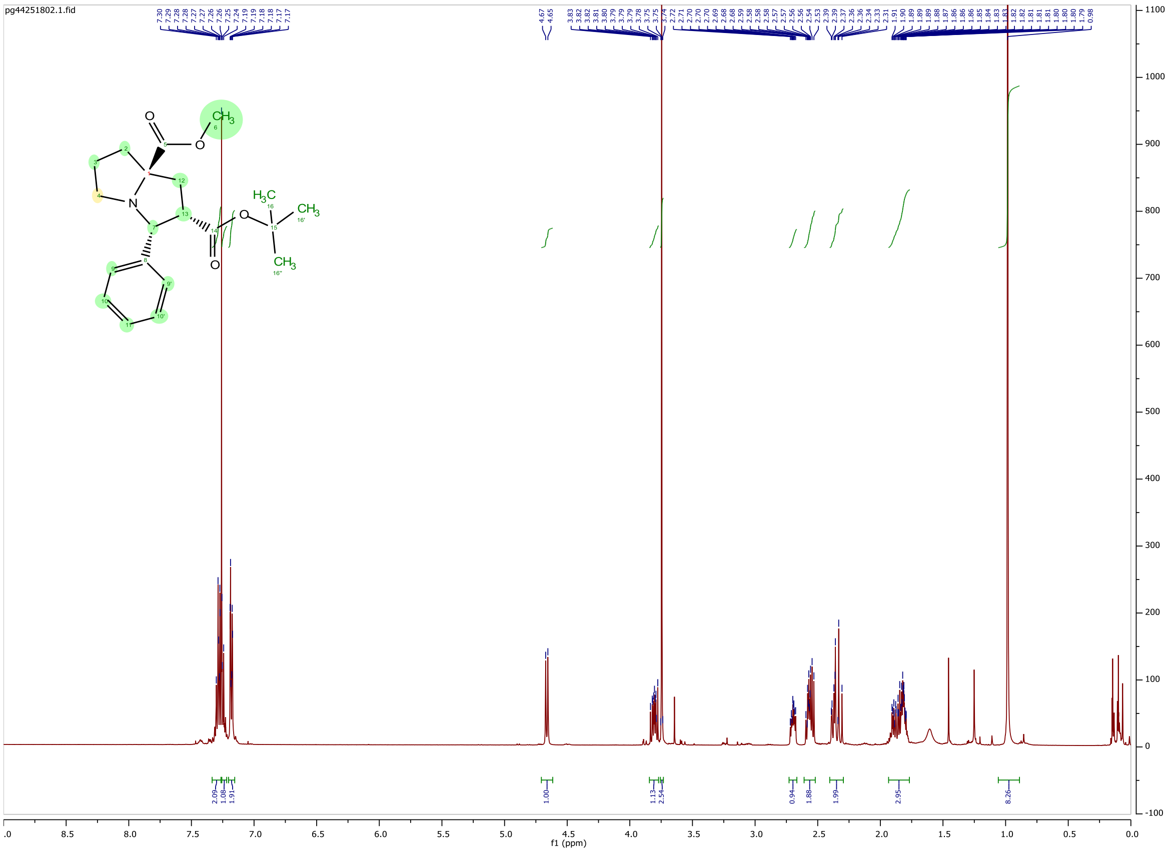

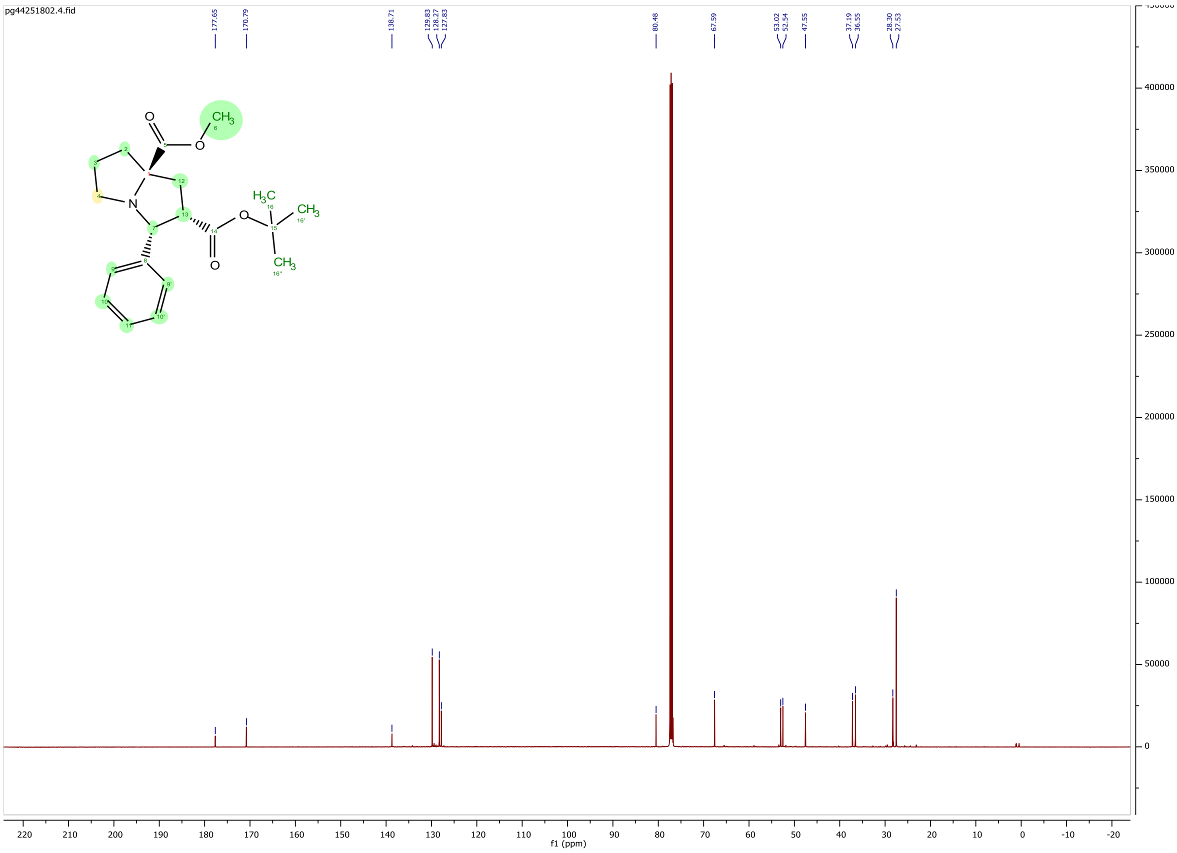

NMR spectra for 3f.

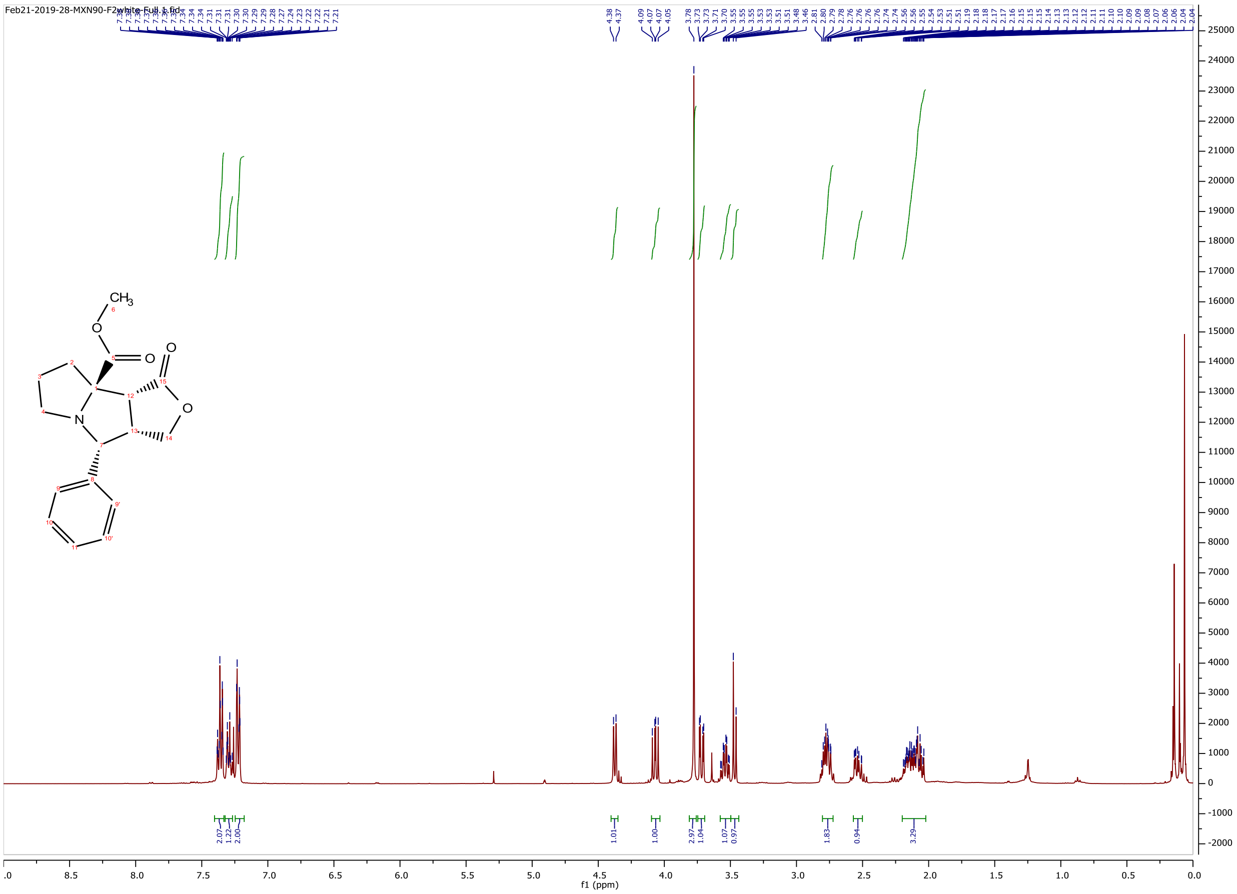

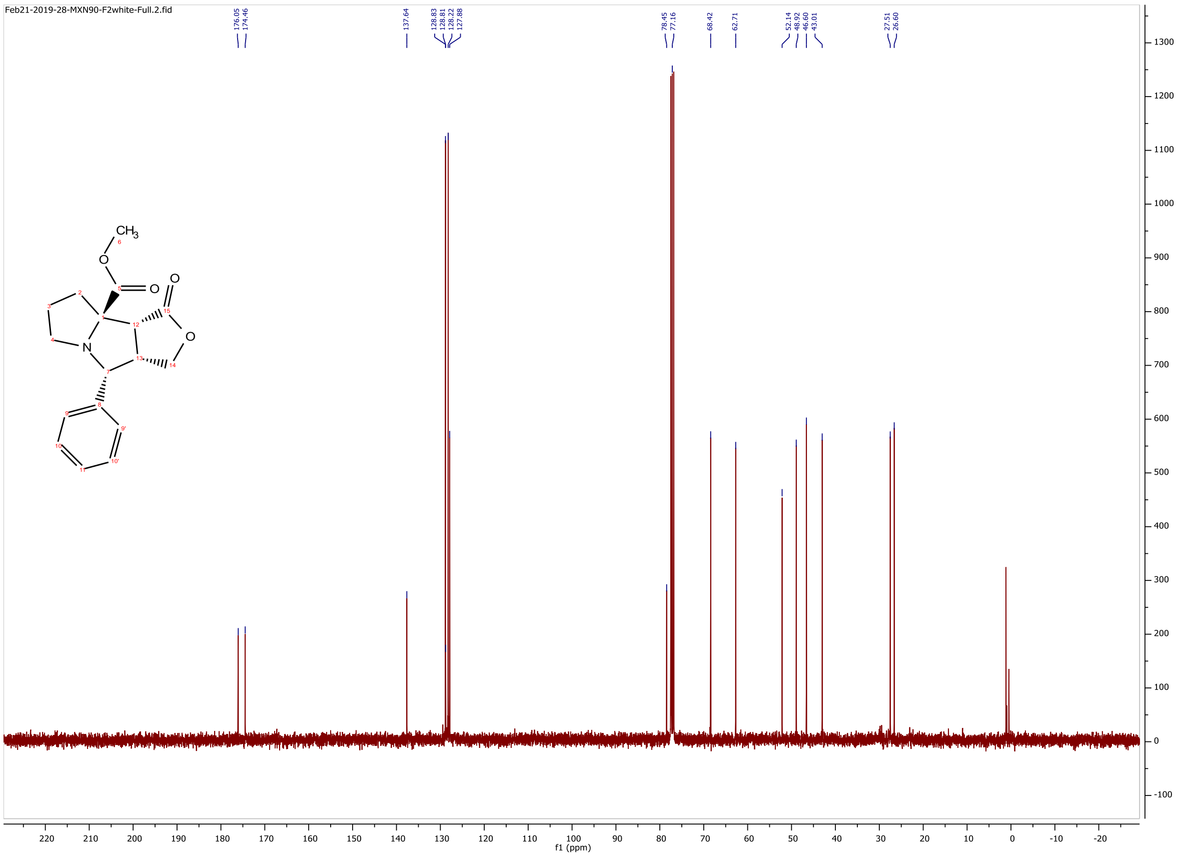

NMR spectra for 3h.

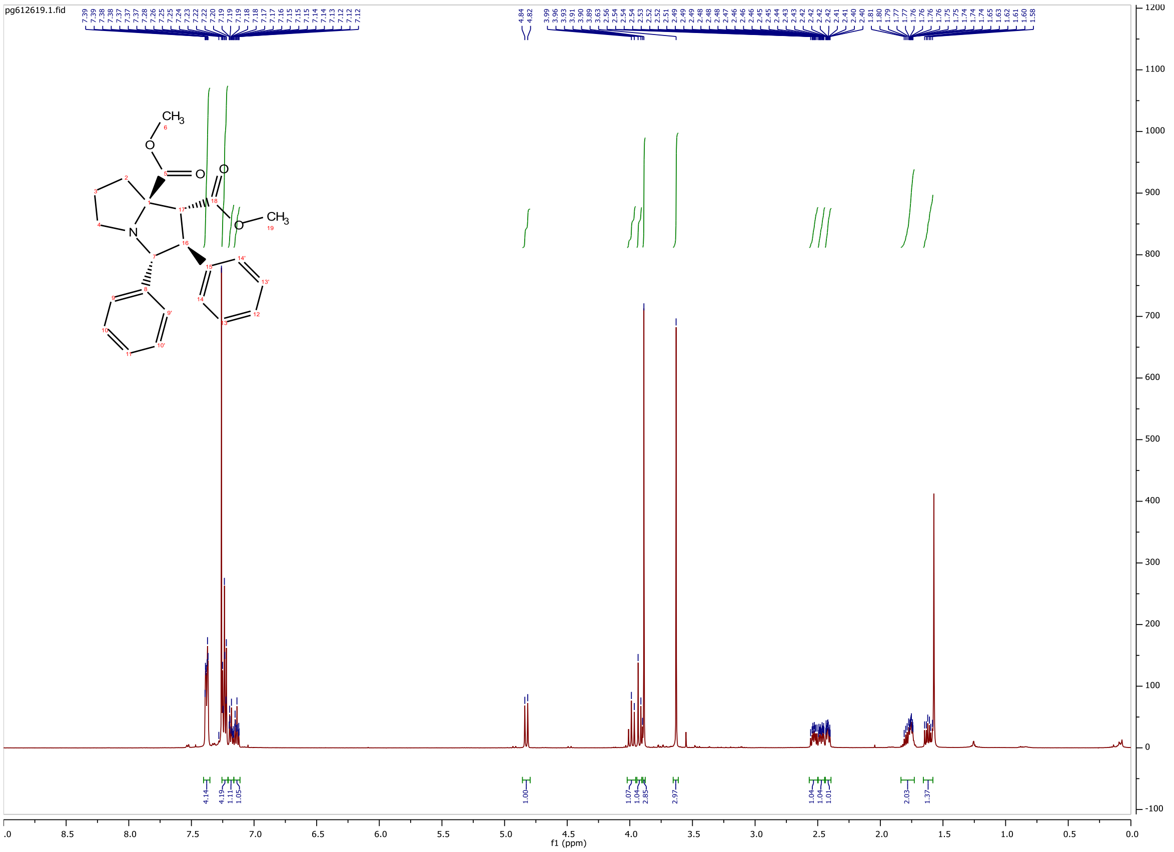

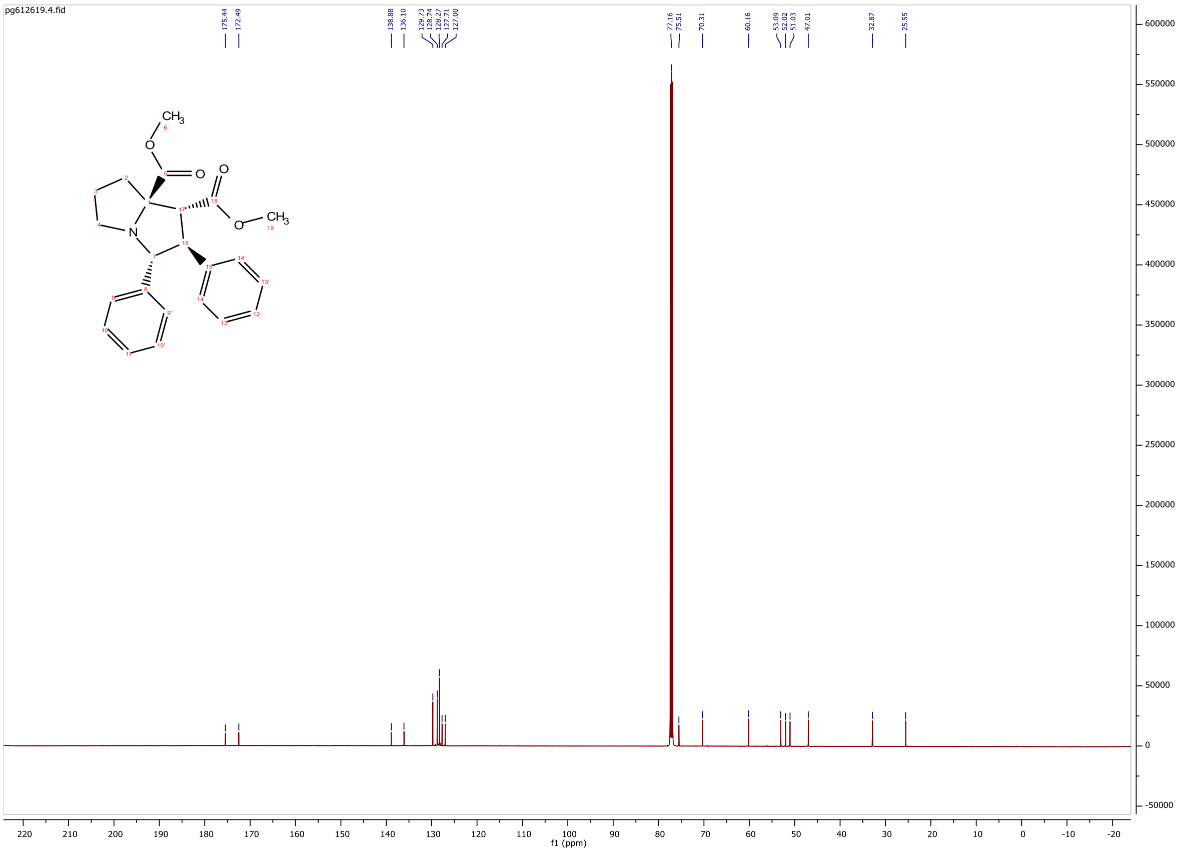

NMR spectra for 3i1 and 3i2.

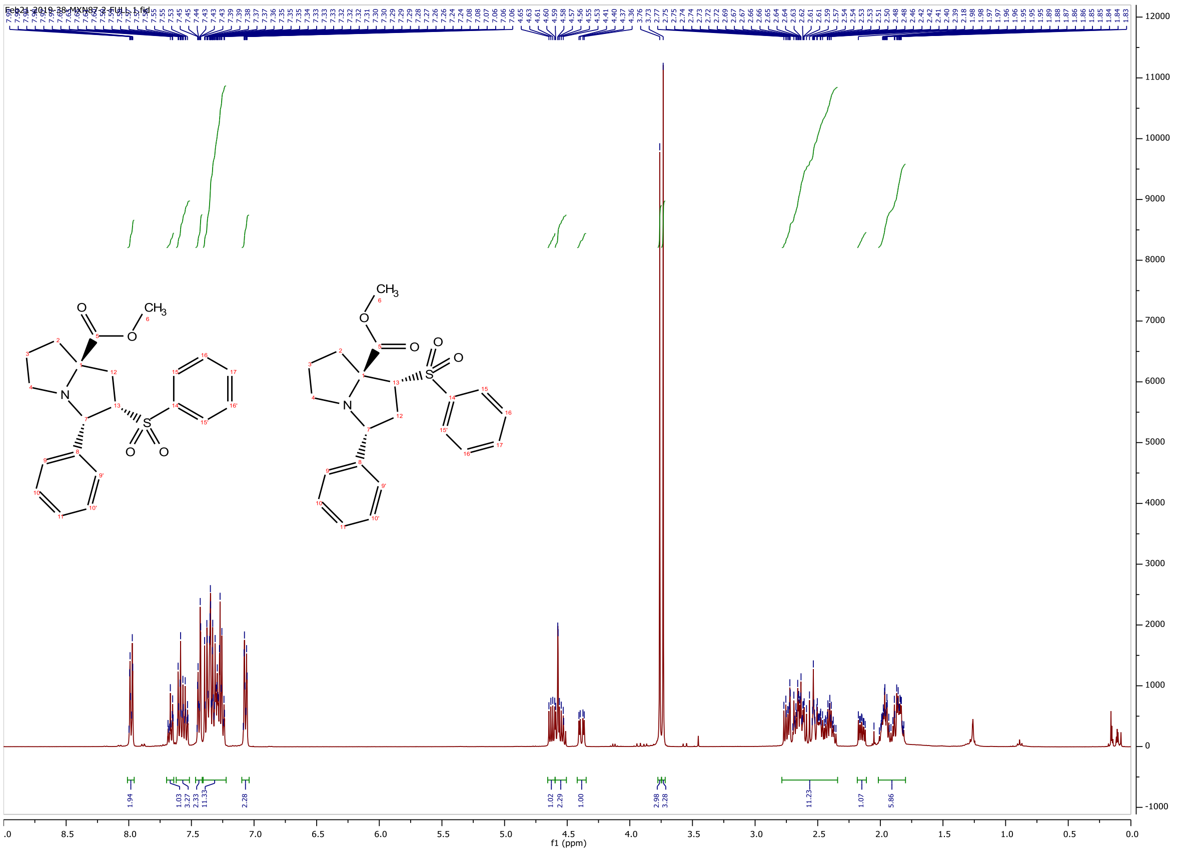

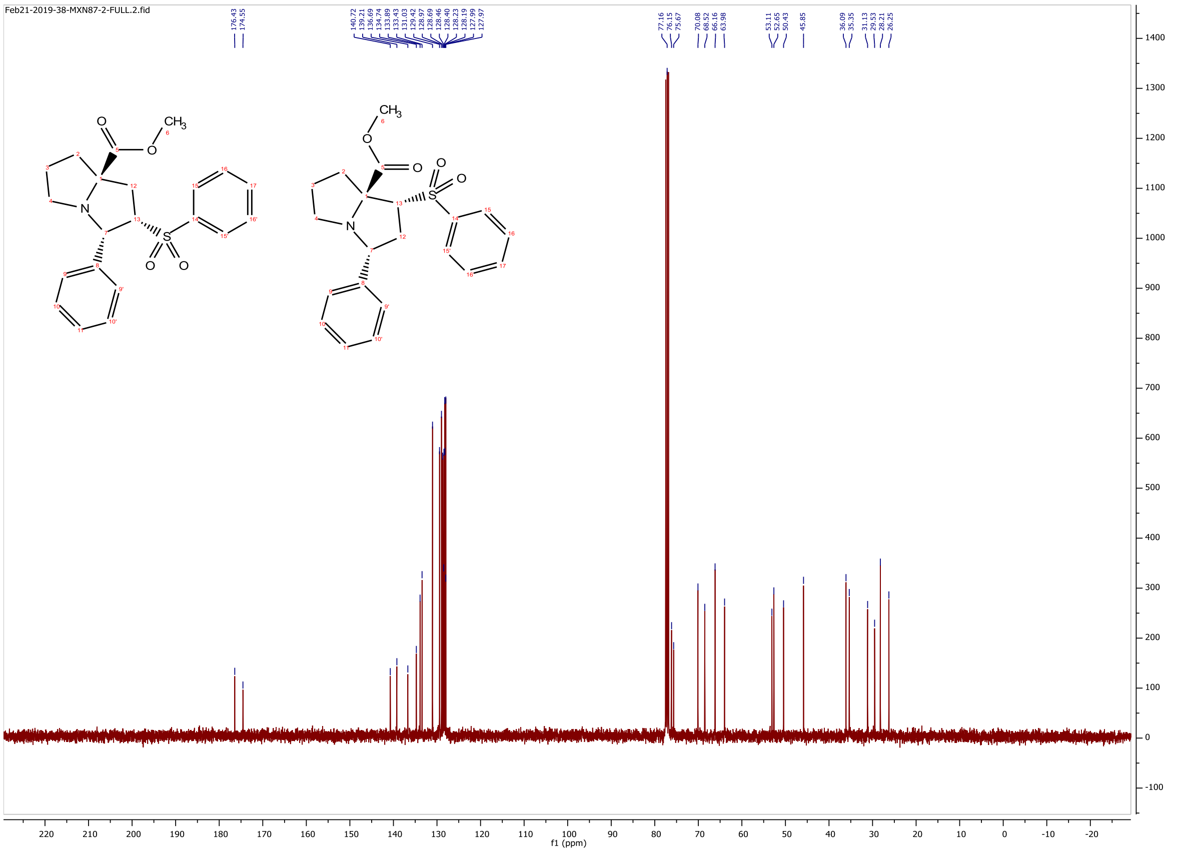

NMR spectra for 3j.

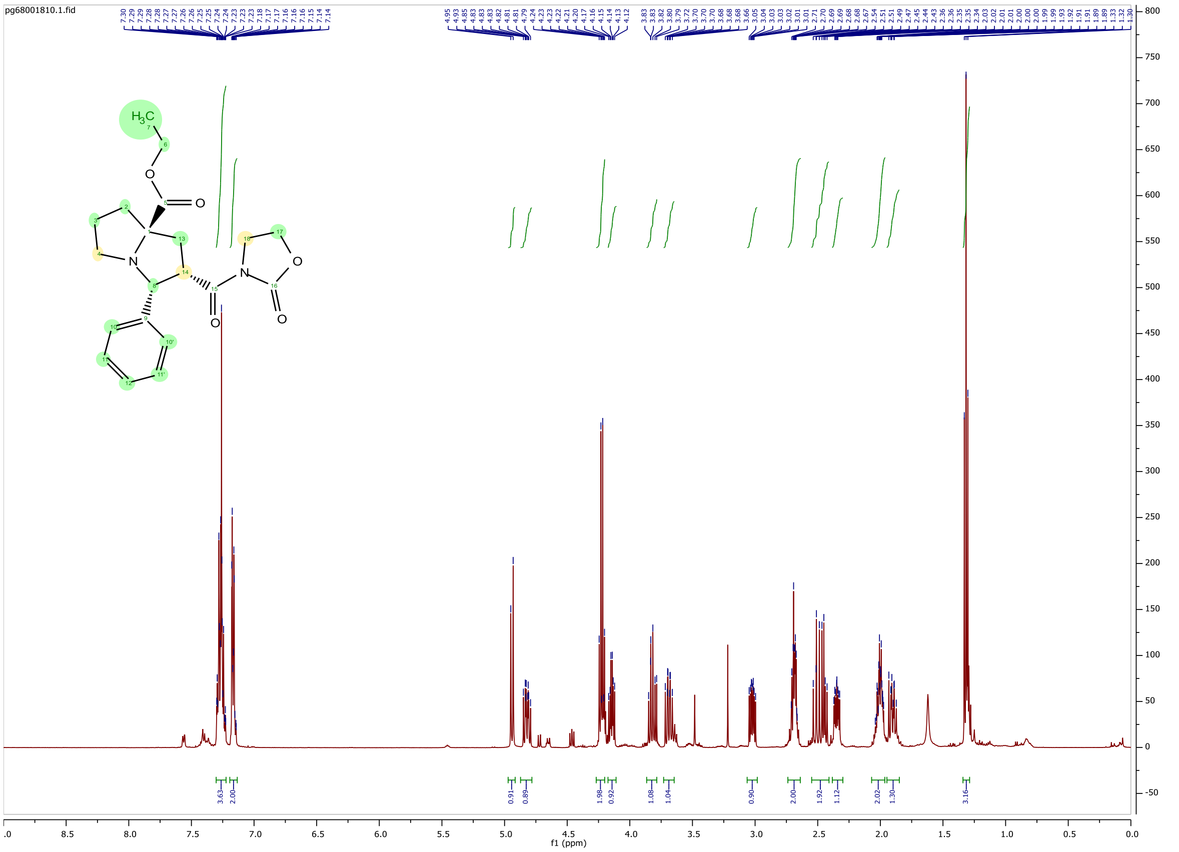

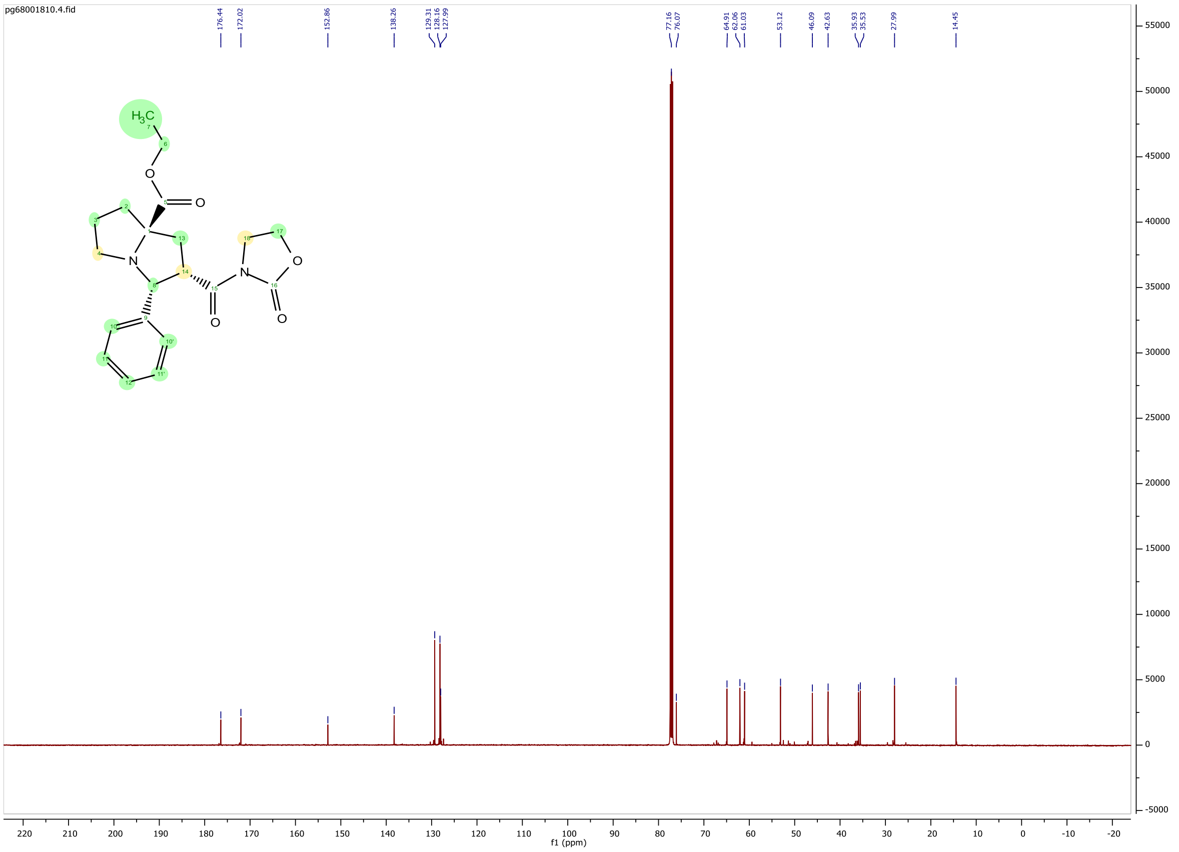

NMR spectra for 3k.

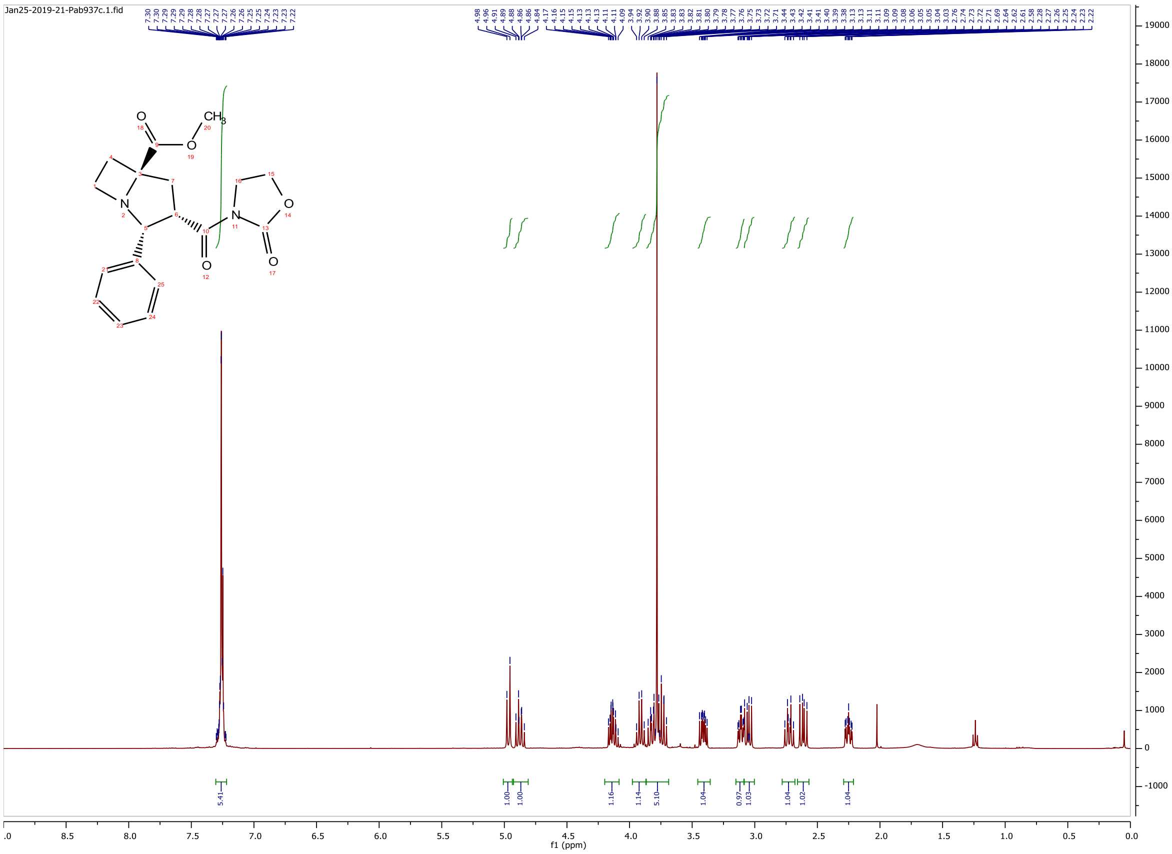

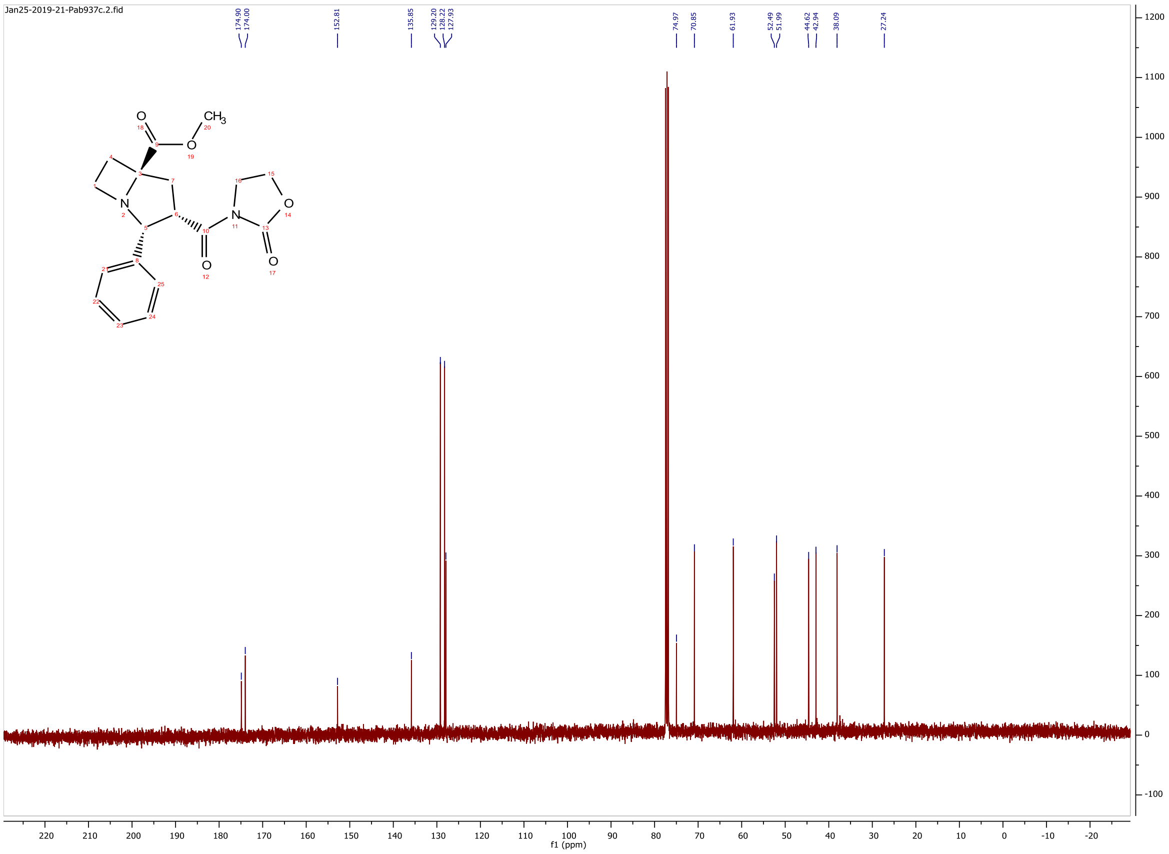

NMR spectra for 311.

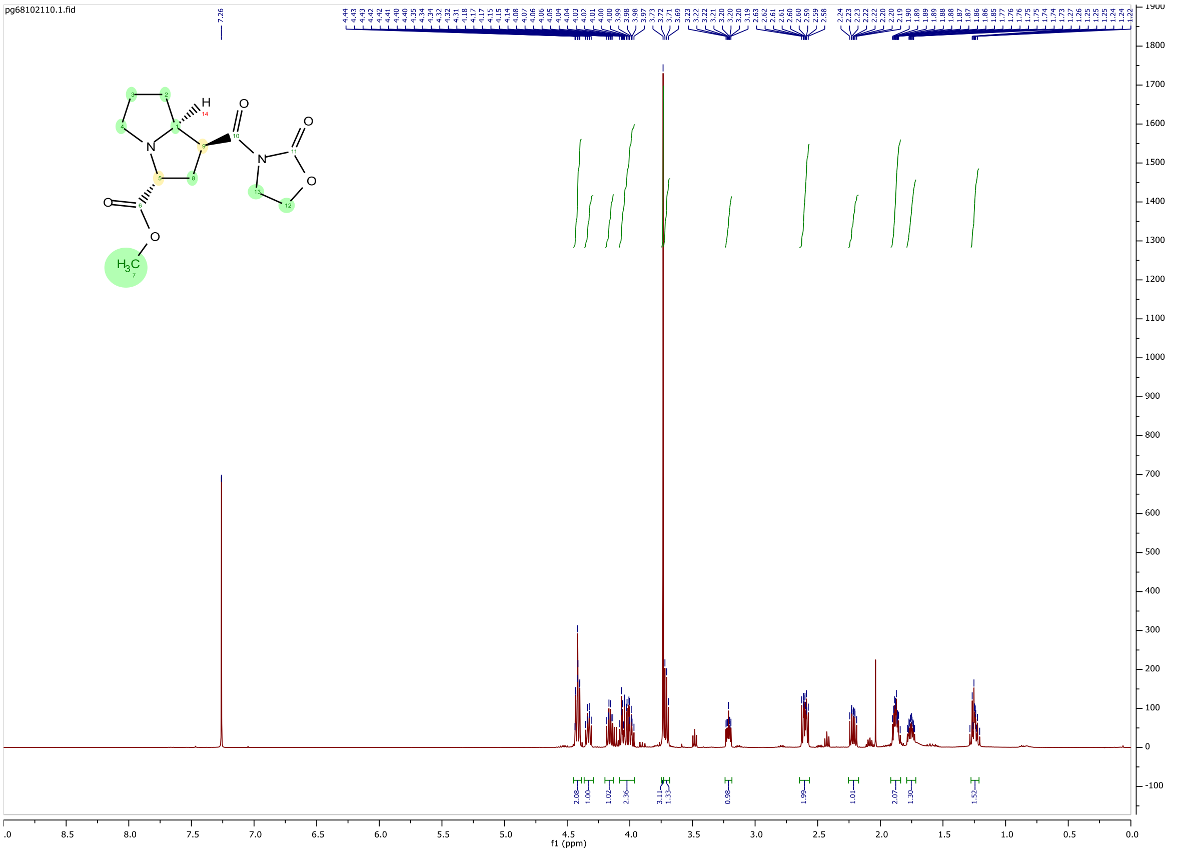

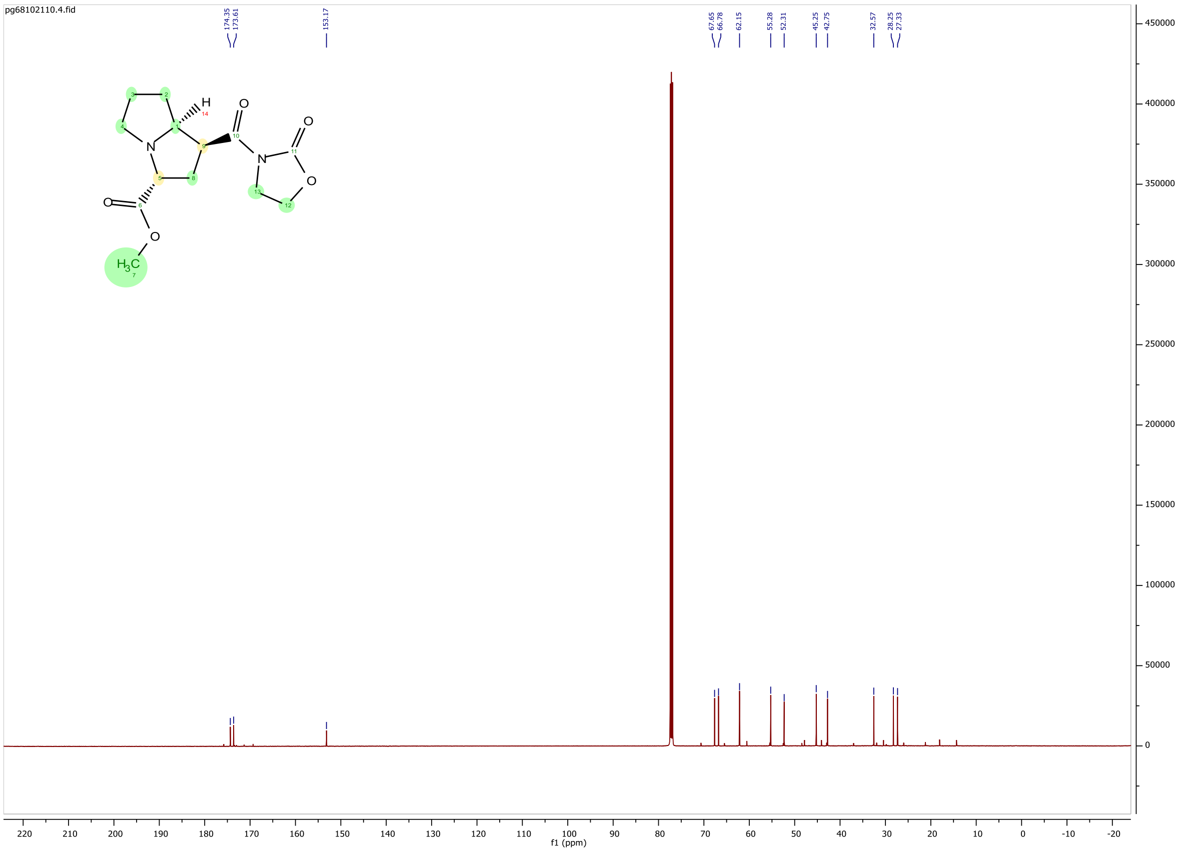

NMR spectra for 312.

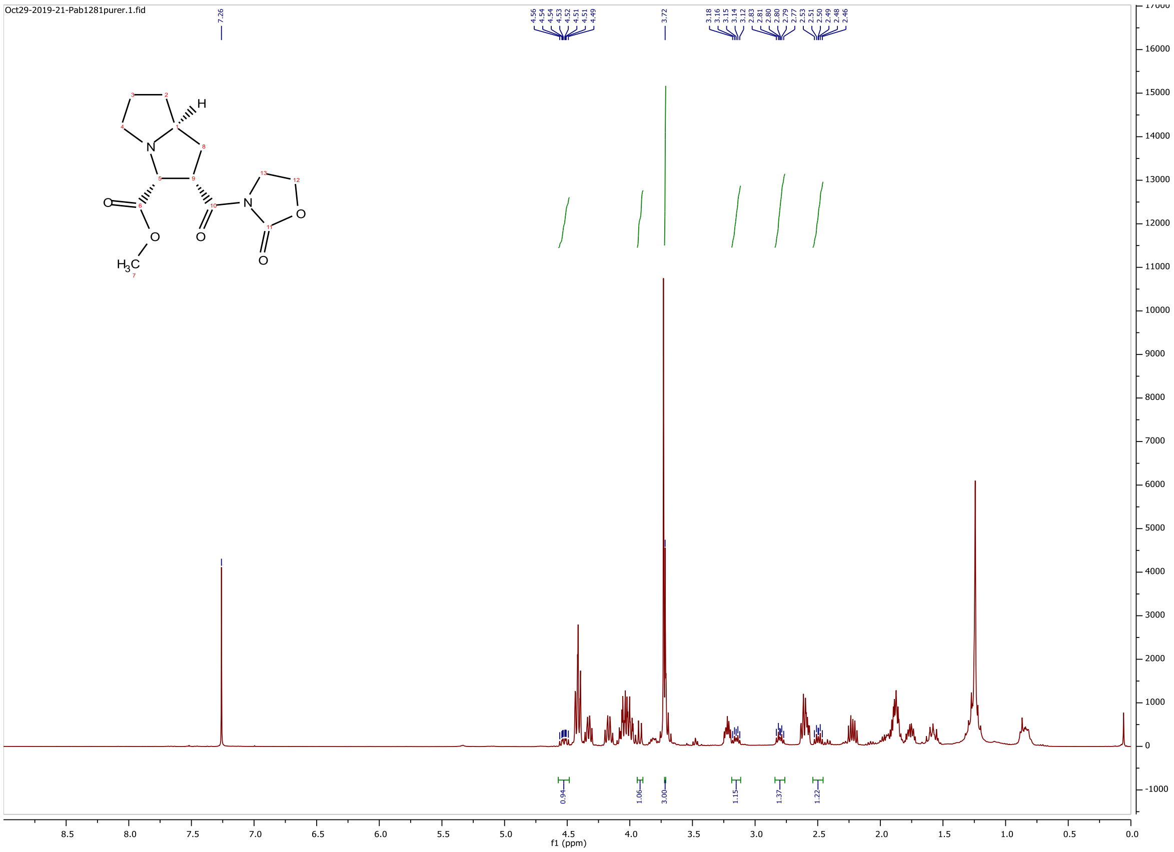

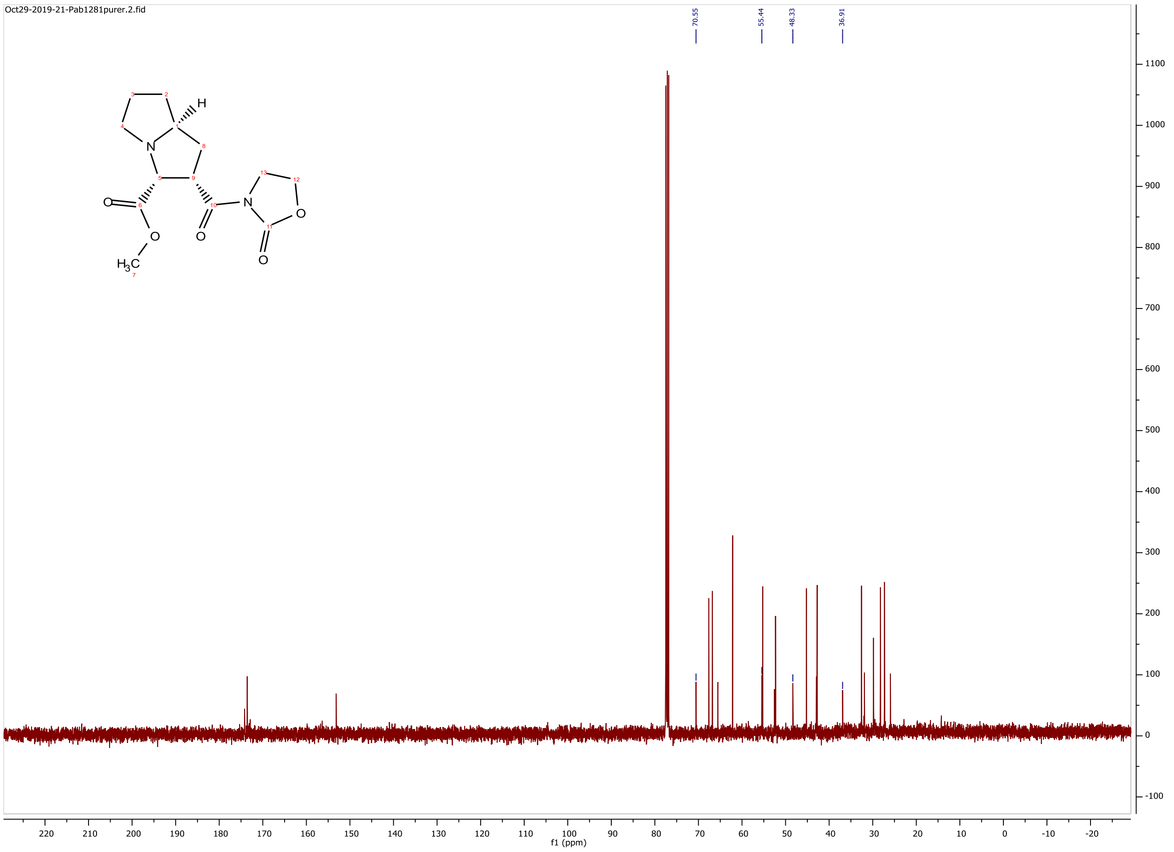

NMR spectra for 3m1 and 3m2.

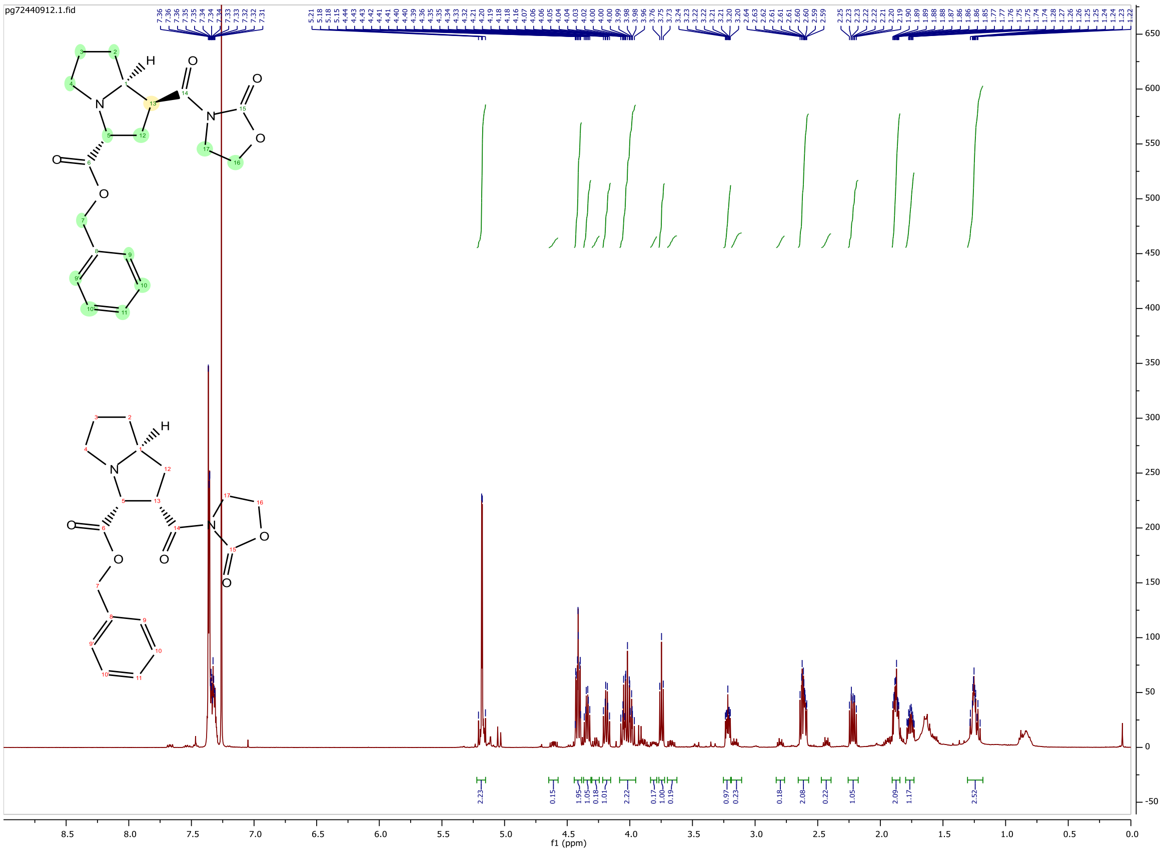

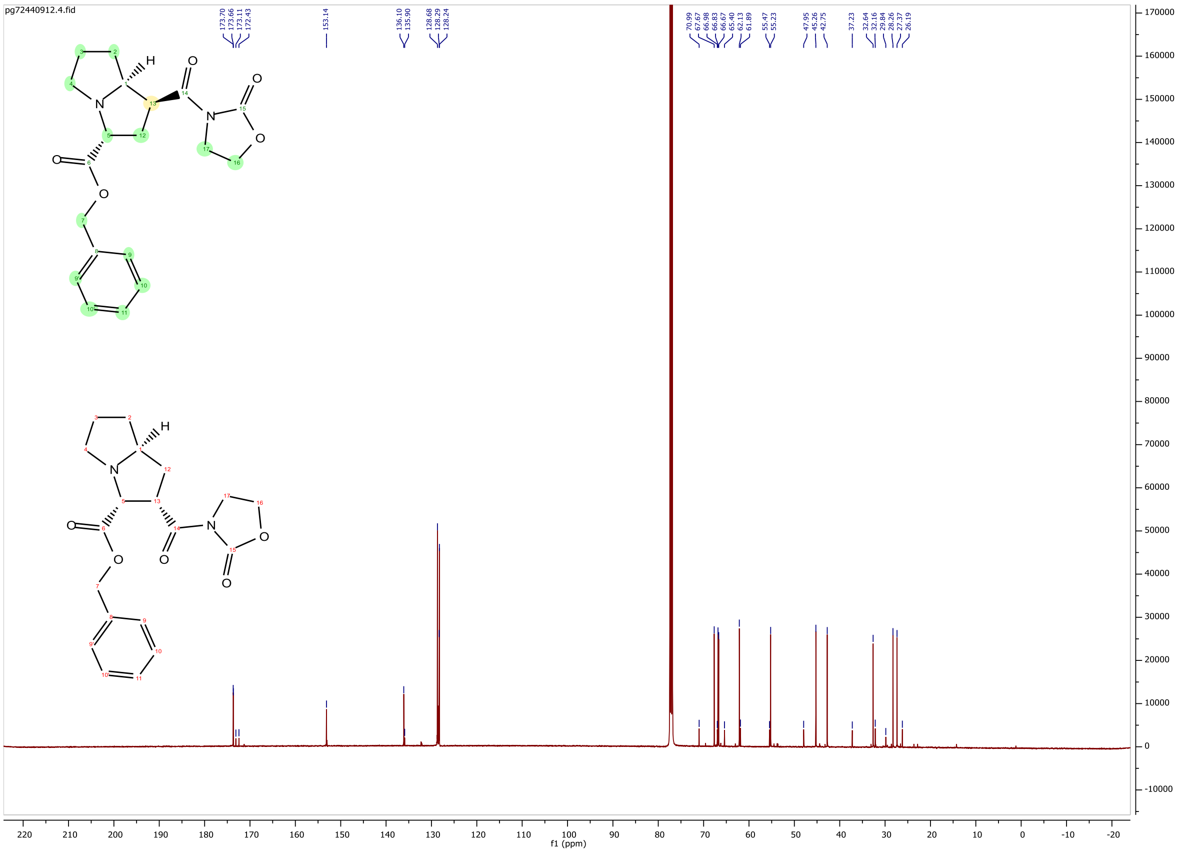

NMR spectra for 3n1 and 3n2.

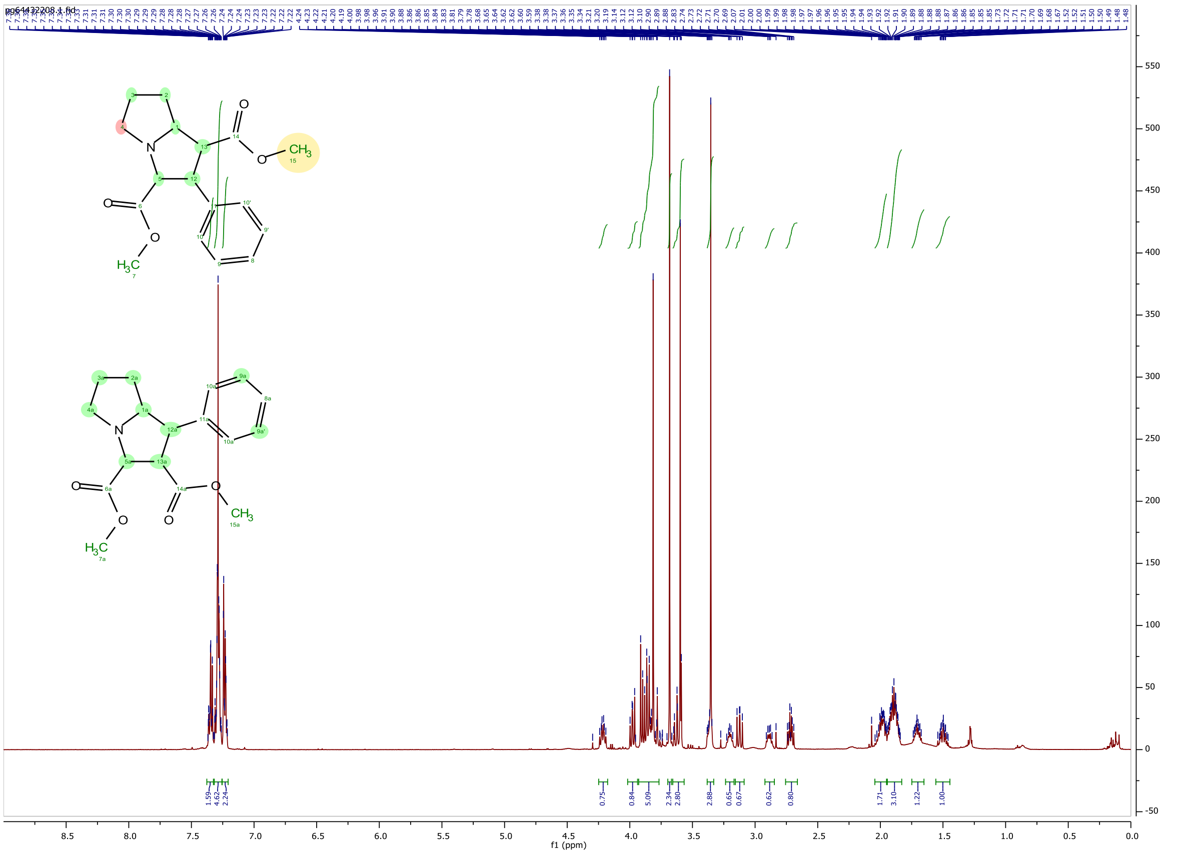

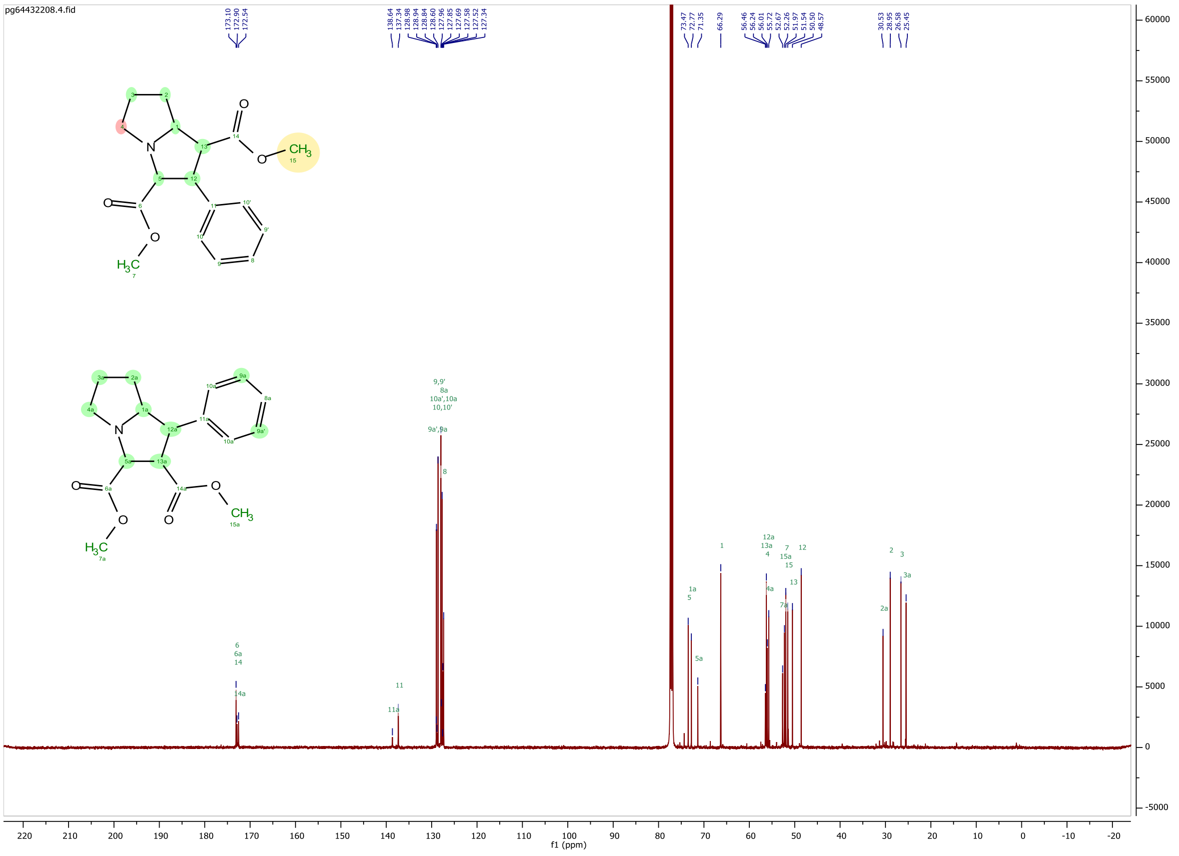

NMR spectra for 3o.

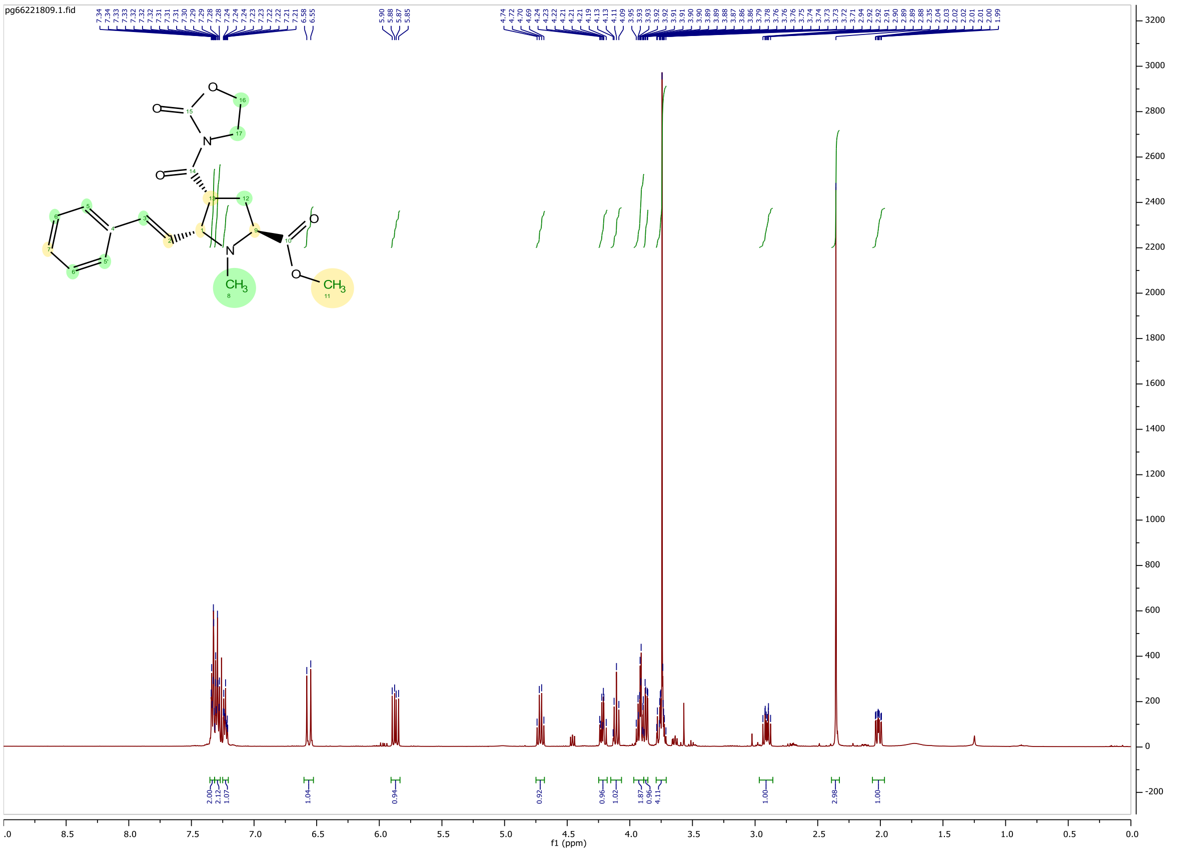

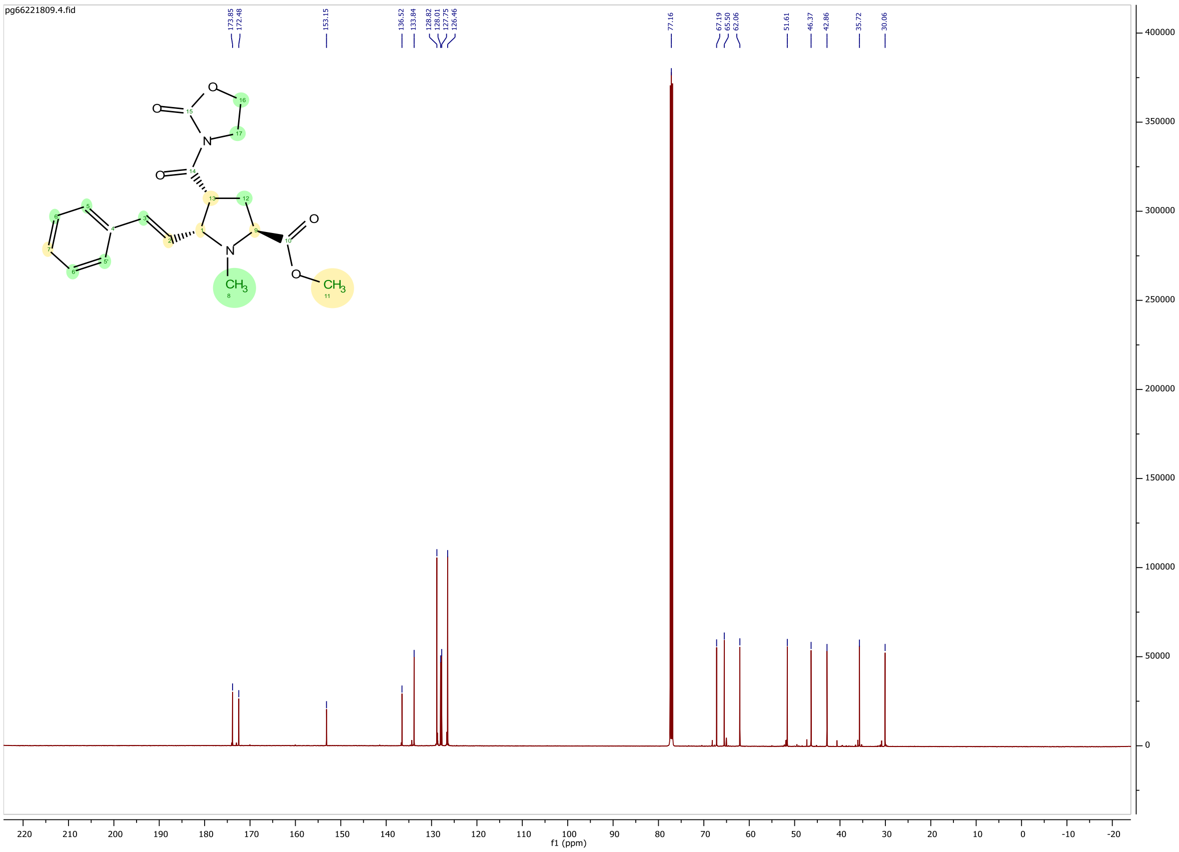

NMR spectra for 5a.

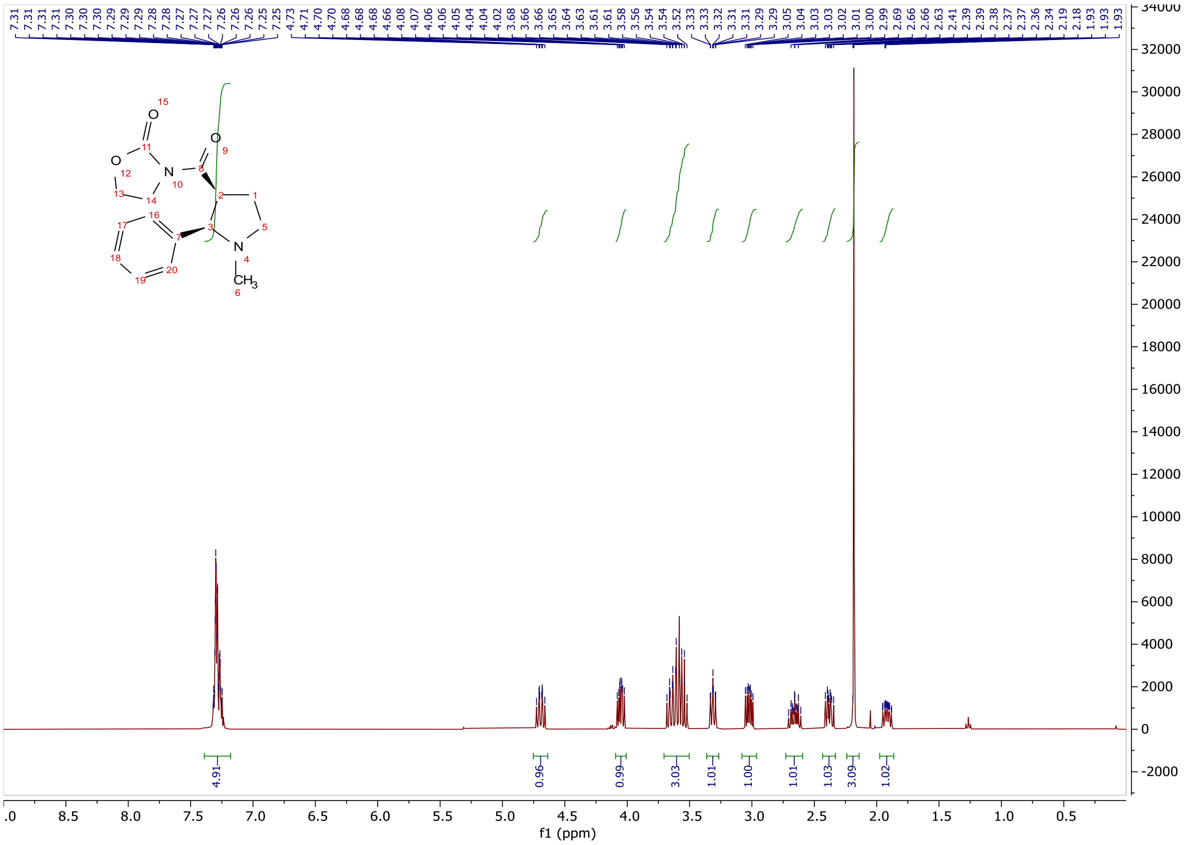

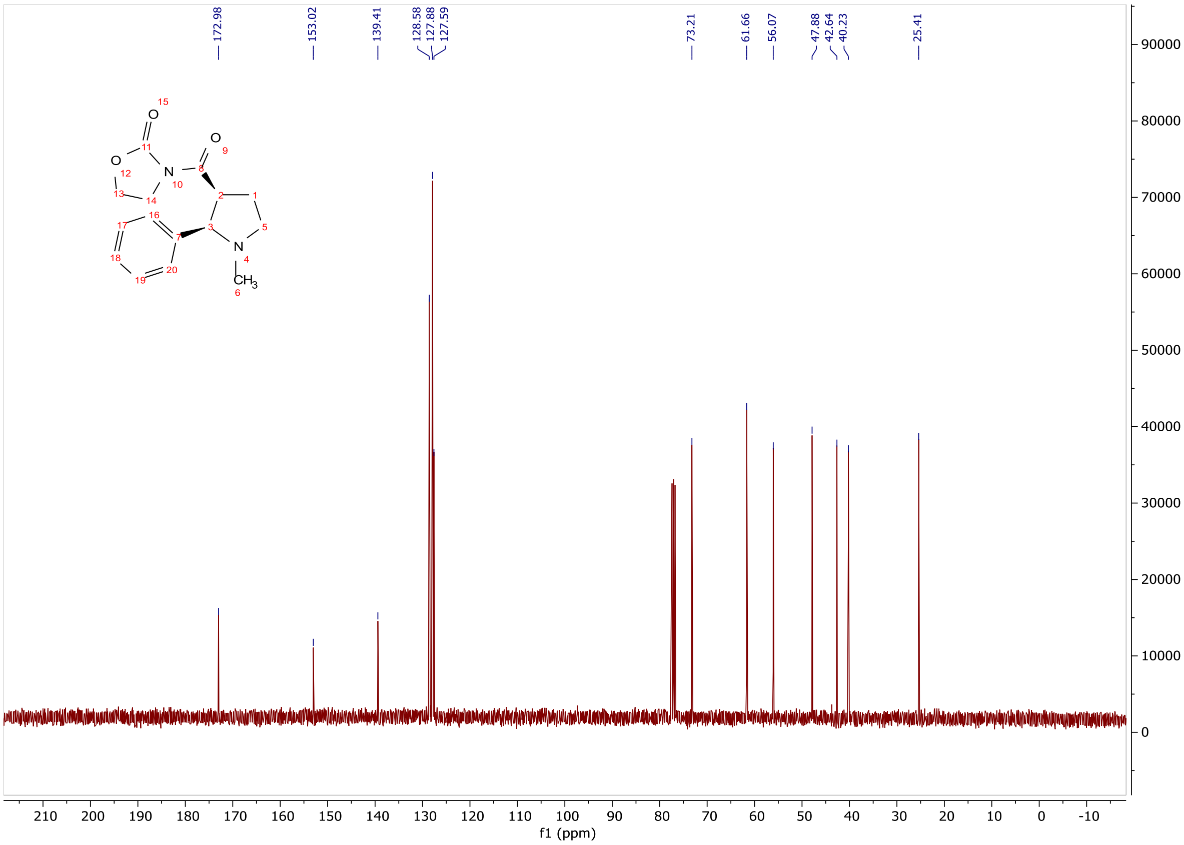

NMR spectra for 5b.

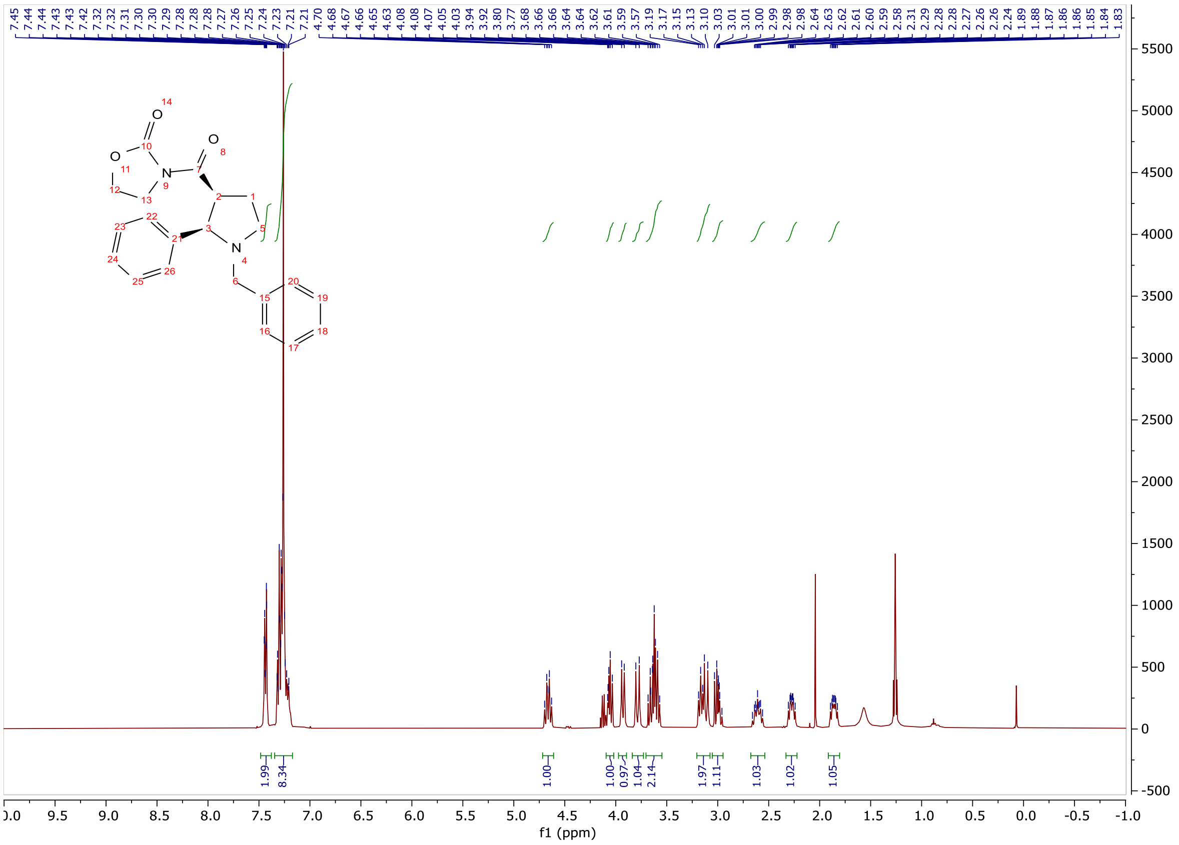

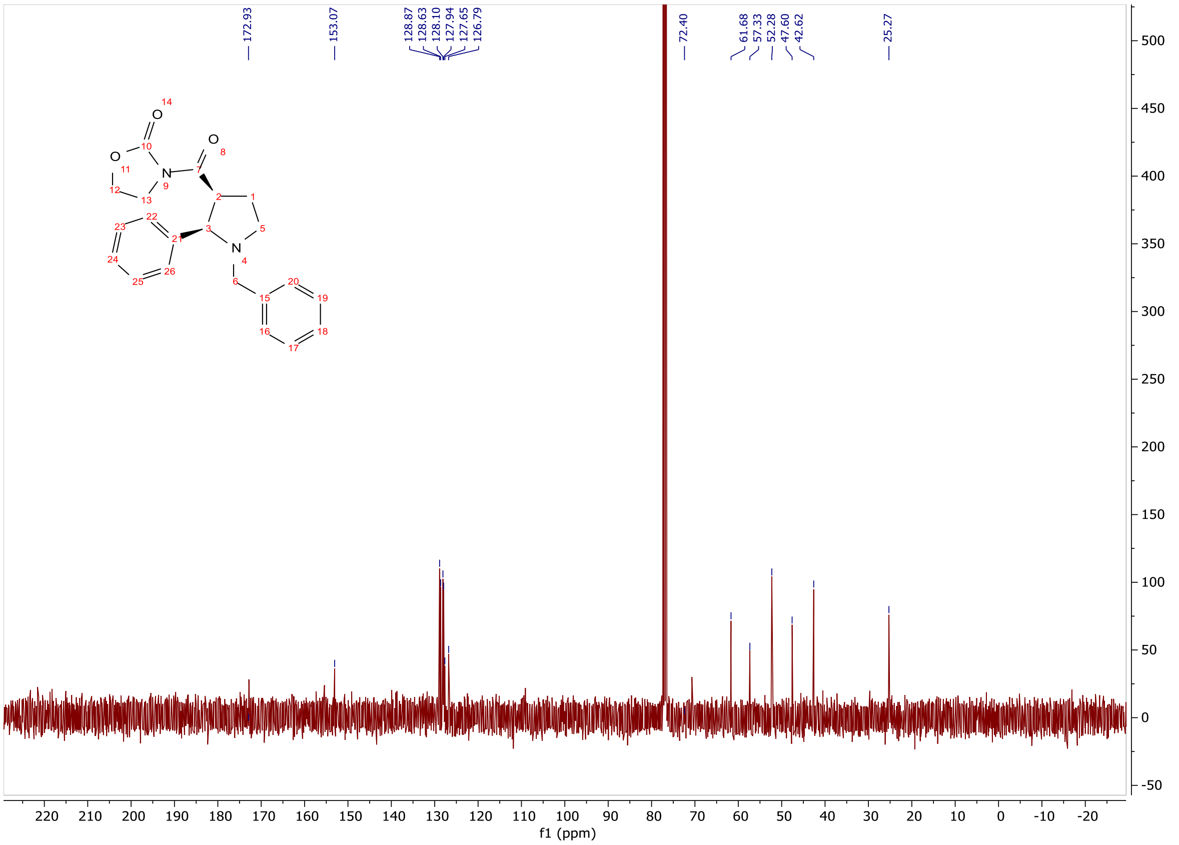

NMR spectra for 5c.

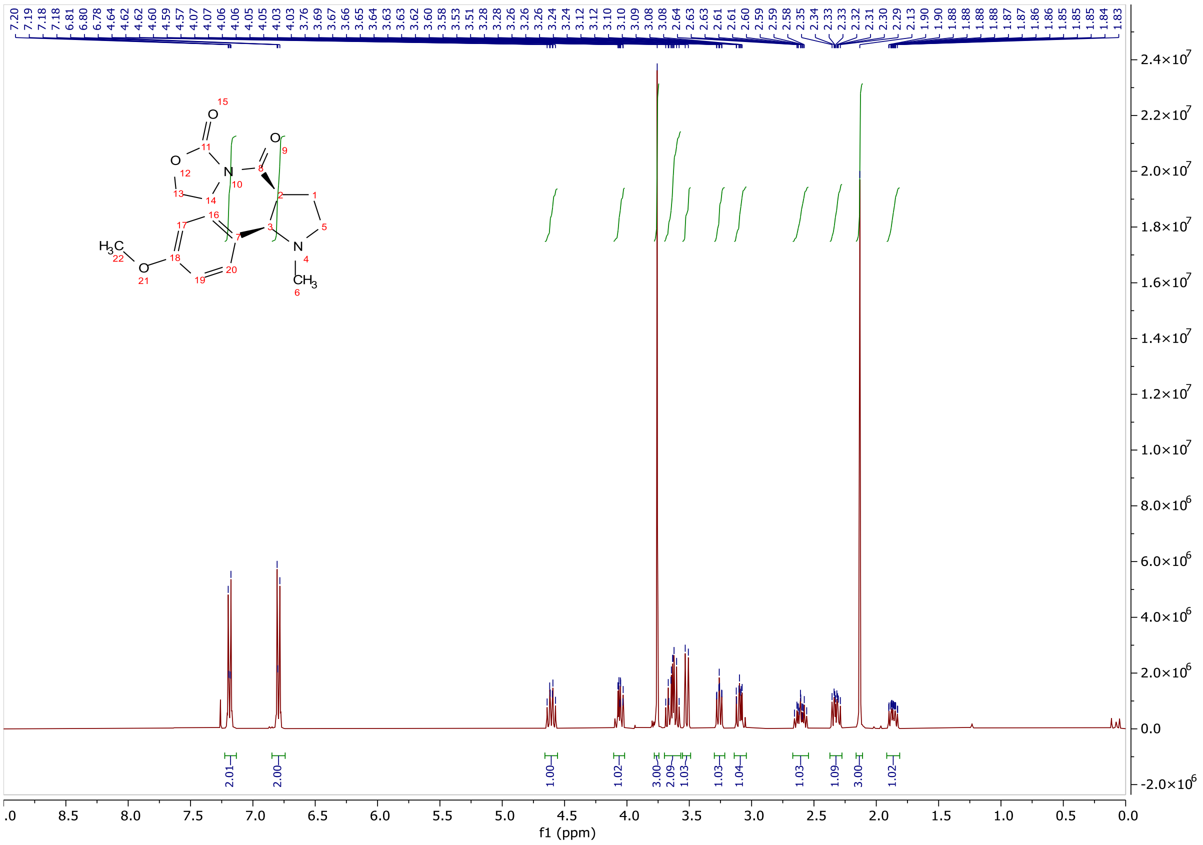

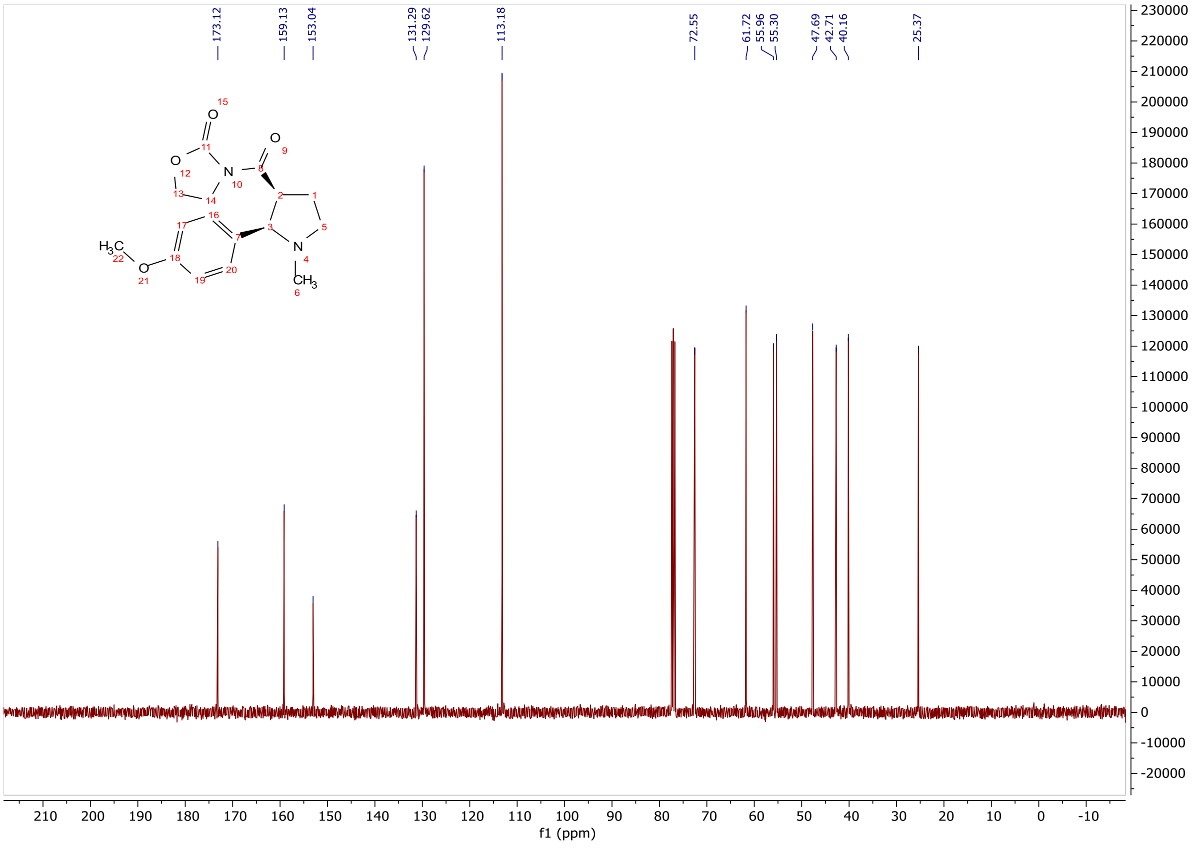

NMR spectra for 5d.

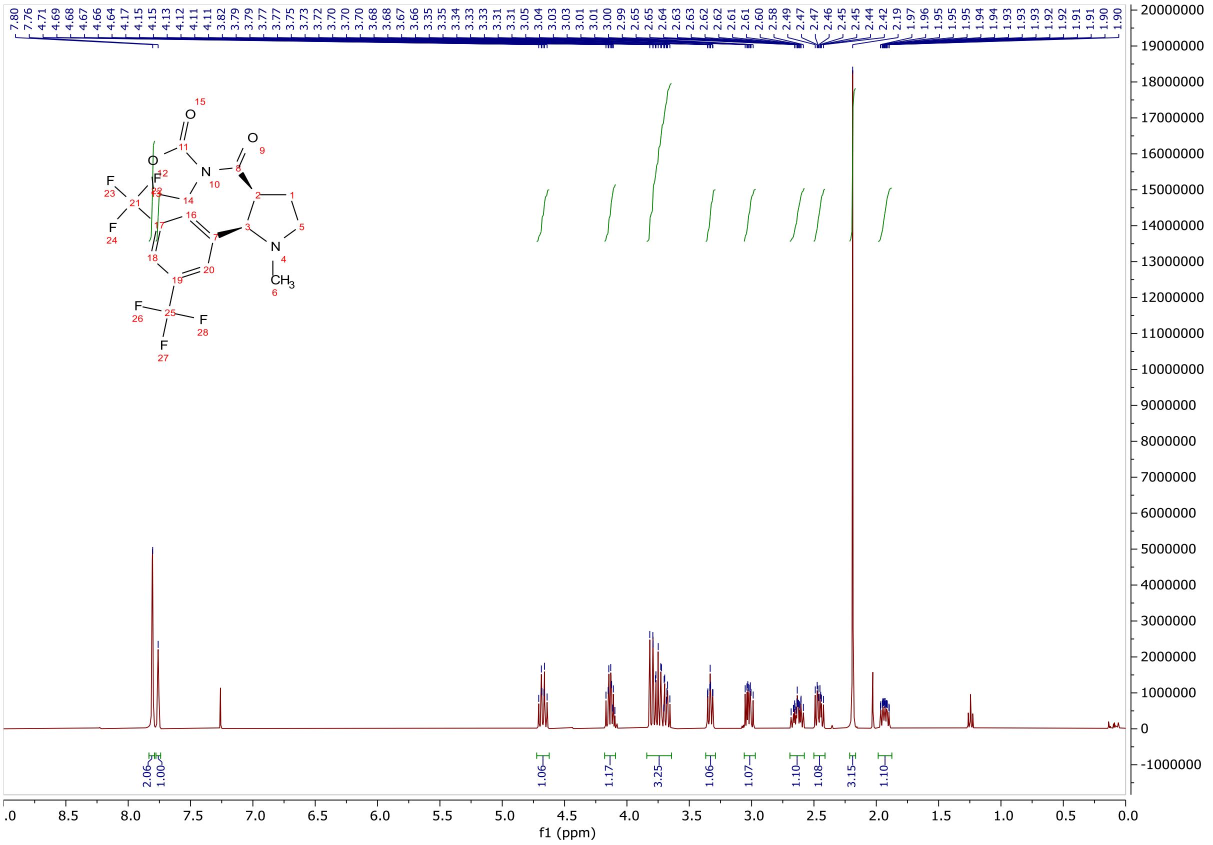

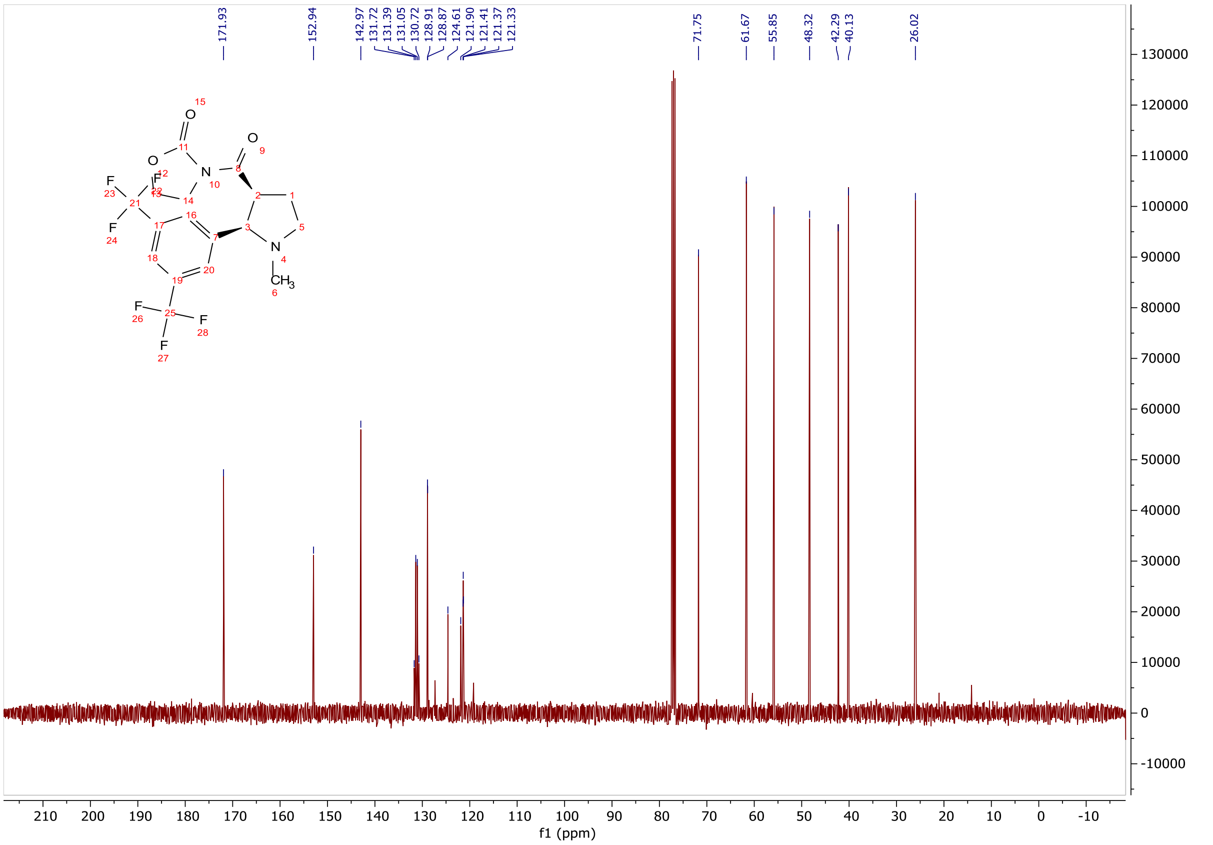

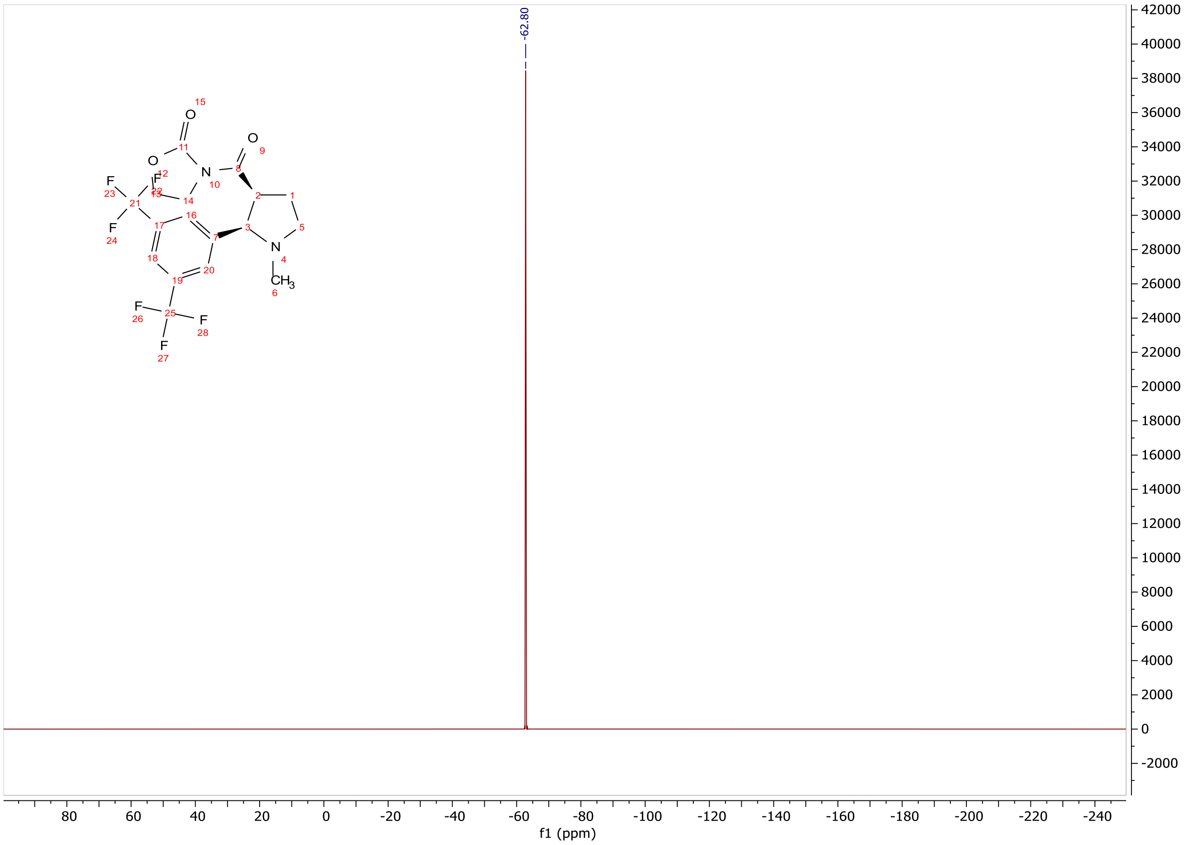
-62.80
f1 (ppm)

NMR spectra for 5e.

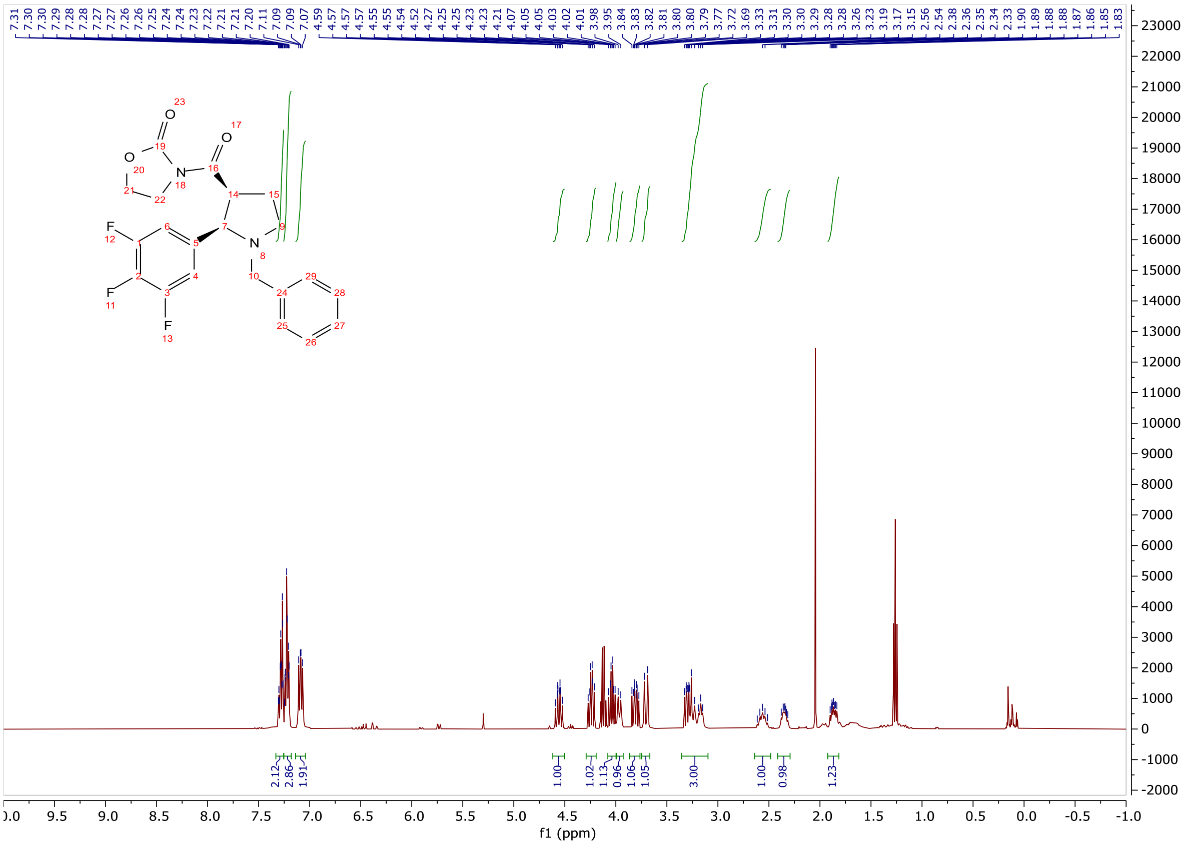

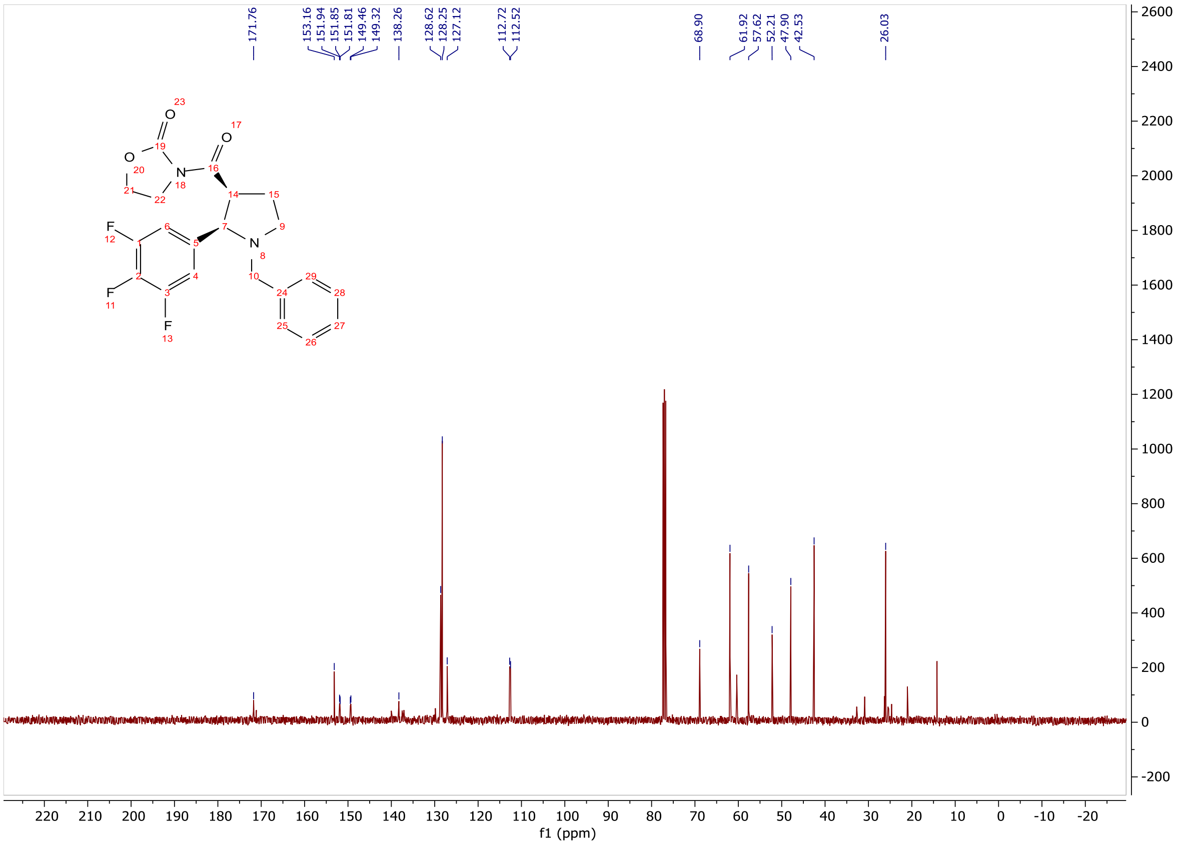

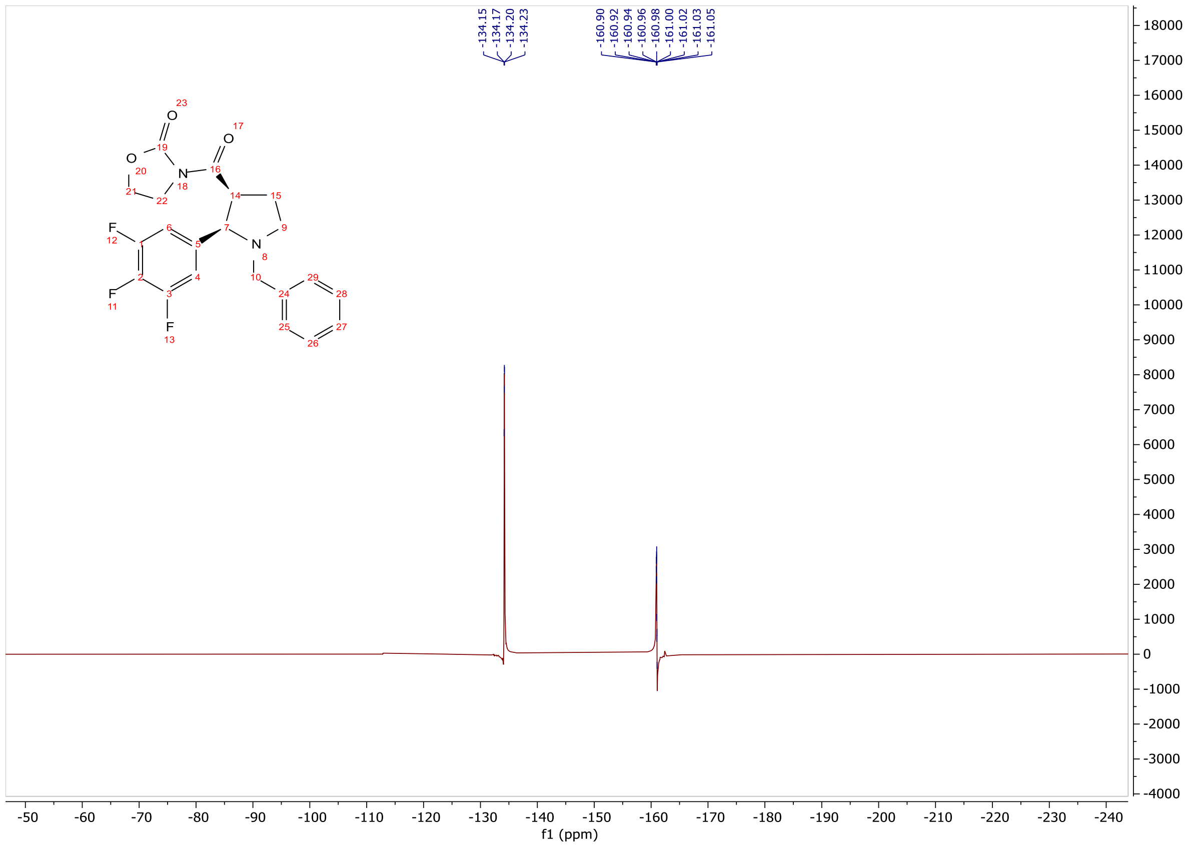
-134.15
-134.17
-134.20
-134.23
-160.90
-160.92
-160.94
-160.96
-160.98
-161.00
-161.02
-161.03
-161.05
f1 (ppm)

NMR spectra for 5f.

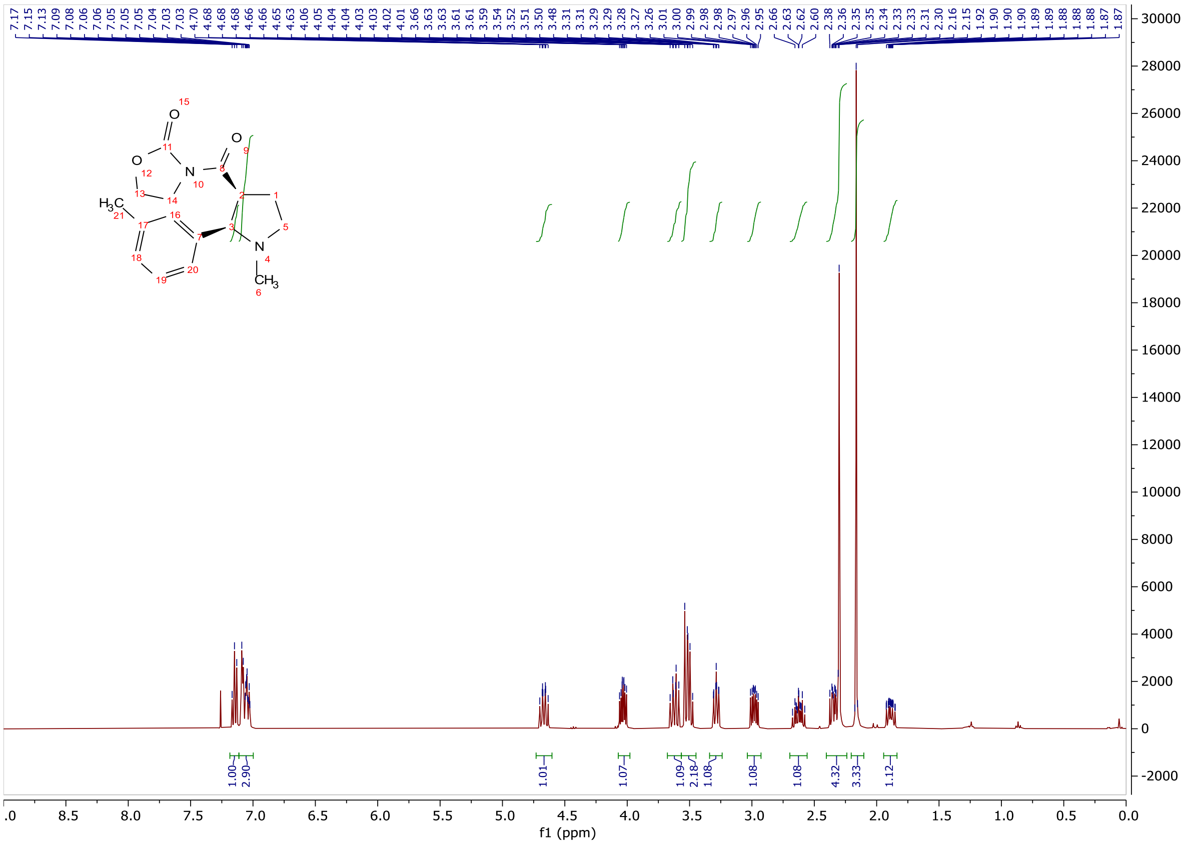

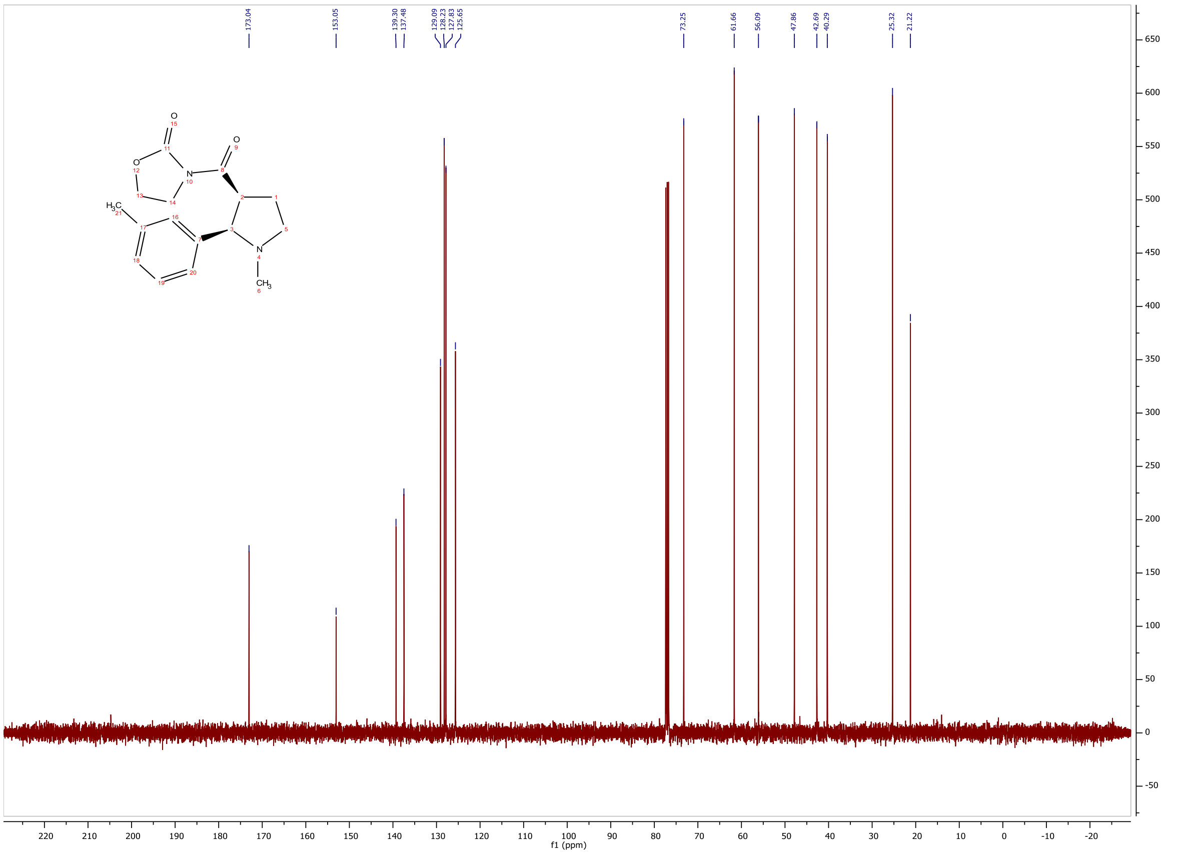

NMR spectra for 5g.

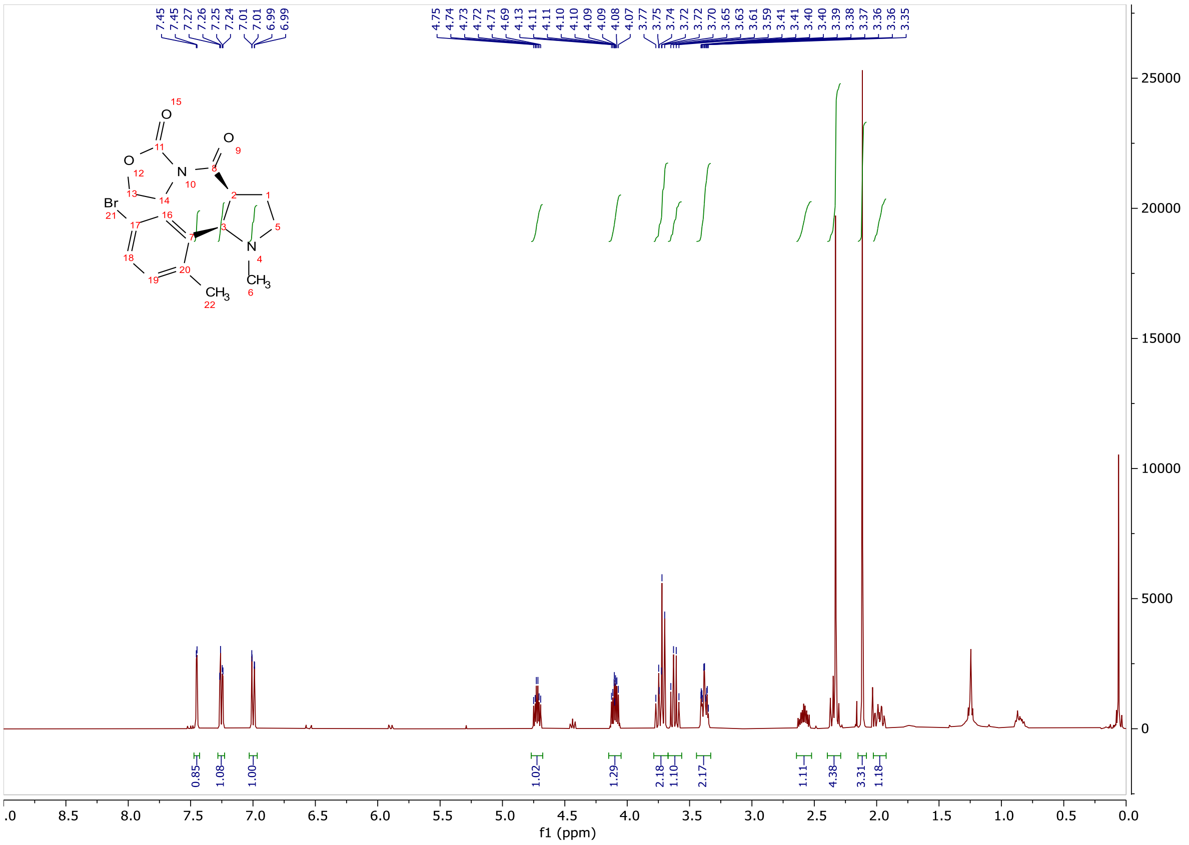

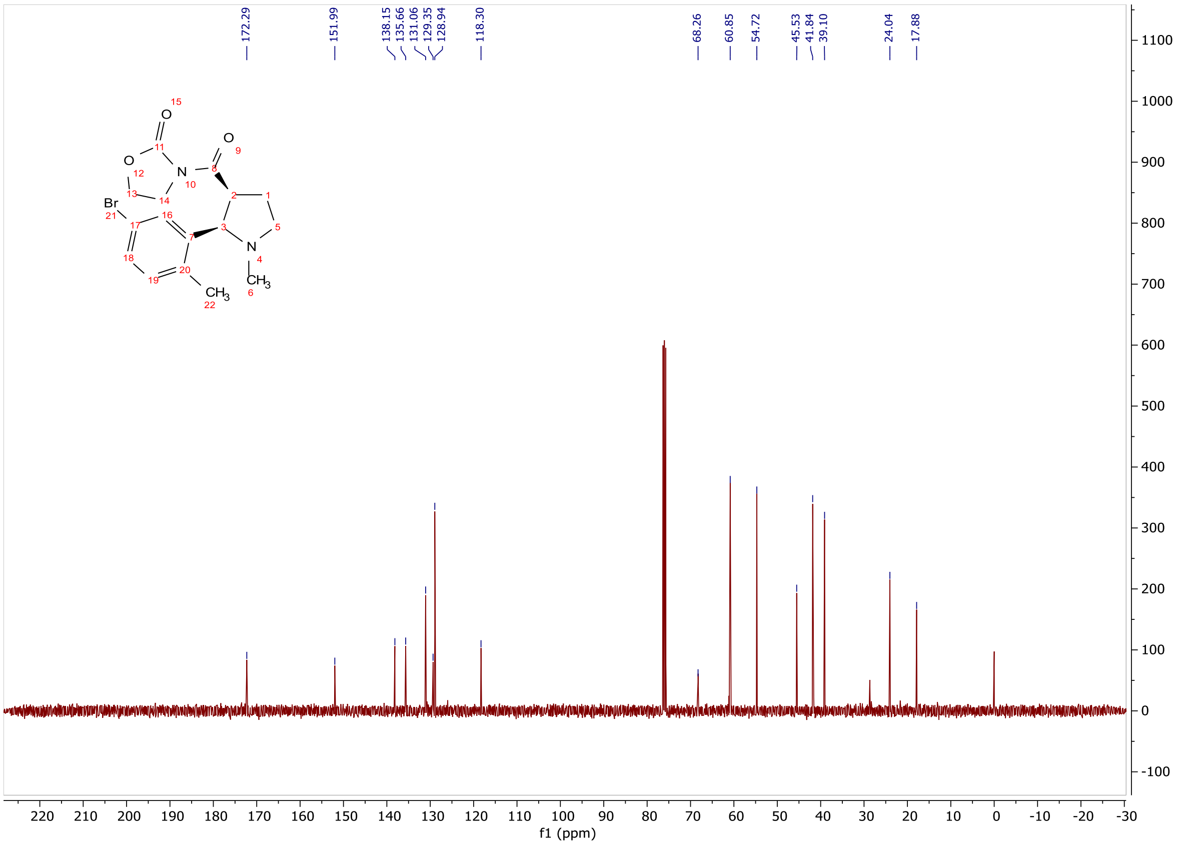

NMR spectra for 5h.

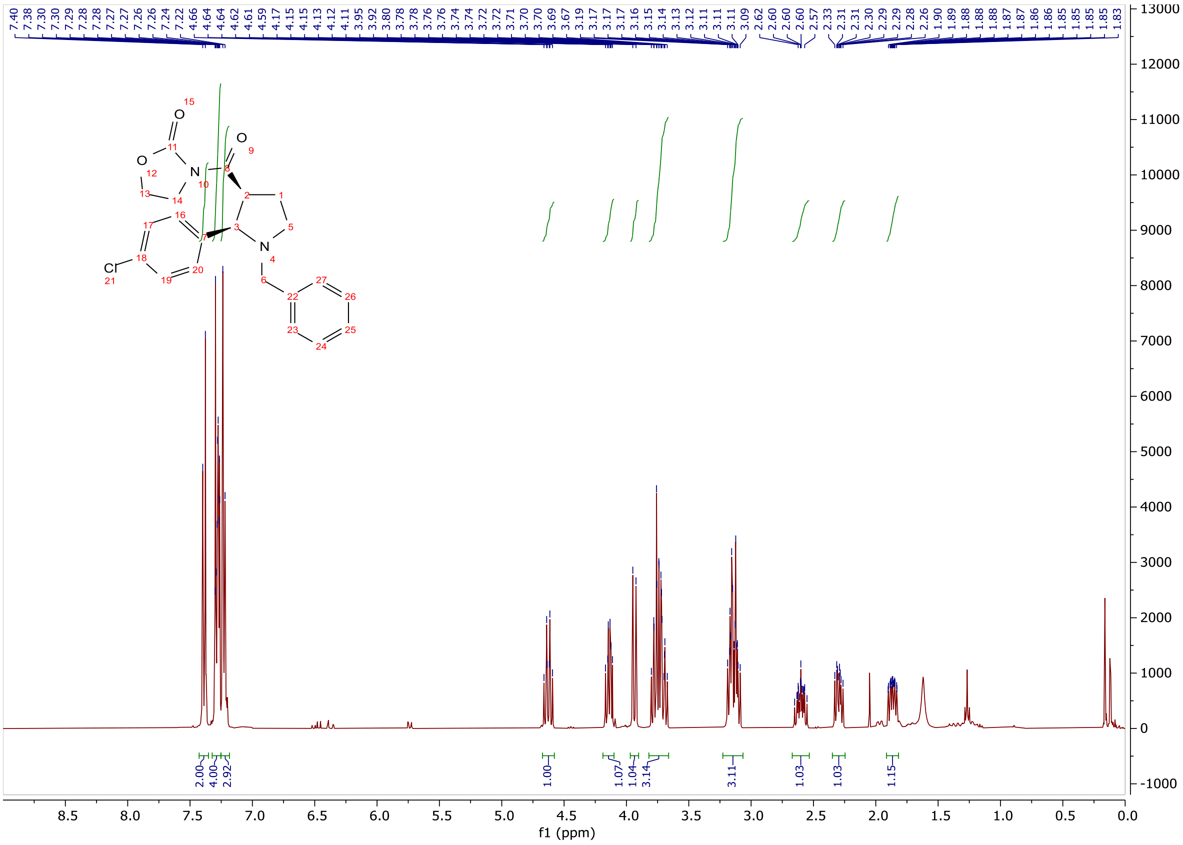

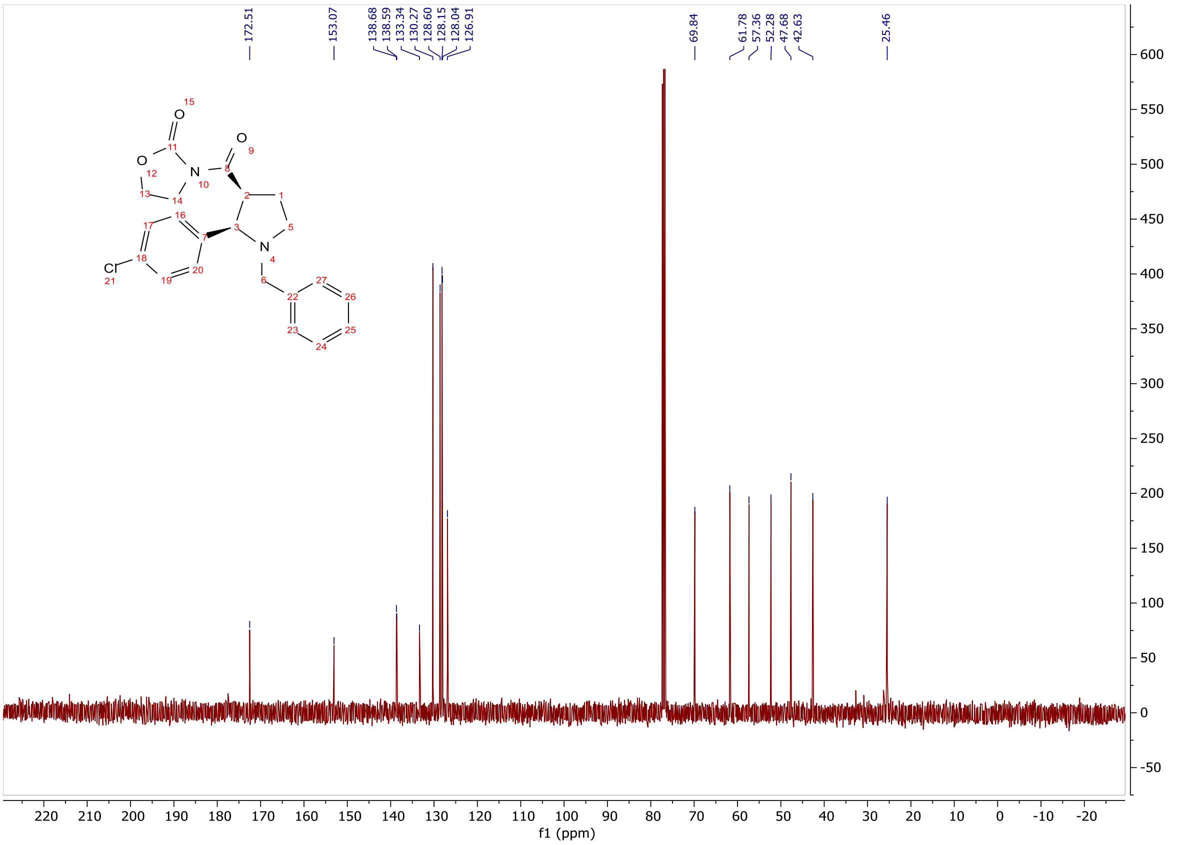

NMR spectra for 5i.

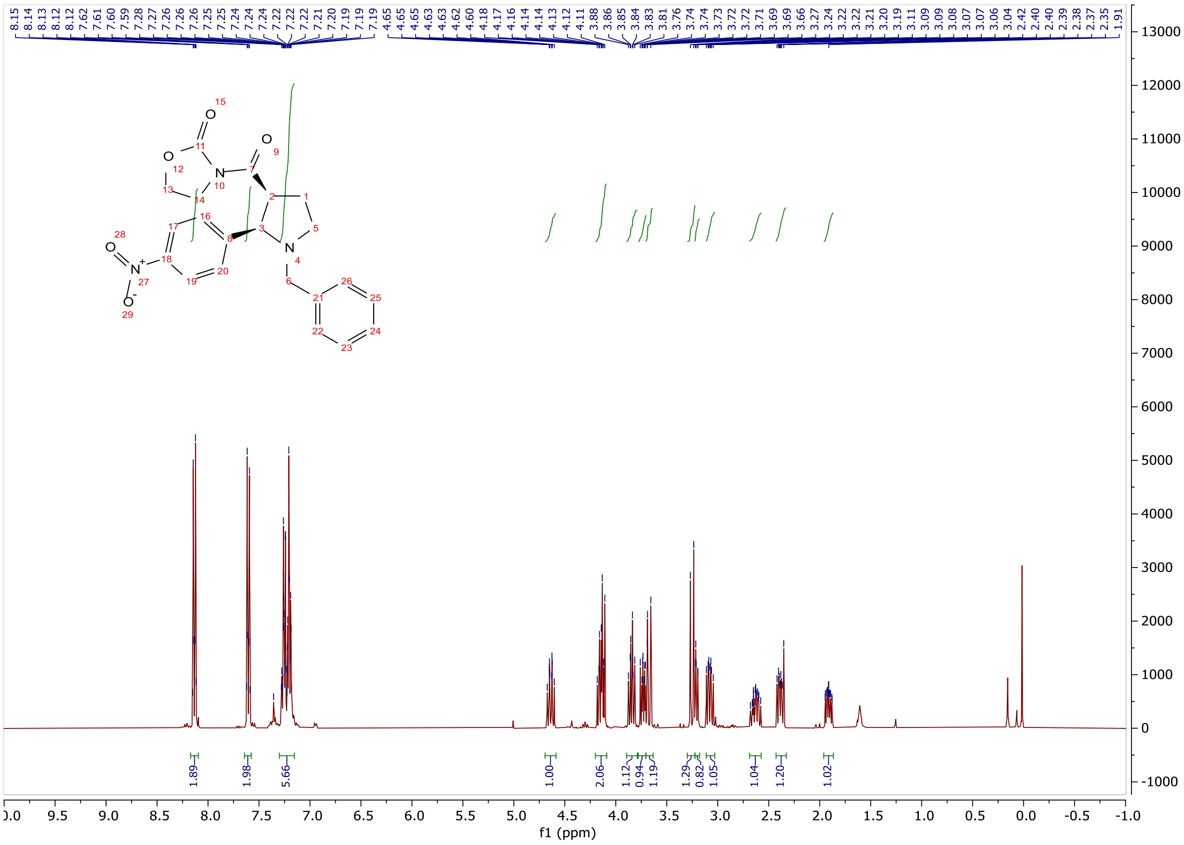

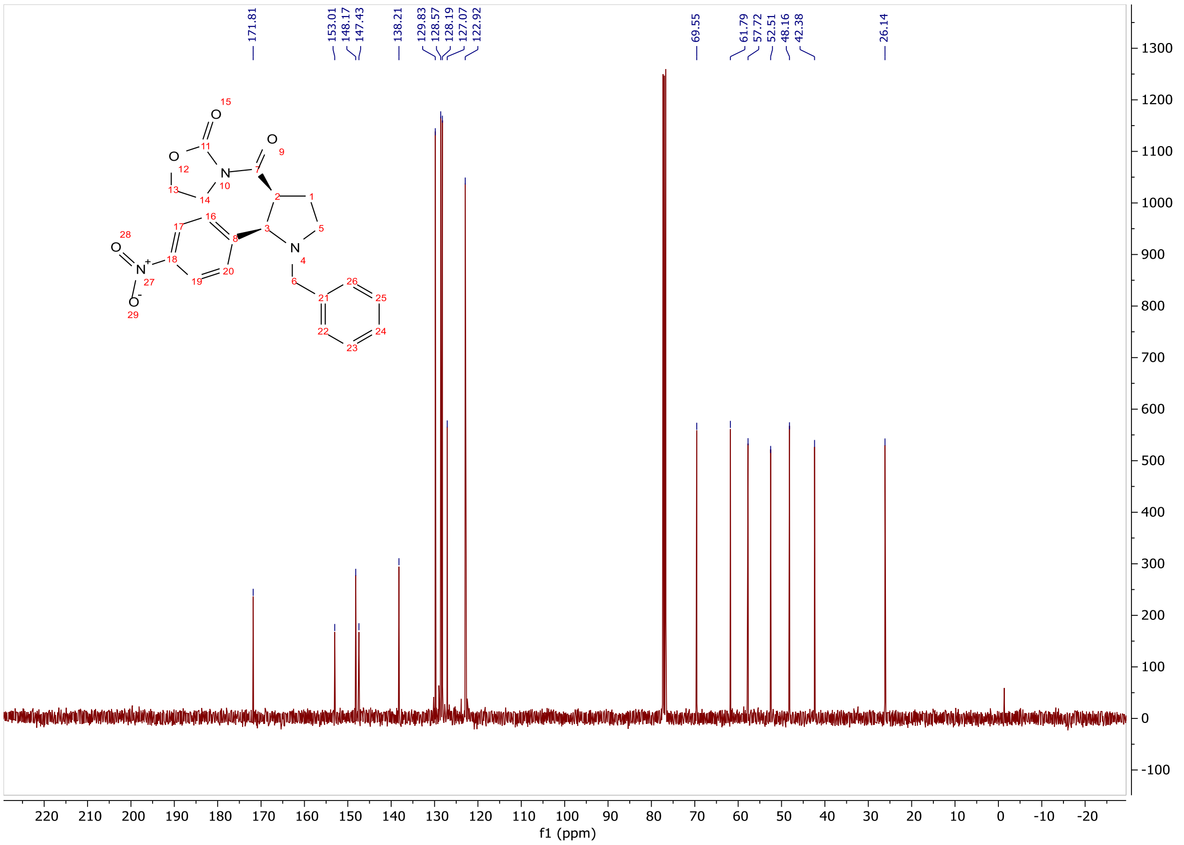

NMR spectra for 5j.

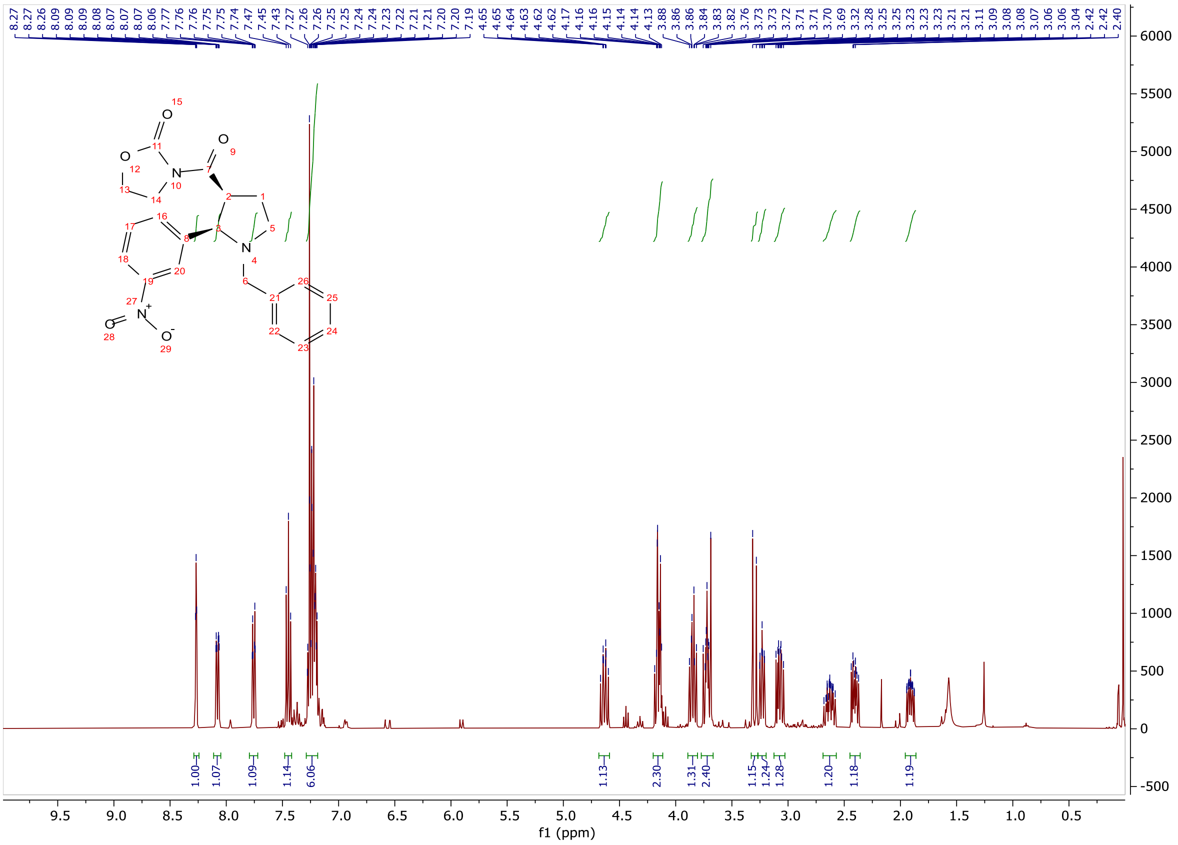

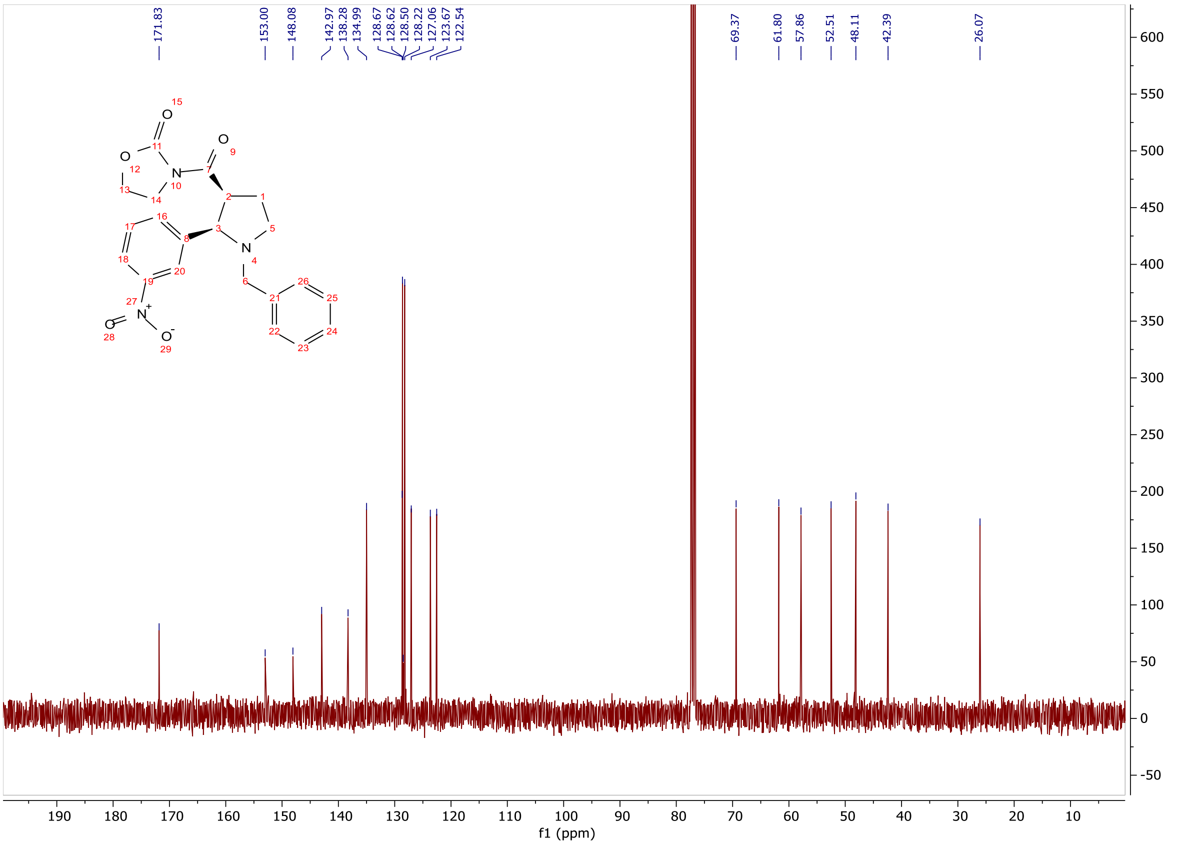

NMR spectra for 5k.

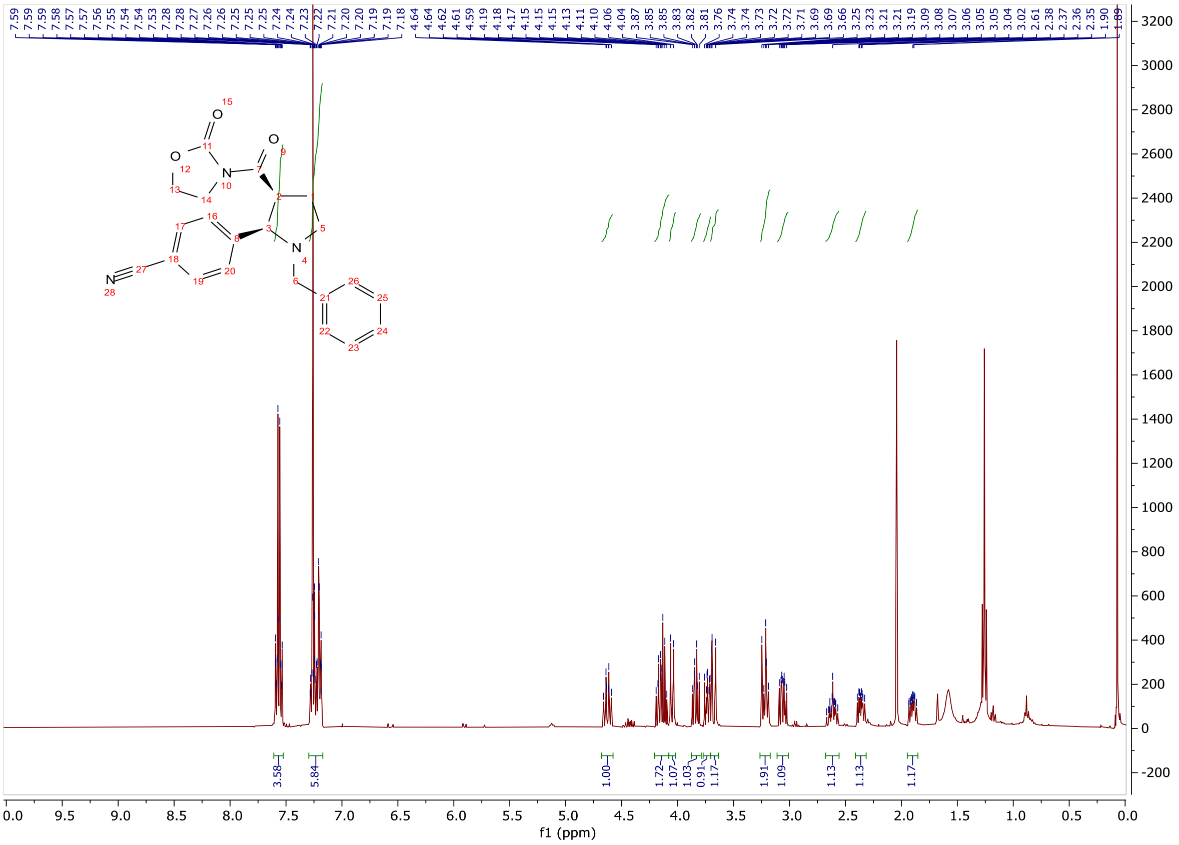

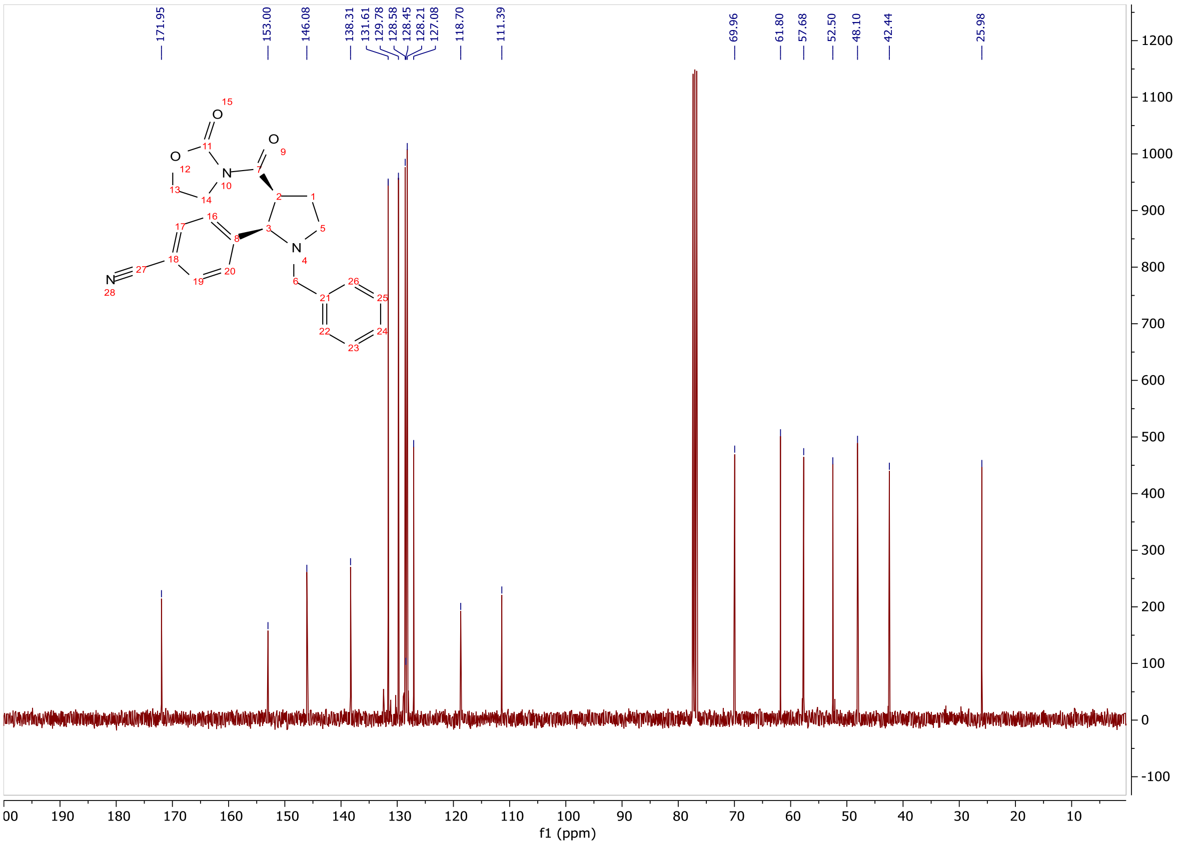

NMR spectra for 5l.

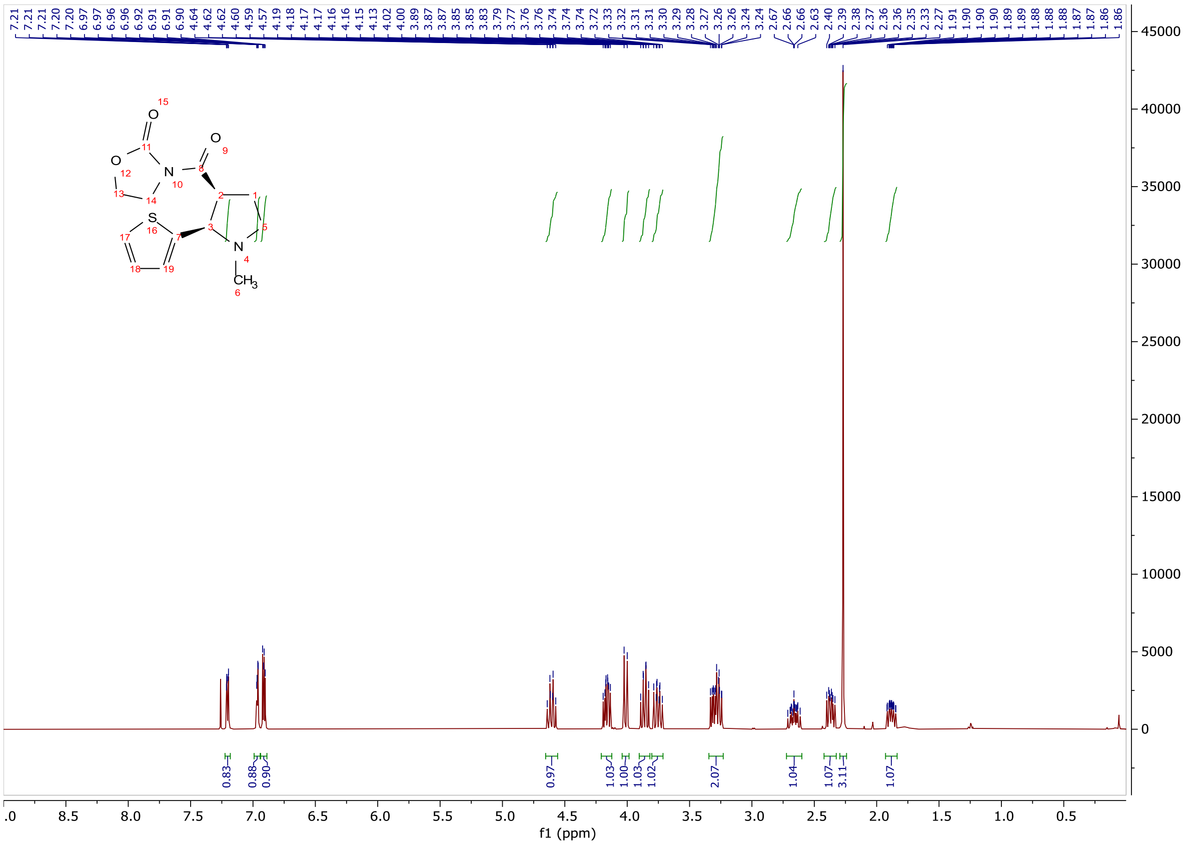

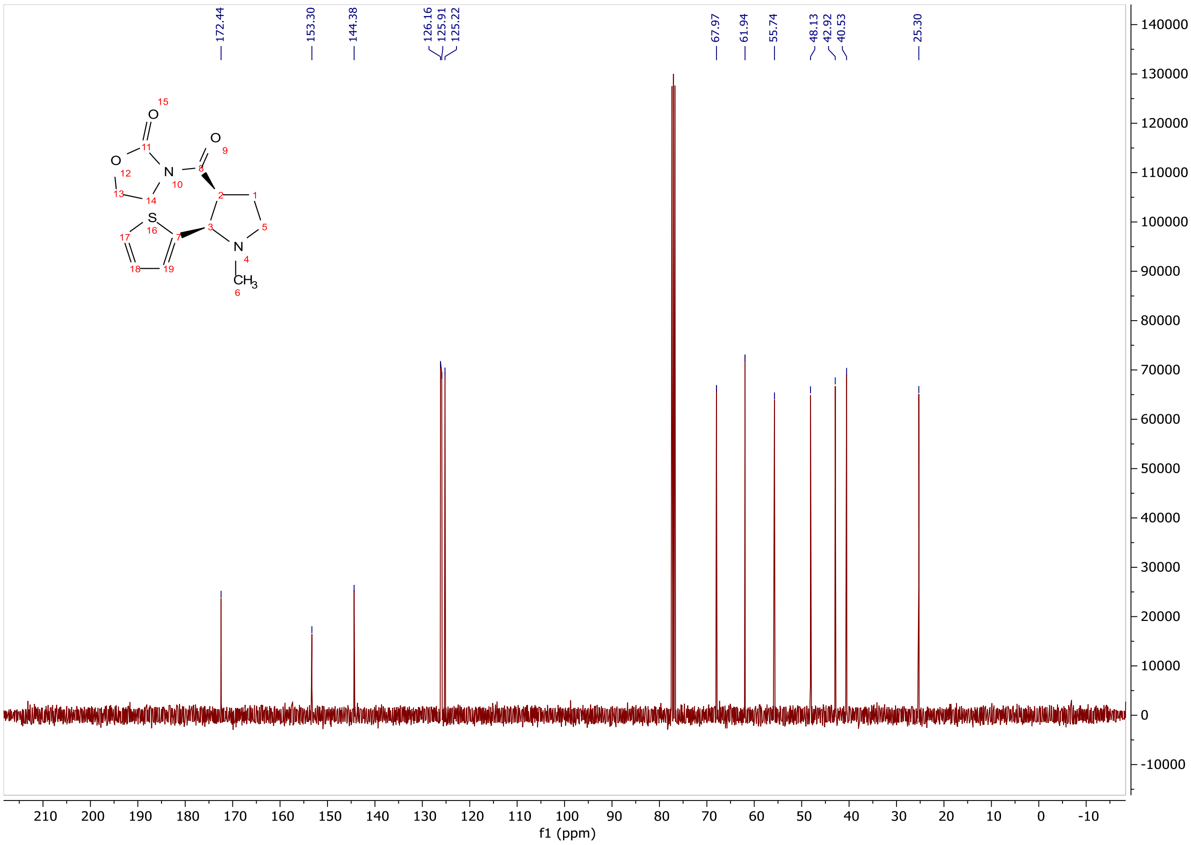

NMR spectra for 5m.

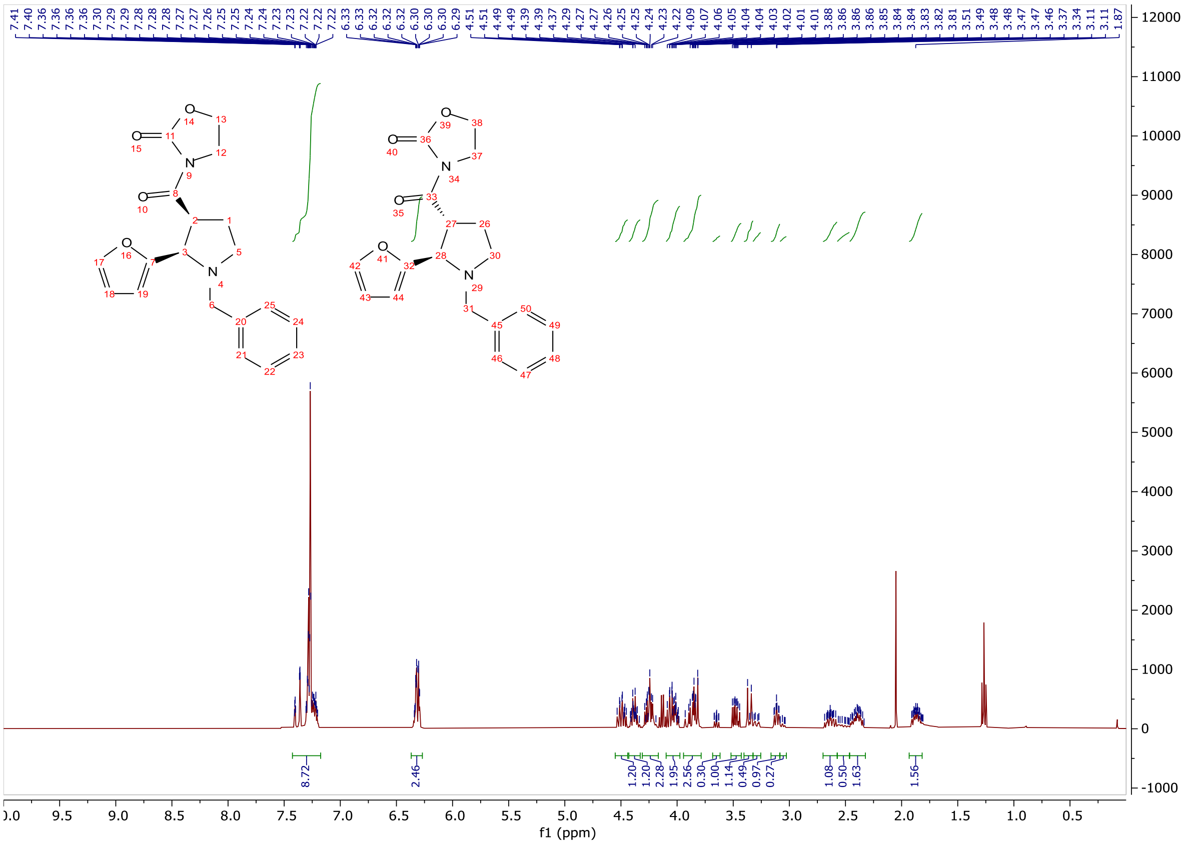

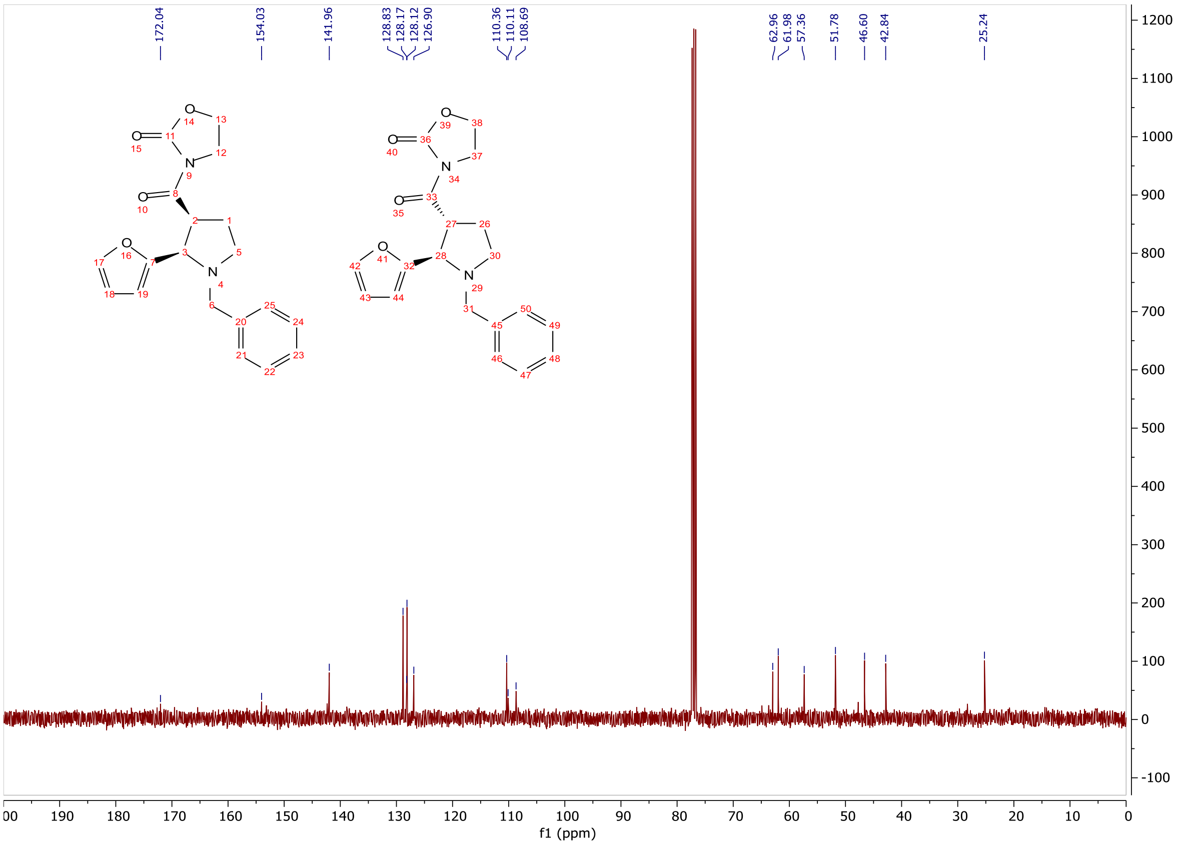

NMR spectra for 5n.

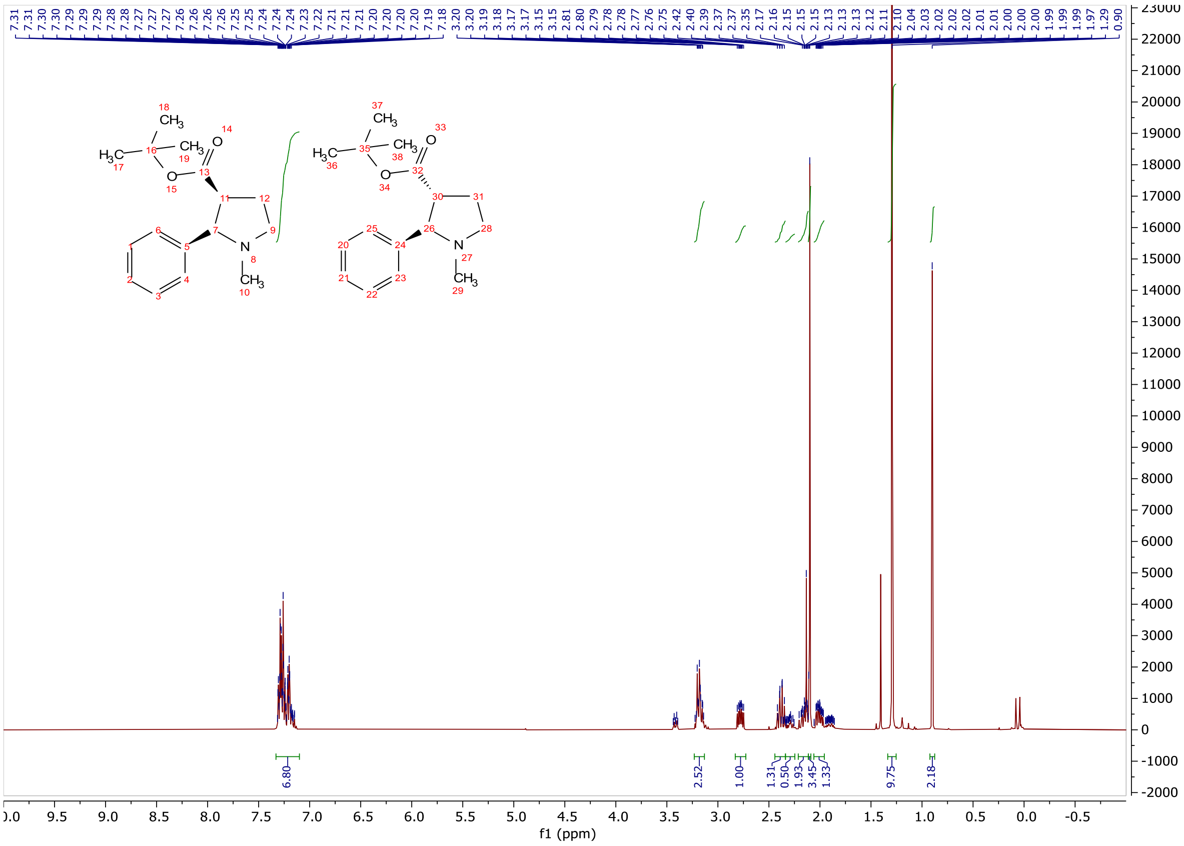

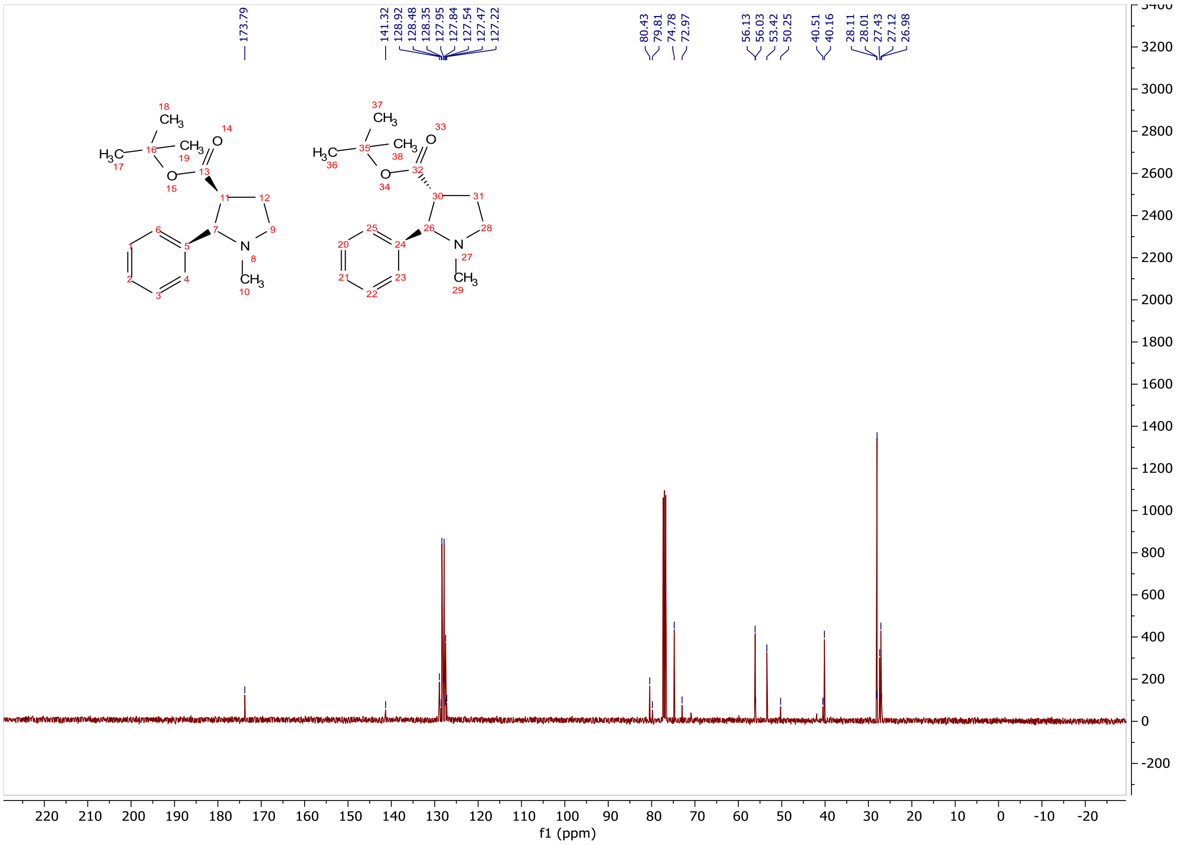

NMR spectra for 5o.

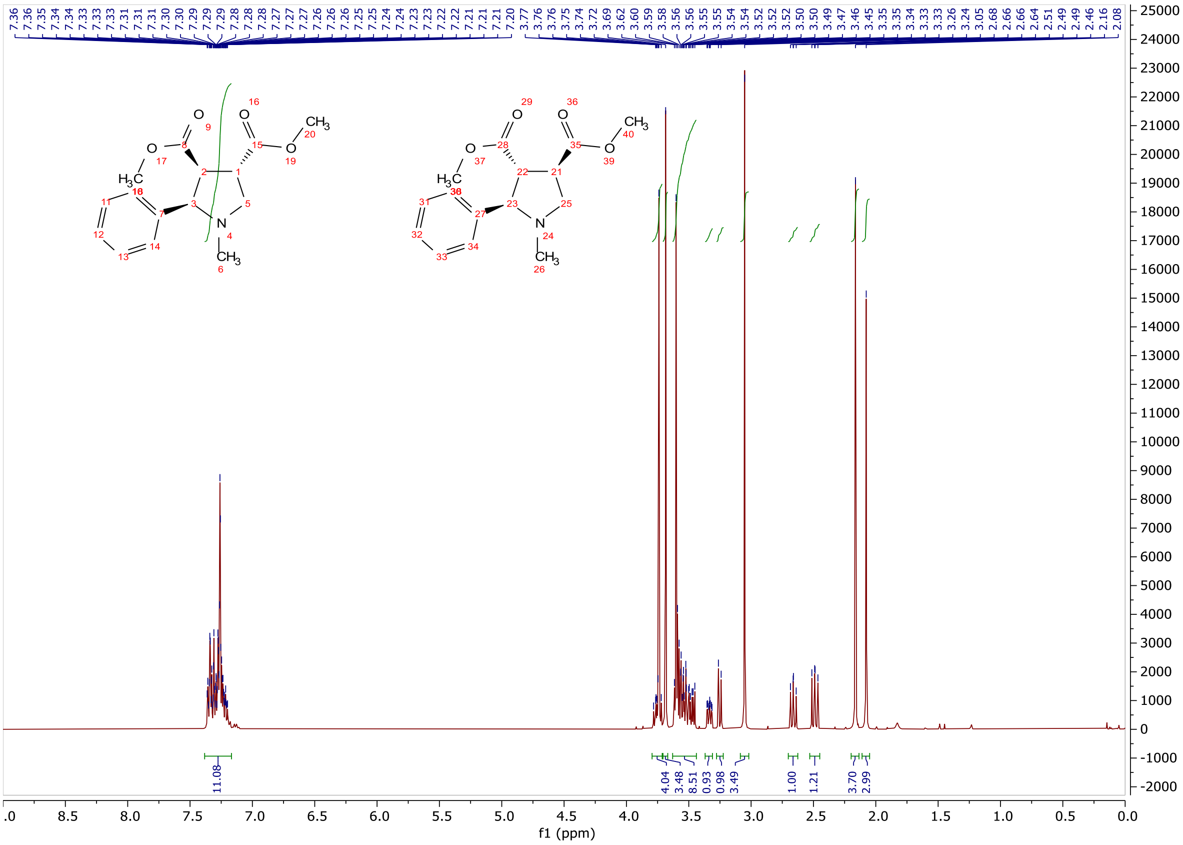

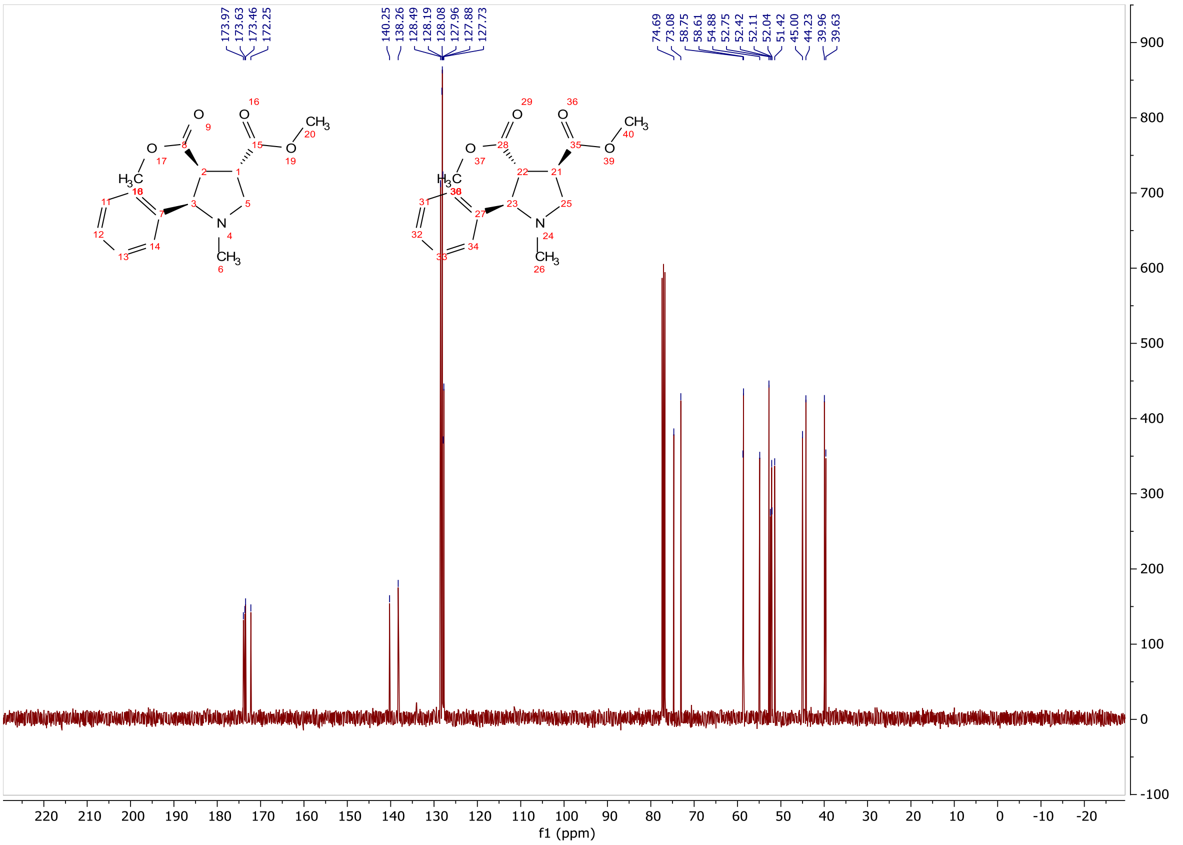

NMR spectra for 5p.

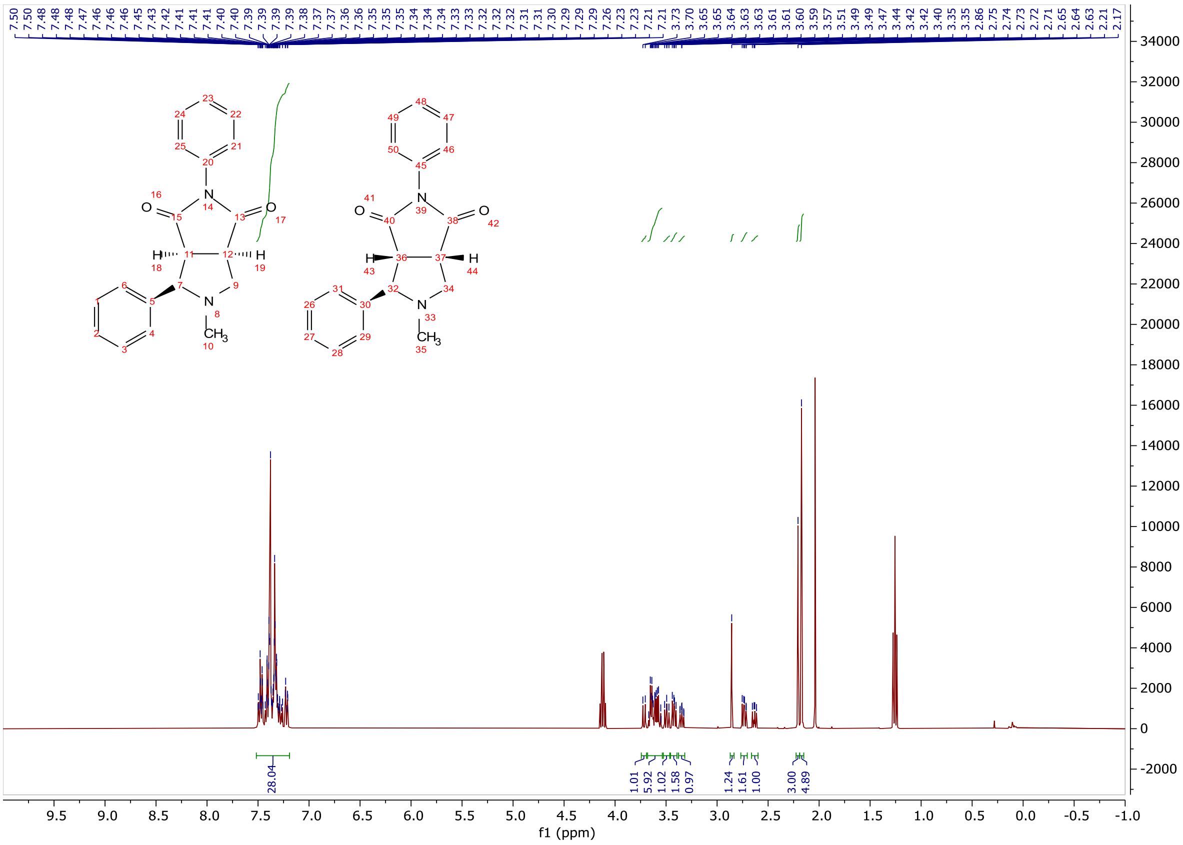

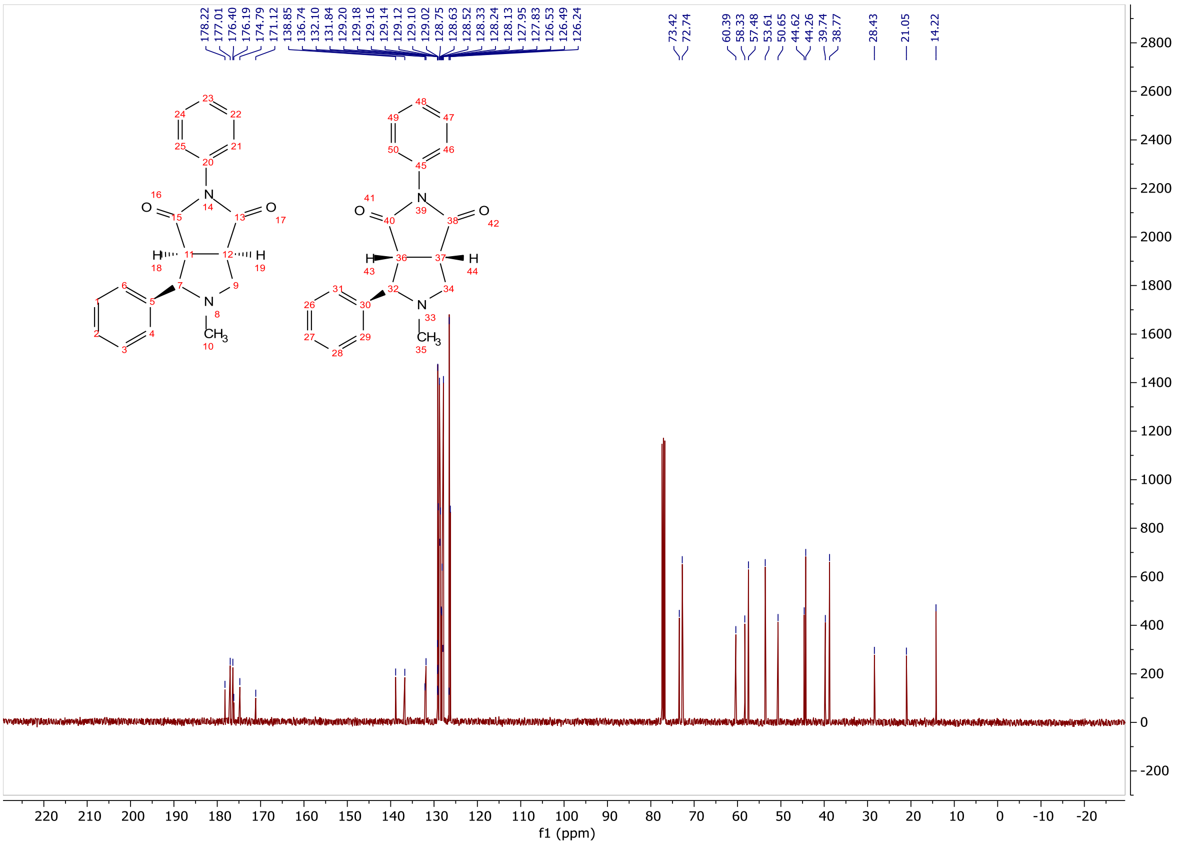

NMR spectra for 5q.

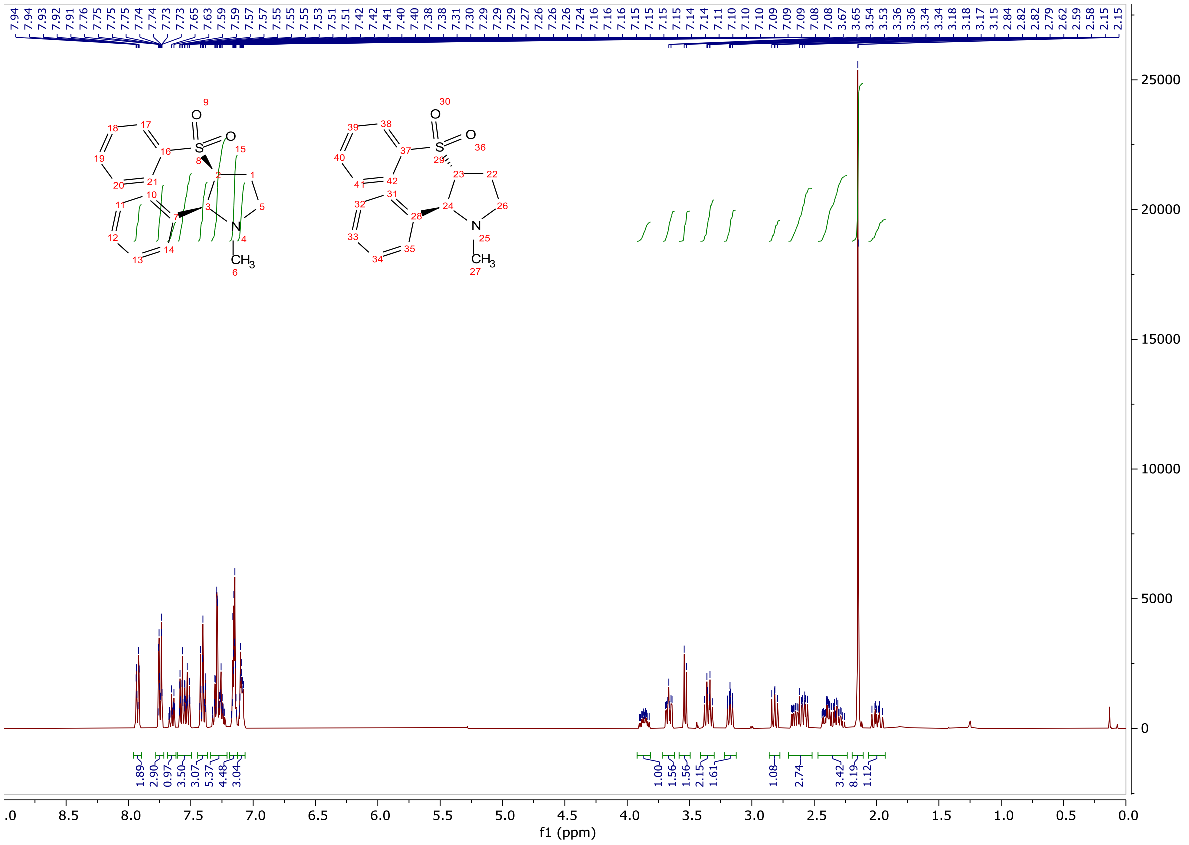

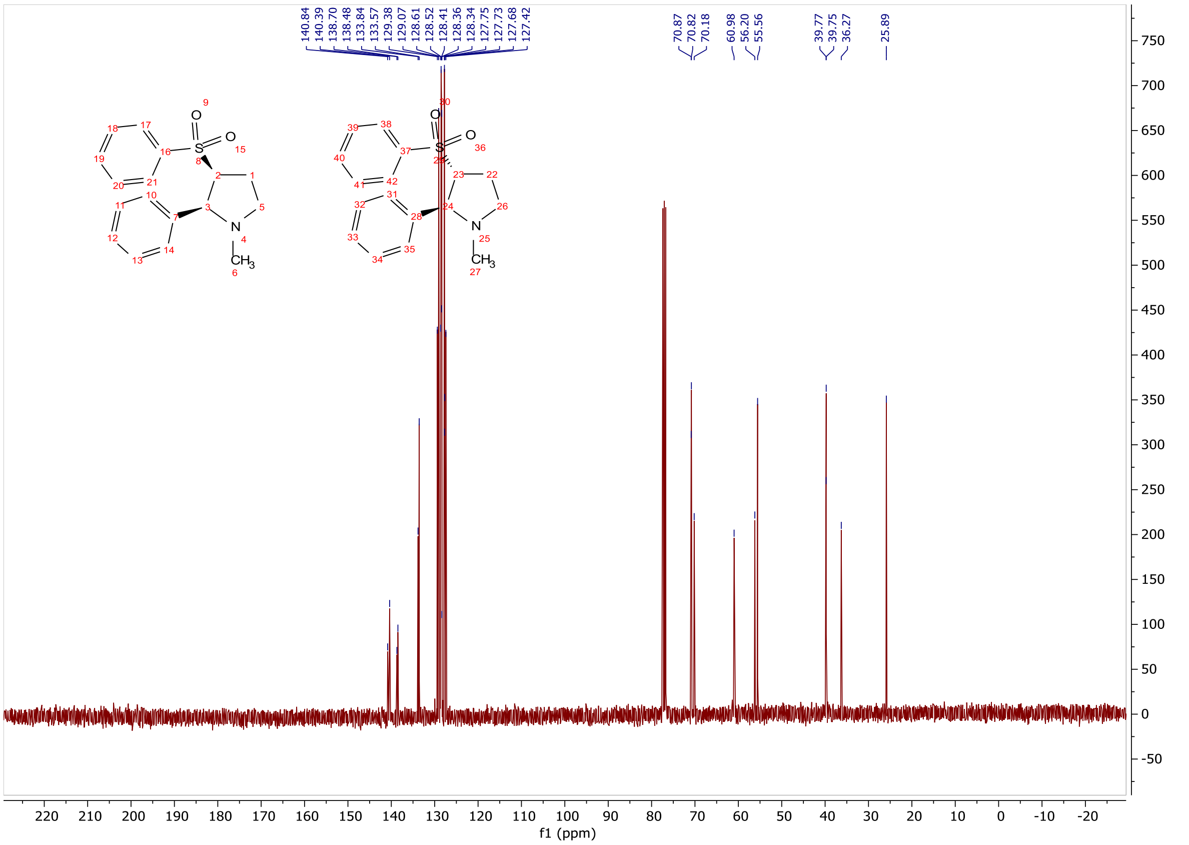

NMR spectra for 5r.

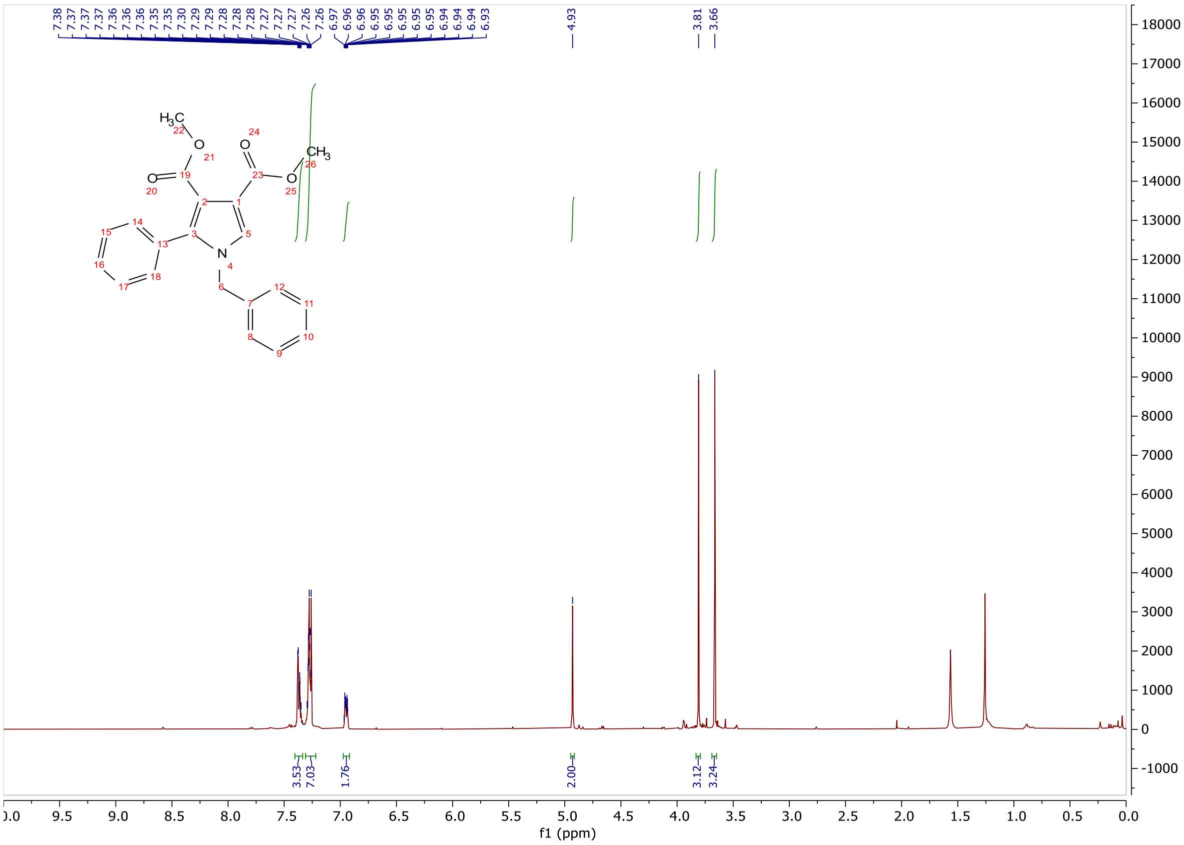

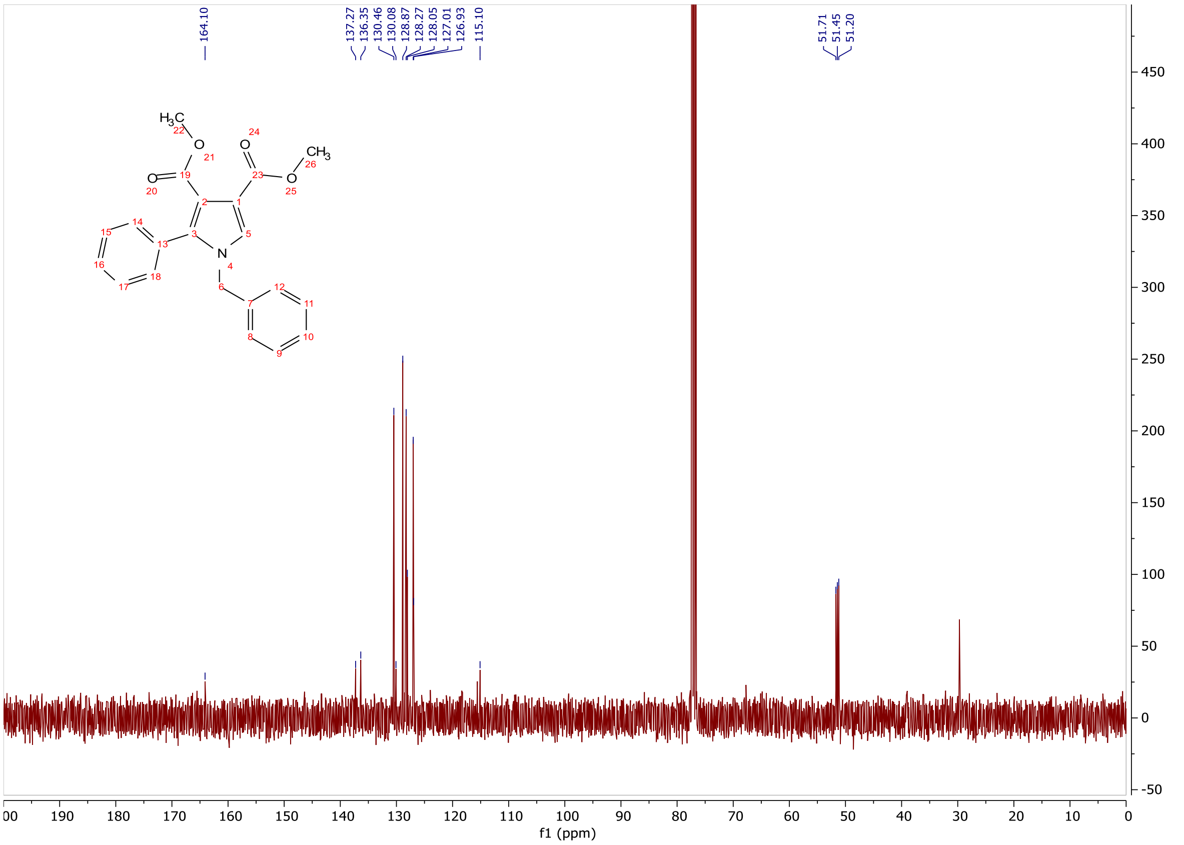

NMR spectra for S1.

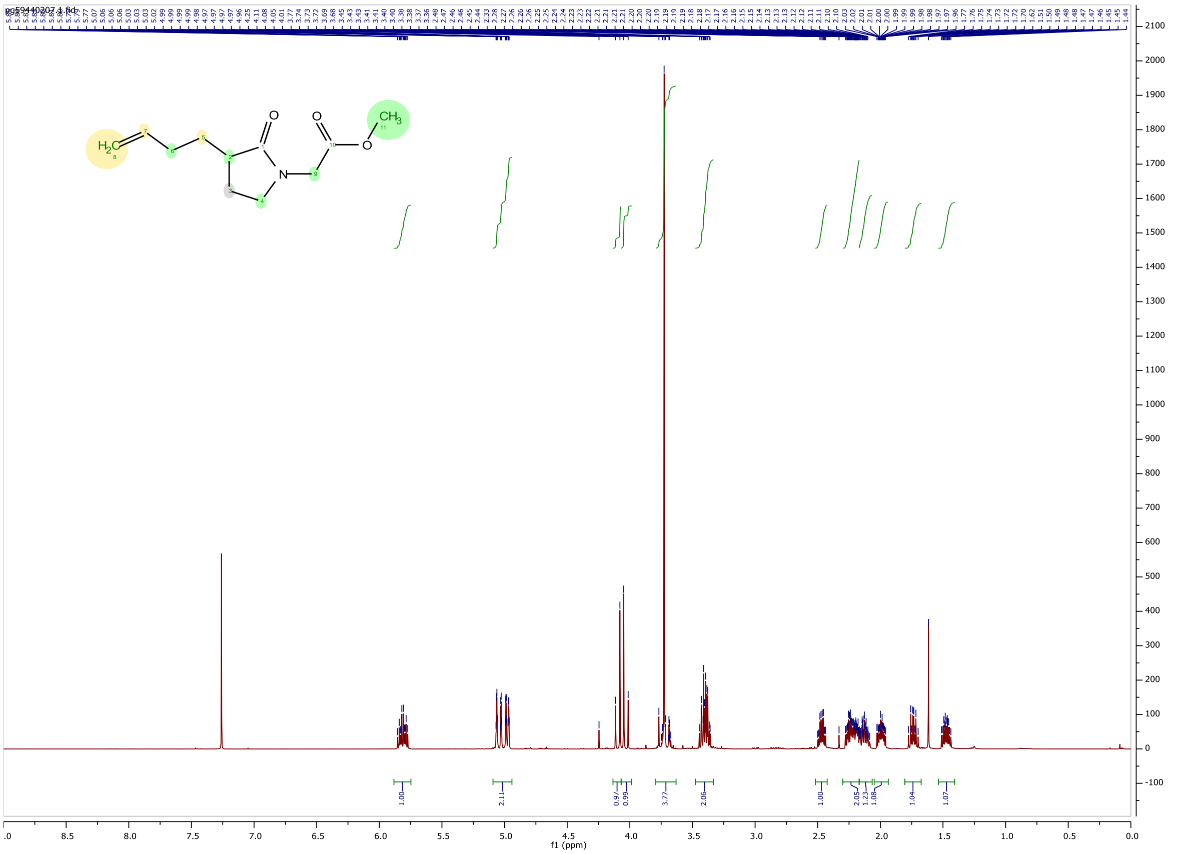

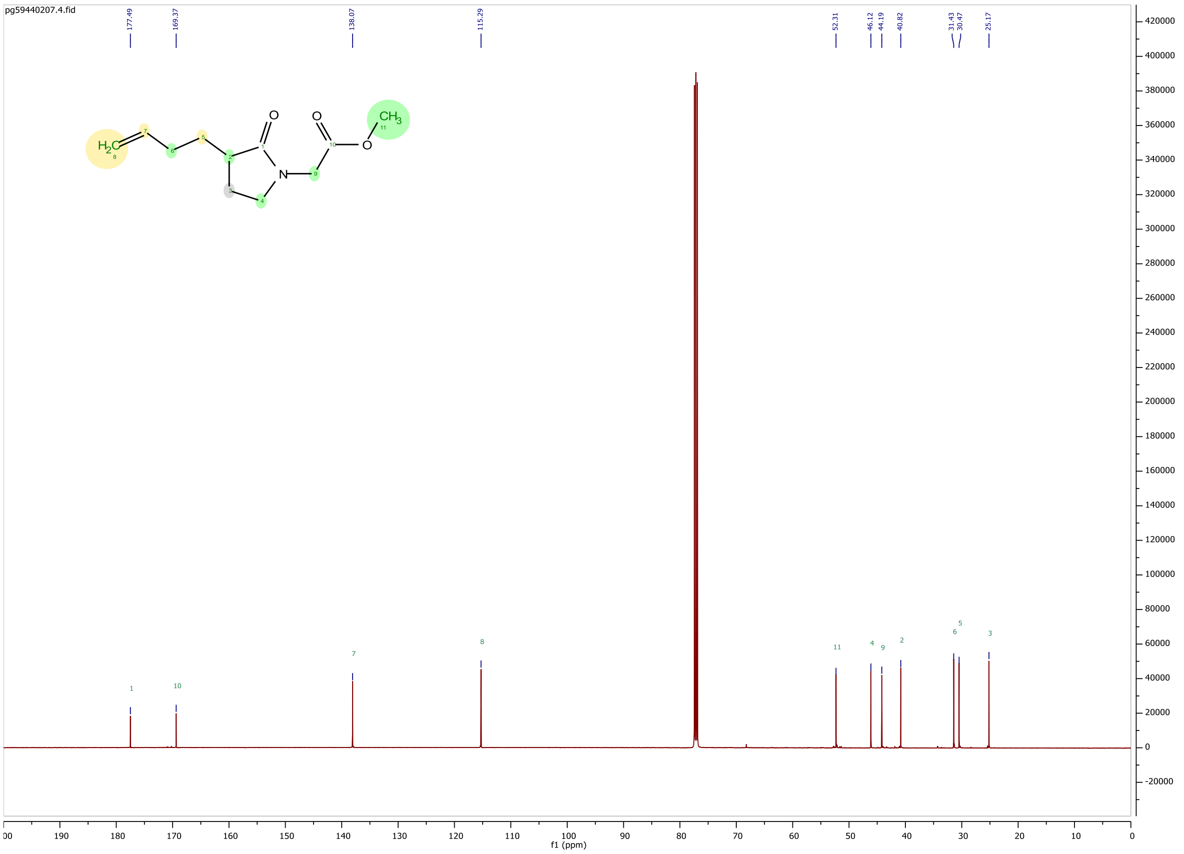

NMR spectra for 6.

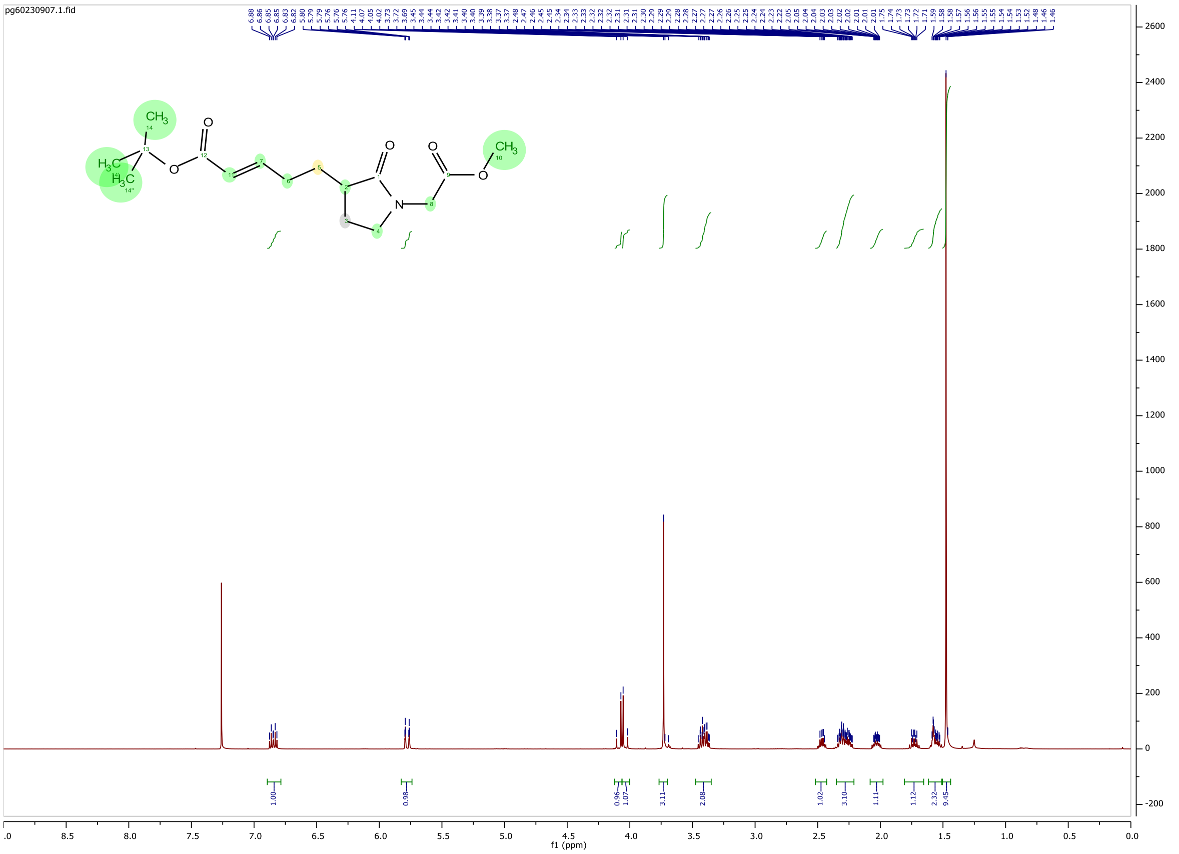

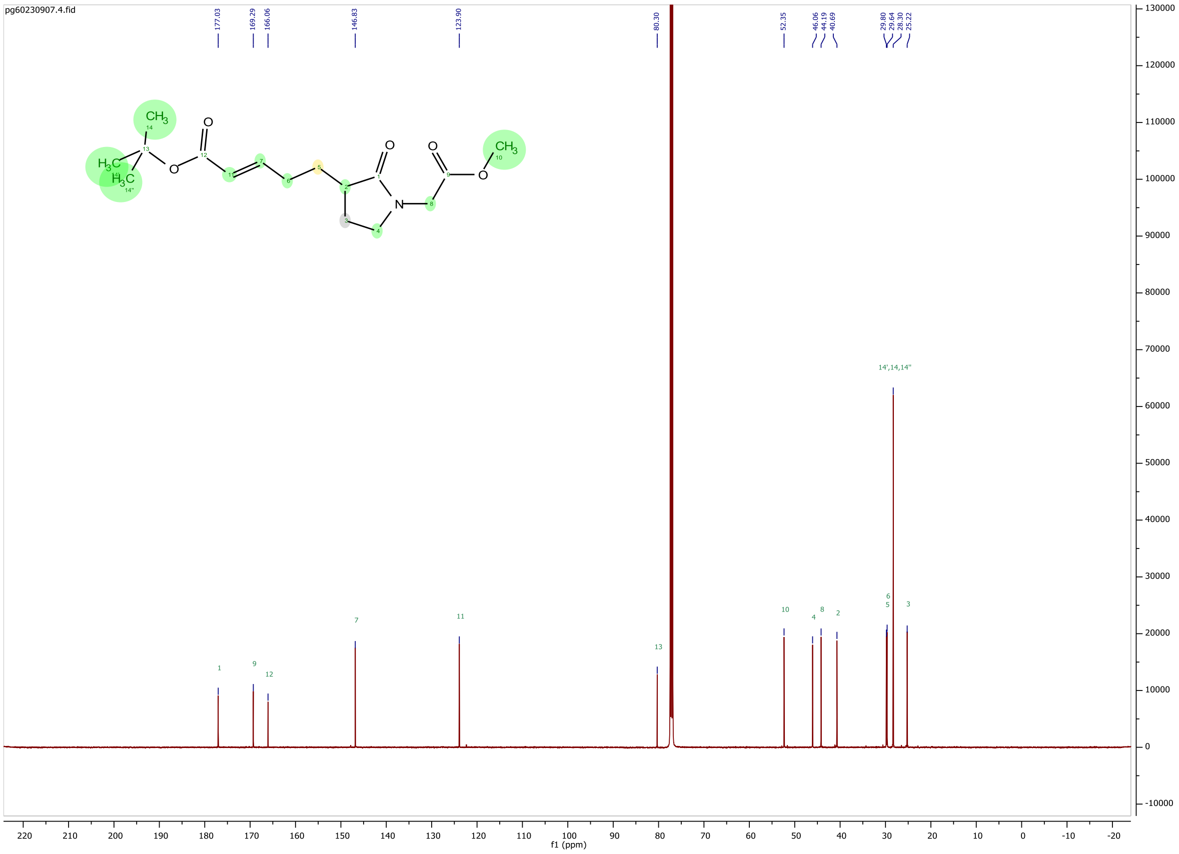

NMR spectra for 7.

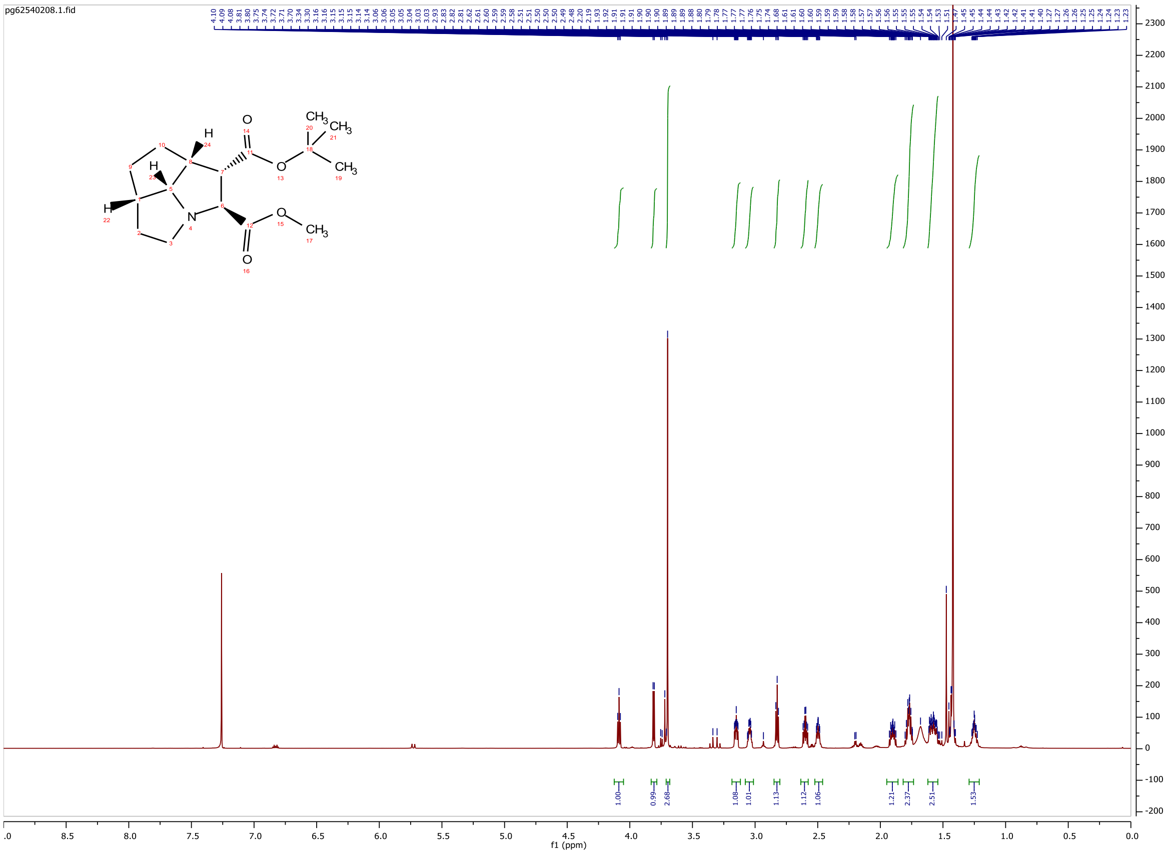

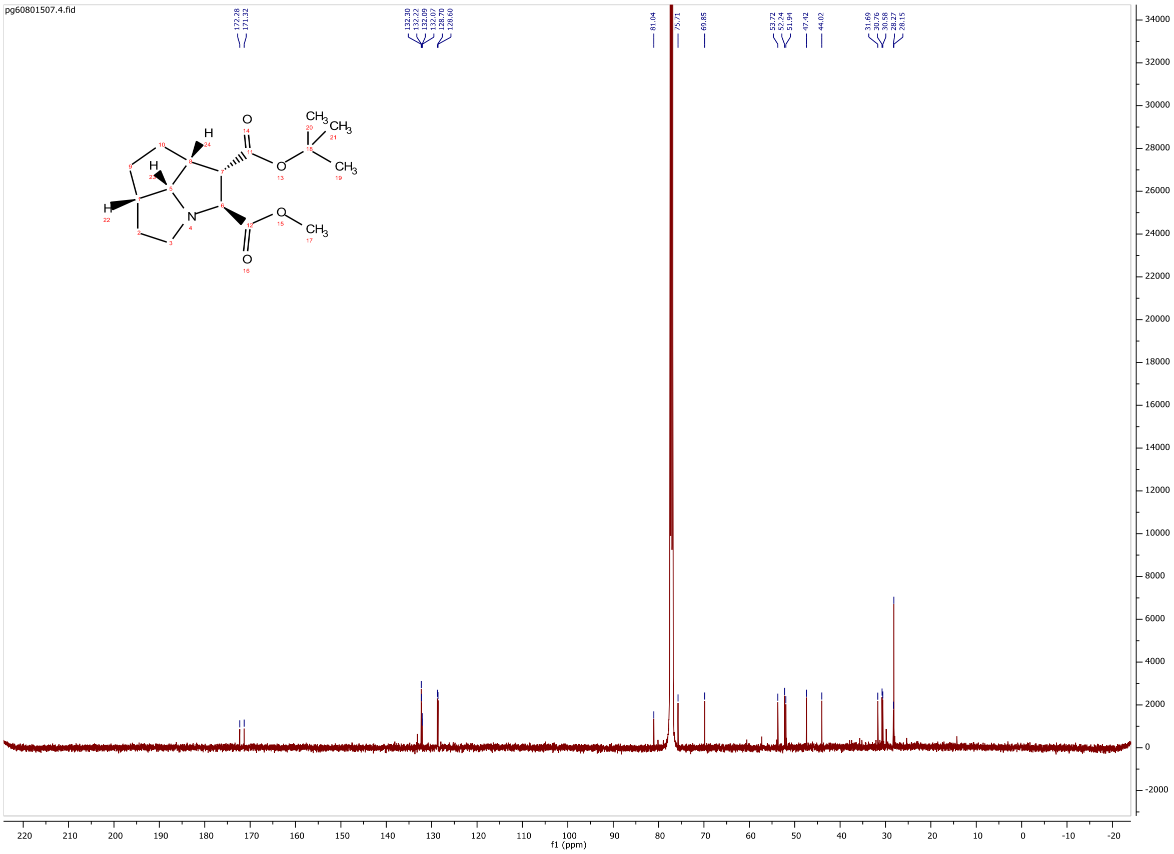

NMR spectra for 8a.

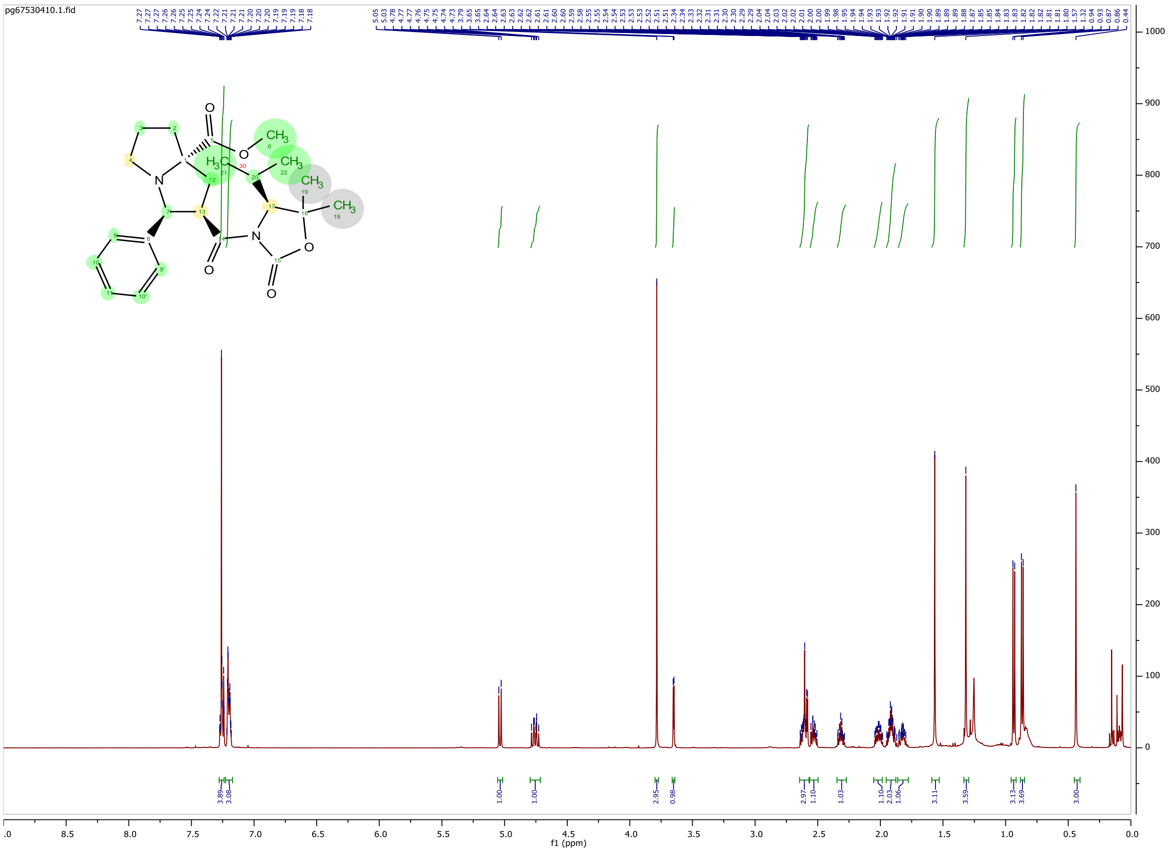

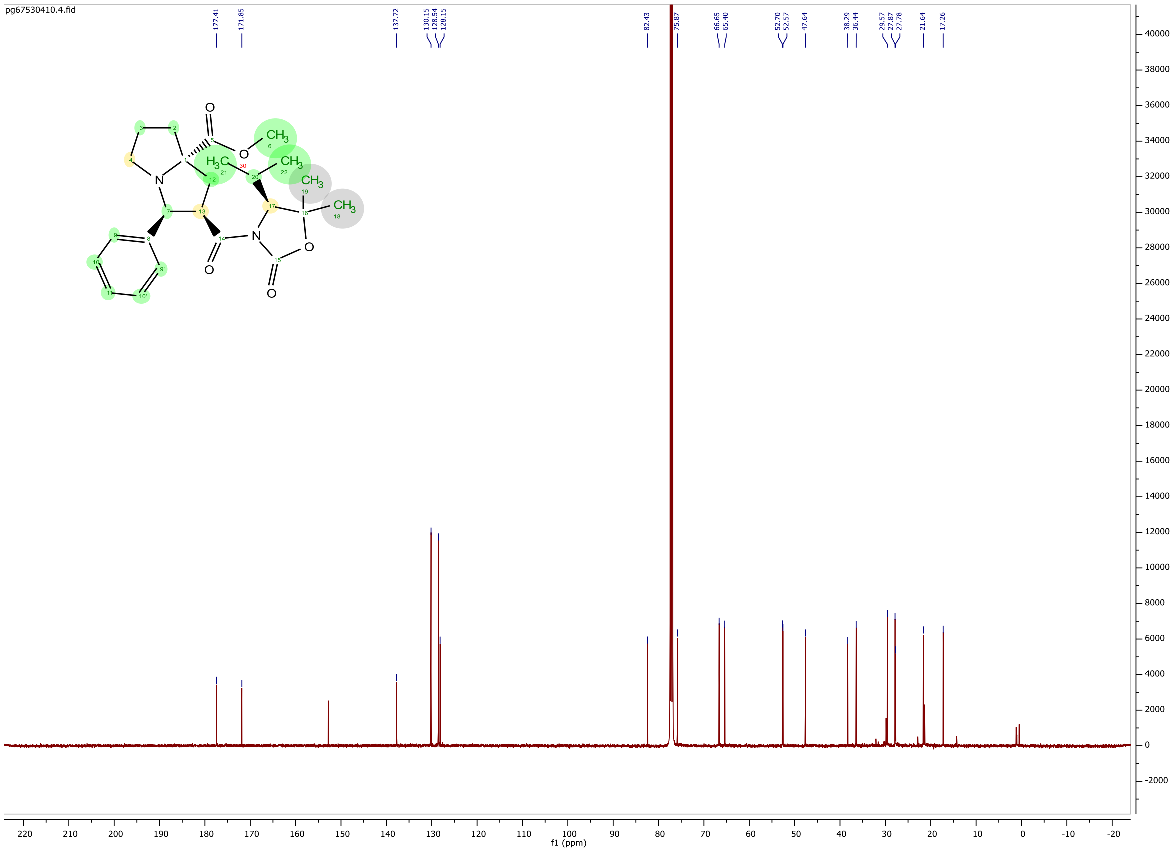

NMR spectra for 8b.

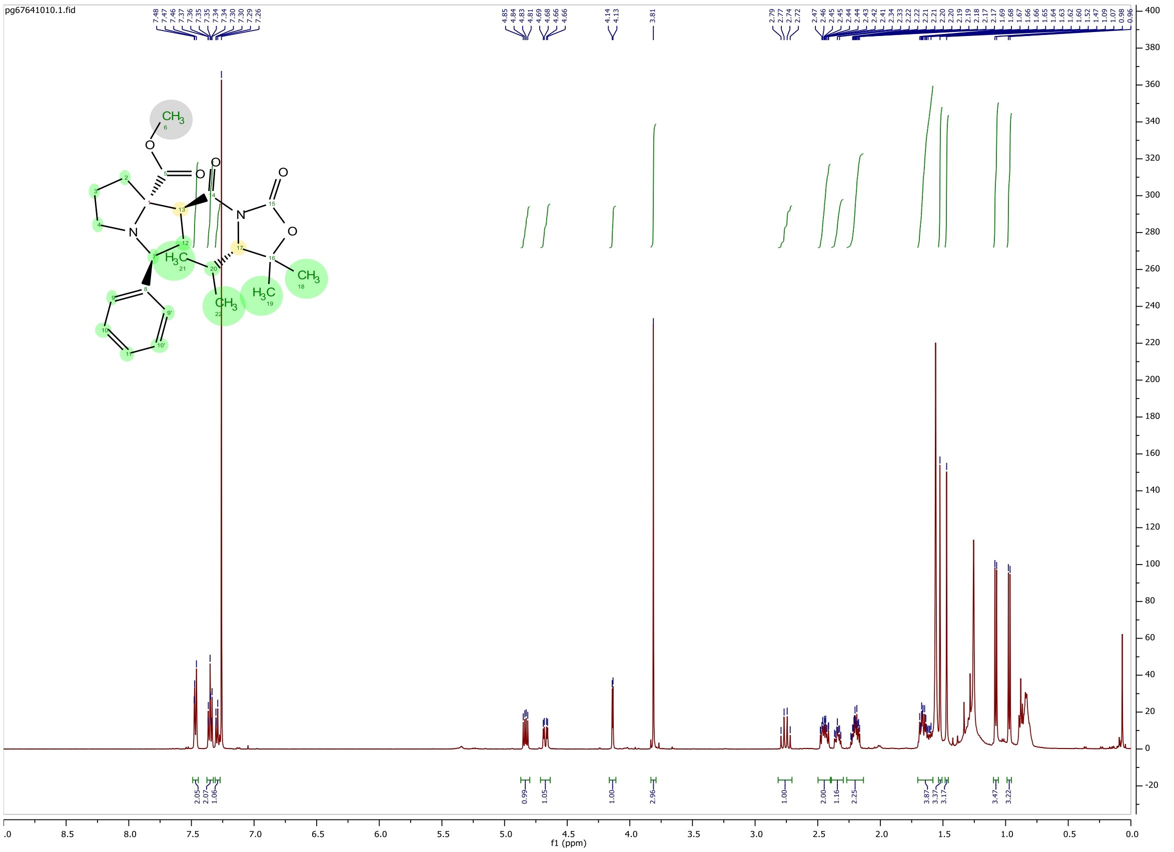

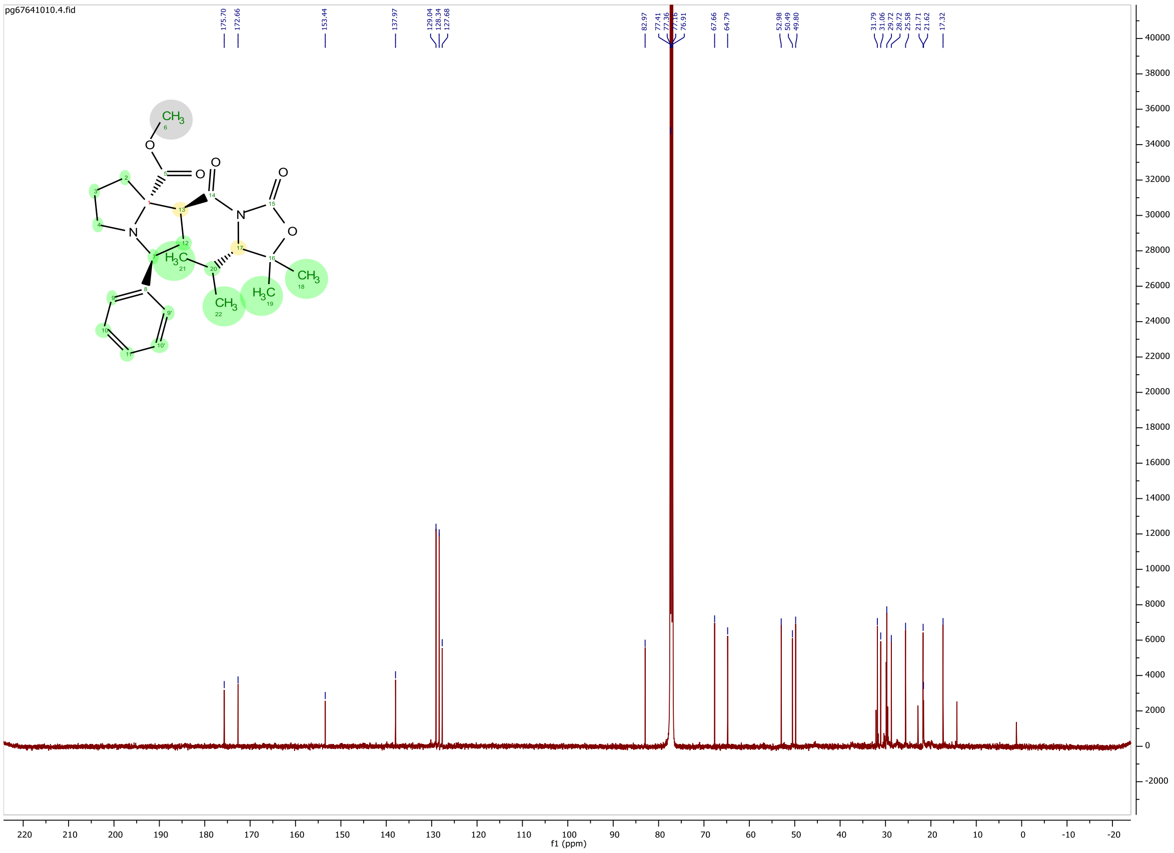

7. Crystallographic Data

X-ray diffraction data has been made available in the Cambridge Crystallographic Data Centre as CCDC 2056517 (**5i**), 2056518 (**3h**) and 2056519 (**8a**).

Data for 3h

Crystal data

Chemical formula	$C_{23}H_{25}NO_4$
M_r	379.44
Crystal System, space group	Monoclinic, $P2_1/c$
Temperature (K)	150
a, *b*, *c* (Å)	10.0439 (1), 18.5010 (3), 10.7555 (2)
α, β, γ (°)	90, 90.7333 (15), 90
V (Å^3)	1998.45 (2)
Z	4
Radiation type	Cu $K\alpha$
U (mm^{-1})	0.695
Crystal size (mm)	0.20 × 0.10 × 0.05

Data collection

Diffractometer	Unknown
Absorption correction	Multi-scan
T_{min}, T_{max}	0.980, 1.000
No. of measured independent and observed	14017, 2947, 2760
R_{int}	–

$(\sin \theta/\lambda)_{max}$ (Å^{-1})	–

Refinement

$R[F^2 > 2\sigma(F^2)]$, $wR(F^2)$, S	0.0358, 0.0915, 0.985
No. of reflections	4219
No. of parameters	254
No. restraints	3
H-atom treatment	H atoms treated by a mixture of independent and constrained refinement
$\Delta\rho_{max}$, $\Delta\rho_{min}$, (e Å□ ^{3})	–
Absolute structure	–
Structure parameter	–

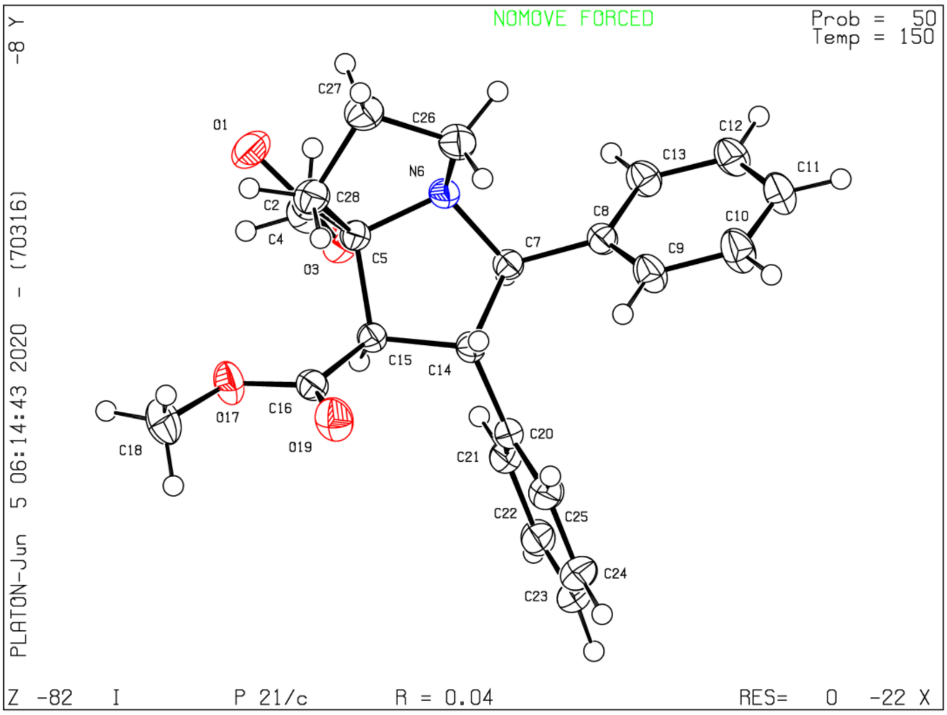

Data for 5i

Crystal data

Crystal data	
Chemical formula	$C_{10.50}H_{10.50}N_{1.25}O_{2.75}$
M_r	198.21
Crystal system, space group	Orthorhombic, $P2_12_12_1$
Temperature (K)	150
a, *b*, *c* (Å)	8.2586 (2), 10.4558 (3), 44.1264 (11)
V (Å^3)	3810.33 (17)
Z	16
Radiation type	Cu $K\alpha$
U (mm^{-1})	0.84
Crystal size (mm)	0.40 × 0.20 × 0.10

Data collection

Diffractometer	Oxford Diffraction SuperNova
Absorption correction	Multi-scan
	CrysAlis PRO (Rigaku Oxford Diffraction, 2017)
T_{min}, T_{max}	0.81, 1.00
No. of measured, independent and observed [I > 2.0σ(I)] reflections	24644, 7919, 7144
R_{int}	0.034
$(\sin \theta/\lambda)_{max}$ (Å^{-1})	0.633

Refinement

$R[F^2 > 2\sigma(F^2)]$, $wR(F^2)$, S	0.040, 0.082, 5.06
No. of reflections	7919
No. of parameters	649
H-atom treatment	Only H-atom coordinates refined
$\Delta\rho_{max}$, $\Delta\rho_{min}$ (e Å^{-3})	0.633

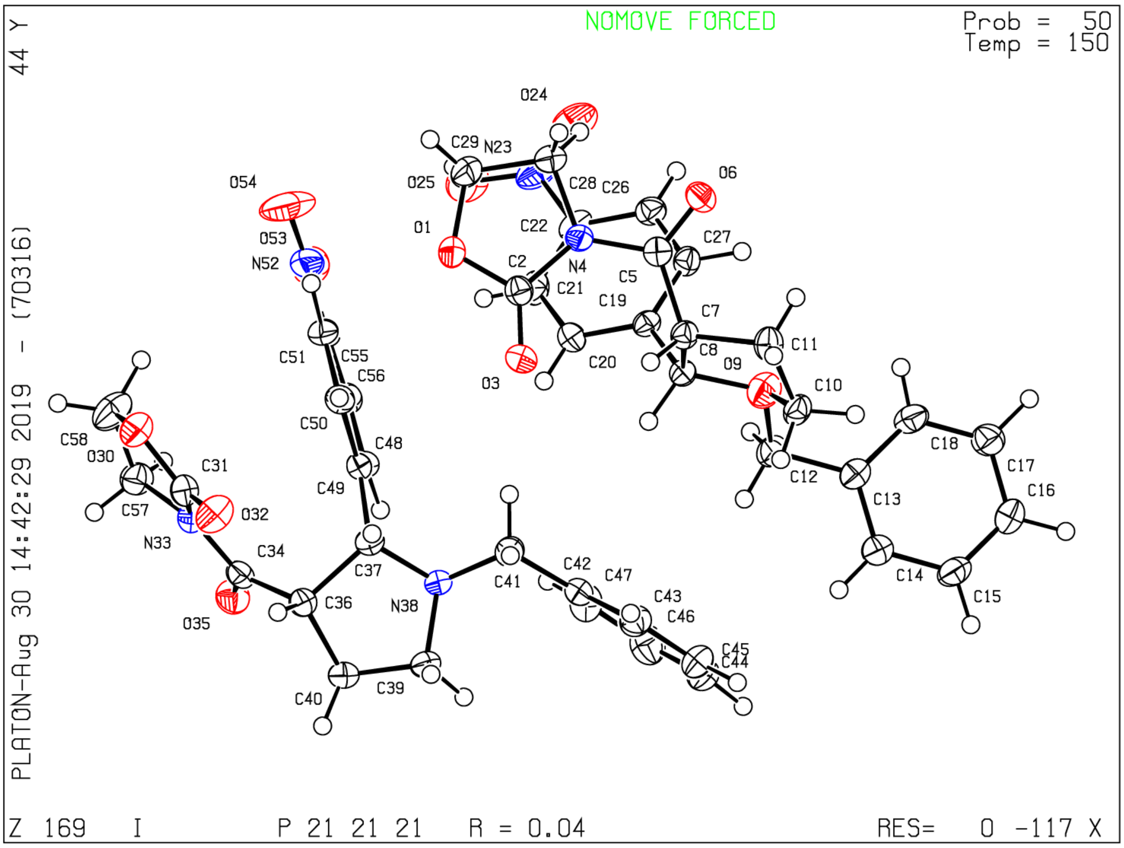

Data for 8a

Crystal data

Crystal data	
Chemical formula	$C_{12}H_{16}N_1O_{2.50}$
M_r	214.26
Crystal system, space group	Orthorhombic, $P2_12_12_1$
Temperature (K)	150
a, *b*, *c* (Å)	9.8584 (1), 10.4074 (2), 21.9655 (4)
V (Å^3)	2253.67 (6)
Z	8
Radiation type	Cu $K\alpha$
U (mm^{-1})	--
Crystal size (mm)	0.20 × 0.20 × 0.15

Data collection

Diffractometer	Oxford Diffraction SuperNova
Absorption correction	Multi-scan
T_{min}, T_{max}	0.870, 0.900
No. of measured, independent and observed [I > 2.0σ(I)] reflections	--
R_{int}	––
(sin θ/λ)$_{max}$ (Å^{-1})	––

Refinement

$R[F^2 > 2\sigma(F^2)]$, $wR(F^2)$, S	0.0329, 0.0833, 0.902
No. of reflections	--
No. of parameters	281
H-atom treatment	--
$\Delta\rho_{max}$, $\Delta\rho_{min}$ (e Å^{-3})	——

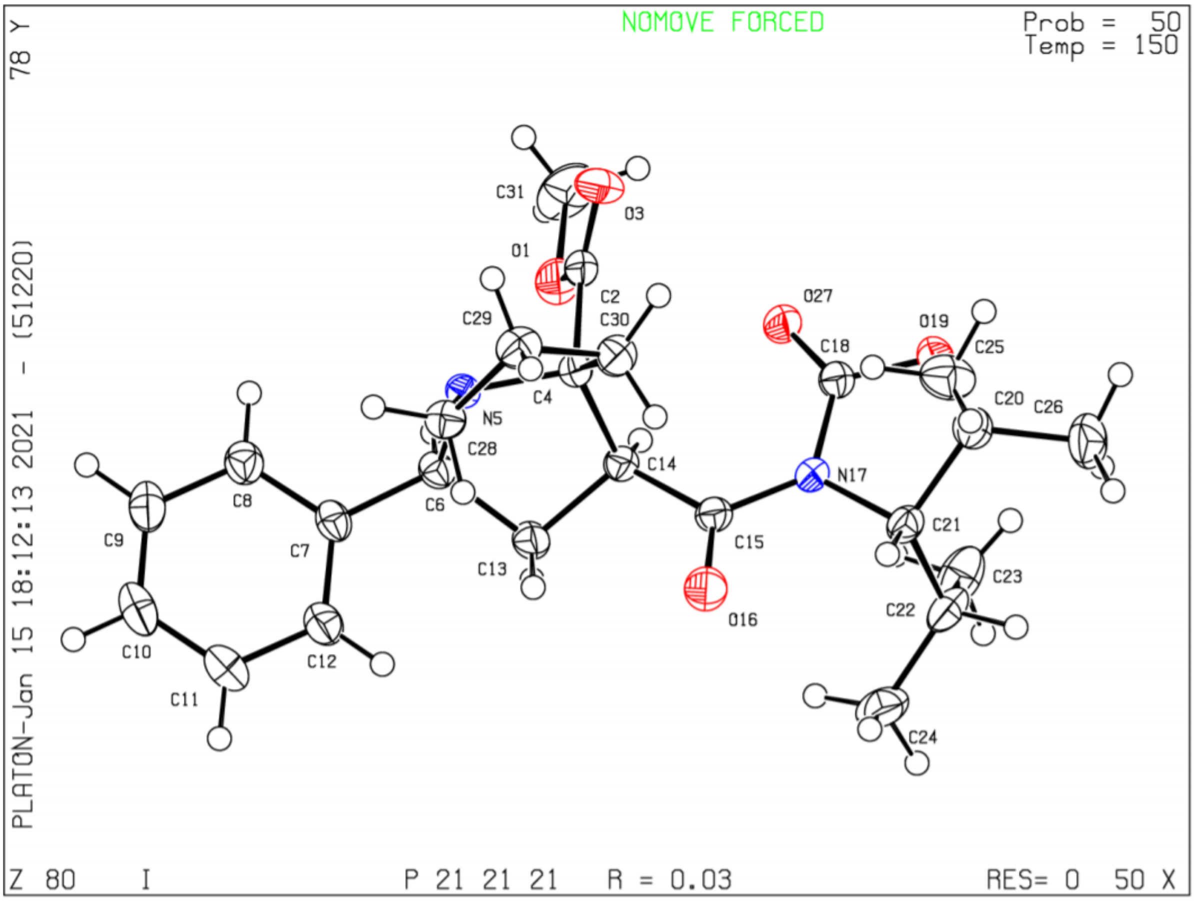

8. Computational details

Computational methods.

All calculations reported in this paper were performed using the Amsterdam Density Functional (ADF) software.[9] Equilibrium structures and transition structure geometries were optimized using the BP86 functional[10] and the TZ2P basis set.[11] The exchange-correlation (XC) functional has been proven to be accurate in calculating the relative trends in activation and reaction energies for cycloadditions.[12] This level is referred to as BP86/TZ2P. All stationary points have been verified, through vibrational analysis, to be minima (zero imaginary frequencies) or transition structures (one imaginary frequency). The character of the normal mode associated with the imaginary frequency has been analyzed to ensure it resembles the reaction under consideration. Optimized structures were illustrated using CYLview.[13] Potential energies were refined by means of single point calculations using the M06-2X functional[14] and the TZ2P basis set.[11] Solvent effects of toluene were accounted for using the COnducter-like Screen MOdel (COSMO) of solvation.[15] This level is denoted COSMO(toluene)-M06-2X/TZ2P//BP86/TZ2P. The reported Gibbs free energies in solution are calculated by adding thermal corrections computed at 298 K from vibrational frequencies obtained through numerical differentiation of the analytical gradient at BP86/TZ2P and a standard concentration (1 mol L^{-1}) to the total electronic energy at COSMO(toluene)-M06-2X/TZ2P.

Quantitative analyses of the activation barriers associated with the cycloaddition were obtained by means of the activation strain model (ASM), which involves decomposing the electronic energy of the transition structure $\Delta E^{\ddagger}$ into the strain $\Delta E^{\ddagger}_{\text{strain}}$ associated with the structural deformation of the reactants from their equilibrium geometry and the interaction $\Delta E^{\ddagger}_{\text{int}}$ between the deformed reactants [Eq. 1].[16] The $\Delta E^{\ddagger}_{\text{strain}}$ is determined by the rigidity of the reactants and by the extent to which they must deform to achieve the geometry of the transition structure. The $\Delta E^{\ddagger}_{\text{int}}$ is usually stabilizing and is related to the electronic structure of the reactants and how they are mutually oriented over the course of the reaction.

$$\Delta E^{\ddagger} = \Delta E^{\ddagger}_{\text{strain}} + \Delta E^{\ddagger}_{\text{int}} \quad (1)$$

The interaction energy between the deformed reactants can be further analyzed in terms of quantitative Kohn-Sham molecular orbital theory (KS-MO) together with a canonical energy decomposition analysis (EDA).[17] The EDA decomposes the $\Delta E^{\ddagger}_{\text{int}}$ into the following three physically meaningful energy terms [Eq. 2]:

$$\Delta E^{\ddagger}_{\text{int}} = \Delta V^{\ddagger}_{\text{elstat}} + \Delta E^{\ddagger}_{\text{Pauli}} + \Delta E^{\ddagger}_{\text{oi}} \quad (2)$$

Herein, $\Delta V^{\ddagger}_{\text{elstat}}$ is the classical electrostatic interaction between the unperturbed charge distributions of the (deformed) reactants and is usually attractive. The Pauli repulsion, $\Delta E^{\ddagger}_{\text{Pauli}}$, comprises the destabilizing interaction between occupied closed-shell orbitals of both fragments due to the Pauli principle. Finally, the orbital interaction energy, $\Delta E^{\ddagger}_{\text{oi}}$, accounts for polarization and charge transfer between the fragments, such as HOMO–LUMO interactions. A detailed, step-by-step, guide on how to perform and interpret the ASM and EDA can be found in Ref. 16a.

Cartesian coordinates and energies.

Table S1. Cartesian coordinates (in Å), energies (in kcal mol^{-1}), and number of imaginary frequencies of all stationary points, computed at COSMO(toluene)-M06-2X/TZ2P//BP86/TZ2P.

TSOMe1

COSMO(toluene)-M06-2X/TZ2P//BP86/TZ2P

E = -10546.82

G = -10318.39

$\boldsymbol{N}_{\textbf{imag}}$ = 1, 281*i* cm^{-1}

C	0.01054977	-1.29732312	-2.15866440
C	1.06329817	0.63117079	-1.33381145
C	2.11545693	-0.12918397	-2.10547621
C	1.28260536	-1.08739248	-2.97948724
H	0.14831767	-2.09681552	-1.41552118
H	-0.88383846	-1.51708151	-2.75130140
H	2.75697215	0.53799968	-2.69106352
H	2.77171447	-0.68687207	-1.41739229
H	1.03447382	-0.60929872	-3.93674774
H	1.79376339	-2.03355257	-3.19015473
C	1.14374129	2.07687799	-1.11379665
O	0.21223966	2.85712725	-0.96715965

O	2.45644166	2.48383044	-1.10527811
C	2.63561735	3.89753479	-0.87558923
H	2.17882228	4.19542782	0.07580743
H	3.71786200	4.05273250	-0.85293267
H	2.17972517	4.48189066	-1.68426270
C	-1.20931732	0.31567955	-0.64944208
C	-2.53533586	-0.32064003	-0.75434337
C	-2.76154426	-1.71048666	-0.76825415
C	-3.65915331	0.52892909	-0.77148001
C	-4.05579552	-2.22435610	-0.82951085
H	-1.92455547	-2.40306732	-0.70271742
C	-4.95220513	0.01450187	-0.83285309
H	-3.50427707	1.60803400	-0.74558783
C	-5.15710375	-1.36641646	-0.86770395
H	-4.20550270	-3.30421346	-0.83520487
H	-5.80355974	0.69511213	-0.85374732
H	-6.16761172	-1.77211470	-0.91381417
H	-1.23165640	1.38258915	-0.43323221
N	-0.15883250	0.00258599	-1.46605555
C	1.10180454	0.17446316	1.06631598
C	-0.26792836	-0.10863125	1.24545151
H	1.40992200	1.18505776	1.33856862
H	-0.59085990	-1.14674366	1.32700980
C	2.16774297	-0.82356597	1.06920176
C	3.51125632	-0.39612799	1.15516314
C	1.93800628	-2.21580172	1.01435648
C	4.56488558	-1.30663484	1.19179260
H	3.71808389	0.67388712	1.19686881
C	2.99210651	-3.12572479	1.04782026
H	0.91675589	-2.59507335	0.96919978
C	4.31430401	-2.68011021	1.13817095
H	5.58966105	-0.94134477	1.26628596
H	2.77991507	-4.19503605	1.01402372
H	5.13655212	-3.39442504	1.17067713
C	-1.06358474	0.82655855	2.06854914
O	-0.82999383	2.00728811	2.26410932
O	-2.15072243	0.18472019	2.60607627
C	-2.99221648	1.01736386	3.42961964
H	-3.80782856	0.36958792	3.76276953
H	-2.43002111	1.40398627	4.28868391
H	-3.38233852	1.86513569	2.85343474

TSOMe2

COSMO(toluene)-M06-2X/TZ2P//BP86/TZ2P

E = -10550.67

G = -10321.24

N_{imag} = 1, 290*i* cm^{-1}

C	-1.22289195	1.76498498	-1.80849935
C	0.82129373	1.38232930	-0.68451306
C	1.16122294	1.91922845	-2.06490860
C	-0.14941474	2.59071882	-2.51443524
H	-1.46516521	0.85423305	-2.37349341
H	-2.14159844	2.32011848	-1.59084090
H	2.01178846	2.60644358	-2.04141210
H	1.42567870	1.09462579	-2.74499602
H	-0.18070813	3.63156195	-2.16408240
H	-0.28369716	2.58975860	-3.60226499
C	1.67332238	1.66404578	0.48151771
O	1.35146735	1.59737238	1.66270375
O	2.93498878	2.00314292	0.08312348
C	3.87276784	2.21615200	1.16111047
H	3.98615263	1.30015276	1.75196039
H	4.81552722	2.47927729	0.67412928
H	3.53201697	3.02936591	1.81294301
C	-1.13751085	0.67502842	0.47577876
C	-2.58214428	0.60513762	0.73976328
C	-3.59124349	0.46998001	-0.23538445
C	-2.97549989	0.62653643	2.09422035
C	-4.93322717	0.39154645	0.13530395
H	-3.33104236	0.38843772	-1.28723216
C	-4.31580496	0.54738897	2.46086214
H	-2.20851985	0.71878784	2.86405728
C	-5.30456412	0.43572283	1.48054616
H	-5.69608992	0.28741380	-0.63672890
H	-4.59005474	0.57552043	3.51549565
H	-6.35549013	0.37550333	1.76303772
H	-0.51806669	0.68278845	1.36875881
N	-0.56194316	1.38718705	-0.53246900
C	1.02587018	-0.87246545	-0.64080980
C	-0.18791993	-1.26732019	-0.02667748
H	1.00539755	-0.86603214	-1.73286355
H	-0.16163542	-1.69198642	0.97568261

C	2.34308812	-1.18069796	-0.06426473
C	3.46009149	-1.27989800	-0.91833514
C	2.54964173	-1.41040573	1.31081427
C	4.72234378	-1.60485081	-0.42733276
H	3.32386786	-1.11129955	-1.98769015
C	3.81277556	-1.73521156	1.80208491
H	1.71487390	-1.33091578	2.00574904
C	4.90694359	-1.83615890	0.93829819
H	5.56544823	-1.68404263	-1.11436241
H	3.94359750	-1.91370542	2.86995896
H	5.89185017	-2.09763584	1.32515771
C	-1.25758657	-1.79849677	-0.87777418
O	-1.45382253	-1.53934661	-2.06124947
O	-2.05065322	-2.66876759	-0.17774210
C	-3.11793928	-3.26818850	-0.93845838
H	-2.71737762	-3.81694778	-1.79961178
H	-3.62090097	-3.94974137	-0.24676105
H	-3.81691195	-2.50299148	-1.29702912

TSOMe3

COSMO(toluene)-M06-2X/TZ2P//BP86/TZ2P

E = -10546.28

G = -10317.11

$\boldsymbol{N}_{\mathbf{imag}}$ = 1, 300*i* cm^{-1}

C	-0.36704465	-1.16808564	2.21611056
C	1.55146566	-0.87741590	0.88197630
C	2.00328455	-0.74040119	2.32445080
C	0.85173928	-1.39747106	3.11072608
H	-0.82965627	-0.18774912	2.40405241
H	-1.13821910	-1.94133727	2.30213942
H	2.97146797	-1.22034599	2.49865045
H	2.11772907	0.31945010	2.59835467
H	1.03665737	-2.47455407	3.21950340
H	0.71230281	-0.97227280	4.11133662
C	2.44051983	-1.42806581	-0.16027116
O	2.10315898	-2.03459138	-1.16568820
O	3.75077294	-1.18944389	0.15206482
C	4.68834403	-1.66653849	-0.83897532
H	4.47006161	-1.21635062	-1.81434628
H	5.67332353	-1.36114756	-0.47533697
H	4.63237082	-2.75824448	-0.92785839

C	-0.50495555	-1.04444373	-0.30747769
C	-1.91371532	-1.42885687	-0.47063667
C	-2.95351250	-1.14446109	0.43721770
C	-2.25294631	-2.08656209	-1.67195415
C	-4.26282071	-1.54345974	0.17099498
H	-2.75431928	-0.57830299	1.34346154
C	-3.56162564	-2.47937577	-1.93770231
H	-1.46513987	-2.30232194	-2.39467021
C	-4.57402096	-2.21695065	-1.01126583
H	-5.04929149	-1.31036908	0.88888953
H	-3.79161272	-2.99600363	-2.86968709
H	-5.59953066	-2.52512348	-1.21460271
H	0.11503299	-1.26530209	-1.17222461
N	0.20934501	-1.19680173	0.85222978
C	1.15719237	1.23821941	-0.03667596
C	-0.11971888	1.08480158	-0.62088660
H	1.24720630	1.70140532	0.94499032
H	-0.11156863	0.98287897	-1.70823323
C	-1.32480153	1.75084239	-0.08861656
C	-2.44515633	1.90778334	-0.92788983
C	-1.41420303	2.28119645	1.21410105
C	-3.59797412	2.55760016	-0.49018851
H	-2.40018782	1.51665487	-1.94508972
C	-2.56908474	2.92376306	1.65633093
H	-0.56103333	2.21197306	1.88975322
C	-3.67095578	3.06632952	0.80766949
H	-4.44455945	2.66681172	-1.16858556
H	-2.60485859	3.32947388	2.66816292
H	-4.57044477	3.57587289	1.15265313
C	2.32519024	1.43777497	-0.89929295
O	2.44247066	1.10565696	-2.07005374
O	3.32242239	2.09256400	-0.21095897
C	4.50155099	2.37488072	-0.98635772
H	4.25287109	2.98722894	-1.86222186
H	5.17187418	2.91876325	-0.31449024
H	4.97441000	1.44665209	-1.33016911

TSOMe4

COSMO(toluene)-M06-2X/TZ2P//BP86/TZ2P

E = -10550.66

G = -10321.66

$\boldsymbol{N}_{\mathbf{imag}}$ = 1, 298i cm^{-1}

C	0.75398563	-2.72257519	-0.06087138
C	1.76252289	-0.79324596	-0.92386158
C	2.88602726	-1.74738957	-0.60331219
C	2.16540540	-3.10506302	-0.50380731
H	0.69685676	-2.61238369	1.02998728
H	-0.03168098	-3.40143129	-0.40770820
H	3.67293379	-1.72521712	-1.36461298
H	3.35185047	-1.48249292	0.35841289
H	2.13486439	-3.59114387	-1.48846886
H	2.64095296	-3.79148734	0.20524431
C	1.91676027	0.37639927	-1.78365205
O	1.03355398	1.00383965	-2.36350149
O	3.24614980	0.68696719	-1.91596930
C	3.51407743	1.84265079	-2.73444126
H	3.04107404	2.73631632	-2.30889169
H	4.60215889	1.95005066	-2.73641565
H	3.13723977	1.69337315	-3.75357355
C	-0.62079942	-0.67663227	-0.63109258
C	-1.93060983	-1.34051273	-0.45693920
C	-2.24534911	-2.20561920	0.60851090
C	-2.95268188	-1.01872356	-1.36974349
C	-3.52143830	-2.75449663	0.72994485
H	-1.49809729	-2.43564335	1.36606810
C	-4.22799771	-1.56646481	-1.24636908
H	-2.73430324	-0.33038708	-2.18656453
C	-4.51677336	-2.44323732	-0.19883743
H	-3.74246061	-3.42075189	1.56412528
H	-4.99994148	-1.30471615	-1.97015709
H	-5.51398898	-2.87162682	-0.09899439
H	-0.61845394	0.13377985	-1.35767360
N	0.55714952	-1.38391743	-0.68345375
C	1.09257629	0.91583520	0.95056246
C	-0.28066986	0.60169691	0.95773001
H	1.44304467	1.85385388	0.52483698
H	-0.56531725	-0.12177794	1.72689220
C	-1.30401814	1.64980535	0.70694935
C	-2.45899881	1.68765006	1.50562144
C	-1.15960271	2.61884213	-0.30115694
C	-3.42708078	2.67563922	1.32274570
H	-2.59061607	0.93841720	2.28735809

C	-2.12765536	3.60520149	-0.48407338
H	-0.29068421	2.58617020	-0.95962829
C	-3.26413644	3.64191730	0.32886604
H	-4.31171695	2.68941128	1.96005848
H	-1.99648581	4.34814274	-1.27149391
H	-4.01929633	4.41451735	0.18344217
C	2.00959537	0.22072003	1.83330222
O	1.79936639	-0.82703870	2.44486969
O	3.20862919	0.89270666	1.93767710
C	4.14505220	0.32043295	2.86883859
H	5.03648686	0.95190653	2.81238694
H	3.73349935	0.32836044	3.88637470
H	4.39151372	-0.71455342	2.60062644

TSOx1

COSMO(toluene)-M06-2X/TZ2P//BP86/TZ2P

E = -11789.69

G = -11542.01

N_{imag} = 1, 271*i* cm^{-1}

C	-1.09417693	1.56433450	-2.17724519
C	-1.83992913	-0.49527258	-1.32346806
C	-3.07947534	0.28930461	-1.69270949
C	-2.53966706	1.38131329	-2.63792925
H	-1.03289376	2.26351516	-1.32926385
H	-0.40392113	1.89868715	-2.95906022
H	-3.83962104	-0.34464962	-2.16140011
H	-3.53817328	0.73292790	-0.79480185
H	-2.55848135	1.02851790	-3.67793907
H	-3.10748720	2.31687090	-2.58248189
C	-1.82833005	-1.96322048	-1.30897871
O	-0.87623417	-2.69942937	-1.52804432
O	-3.07916335	-2.44440469	-1.00859492
C	-3.15956169	-3.88410735	-0.94079745
H	-2.45855984	-4.27534310	-0.19369932
H	-4.19251322	-4.10391391	-0.65715815
H	-2.92394377	-4.33123308	-1.91426249
C	0.52247349	-0.13585354	-1.27035094
C	1.76060497	0.55213038	-1.67907458
C	1.97347180	1.94119240	-1.59642170
C	2.82433614	-0.25471700	-2.13174118
C	3.18922836	2.50102844	-1.98541695

H	1.20198072	2.58999845	-1.18755100
C	4.03798442	0.30718245	-2.52277377
H	2.68057612	-1.33427923	-2.18751314
C	4.22357173	1.69008885	-2.45757432
H	3.33446640	3.57799935	-1.90258536
H	4.84077093	-0.33715989	-2.88208044
H	5.17269731	2.13250132	-2.75987771
H	0.61769547	-1.21310062	-1.14473302
N	-0.71427477	0.20589419	-1.72494503
C	-1.27965232	-0.33457862	0.98040860
C	0.06379247	0.09397867	0.87104246
H	-1.41823791	-1.39662115	1.18975922
H	0.29139394	1.15185844	0.97763943
C	-2.37500508	0.52897162	1.42688022
C	-3.60964263	-0.05544160	1.78275258
C	-2.26261113	1.93036512	1.54924453
C	-4.67523152	0.71734155	2.23905095
H	-3.72227295	-1.13671225	1.69572481
C	-3.32996356	2.70258930	2.00251420
H	-1.31788326	2.42212617	1.31733147
C	-4.54410038	2.10373032	2.35109309
H	-5.61370510	0.23371324	2.51203443
H	-3.20854987	3.78221845	2.09720623
H	-5.37467597	2.71009842	2.71181011
C	1.11935890	-0.80820336	1.34066901
O	1.04515940	-2.03415155	1.38832699
C	3.42753106	-1.10230219	2.19768462
C	4.57929082	-0.10729644	2.36639302
H	3.62954716	-1.87897643	1.45311699
H	5.23588932	-0.07678956	1.48584825
C	2.65311477	1.12074364	2.02500749
O	1.95816988	2.10519016	1.91541185
O	3.94107393	1.18703804	2.49580037
N	2.35164424	-0.21841563	1.74667835
H	3.14719250	-1.59718725	3.13840973
H	5.17852386	-0.28003361	3.26612954

TSOx2

COSMO(toluene)-M06-2X/TZ2P//BP86/TZ2P

E = -11793.40

G = -11545.10

N_{imag} = 1, 268i cm^{-1}

C	-0.29560838	2.33910067	-1.75674688
C	1.56072764	1.38064200	-0.61670960
C	2.04678294	1.87165881	-1.97779031
C	0.97330948	2.88773899	-2.40366820
H	-0.74429347	1.54197787	-2.36521412
H	-1.04736777	3.10249227	-1.52997361
H	3.04959118	2.30287353	-1.92413030
H	2.08511221	1.03551995	-2.69223533
H	1.20602229	3.88180453	-1.99736291
H	0.87169332	2.97610828	-3.49166985
C	2.42427685	1.55085134	0.57917872
O	2.05600056	1.55735916	1.74616846
O	3.73296729	1.67342823	0.22151334
C	4.65901584	1.74407972	1.32933398
H	4.59656186	0.83081089	1.93180389
H	5.64732785	1.83780102	0.87196102
H	4.43739272	2.61261133	1.96050417
C	-0.54236328	1.28038951	0.52371021
C	-1.96955003	1.50800600	0.73005442
C	-2.94201799	1.64441249	-0.28439445
C	-2.40837064	1.54290682	2.07249648
C	-4.28302897	1.84522563	0.04018476
H	-2.66174016	1.55597997	-1.32988238
C	-3.74696858	1.74496920	2.39056299
H	-1.67562432	1.41274165	2.86910130
C	-4.69248919	1.90674150	1.37406760
H	-5.01644039	1.95105990	-0.75978009
H	-4.05466170	1.77518761	3.43562450
H	-5.74179609	2.06955868	1.61988883
H	0.03700159	1.06081755	1.41434298
N	0.20986070	1.75657479	-0.48705224
C	1.33132923	-0.70521842	-0.66302064
C	0.10920034	-0.99341700	-0.00111870
H	1.24830618	-0.69102420	-1.75311577
H	0.10429567	-1.41100793	1.00106465
C	2.60480610	-1.29645743	-0.18355665
C	3.62672369	-1.56977086	-1.11098015
C	2.83428007	-1.62754577	1.16463125
C	4.82868579	-2.15361566	-0.71348829
H	3.46514245	-1.33270239	-2.16376274

C	4.03586827	-2.21171205	1.56284216
H	2.06542882	-1.43133822	1.91054690
C	5.04000280	-2.47837095	0.62811045
H	5.59971673	-2.36312336	-1.45556889
H	4.18566584	-2.46825072	2.61209584
H	5.97502923	-2.94252683	0.94200026
C	-1.08449091	-1.18522066	-0.79158265
O	-1.23145996	-0.84308784	-1.97608825
C	-3.37656125	-2.12723597	-0.99195206
C	-4.37176549	-2.58986061	0.07662193
H	-3.14246104	-2.91141810	-1.72659974
H	-4.96006492	-3.46354315	-0.22249850
C	-2.30768015	-2.39985786	1.09403909
O	-1.49606851	-2.44531584	1.98966026
O	-3.55748535	-2.96373561	1.21336692
N	-2.20011103	-1.82779752	-0.17675673
H	-3.71005818	-1.23846307	-1.53639407
H	-5.04604512	-1.78107702	0.39160796

TSOx3

COSMO(toluene)-M06-2X/TZ2P//BP86/TZ2P

E = -11787.79

G = -11539.62

$\boldsymbol{N}_{\mathbf{imag}}$ = 1, 300*i* cm^{-1}

C	-1.41488470	-0.32832372	2.45961559
C	0.67025692	-0.79277599	1.48033491
C	0.96972089	-0.15815859	2.82073810
C	-0.36655181	-0.31292225	3.57411679
H	-1.76115781	0.68344262	2.20562302
H	-2.28749133	-0.95373997	2.67792603
H	1.80670068	-0.64936959	3.32740351
H	1.24920996	0.89764732	2.69370478
H	-0.38465018	-1.26723198	4.11727272
H	-0.55000848	0.49190241	4.29467442
C	1.58589609	-1.72612033	0.81271463
O	1.29548481	-2.59018877	-0.00266298
O	2.86871561	-1.54354283	1.26684414
C	3.82750939	-2.46253101	0.70340624
H	3.81189775	-2.41480349	-0.39187762
H	4.79751969	-2.14945599	1.09949917
H	3.60291940	-3.49115969	1.01197164

C	-1.21615468	-1.06881279	0.04308890
C	-2.62942767	-1.41986048	-0.19730551
C	-3.74472452	-0.79968990	0.39918382
C	-2.87104962	-2.42725240	-1.15404145
C	-5.04102911	-1.20628142	0.08277437
H	-3.61188371	0.02654049	1.09280093
C	-4.16602314	-2.82599766	-1.47422535
H	-2.02069100	-2.90948024	-1.63752422
C	-5.25962586	-2.22242630	-0.84858964
H	-5.88765872	-0.71219972	0.55958628
H	-4.32151872	-3.61515230	-2.21001370
H	-6.27490379	-2.53452518	-1.09310925
H	-0.52796230	-1.63908043	-0.57769473
N	-0.68164968	-0.90152506	1.30429902
C	0.67357819	0.92940421	-0.37640280
C	-0.63630307	0.70886983	-0.86606394
H	0.87368509	1.67284147	0.38864759
H	-0.67073098	0.26017615	-1.86180704
C	-1.72100442	1.69110551	-0.62344142
C	-2.83348567	1.70658210	-1.48516975
C	-1.68091994	2.65128199	0.40628590
C	-3.86387473	2.63273643	-1.32473097
H	-2.88089461	0.98304565	-2.29973290
C	-2.71371916	3.57207184	0.57326685
H	-0.81873299	2.70056157	1.07156571
C	-3.81361747	3.56772072	-0.28994860
H	-4.70855708	2.62410980	-2.01403187
H	-2.65183762	4.31015944	1.37364357
H	-4.61574678	4.29466009	-0.16302410
C	1.79979618	0.53119099	-1.19566671
O	1.74347591	-0.19505654	-2.19220463
C	3.45782323	1.91859662	0.17170519
O	2.77872764	2.41635830	1.04267524
O	4.80252635	2.18705475	0.05857404
C	4.20624718	0.79182968	-1.76056045
H	4.05289956	1.33442498	-2.70471814
H	4.28808874	-0.27394356	-1.99705320
C	5.39007727	1.32937211	-0.94883238
H	6.08877085	1.92934911	-1.54107741
H	5.94147884	0.52951828	-0.43479527
N	3.09225603	1.04046471	-0.84686307

TSOx4

COSMO(toluene)-M06-2X/TZ2P//BP86/TZ2P

E = -11793.29

G = -11544.83

N_{imag} = 1, 286*i* cm^{-1}

C	0.17639247	-2.83459967	0.04833016
C	-0.85523151	-1.13894882	1.29063318
C	-1.90051754	-2.20404530	1.08577069
C	-1.06190535	-3.43914391	0.70832918
H	0.00442028	-2.63511006	-1.01700078
H	1.09168378	-3.42168265	0.17379681
H	-2.51837528	-2.34973839	1.97818229
H	-2.57413761	-1.92402407	0.25961531
H	-0.77767878	-3.99409296	1.61264721
H	-1.58654641	-4.12487485	0.03402630
C	-0.98078207	-0.04672102	2.25359368
O	-0.07938747	0.64956262	2.71282559
O	-2.28789357	0.07565819	2.64103330
C	-2.55792753	1.18801054	3.52279408
H	-2.45740110	2.13062440	2.97128369
H	-3.59162260	1.05193369	3.85185091
H	-1.87089721	1.18119977	4.37633738
C	1.39649779	-0.68053852	0.56630096
C	2.71079887	-1.16614193	0.09508197
C	2.91031150	-1.88317371	-1.10011008
C	3.84481732	-0.81601656	0.85200259
C	4.19073301	-2.26615818	-1.49811910
H	2.06406242	-2.12465939	-1.74123605
C	5.12369509	-1.19956607	0.45340878
H	3.71186664	-0.23811784	1.76683937
C	5.30236611	-1.93268900	-0.72132975
H	4.32107994	-2.81970810	-2.42825202
H	5.98440975	-0.92090554	1.06147941
H	6.30185936	-2.23199436	-1.03645508
H	1.44411490	0.06159957	1.36108535
N	0.33068800	-1.52919190	0.75187386
C	-0.71759188	0.86751215	-0.50406166
C	0.64604068	0.68727469	-0.80647005
H	-1.04860444	1.71646412	0.08661492
H	0.83867894	0.05711149	-1.67894610

C	1.61334034	1.80144005	-0.63788871
C	2.61724016	1.99099166	-1.60212797
C	1.56207128	2.68506800	0.45424378
C	3.52836860	3.04204934	-1.49333151
H	2.67536541	1.30911127	-2.45153147
C	2.47307360	3.73418597	0.56275701
H	0.81376598	2.53716563	1.23350818
C	3.45887653	3.92022677	-0.41085862
H	4.29527760	3.17369798	-2.25715019
H	2.41630346	4.40928740	1.41712145
H	4.16999472	4.74173147	-0.32267087
C	-1.72143098	0.12291327	-1.22031111
O	-1.51407384	-0.84504907	-1.97068556
C	-3.62881777	1.61849977	-0.39636203
O	-3.07768990	2.45103970	0.28682190
O	-4.98731824	1.64331945	-0.61967668
C	-4.09828887	-0.11611149	-1.92157981
H	-4.02382832	-1.20658068	-1.86629071
H	-3.96245471	0.17953719	-2.97239126
C	-5.38865116	0.43749326	-1.31210519
H	-5.82901602	-0.24977064	-0.57527299
H	-6.14408311	0.70769072	-2.05704740
N	-3.08797376	0.52542864	-1.07926637

Azomethine ylide

COSMO(toluene)-M06-2X/TZ2P//BP86/TZ2P

E = -6111.38

G = -5981.32

N_{imag} = 0

C	-0.99133222	0.76721814	2.67722570
C	0.25265637	-1.05111280	1.89059000
C	1.24566773	-0.08425630	2.47294093
C	0.36469727	0.78933634	3.37987552
H	-1.05660402	1.54014543	1.89822281
H	-1.85054670	0.86372170	3.34728631
H	2.04519542	-0.59568623	3.02169572
H	1.73650707	0.52182235	1.68976652
H	0.27466116	0.33107479	4.37395851
H	0.73904848	1.81184872	3.50639817
C	0.55357407	-2.29794146	1.26708176
O	-0.24428078	-3.12203979	0.80387045

O	1.92460503	-2.50592485	1.22904665
C	2.31507675	-3.74885552	0.62202317
H	1.97325180	-3.80199748	-0.41966059
H	3.40825277	-3.76524396	0.66672566
H	1.89634651	-4.60276016	1.17035001
C	-2.17443904	-1.16217766	1.62994925
C	-3.52731155	-0.66756710	1.69206938
C	-3.95763504	0.67991049	1.80004244
C	-4.54267172	-1.65438532	1.57052742
C	-5.31233920	1.00258368	1.82453374
H	-3.23778364	1.49292357	1.82422093
C	-5.89056031	-1.32413631	1.59808156
H	-4.24456491	-2.69774640	1.46261213
C	-6.29043963	0.00981867	1.73464940
H	-5.60704364	2.04966697	1.90192718
H	-6.63801684	-2.11311541	1.51121666
H	-7.34764525	0.27217980	1.75545957
H	-2.01265334	-2.16445577	1.23660330
N	-1.03624778	-0.56849116	2.00953570

Methyl cinnamate

COSMO(toluene)-M06-2X/TZ2P//BP86/TZ2P

E = -4436.62

G = -4353.82

N_{imag} = 0

H	3.04733425	-3.93121960	1.93843356
H	5.23556758	-3.05696870	1.13353803
C	-1.17221870	1.12764875	2.15533106
O	-0.99987500	2.29796424	1.85913532
O	-2.36705020	0.63852973	2.61477133
C	-3.41646514	1.62186727	2.72456664
H	-4.28852608	1.07967428	3.09965847
H	-3.12583526	2.41861714	3.42029212
H	-3.62872284	2.06911694	1.74565562
C	1.07860698	0.29609242	1.65928863
C	-0.17551953	0.04450012	2.08234245
H	1.29826261	1.33143077	1.38138212
H	-0.50959055	-0.94675734	2.38700028
C	2.18249764	-0.64854020	1.53005648
C	3.42787417	-0.17264931	1.07532563
C	2.06772757	-2.01958072	1.83820752

C	4.51790365	-1.02899224	0.93359256
H	3.53154324	0.88584521	0.83268257
C	3.15527692	-2.87393449	1.69592974
H	1.11741731	-2.41929871	2.19117499
C	4.38570690	-2.38350083	1.24356197
H	5.47226974	-0.63865142	0.58030840

***N*-enoyl oxazolidinone**

COSMO(toluene)-M06-2X/TZ2P//BP86/TZ2P

E = -5676.98

G = -5575.14

N_{imag} = 0

C	4.33423159	-0.53793785	0.70684800
H	5.42193334	-0.49927838	0.55764956
H	4.09158113	-1.32070530	1.43236224
N	3.17434445	0.71767275	-0.85990022
C	2.19738854	2.44553985	-2.32620125
C	1.66580912	2.80865037	-3.51351194
H	2.34833093	3.13943191	-1.50290022
H	1.55950485	2.02205671	-4.26639387
C	1.22063383	4.13882527	-3.90710202
C	0.73022457	4.32691773	-5.21472887
C	1.25654194	5.25222938	-3.04157207
C	0.29624451	5.57831556	-5.64677289
H	0.69557342	3.47303643	-5.89277339
C	0.82163423	6.49971891	-3.47336998
H	1.62594901	5.13507202	-2.02325481
C	0.34065016	6.66939372	-4.77723131
H	-0.07825617	5.70279456	-6.66276086
H	0.85523824	7.34944111	-2.79148158
H	0.00110092	7.64994800	-5.11075465
C	2.59480274	1.04694144	-2.11057958
O	2.47649510	0.14278462	-2.93520896
C	3.32292813	1.50115473	0.29642466
O	3.00111489	2.65009104	0.48669276
O	3.91395775	0.73236861	1.26479443
C	3.56792185	-0.66966500	-0.61409608
H	4.18105084	-1.04828747	-1.43854129
H	2.67748770	-1.30872576	-0.53200214

9. References

(1) Koelsch, C. F.; Stephens Jr, C. R. The Internal Michael Reaction. II. Formation of Arylated Coumarans, of an Indoline, a Dihydrothionaphthene and a Hydrocarbostyril. *J. Am. Chem. Soc.* **1950**, *72*, 5, 2209–2212.

(2) Andersen, T. P.; Ghattas, A.-B. A. G.; Lawesson, S.-O. Studies on Amino Acids and Peptides—IV: 1,2,4-Triazines from Thioacylated Amino-Acid Esters. *Tetrahedron* **1983**, *39*, 20, 3419–3427.

(3) Kono, M.; Harada, S.; Nemoto, T. Chemoselective Intramolecular Formal Insertion Reaction of Rh–Nitrenes into an Amide Bond Over C−H Insertion. *Chem.-Eur. J.* **2019**, *25*, 3119–3124.

(4) Lauzon, S.; Keipour, H.; Gandon, V.; Ollevier, T. Asymmetric Fe^{II}-Catalyzed Thia-Michael Addition Reaction to *α,β*-Unsaturated Oxazolidin-2-one Derivatives. *Org. Lett.* **2017**, *19*, 6324–6327.

(5) Padwa, A.; Haffmanns, G.; Tomas, M. Generation of Azomethine Ylides via the Desilylation Reaction of Immonium Salts. *J. Org. Chem.* **1984**, *49*, 3314–3322.

(6) Luca Schwarz, J.; Kleinmans, R.; Paulisch, T. O.; Glorius, F. 1,2-Amino Alcohols via Cr/Photoredox Dual-Catalyzed Addition of α-Amino Carbanion Equivalents to Carbonyls. *J. Am. Chem. Soc.* **2020**, *142*, 2168–2174.

(7) Felluga, F.; Pitacco, G.; Visintin, C.; Valentin, E. Synthesis of Polysubstituted Pyrrolizidines from Proline Derivatives and Conjugated Nitroolefins. *Helv. Chimica Acta* **1997**, *80*, 1457–1472.

(8) Cry, D. J. St.; Arndtsen, B. A. A New Use of Wittig-Type Reagents as 1,3-Dipolar Cycloaddition Precursors and in Pyrrole Synthesis. *J. Am. Chem. Soc.* **2007**, *129*, 12366–12367.

(9) (a) te Velde, G.; Bickelhaupt, F. M.; Baerends, E. J.; Fonseca Guerra, C.; van Gisbergen, S. J. A.; Snijders, J. G.; Ziegler, T. Chemistry with ADF. *J. Comput. Chem.* **2001**, *22*, 931–967. (b) Fonseca Guerra, C.; Snijders, J. G.; te Velde, G.; Baerends, E. J. Towards an Order-N DFT Method. *Theor. Chem. Acc.* **1998**, *99*, 391–403. (c) ADF2018.105, SCM Theoretical Chemistry, Vrije Universiteit: Amsterdam (Netherlands). http://www.scm.com.

(10) (a) Becke, A. D. Density-functional Exchange-energy Approximation with Correct Asymptotic Behavior. *Phys. Rev. A,* **1988**, *38*, 3098–3100. (b) Perdew, J. P., Density-functional Approximation

for the Correlation Energy of the Inhomogeneous Electron Gas. *Phys. Rev. B: Condens. Matter Mater. Phys.* **1986**, *33*, 8822–8824.

(11) van Lenthe, E.; Baerends, E. J. Optimized Slater-type Basis Sets for the Element 1–118. *J. Comput. Chem.* **2003**, *24*, 1142–1156.

(12) (a) Talbot, A.; Devarajan, D; Gustafson, S. J.; Fernández, I.; Bickelhaupt, F. M.; Ess, D. H. Activation-Strain Analysis Reveals Unexpected Origin of Fast Reactivity in Heteroaromatic Azadiene Inverse-Electron-Demand Diels–Alder Cycloadditions. *J. Org. Chem.* **2015**, *80*, 548–558. (b) Hamlin, T. A.; Svatunek, D.; Yu, S.; Ridder, L.; Infante, I.; Visscher, L.; Bickelhaupt, F. M. Elucidating the Trends in Reactivity of Aza-1,3-Dipolar Cycloadditions. *Eur. J. Org. Chem.* **2019**, 378–386. (c) Yu, S.; de Bruijn, H. M.; Svatunek, D.; Hamlin, T. A.; Bickelhaupt, F. M. Factors Controlling the Diels−Alder Reactivity of Hetero-1,3-Butadienes. *ChemistryOpen* **2018**, *7*, 995–1004.

(13) Legault, C. Y. CYLview (Unversité de Sherbrooke: Sherbrooke, QC, Canada, 1.0b, 2009) http://www.cylview.org.

(14) Zhao, Y.; Truhlar, D. The M06 suite of density functionals for main group thermochemistry, thermochemical kinetics, noncovalent interactions, excited states, and transition elements: two new functionals and systematic testing of four M06-class functionals and 12 other functionals. *Theor. Chem. Acc.* **2008**, *120*, 215–241.

(15) (a) Klamt, A.; Schüürmann, G. COSMO: a new approach to dielectric screening in solvents with explicit expressions for the screening energy and its gradient. *J. Chem. Soc. Perkin Trans. 2* **1993**, 799–805. (b) Klamt, A. Conductor-like Screening Model for Real Solvents: A New Approach to the Quantitative Calculation of Solvation Phenomena. *J. Phys. Chem.* **1995**, *99*, 2224–2235. (c) Klamt, A.; Jonas, V. Treatment of the outlying charge in continuum solvation models. *J. Chem. Phys.* **1996**, *105*, 9972–9981. (d) Pye, C. C.; Ziegler, T. An implementation of the conductor-like screening model of solvation within the Amsterdam density functional package. *Theor. Chem. Acc.* **1999**, *101*, 396–408.

(16) For a step-by-step protocol, see: (a) Vermeeren, P.; van der Lubbe, S. C. C.; Fonseca Guerra, C.; Bickelhaupt, F. M.; Hamlin, T. A. Understanding Chemical Reactivity Using the Activation Strain Model. *Nat. Protoc.* **2020**, *15*, 649–667. for reviews, see: (b) Bickelhaupt, F. M. Understanding

Reactivity with Kohn-Sham Molecular Orbital Theory: E2–SN2 Mechanistic Spectrum and Other Concepts. *J. Comp. Chem.* **1999**, *20*, 114–128. (c) van Zeist, W.-J.; Bickelhaupt, F. M. The Activation Strain Model of Chemical Reactivity. *Org. Biomol. Chem.* **2010**, *8*, 3118−3127. (d) Fernández, I.; Bickelhaupt, F. M. The Activation Strain Model and Molecular Orbital Theory: Understanding and Designing Chemical Reactions. *Chem. Soc. Rev.* **2014**, *43*, 4953–4967. (e) Wolters, L. P.; Bickelhaupt, F. M. The Activation Strain Model and Molecular Orbital Theory. *WIRES Comput. Mol. Sci.* **2015**, *5*, 324–343. (f) Bickelhaupt, F. M.; Houk, K. N. Analyzing Reaction Rates with the Distortion/Interaction-Activation Strain Model. *Angew. Chem.* **2017**, *129*, 10204–10221; *Angew. Chem. Int. Ed.* **2017**, *56*, 10070–10086.

(17) For an overview of the EDA method, see: (a) Hamlin, T. A.; Vermeeren, P.; Fonseca Guerra, C.; Bickelhaupt, F. M. Energy Decomposition Analysis in the Context of Quantitative Molecular Orbital Theory. In Complementary Bonding Analysis; Grabowsky, S. Eds.; De Gruyter: Berlin, **2021**, 199–212. For a detailed overview of the EDA method, see: (b) Bickelhaupt, F. M.; Baerends, E. J. Kohn-Sham Density Functional Theory: Predicting and Understanding Chemistry. In Reviews in Computational Chemistry; Lipkowitz, K. B.; Boyd, D. B., Eds.; Wiley-VCH: New York, **2000**; 15, 1−86.
